# Rapid Synthesis and Antiviral Activity of (Quinazolin-4-Ylamino)Methyl-Phosphonates Through Microwave Irradiation

**DOI:** 10.3390/ijms13066730

**Published:** 2012-06-01

**Authors:** Hui Luo, Deyu Hu, Jian Wu, Ming He, Linhong Jin, Song Yang, Baoan Song

**Affiliations:** State Key Laboratory Breeding Base of Green Pesticide and Agricultural Bioengineering/Key Laboratory of Green Pesticide and Agricultural Bioengineering, Ministry of Education, Guizhou University, Guiyang 550025, Guangzhou, China; E-Mails: luohui8732@163.com (H.L.); fcc.dyhu@gzu.edu.cn (D.H.); jianwu2691@yahoo.com.cn (J.W.); hmher@126.com (M.H.); fcc.jinlh@gzu.edu.cn (L.J.)

**Keywords:** quinazoline derivatives, α-aminophosphonate moiety, synthesis, microwave irradiation

## Abstract

This study describes the simple synthesis of new (quinazolin-4-ylamino) methylphosphonates via microwave irradiation. Substituted-2-aminobenzonitrile reacted with 1,1-dimethoxy-*N,N*-dimethylmethanamine at a reflux condition to obtain *N*′-(substituted-2-cyanophenyl)-*N*,*N*-dimethylformamidine (1). The subsequent reaction of this intermediate product with α-aminophosphonate (2) in a solution containing glacial acetic acid in 2-propanol through microwave irradiation resulted in the formation of (quinazolin-4-ylamino)methyl-phosphonate derivatives 3a to 3x, which were unequivocally characterized by the spectral data and elemental analysis. The influence of the reaction conditions on the yield of 3a was investigated to optimize the synthetic conditions. The relative optimal conditions for the synthesis of 3a include a 1:1 molar ratio of *N*′-(2-cyanophenyl)-*N*,*N*-dimethylformamidine to diethyl amino(phenyl)methylphosphonate and a 4:1 volume ratio of isopropanol to HOAc in the solvent mixture, at a reaction temperature of 150 °C, with a microwave power of 100 W and a corresponding pressure of 150 psi for 20 min in the microwave synthesizer. The yield of 3a was approximately 79%, whereas those of 3b to 3x were approximately 77% to 86%. Some of the synthesized compounds displayed weak to good anti-Tobacco mosaic virus (TMV) activity.

## 1. Introduction

4-Aminoquinazoline derivatives, an important class of heterocyclic compounds [1], have received wide attention due to their anticancer [2–4], antimalarial [5], antimicrobial [6], and antiviral activities [7,8]. In addition, these derivatives can act as selective inhibitors of tyrosine kinase [9,10]. Suitably substituted derivatives of α-aminophosphonates exhibit a wide range of bioactivities [11–15], thus incorporating this pharmacaphore into the parent quinazoline unit may elicit synergistic effects in the design of lead structures with potent antiviral activity for plants. Based on this concept, we planned to simultaneously synthesize novel compounds containing α-aminophosphonates and chloroquinazolines (**3**). A classic, well-known method for synthesis of **3** is through a conventional nucleophilic reaction involving α-aminophosphonates and chloroquinazolines (Scheme I). This nucleophilic reaction offers the advantage of cheap and available materials with mild reaction conditions. However, it requires long reaction times, poor yield, and the sensitive nature of 4-chloroquinazoline, accompanied by a complicated route to access the desired product through the classic mode, has motivated us to investigate alternative methodologies [16].

Microwave heating has been developed as a possible alternative to conventional heating with the potential to emerge as the preferred heating mode in organic synthesis [17–20]. In this perspective, microwave heating has an edge over the traditional method both in medicinal and high-throughput chemistry applications because it can offer an expanded reaction range and diminished reaction times from several hours or days down to minutes. Microwave heating is the result of the molecular rotation of materials that contain polar molecules with an electrical dipole moment. In the presence of an oscillating electromagnetic field, rotating molecules continuously collide with one another, thereby distributing the energy to adjacent molecules and causing an increase in temperature [21–23]. This kind of dipole rotation, normally referred to as dielectric heating, is usually observed in liquid water and other polar solvents placed in the microwave oven, but much less on fats, sugars, and frozen water [24–26]. Microwave irradiation has attracted the attention of the scientific community as a powerful tool for rapid, green, and efficient synthesis of a variety of quinazoline compounds [27–29]. In view of the advantages and disadvantages of the classical synthetic methods, we followed a cyclic method under microwave irradiation as illustrated in Scheme II. Title compounds (**3a**–**3x**) can easily be synthesized in a solvent of isopropanol and acetic acid (v/v, 4:1) using *N*′-(substituted-2-cyanophenyl)-*N*,*N*-dimethyl-formamidine derivatives and dialkyl amino (phenyl) methylphosphonate as material under microwave irradiation (100 °C, 100 psi) for 20 min. The new method requires short reaction times; it is very easy, mild, and environmentally friendly. To the best of our knowledge, this is the first report on the synthesis of new quinazoline compounds containing α-aminophosphonate moieties using microwave irradiation. This study aims to optimize the reaction conditions of each step and supply useful data for a one-pot reaction. Preliminary antiviral activity tests showed that some compounds exhibited *in vivo* antiviral activities against tobacco mosaic virus (TMV) at 500 mg/L. Among them, compounds **3p**, **3v** exhibited slightly similar activities compared to the commercial agent Ningnanmycin.

## 2. Results

### 2.1. Chemistry

Microwave technology was applied to the synthetic reaction to shorten the reaction time and increase the yields of (quinazolin-4-ylamino)methylphosphonate derivatives (**3**). As expected, when the mixture of formamidine (obtained from 2-amino-benzonitrile) (**1**) and α-aminophosphonate (**2**) in ethanol was irradiated with microwaves, the reaction was completed in 25 min with higher yields compared with the conventional mode of heating. We optimized the reaction parameters for the synthesis of compound **3a** as a model under different conditions. The influence of the reaction times and reaction temperatures with or without microwave irradiation and the role of microwave power were investigated. Table 1 indicates that microwave irradiation can accelerate the reaction (Table 1, entry 1 to 16). The reaction was relatively slow and only 34.0% yield was obtained within 6 h when microwave irradiation was not applied (Table 1, entry 12). The effect of three different mixture solvents was studied. Table 1 also shows that the reaction can yield 59.0% of the product in the mixture solvent containing isopropanol/acetic acid (v/v, 4:1), whereas in the other solvents, the yield of **3a** was significantly lower, particularly in the mixture solvent containing acetonitrile/acetic acid (v/v, 4:1), which only produced 40.1% yield. This result indicated the superior efficiency of isopropanol/acetic acid compared with other mixture solvents (Table 1, entries 1 to 3). The yield of **3a** increased from 45.1% and 59.0% to 62.5% (Table 1, entries 1, 5, and 6) when the reaction time was prolonged from 10 and 20 min to 30 min at 100 °C and 100 psi. When the reaction time was further prolonged to 30 min, the yield of **3a** (62.5%, entry 6) was slightly increased compared with that produced after 20 min. A decrease in the yield was observed at lower temperatures (Table 1, entries 1 and 7) compared with that at 150 °C (Table 1, entry 5), and a slight improvement was noted when the reaction system was heated to 170 °C (Table 1, entry 11). In addition, the effect of microwave power and pressure on the reaction was also studied. Compound **3a** was obtained with 43.0%, 70.0%, and 79.0% yields, when the microwave power was increased from 60 W to 80 and 100 W with the corresponding pressure of 100, 100, and 150 psi, respectively (Table 1, entries 10 and 11). Hence, the reaction should be carried out at 100 W and 150 psi to yield better results than at a different power output or pressure.

The optimized reaction conditions were exploited with starting materials containing different substituents and resulted in excellent yields. The results are listed in Table 2. This indicates the versatility of the reported method. The proposed mechanism shown in Scheme III can account for the role of acetic acid in the reaction. Protonation of nitrogen by acetic acid initially activates the electrophilic carbon of the nitrile group toward a nucleophilic attack by the phosphonate amine. This reaction is followed by an increase in the electrophilic reactivity of the imine carbon on the formamidine group due to the possible protonation of nitrogen and the subsequent expulsion of a secondary amine. The intramolecular attack by the nitrogen at the electrophilic center leads to the formation of the desired cyclic product.

### 2.2. Antiviral Activity Bioassay

The antiviral activities of compounds **3a**–**x** against TMV were assayed by the reported method [30]. The results of the *in vivo* bioassay against TMV are given in Table 3. Ningnanmycin was used as a reference antiviral agent. The data provided in Table 3 indicate that the title compounds **3a**–**x** showed curative rates ranging from 30.1%–52.0%. Among them, compounds **3p**, **3v** exhibited slightly similar activities compared to the commercial agent Ningnanmycin.

## 3. Experimental Section

### 3.1. General

Unless otherwise stated, all the reagents and reactants were purchased from commercial suppliers; the melting points were uncorrected and were determined on a XT-4 binocular microscope (Beijing Tech Instrument Co., China). ^1^H NMR and ^13^C NMR spectra were obtained using a JEOL ECX 500 NMR spectrometer (Tokyo, Japan) at room temperature, operating at 500 MHz for ^1^H NMR, 125 MHz for ^13^C NMR, and 470 MHz for ^19^F NMR, using CDCl_3_ as solvents and tetramethylsilane as an internal standard. Infrared spectra were obtained using KBr on a Bruker VECTOR 22 spectrometer (Ettlingen, Germany). Microwave irradiations were carried out using Discovery™ LabMate microwave apparatus. Elemental analysis was performed on the Elemental Vario-III CHN analyzer (Hanau, Germany). The course of the reactions was monitored through TLC; analytical TLC was performed on silica gel GF_254_; column chromatographic purification was carried out using silica gel. The intermediate products, including diethyl amino(phenyl)methylphosphonate (**2a**), di-*n*-propyl amino(phenyl)methylphosphonate (**2b**), diisopropyl amino(phenyl)methylphosphonate (**2c**), diethyl amino(2-fluorophenyl)methylphosphonate (**2d**), di-*n*-propyl amino(2-fluorophenyl)methylphosphonate (**2e**), and diisopropyl amino (2-fluorophenyl)methylphosphonate (**2f**) were prepared according to a previously described procedure [31].

### 3.2. Preparation of the Intermediates *N*′-(2-cyanophenyl)-*N*,*N*-Dimethylformamidine (**1a** to **1d**)

The substituted-2-aminobenzonitrile (10 mmol) and 1,1-dimethoxy-*N*,*N-*dimethylmethanamine (23.8 g, 20 mmol) were placed in a dried, round-bottom flask containing a magnetic stir bar. The reaction mixture was refluxed for 3 h, and then cooled to room temperature. Water (20 mL) was then added and the resulting mixture was stirred for 10 min. The mixture was subsequently filtered and recrystallized from methanol to obtain pure *N′*-(substituted-2-cyanophenyl)-*N*,*N*-dimethylformamidine **1**.

*N′-(2-cyanophenyl)-N,N-dimethylformamidine* (**1a**): White crystal, yield 90.8%; mp 101 °C to 103 °C; ^1^H NMR (CDCl_3_, 500 MHz): δ 8.66 (s, 1H, CH), 7.49 to 7.66 (m, 4H, Ar-3,4,5,6-H), 3.32 (s, 6H, 2CH_3_); ^13^C NMR (CDCl_3_, 125 MHz): δ 156.53, 141.06, 135.16, 134.15, 128.02, 125.43, 116.92, 107.69, 40.86, 40.19; IR (KBr): ν 2949.16, 2709.99, 2227.78, 1701.22, 1597.06, 1338.60, 1134.14, 1058.92, 769.60, 532.35 cm^−1^; *Anal.* Calcd for C_10_H_11_N_3_: C 69.34%, H 6.40%, N 24.26%; Found. C 69.51%, H 6.23%, N 24.41%.

*N′-(2-cyano-4-nitrophenyl)-N,N-dimethylformamidine* (**1b**): White crystal, yield 91.4%; mp 137 °C to 139 °C; ^1^H NMR (CDCl_3_, 500 MHz): δ 8.42 (s, 1H, Ar-3-H), 8.23 to 8.25 (m, 1H, Ar-5-H), 7.79 (s, 1H, CH), 7.00 (d, 1H, *J* = 10.0 Hz, Ar-6-H), 3.19 (s, 6H, 2CH_3_); ^13^C NMR (CDCl_3_, 125 MHz): δ 156.54, 135.16, 134.46, 128.15, 125.59, 116.89, 107.71, 43.72, 40.01, 38.09; IR (KBr): ν 2223.92, 1625.99, 1595.13, 1330.88, 1109.07, 829.39, 501.49 cm^−1^; *Anal.* Calcd for C_10_H_10_N_4_O_2_: C 55.04%, H 4.62%, N 25.68%; Found. C 55.11%, H 4.73%, N 26.51%.

*N′-(2-cyano-4-fluorophenyl)-N,N-dimethylformamidine* (**1c**): White crystal, yield 92.2%; mp 83 °C to 85 °C; ^1^H NMR (CDCl_3_, 500 MHz): δ 8.43 (s, 1H, Ar-3-H), 8.24 to 8.26 (m, 1H, Ar-5-H), 7.77 (s, 1H, CH), 7.02 (d, 1H, *J* = 5.0 Hz, Ar-6-H), 3.18 (s, 6H, 2CH_3_); ^13^C NMR (CDCl_3_, 125 MHz): δ 160.29 (d, ^1^*J*_CF_ = 246.4 Hz), 154.41, 141.39, 129.61 (d, ^2^*J*_CF_ = 23.8 Hz), 128.69 (d, ^2^*J*_CF_ = 22.6 Hz), 118.81(d, ^3^*J*_CF_ = 7.8 Hz), 107.28 (d, ^3^*J*_CF_ = 7.4 Hz), 77.16, 40.98, 35.19; IR (KBr): ν 2223.92, 1627.92, 1502.55, 1332.81, 1172.72, 829.39, 520.78 cm^−1^; *Anal.* Calcd for C_10_H_10_FN_3_: C 62.82%, H 5.27%, N 21.98%; Found. C 62.56%, H 5.08%, N 21.95%.

*N′-(2,4-dichloro-6-cyanophenyl)-N,N-dimethylformamidine* (**1d**): White crystal, yield 89.7%; mp 153 °C to 155 °C; ^1^H NMR (CDCl_3_, 500 MHz): δ 8.42 (s, 1H, Ar-3-H), 8.24 to 8.26 (m, 1H, Ar-5-H), 7.78 (s, 1H, CH), 3.19 (s, 6H, 2CH_3_); ^13^C NMR (CDCl_3_, 125 MHz): δ 160.29, 154.41, 141.39, 129.61, 128.69, 118.81, 107.28, 77.16, 40.98, 35.19; IR (KBr): ν 2358.94, 1627.92, 1502.55, 1311.59, 1109.57, 889.18, 520.79 cm^−1^; *Anal.* Calcd for C_10_H_9_Cl_2_N_3_: C 49.61%, H 3.75%, N 17.36%; Found. C 49.46%, H 3.83%, N 17.05%.

### 3.3. Preparation of Title Compounds **3a–3x**

The intermediates *N′*-(2-cyanophenyl)-*N*,*N*-dimethylformamidine **1** (0.5 mmol) and diethyl amino(phenyl)methylphosphonate (0.5 mmol) were added to a 10 mL microwave reaction vial equipped with a magnetic stir bar. Glacial acetic acid [1 mL; 20% (v/v)] solution in 2-propanol (4 mL) was also added. The mixture was capped and irradiated in the microwave for 20 min at 150 °C then cooled to ambient temperature and purified in parallel using the following procedure. The contents of the vial were poured into single-mouth bottles (50 mL). The reaction vessel was washed with 2-propanol (3 mL × 5 mL), and then 2-propanol was poured into the single-mouth bottles. The solvent was concentrated under reduced pressure, the solid was obtained, and the crude product was purified then preparative TLC with a mixture of petroleum ether and ethyl acetate (v/v = 1:1) as the developing solvent to yield the title compounds **3a** to **3x**.

*Diethyl phenyl(quinazolin-4-ylamino)methylphosphonate* (**3a**): White solid, yield 78.5%; mp 201 °C to 203 °C; ^1^H NMR: (CDCl_3_, 500 MHz): δ 8.65 (s, 1H, quinazoline-2-H), 7.95 (d, *J* = 8.0 Hz, 1H, quinazoline-8-H), 7.86 (d, *J* = 8.6 Hz, 1H, quinazoline-5-H), 7.76 to 7.78 (m, 1H, Ar-4-H), 7.51 to 7.61 (m, 2H, quinazoline-6,7-H), 7.26 to 7.37 (m, 4H, Ar-2,3,5,6-H), 6.84 (s, 1H, N-H), 6.12 (d, *J* = 10.0 Hz, 1H, CH), 3.97 to 4.15 (m, 4H, 2CH_2_), 1.11 to 1.25 (m, 6H, 2CH_3_ ); ^13^C NMR (CDCl_3_, 125 MHz): δ 155.05, 133.04, 128.78, 128.65, 128.35, 128.28, 128.23, 126.46, 120.90, 77.36, 77.11, 76.86, 63.77, 63.71, 63.29, 52.01, 50.79, 16.48,16.28; ^31^P NMR(CDCl_3_, 500 MHz): δ 22.7; IR: ν 3296.76 (NH), 3070.14 (ArH), 1779.78 (CN), 1577.65 (Ar), 1206.79 (P=O), 987.02 (P-O-C) cm^−1^; *Anal*. Calcd for C_19_H_22_N_3_O_3_P: C 61.45%, H 5.97%, N 11.31%; Found. C 61.05%, H 5.93%, N 10.99%.

*Di-n-propyl phenyl(quinazolin-4-ylamino)methylphosphonate* (**3b**): White solid, yield 78.0%; mp 173 °C to 175 °C; ^1^H NMR (CDCl_3_, 500 MHz): δ 8.65 (s, 1H, quinazoline-2-H), 7.98 d, *J* = 8.0 Hz, 1H, quinazoline-8-H), 7.84 (d, *J* = 8.1 Hz, 1H, quinazoline-5-H), 7.74 to 7.77 (m, 1H, Ar-4-H), 7.61 to 7.63 (m, 2H, quinazoline-6,7-H), 7.26 to 7.34 (m, 4H, Ar-2,3,5,6-H), 6.79 (s, 1H, N-H), 6.18 (d, *J* = 20.0 Hz, 1H, CH), 3.63 to 4.07 (m, 4H, 2OCH_2_), 1.47 to 1.61 (m, 4H, 2CH_2_), 0.78 to 0.83 (m, 6H, 2CH_3_); ^13^C NMR (CDCl_3_, 125 MHz): δ 158.66, 158.59, 155.02, 149.69, 135.46, 132.84, 128.65, 128.61, 128.27, 128.22, 126.24, 120.95, 68.93, 68.60, 51.88, 50.65, 23.70, 23.66, 9.90, 9.82; ^31^P NMR(CDCl_3_, 500 MHz): δ 22.6; IR: ν 3263.64 (NH), 2968.56 (ArH), 1714.78 (CN), 1579.72 (Ar), 1238.36 (P=O), 1012.24 (P-O-C) cm^−1^; *Anal*. Calcd for C_21_H_26_N_3_O_3_P: C 63.15%, H 6.56%, N 10.52%; Found. C 63.19%, H 6.22%, N 10.63%.

*Diisopropyl phenyl(quinazolin-4-ylamino)methylphosphonate* (**3c**): White solid, yield 78.1%; mp 188 °C to 190 °C; ^1^H NMR (CDCl_3_, 500 MHz): δ 8.65 (s, 1H, quinazoline-2-H), 8.00 (d, *J* = 8.6 Hz, 1H, quinazoline-8-H), 7.85 (d, *J* = 8.0 Hz, 1H, quinazoline-5-H), 7.73 to 7.77 (m, 1H, Ar-4-H), 7.47 to 7.50 (m, 2H, quinazoline-6,7-H), 7.26 to 7.48 (m, 5H, Ar-2,3,5,6-H), 7.02 (s, 1H, N-H), 6.10 (d, *J* = 9.2 Hz, 1H, CH), 4.47 to 4.79 (m, 2H, 2CH), 0.89 to 1.31 (m, 12H, 4CH_3_); ^13^C NMR (CDCl_3_, 125 MHz): δ 158.77, 158.70, 155.10, 149.68, 132.78, 128.57, 128.52, 128.46, 128.41, 128.05, 126.21, 120.95, 71.99, 58.34, 52.48, 51.24, 24.28, 24.26, 24.16, 24.13; ^31^P NMR(CDCl_3_, 200 MHz): δ 20.9; IR: ν 3275.15 (NH), 2980.07 (ArH), 1620.24 (CN), 1577.85 (Ar), 1238.36 (P=O), 987.68 (P-O-C) cm^−1^; *Anal*. Calcd for C_21_H_26_N_3_O_3_P: C 63.15%, H 6.56%, N 10.52%; Found. C 63.04%, H 6.36%, N 10.62%.

*Diethyl(2-fluorophenyl)(quinazolin-4-ylamino)methylphosphonate* (**3d**): White solid, yield 77.8%; mp 163 °C to 165 °C; ^1^H NMR (CDCl_3_, 500 MHz): δ 8.65 (s, 1H, quinazoline-2-H), 7.95 (d, *J* = 10.0 Hz, 1H, Ar-3-H), 7.85 (d, *J* = 15.0 Hz, 1H, quinazoline-8-H), 7.76 to 7.79 (m, 1H, quinazoline-5-H), 7.51 to 7.61 (m, 2H, quinazoline-6,7-H), 7.26 to 7.38 (m, 4H, Ar-4, 5, 6-H), 6.84 (s, 1H, NH), 6.16 (d, *J* = 10.0 Hz, 1H, CH), 4.15 to 4.17 (m, 2H, CH_2_), 3.98 to 4.14 (m, 2H, CH_2_), 1.11 to 1.27 (m, 6H, 2CH_3_); ^13^C NMR (CDCl_3_, 125 MHz): δ 161.92, 159.94, 158.67 (d, ^1^*J*_CF_ = 246.5 Hz), 155.15, 149.78, 133.05, 129.57 (d, ^2^*J*_CF_ = 23.8 Hz), 128.75 (d, ^3^*J*_CF_ = 7.7 Hz), 126.46 (d, ^3^*J*_CF_ = 7.6 Hz), 124.53, 121.15, 115.92 (d, ^2^*J*_CF_ = 24.2 Hz), 115.13, 63.84, 63.77, 46.49, 45.14, 16.43, 16.26; ^31^P NMR (CDCl_3_, 200 MHz): δ 21.7; IR: ν 3304.06 (NH), 2987.74 (CH_3_), 1577.74, 1525.69, 1409.96 (C=C), 1361.74 (CH_3_), 1234.44 (P=O), 1228.2 (P-O-C), 804.32, 769.60 (=C–H) cm^−1^; *Anal*. Calcd for C_19_H_21_FN_3_O_3_P: C 58.61%; H, 5.44%, N 10.79%; Found. C 58.42%, H 5.33%, N 10.58%.

*Di-n-propyl(2-fluorophenyl)(quinazolin-4-ylamino)methylphosphonate* (**3e**): White solid, yield 77.5%; mp 111 °C to 113 °C; ^1^H NMR (DMSO-*d*_6_, 500 MHz): δ 9.05 (d, *J* = 6.0 Hz, 2H, quinazoline-2-H, Ar- 3-H), 7.85 to 8.10 (m, 4H, quinazoline-5,6,7,8-H), 7.35 (s, 1H, NH), 7.31 to 7.33 (m, 3H, Ar-4,5,6-H), 6.85 (d, *J* = 20.0 Hz, CH), 3.98 to 4.02 (m, 4H, 2-OCH_2_), 1.49 to 1.51 (m, 4H, 2-CH_2_), 1.46 to 1.51 (m, 4H, 2CH_2_), 0.74 to 0.78 (m, 3H, *J* = 7.5 Hz, CH_3_), 0.73 to 0.75 (m, H, 2CH_3_), 0.71 (t, 3H, *J* = 7.5 Hz, CH_3_); ^13^C NMR (DMSO-*d*_6_, 125 MHz): δ 161.30, 151.85 (d, ^1^*J*_CF_ = 245.8 Hz), 136.64, 131.53, 131.15, 128.92 (d, ^2^*J*_CF_ =24.2 Hz), 125.34 (d, ^3^*J*_CF_ = 7.5 Hz), 125.15 (d, ^3^*J*_CF_ = 7.5 Hz), 121.87, 121.76, 121.12, 115.91 (d, ^2^*J*_CF_ =23.6 Hz), 115.57, 113.70, 68.71, 68.65, 45.74, 23.88, 23.84, 10.24, 10.19; ^31^P NMR(DMSO-*d*_6_, 200 MHz): δ 19.5; IR: ν 3294.05 (NH), 2977.74 (CH_3_), 1571.17, 1520.61, 1412.12 (C=C) 1360.12 (CH_3_), 1234.44 (P=O), 1228.2 (P-O-C) cm^−1^; *Anal*. Calcd for C_21_H_25_FN_3_O_3_P: C 60.43%, H 6.04%, N 10.07%; Found. C 60.14%, H 6.16%, N 10.42%.

*Diisopropyl (2-fluorophenyl)(quinazolin-4-ylamino)methylphosphonate* (**3f**): White solid, yield 77.5%; mp 111 °C to 113 °C; ^1^H NMR (DMSO-*d*_6_, 500 MHz): δ 9.05 (d, *J* = 6.0 Hz, 2H, quinazoline-2-H, Ar- 3-H), 7.85 to 8.10 (m, 4H, quinazoline-5,6,7,8-H), 7.35 (s, 1H, NH), 7.31 to 7.33 (m, 3H, Ar-4,5,6-H), 6.85 (d, *J* = 20.0 Hz, CH), 3.98 to 4.02 (m, 4H, 2-OCH_2_), 1.49 to 1.51 (m, 4H, 2-CH_2_), 1.46 to 1.51 (m, 4H, 2CH_2_), 0.74 to 0.78 (m, 3H, *J* = 7.5 Hz, CH_3_), 0.73 to 0.75 (m, H, 2CH_3_), 0.71 (t, 3H, *J* = 7.5 Hz, CH_3_); ^13^C NMR (DMSO-*d*_6_, 125 MHz): δ 161.30, 151.85 (d, ^1^*J*_CF_ = 246.5 Hz), 136.64, 131.53, 131.15, 128.92 (d, ^2^*J*_CF_ = 24.6 Hz), 125.34 (d, ^3^*J*_CF_ = 7.5 Hz), 125.15 (d, ^3^*J*_CF_ = 7.5 Hz), 121.87, 121.76, 121.12, 115.91 (d, ^2^*J*_CF_ = 23.6 Hz), 115.57, 113.70, 68.71, 68.65, 45.74, 23.88, 23.84, 10.24, 10.19; ^31^P NMR(DMSO-*d*_6_, 200 MHz): δ 19.5; IR: ν 3294.05 (NH), 2977.74 (CH_3_), 1571.17, 1520.61, 1412.12 (C=C) 1360.12 (CH_3_), 1234.44 (P=O), 1228.2 (P-O-C) cm^−1^; *Anal*. Calcd for C_21_H_25_FN_3_O_3_P: C 60.43%, H 6.04%, N 10.07%; Found. C 60.14%, H 6.16%, N 10.42%.

*Diethyl (6-nitroquinazolin-4-ylamino)(phenyl)methylphosphonate* (**3g**): White solid, yield 85.9%; mp 132 °C to 134 °C; ^1^H NMR (CDCl_3_, 500 MHz): δ 8.62 (s, 1H, quinazoline-2-H), 8.30 (s, 1H, quinazoline-5-H), 8.05 (s, 1H, NH), 7.70 to 7.74 (m, 2H, quinazoline-7,8-H), 7.26~7.69 (m, 5H, Ar-2,3,4,5,6-H), 6.36 (d, *J* = 10 Hz, CH), 3.85 to 4.23 (m, 4H, 2-CH_2_), 1.16 to 1.24 (m, 6H, 2CH_3_); ^13^C NMR (CDCl_3_, 125 MHz): δ 158.46, 1H, 155.20, 135.24, 133.60, 131.47, 129.93, 128.66, 128.19, 121.55, 116.20, 119.13, 98.37, 63.72, 52.01, 50.81, 16.52, 16.47; ^31^P NMR(CDCl_3_, 200 MHz): δ 22.1; IR: ν 3435.28 (NH), 3134.83 (ArH), 1713.74 (CN), 1571.31 (Ar), 1227.79 (P=O), 1001.53 (P-O-C) cm^−1^; *Anal*. Calcd for C_19_H_21_N_4_O_5_P: C 54.81%, H 5.08%, N 13.46%; Found. C 54.62%, H 5.13%, N 13.66%.

*Dipropyl (6-nitroquinazolin-4-ylamino)(phenyl)methylphosphonate* (**3h**): Yellow solid, yield 85.7%; mp 196 °C to 198 °C; ^1^H NMR (CDCl_3_, 500 MHz): δ 8.62 (s, 1H, quinazoline-2-H), 8.73 (s, 1H, quinazoline-5-H), 8.13 (s, 1H, NH), 7.64 to 7.81 (m, 2H, quinazoline-7,8-H), 7.27 to 7.62 (m, 5H, Ar-2,3,4,5,6-H), 6.28 (d, 1H, *J* = 10 Hz, CH), 3.77 to 4.22 (m, 4H, 2-OCH_2_), 1.79 to 2.18 (m, 4H, 2CH_2_), 1.13 to 1.26 (m, 6H, 2CH_3_); ^13^C NMR (CDCl_3_, 125 MHz): δ 158.39, 158.33, 155.65, 145.42, 134.87, 133.22, 130.86, 128.68, 128.45, 128.40, 128.30, 120.09, 76.69, 63.81, 63.42, 63.36, 52.23, 50.90, 30.97, 16.44, 16.39; ^31^P NMR(CDCl_3_, 200 MHz): δ 21.6; IR: ν 3281.12 (NH), 2989.66 (–CH), 1630.35 (C=N), 1565.56, 1515.33, 1470.85, 1448.21 (Ar), 1240.13 (P=O) cm^−1^; *Anal*. Calcd for C_21_H_25_N_4_O_5_P: C 56.75%, H 5.67%, N 12.61%; Found. C 56.55%, H 5.58%, N 12.55%.

*Dipropyl (6-nitroquinazolin-4-ylamino)(phenyl)methylphosphonate* (**3i**): Yellow solid, yield 85.6%; mp 43 °C to 45 °C; ^1^H NMR (CDCl_3_, 500 MHz): δ 8.61 (s, 1H, quinazoline-2-H),7.92 (s, 1H, quinazoline-5-H),7.89 (s, 1H, NH), 7.67 to 7.68 (m, 2H, quinazoline-7,8-H), 7.25 to 7.64 (m, 5H, Ar-2,3,4,5,6-H), 6.22 (d, 1H, *J* = 10 Hz, CH), 4.58 to 4.84 (m, 4H, 2-OCH), 1.15 to 1.31 (m, 6H, 4CH_3_); ^13^C NMR (CDCl_3_, 125 MHz): δ 160.94, 158.93, 158.87, 154.56, 146.60, 135.73, 130.82, 128.77, 128.67, 128.49, 127.96, 122.35, 122.14, 106.34, 106.16, 72.52, 52.58, 51.36, 24.25, 24.18; ^31^P NMR(CDCl_3_, 200 MHz): δ 20.0; IR: ν 3277.06 (NH), 2980.02 (CH), 1614.42 (C=N), 1573.91, 1527.62, 1490.97, 1456.26(Ar), 1224.80 (P=O) cm^−1^; *Anal*. Calcd for C_21_H_25_N_4_O_5_P: C 56.75%, H 5.67%, N 12.61%; Found. C 56.61%, H 5.82%, N 12.83%.

*Diethyl (2-fluorophenyl)(6-nitroquinazolin-4-ylamino)methylphosphonate* (**3j**): Yellow solid, yield 85.2%; mp 176 °C to 178 °C; ^1^H NMR (DMSO-*d*_6_, 500 MHz): δ 9.84 (s, 1H, quinazoline-2-H), 9.68 to 9.70 (d, *J* = 8.0 Hz, 1H, Ar-3-H), 8.67 (s, 1H, quinazoline-5-H), 8.04 to 8.50 (m, 2H, quinazoline-7, 8-H), 7.8 (s, 1H, NH), 7.20 to 7.35 (m, 3H, Ar-4,5,6-H), 6.70 (d, *J* = 8.0 Hz, 1H, CH), 3.90 to 4.04 (m, 4H, 2CH_2_), 1.01 to 1.07 (m, 6H, 2CH_3_); ^13^C NMR (DMSO-*d*_6_, 125 MHz): δ 160.57, 160.51, 158.12 (d, ^1^*J*_CF_ = 246.4 Hz), 153.43, 145.03, 131.26, 129.89 (d, ^2^*J*_CF_ = 24.8 Hz), 127.36 (d, ^3^*J*_CF_ = 7.4 Hz), 125.06 (d, ^3^*J*_CF_ = 7.5 Hz), 121.82, 115.78 (d, ^2^*J*_CF_ = 23.5 Hz), 114.53, 63.48, 63.42, 44.73, 43.45, 39.98, 16.78, 16.45; ^31^P NMR(DMSO-*d*_6_, 200 MHz): δ 20.8; IR: ν 3259.70 (NH), 1575.84, 1525.69, 1490.97, 1456.26 (Ar), 2989.66 (–CH), 1622.13 (C=N), 1234.44 (P=O) cm^−1^; *Anal*. Calcd for C_19_H_20_FN_4_O_5_P: C 52.54%; H 4.64%; N 12.90%; Found. C 52.41%, H 4.59%, N 12.86%.

*Dipropyl (2-fluorophenyl)(6-nitroquinazolin-4-ylamino)methylphosphonate* (**3k**): Yellow solid, yield 85.0%; mp 168 °C to 170 °C; ^1^H NMR (CDCl_3_, 500 MHz): δ 9.30 (s, 1H, quinazoline-2-H), 8.73 to 8.75 (d, *J* = 10.0 Hz, 1H, Ar-3-H), 8.65 (s, 1H, quinazoline-5-H), 7.89 to 8.47 (m, 2H, quinazoline-7,8- H), 7.80 (s, 1H, NH), 7.09 to 7.27 (m, 3H, Ar-4,5,6-H), 6.81 (d, *J* = 15.0 Hz, 1H, CH), 4.14 to 4.19 (m, 4H, 2OCH_2_), 1.54 to 1.62 (m, 4H, 2CH_2_), 0.82 to 0.85 (m, 6H, 2CH_3_); ^13^C NMR (CDCl_3_, 125 MHz): δ 161.53, 160.16, 159.65 (d, ^1^*J*_CF_ = 245.8 Hz), 158.03, 153.25, 144.74, 130.27, 129.91 (d, ^2^*J*_CF_ = 24.8 Hz), 126.52 (d, ^3^*J*_CF_ = 7.8 Hz), 124.56 (d, ^3^*J*_CF_ = 7.5 Hz), 122.63, 119.92, 115.58 (d, ^2^*J*_CF_ = 22.8 Hz), 114.69, 69.34, 45.37, 44.09, 23.72, 9.93; ^31^P NMR(CDCl_3,_ 200 MHz): δ 20.2; IR: ν 3230.34 (NH), 1567.34, 1523.22, 1493.17, 1452.56 (Ar), 2990.05 (–CH), 1625.02 (C=N) 1230.48 (P=O) cm^−1^; *Anal*. Calcd for C_21_H_24_FN_4_O_5_P: C 54.55%, H 5.23%, N 12.12%; Found. C 54.43%, H 5.32%, N 12.20%.

*Diisopropyl(2-fluorophenyl)(6-nitroquinazolin-4-ylamino)methylphosphonate* (**3l**): Yellow solid, yield 83.9%; mp 141 °C to 142 °C; ^1^H NMR (CDCl_3_, 500 MHz): δ 9.14 (s, 1H, quinazoline-2-H), 8.74 (d, *J* = 8.5 Hz, 1H, Ar-3-H), 8.50 (s, 1H, quinazoline-5-H), 7.91 to 7.92 (m, 2H, quinazoline-7, 8-H), 7.25 (s, 1H, NH), 7.07 to 7.12 (m, 3H, Ar-4,5,6-H), 6.56 (d, *J* = 10.0 Hz, 1H, CH), 4.58 to 4.83 (m, 4H, 2OCH), 0.92 to 1.29 (m, 12H, 4CH_3_); ^13^C NMR (CDCl_3_, 125 MHz): δ 161.80, 159.80, 158.14 (d, ^1^*J*_CF_ = 246.2 Hz), 153.19, 144.87, 130.15, 129.88, 129.73 (d, ^2^*J*_CF_ = 24.5 Hz), 126.61 (d, ^3^*J*_CF_ = 7.9 Hz), 124.53 (d, ^3^*J*_CF_ = 7.6 Hz), 122.96, 119.18, 115.58 (d, ^2^*J*_CF_ = 23.5 Hz), 114.36, 73.15, 46.71, 45.43, 24.20, 24.11, 23.91, 23.17; ^31^P NMR(CDCl_3,_ 200 MHz): δ 18.8; IR: ν 3255.84 (NH), 1577.77, 1527.62, 1490.97, 1456.26 (Ar), 2981.95 (–CH), 1622.13 (C=N), 1236.37 (P=O) cm^−1^; *Anal*. Calcd For C_21_H_24_FN_4_O_5_P: C 54.55%, H 5.23%, N 12.12%; Found. C 54.62%, H 5.18%, N 11.98%.

*Diethyl (6-fluoroquinazolin-4-ylamino)(phenyl)methylphosphonate* (**3m**): White solid, yield 86.1%; mp >300 °C; ^1^H NMR (CDCl_3_, 500 MHz): δ 8.62(s, 1H, quinazoline-2-H), 7.85 (s, 1H, quinazoline-5-H), 7.66 to 7.80 (m, 2H, quinazoline-7,8-H), 7.65 (s, 1H, N-H), 7.24 to 7.51 (m, 5H, Ar-2,3,4,5,6-H), 6.24 (d, *J* = 10.0 Hz, 1H, C-H), 4.03 to 4.22 (m, 4H, 2CH_2_), 1.14 to 1.26 (m, 6H, 2CH_3_); ^13^C NMR (CDCl_3_, 125 MHz): δ 160.97 (d, ^1^*J*_CF_ = 243.8 Hz), 158.60, 154.41, 146.73, 135.24 (d, ^3^*J*_CF_ = 8.0 Hz), 128.60, 128.35, 122.46 (d, ^2^*J*_CF_ = 24.5 Hz), 122.27 (d, ^3^*J*_CF_ = 7.9 Hz), 106.09 (d, ^2^*J*_CF_ = 22.8 Hz), 77.27, 76.76, 63.74, 63.63, 63.31, 63.25, 52.00, 16.43, 16.10; ^31^P NMR(CDCl_3,_ 200 MHz): δ 22.5; IR: ν 3267.4 (NH), 3068.8 (ArH), 1629.9 (CN), 1232.7 (P=O), 1022.2 (P-O-C) cm^−1^; *Anal*. Calcd for C_19_H_21_FN_3_O_3_P: C 58.61%, H 5.44%, N 10.97%; Found. C 58.77%, H 5.63%, N 10.92%.

*Di-n-propyl(6-fluoroquinazolin-4-ylamino)(phenyl)methylphosphonate* (**3n**): White solid, yield 86.1%; mp 186 °C to 188 °C; ^1^H NMR (CDCl_3_, 500 MHz): δ 8.62 (s, 1H, quinazoline-2-H), 8.20 (s, 1H, quinazoline-5-H N-H), 7.82 to 8.10 (m, 2H, quinazoline-7,8-H), 7.80 (s, 1H, N-H), 7.23 to 7.72 (m, 5H, Ar-2,3,4,5,6-H), 6.46 (d, *J* = 8.0 Hz, 1H, C-H), 3.99 to 4.14 (m, 4H, 2OCH_2_), 1.56 to 1.57 (m, 4H, 2CH_2_), 0.78~0.85 (m, 6H, 2CH_3_); ^13^C NMR (CDCl_3_, 125 MHz): δ 160.80 (d, ^1^*J*_CF_ = 246.2 Hz), 159.05, 158.84, 154.40, 146.79, 135.38, 130.59 (d, ^3^*J*_CF_ = 8.1 Hz), 130.52, 128.67, 128.62, 128.45, 127.96, 122.02 (d, ^2^*J*_CF_ = 24.8 Hz), 106.96 (d, ^3^*J*_CF_ = 7.9 Hz), 106.78 (d, ^2^*J*_CF_ = 23.2 Hz), 68.89, 68.83, 51.86, 50.61, 23.86, 23.68; ^31^P NMR(CDCl_3,_ 200 MHz): δ 22.1; IR: ν 3261.6 (NH), 2968.5 (ArH), 1714.7 (CN), 1236.4 (P=O), 1012.2 (P-O-C) cm^−1^; *Anal*. Calcd for C_21_H_25_FN_3_O_3_P: C 60.43%, H 6.04%, N 10.07%; Found. C 60.33%, H 5.95%, N 10.12%.

*Diisopropyl (6-fluoroquinazolin-4-ylamino)(phenyl)methylphosphonate* (**3o**): White solid, yield 85.3%; mp 204 °C to 206 °C; ^1^H NMR (CDCl_3_, 500 MHz): δ 8.63 (s, 1H, quinazoline-2-H), 8.13 (s, 1H, quinazoline-5-H), 7.77 to 8.12 (m, 2H, quinazoline-7,8-H), 7.80 (s, 1H, N-H), 7.27 to 7.75 (m, 5H, Ar-2,3,4,5,6-H), 6.17 (d, *J* = 10.0 Hz, 1H, C-H), 4.57 to 4.82 (m, 2H, 2CH), 1.16 to 1.31 (m, 12H, 4CH_3_); ^13^C NMR (CDCl_3_, 125 MHz): δ 160.92 (d, ^1^*J*_CF_ = 245.4 Hz), 158.94, 158.76, 154.48, 146.76, 135.66, 130.8 (d, ^3^*J*_CF_ = 8.0 Hz), 128.69, 128.64, 128.43, 127.95, 122.35 (d, ^2^*J*_CF_ = 23.8 Hz), 122.15 (d, ^3^*J*_CF_ = 8.0 Hz), 106.28, 106.09 (d, ^2^*J*_CF_ = 23.4 Hz), 72.48, 52.55, 51.30, 24.20, 24.18, 24.15; ^31^P NMR(CDCl_3,_ 200 MHz): δ 20.5; IR: ν 3288.6 (NH), 2985.8 (ArH), 1629.9 (CN), 1224.7 (P=O), 987(P-O-C) cm^−1^; *Anal*. Calcd for C_21_H_25_FN_3_O_3_P: C 60.43%, H 6.04%, N 10.07%; Found. C 60.21%, H 5.94%, N 9.85%.

*Diethyl (2-fluorophenyl)(6-fluoroquinazolin-4-ylamino)methylphosphonate* (**3p**): White solid, yield 85.3%; mp 142 °C to 144 °C; ^1^H NMR (CDCl_3_, 500 MHz): δ 8.61 (s, 1H, quinazoline-2-H), 8.03 (s, 1H, quinazoline-5-H), 7.79 to 7.82 (m, 1H, Ar-3-H), 7.68~7.70 (m, 2H, quinazoline-7,8-H), 7.46 (brs, 1H, N-H), 7.23 to 7.26 (m, 3H, Ar-4,5,6-H), 6.35 (d, *J* = 10.0 Hz, 1H, C-H), 3.83 to 4.22 (m, 4H, 2CH_2_), 1.16 to 1.23 (m, 6H, 2CH_3_); ^13^C NMR (CDCl_3_, 125 MHz): δ 160.94 (d, ^1^*J*_CF_ = 244.5 Hz), 158.97 (d, ^1^*J*_CF_ = 245.2 Hz), 154.48, 146.84, 135.32 (d, ^3^*J*_CF_ = 8.0 Hz), 130.61(d, ^2^*J*_CF_ = 24.8 Hz), 130.71(d, ^2^*J*_CF_ = 24.5 Hz), 128.65 (d, ^3^*J*_CF_ = 7.8 Hz), 128.12 (d, ^3^*J*_CF_ = 7.6 Hz), 122.39 (d, ^3^*J*_CF_ = 7.6 Hz), 106.77 (d, ^2^*J*_CF_ = 23.2 Hz), 77.10, 63.47, 63.41, 51.98, 50.74, 16.50, 16.21, 16.17; ^31^P NMR(CDCl_3,_ 200 MHz): δ 21.8; IR: ν 3265.5 (NH), 2985.8-3068.7 (ArH), 1714.7 (CN), 1244.9 (P=O), 1022.2 (P-O-C) cm^−1^; *Anal*. Calcd for C_19_H_20_F_2_N_3_O_3_P: C 56.02%, H 4.95%, N 10.32%; Found. C 56.26%, H 4.91%, N 9.89%.

*Di-n-propyl (2-fluorophenyl)(6-fluoroquinazolin-4-ylamino)methylphosphonate* (**3q**): White solid, yield 85.3%; mp 213 °C to 214 °C; ^1^H NMR (CDCl_3_, 500 MHz): δ 8.72 (s, 1H, quinazoline-2-H), 8.13 (s, 1H, quinazoline-5-H), 7.78 to 7.81 (m, 1H, Ar-3-H), 7.63 to 7.66 (m, 2H, quinazoline-7,8-H), 7.39 (s, 1H, N-H), 7.25 to 7.27 (m, 3H, Ar-4,5,6-H), 6.28 (d, *J* = 10.0 Hz, 1H, C-H), 3.92 to 4.10 (m, 4H, 2OCH_2_), 1.49 to 1.81 (m, 4H, 2CH_2_), 0.78 to 0.81 (m, 6H, 2CH_3_); ^13^C NMR (CDCl_3_, 125 MHz): δ 158.50 (d, ^1^*J*_CF_ = 245.2 Hz), 155.71 (d, ^1^*J*_CF_ = 244.5 Hz), 145.51, 133.29 (d, ^3^*J*_CF_ = 7.8 Hz), 128.75 (d, ^2^*J*_CF_ = 24.3 Hz), 128.55 (d, ^3^*J*_CF_ = 7.6 Hz), 128.51 (d, ^3^*J*_CF_ = 7.8 Hz), 128.34 (d, ^2^*J*_CF_ = 23.8 Hz), 120.23 (d, ^3^*J*_CF_ = 7.7 Hz), 116.85 (d, ^2^*J*_CF_ = 22.5 Hz), 77.11, 69.24, 68.88, 52.26, 51.02, 23.93, 23.69, 9.99, 9.94; ^31^P NMR(CDCl_3,_ 200 MHz): δ 21.7; IR: ν 3271.33 (NH), 2985.85 to 3070.72 (ArH), 1629.86 (CN), 1246.07 (P=O), 1020.34 (P-O-C) cm^−1^; *Anal*. Calcd for C_21_H_24_F_2_N_3_O_3_P: C 57.93%, H 5.56%, N 9.65%; Found. C 58.39%, H 5.27%, N 9.80%.

*Diisopropyl(2-fluorophenyl)(6-fluoroquinazolin-4-ylamino)methylphosphonate* (**3r**): White solid, yield 83.7%; mp 251 °C to 253 °C; ^1^H NMR (CDCl_3_, 500 MHz): δ 8.60 (s, 1H, quinazoline-2-H), 8.03(s, 1H, quinazoline-5-H), 7.80 to 7.82 (m, 1H, Ar-3-H), 7.65 to 7.69 (m, 2H, quinazoline-7,8-H), 7.43 (brs, 1H, N-H), 7.21 to 7.43 (m, 3H, Ar-4,5,6-H), 6.30 (d, *J* = 10.0 Hz, 1H, C-H), 4.60 to 4.86 (m, 2H, 2OCH), 1.15 to 1.32 (m, 12H, 4CH_3_); ^13^C NMR (CDCl_3_, 125 MHz): δ 160.90 (d, ^1^*J*_CF_ = 245.6 Hz), 158.94 (d, ^1^*J*_CF_ = 244.6 Hz), 154.57, 154.56, 146.84, 135.76 (d, ^3^*J*_CF_ = 7.8 Hz), 130.74 (d, ^2^*J*_CF_ = 25.8 Hz), 128.91 (d, ^2^*J*_CF_ = 24.8 Hz), 128.87 (d, ^3^*J*_CF_ = 7.5 Hz), 128.40 (d, ^3^*J*_CF_ = 7.6 Hz), 127.95 (d, ^2^*J*_CF_ = 23.8 Hz), 122.32 (d, ^3^*J*_CF_ = 7.4 Hz), 106.83 (d, ^2^*J*_CF_ = 22.5 Hz), 77.12, 72.54, 72.09, 52.55, 51.30, 24.24, 23.84, 23.23; ^31^P NMR(CDCl_3,_ 200 MHz): δ 21.2; IR: ν 3270.21 (NH), 2981.93 to 3072.64 (ArH), 1629.58 (CN), 1238.43 (P=O), 993.32 (P-O-C) cm^−1^; *Anal*. Calcd for C_21_H_24_F_2_N_3_O_3_P: C 57.93%, H 5.56%, N 9.65%; Found. C 58.06%, H 5.86%, N9.66%.

*Diethyl(6,8-dichloroquinazolin-4-ylamino)(phenyl)methylphosphonate* (**3s**): White solid, yield 86.0%; mp 178 °C to 180 °C; ^1^H NMR (CDCl_3_, 500 MHz): δ 8.73 (s, 1H, quinazoline-2-H), 8.13 (s, 1H, quinazoline-7-H), 7.81 (s, quinazoline-5-H), 7.74 (brs, 1H, N-H), 7.27 to 7.63 (m, 5H, Ar-2,3,4,5,6-H), 6.26 (d, *J* = 10.0 Hz, 1H, C-H), 3.76 to 4.26 (m, 4H, 2CH_2_), 1.13 to 1.26 (m, 6H, 2CH_3_); ^13^C NMR (CDCl_3_, 125 MHz): δ 158.39, 155.33, 155.65, 145.43, 134.87, 133.23, 130.86, 128.68, 128.45, 128.40, 128.29, 63.81, 63.42, 63.36, 52.23, 50.90, 30.96, 16.44, 16.40; ^31^P NMR(CDCl_3_, 200 MHz): δ 22.9; IR: ν 3275.18 (NH), 3072.64 (ArH), 1604.82 (CN), 1228.75 (P=O), 1022.27 (P-O-C) cm^−1^; *Anal*. Calcd for C_19_H_20_Cl_2_N_3_O_3_P: C 51.83%, H 4.58%, N 9.54%; Found. C 52.06%, H 4.71%, N 9.67%.

*Dipropyl(6,8-dichloroquinazolin-4-ylamino)(phenyl)methylphosphonate* (**3t**): White solid, yield 85.7%; mp 150 °C to 152 °C; ^1^H NMR (CDCl_3_, 500 MHz): δ 8.73 (s, 1H, quinazoline-2-H), 8.23 (s, 1H, quinazoline-7-H), 8.13 (brs, 1H, N-H), 7.79 (s, 1H, quinazoline-5-H), 7.25 to 7.68 (m, 5H, Ar-2,3,4,5,6-H), 6.35 (d, *J* = 10.0 Hz, 1H, C-H), 3.97 to 4.14 (m, 4H, 2OCH_2_), 1.52 to 1.61 (m, 4H, 2CH_2_), 0.81 to 0.82 (m, 6H, 2CH_3_); ^13^C NMR (CDCl_3_, 125 MHz): δ 158.55, 155.64, 145.47, 134.98, 133.16, 128.61, 128.57, 128.52, 128.19, 120.41, 116.89, 69.05, 68.91, 52.17, 50.93, 23.86, 23.62, 9.90, 19.88; ^31^P NMR(CDCl_3,_ 200 MHz): δ 21.7; IR: ν 3275.18 (NH), 3072.66 (ArH), 1714.79 (CN), 1224.81 (P=O), 1031.26 (P-O-C) cm^−1^; *Anal*. Calcd for C_21_H_24_Cl_2_N_3_O_3_P: C 53.86%, H 5.17%, N 8.97%; Found. C 53.39%, H 5.27%, N 8.80%.

*Diisopropyl (6,8-dichloroquinazolin-4-ylamino)(phenyl)methylphosphonate* (**3u**): White solid, yield 85.2%; mp 187 °C to 190 °C; ^1^H NMR (CDCl_3_, 500 MHz): δ 8.73 (s, 1H, quinazoline-2-H), 8.05 (s, 1H, quinazoline-7-H), 7.82 (s, 1H, quinazoline-5-H), 7.64 (s, 1H, N-H), 7.27 to 7.43 (m, 5H, Ar-2,3,4,5,6-H), 6.16 (d, *J* = 10.0 Hz, 1H, C-H), 4.53 to 4.78 (m, 2H, 2OCH), 0.88 to 1.29 (m, 12H, 4CH_3_); ^13^C NMR (CDCl_3_, 125 MHz): δ 158.31, 158.24, 155.71, 145.42, 133.25, 128.59, 128.54, 128.21, 119.68, 116.64, 72.86, 60.40, 52.86, 51.61, 24.25, 24.23, 24.12, 23.87, 14.20; ^31^P NMR(CDCl_3,_ 200 MHz): δ 20.2; IR: ν 3275.18 (NH), 3271.3 (NH), 3076.5 (ArH), 1730.2 (CN), 1238.7 (P=O), 1022.2 (P-O-C) cm^−1^; *Anal*. Calcd for C_21_H_24_Cl_2_N_3_O_3_P: C 53.86%, H 5.17%, N 8.97%; Found. C 53.92%, H 5.29%, N 8.33%.

*Diethyl(6,8-dichloroquinazolin-4-ylamino)(2-fluorophenyl)methylphosphonate* (**3v**): White solid, yield 84.5%; mp 128 °C to 130 °C; ^1^H NMR (CDCl_3_, 500 MHz): δ 8.61 (s, 1H, quinazoline-2-H), 7.96 (s, 1H, N-H), 7.79 to 7.84 (m, 2H, quinazoline-5,7-H), 7.23 to 7.27 (m, 4H, Ar-H), 6.35 (d, *J* = 10.0 Hz, 1H, C-H), 3.82 to 4.22 (m, 4H, 2CH_2_), 1.16 to 1.22 (m, 6H, 2CH_3_); ^13^C NMR (CDCl_3_, 125 MHz): δ 160.96 (d, ^1^*J*_CF_ = 244.8 Hz), 158.99, 154.48, 146.83, 135.32, 130.82, 130.75(d, ^2^*J*_CF_ = 24.8 Hz), 128.61 (d, ^3^*J*_CF_ = 7.6 Hz), 128.15 (d, ^3^*J*_CF_ = 7.4 Hz), 122.42 (d, ^2^*J*_CF_ = 22.8 Hz), 106.69, 77.10, 63.59, 63.46, 51.99, 50.75, 16.50, 16.46, 16.22; ^31^P NMR(CDCl_3,_ 200 MHz): δ 22.3; IR: ν 3267.45 (NH), 2947.43~3068.76 (ArH), 1629.82 (CN), 1246.19 (P=O), 1022.37 (P-O-C) cm^−1^; *Anal*. Calcd for C_19_H_19_Cl_2_FN_3_O_3_P: C 49.80%, H 4.18%, N 9.17%; Found. C 50.26%, H 4.61%, N 9.62%.

*Dipropyl (6,8-dichloroquinazolin-4-ylamino)(2-fluorophenyl)methylphosphonate* (**3w**): White solid, yield 83.4%; mp 178 °C to 180 °C; ^1^H NMR (CDCl_3_, 500 MHz): δ 8.72 (s, 1H, quinazoline-2-H), 8.12 (s, 1H, N-H), 7.80 (s, 1H, quinazoline-7-H), 7.63 (s, 1H, quinazoline-5-H), 7.26 to 7.29 (m, 4H, Ar-3,4,5,6-H), 6.27 (d, *J* = 8.5 Hz, 1H, C-H), 3.64 to 4.10 (m, 4H, 2OCH_2_), 1.48~1.80 (m, 4H, 2CH_2_), 0.79 to 0.81 (m, 6H, 2CH_3_); ^13^C NMR (CDCl_3_, 125 MHz): δ 158.46 (d, ^1^*J*_CF_ = 246.2 Hz), 155.71, 145.50, 133.31, 130.95, 128.75 (d, ^2^*J*_CF_ = 23.8 Hz), 128.54 (d, ^3^*J*_CF_ = 7.4 Hz), 128.52, 128.49 (d, ^3^*J*_CF_ = 7.5 Hz), 120.16 (d, ^2^*J*_CF_ = 23.5 Hz), 116.83, 77.10, 69.25, 52.27, 51.03, 23.88, 23.74, 9.99, 9.94; ^31^P NMR(CDCl_3,_ 200 MHz): δ 21.9; IR: ν 3275.1 (NH), 3072.6 (ArH), 1714.7 (CN), 1224.8 (P=O), 997.2 (P-O-C) cm^−1^; *Anal*. Calcd for C_21_H_23_Cl_2_FN_3_O_3_P: C 51.87%, H 4.77%, N 8.64%; Found. C 51.39%, H 5.21%, N 8.80%.

*Diisopropyl (6,8-dichloroquinazolin-4-ylamino)(2-fluorophenyl)methylphosphonate* (**3x**): White solid, yield 82.6%; mp 202 °C to 204 °C; ^1^H NMR (CDCl_3_, 500 MHz): δ 8.60 (s, 1H, quinazoline-2-H), 8.07 (s, 1H, N-H), 7.81 (s, 1H, N-H), 7.60 (s, 1H, quinazoline-7-H), 7.51 (s, 1H, quinazoline-5-H), 7.21 to 7.25 (m, 4H, Ar-3,4,5,6-H), 6.27 (d, *J* = 25.0Hz, 1H, CH), 4.60 to 4.86 (m, 2H, 2CH), 1.14 to 1.31 (m, 12H, 4CH_3_ ); ^13^C NMR (CDCl_3_, 125 MHz): δ 160.91, 159.06 (d, ^1^*J*_CF_ = 246.2 Hz), 158.94, 154.56, 146.84, 135.75, 128.90 (d, ^2^*J*_CF_ = 24.5 Hz), 128.45 (d, ^3^*J*_CF_ = 7.4 Hz), 127.95(d, ^3^*J*_CF_ = 7.6 Hz), 122.33, 122.14 (d, ^2^*J*_CF_ = 23.6Hz), 116.02, 77.12, 72.50, 52.55, 24.24, 23.84, 23.27; ^31^P NMR(CDCl_3,_ 200 MHz): δ 20.4; IR: ν 3269.36 (NH), 2981.91 (ArH), 1712.8 (CN), 1238.38 (P=O), 1014.66 (P-O-C) cm^−1^; *Anal*. Calcd for C_21_H_23_C_l2_FN_3_O_3_P: C 51.87%, H 4.77%, N 8.64%; Found. C 51.46%, H 4.86%, N 8.66%.

### 3.3. Antiviral Activity Bioassay

The purification of the tobacco mosaic virus. Using Gooding’s method [32], the upper leaves of *Nicotiana tabacum* L. inoculated with TMV were selected and ground in a phosphate buffer, then filtered through double layer pledget. The filtrate was centrifuged at 10,000 g, treated twice with PEG and centrifuged again. The whole experiment was carried out 4 °C. Absorbance values were estimated at 260 nm using an ultraviolet spectrophotometer:

$$\text{virus concn}=({\text{A}}_{260}\times \text{dilution ratio})/E_{1\;\text{cm}}^{0.1\%,260\;\text{nm}}$$

Curative effect of compounds against TMV *in vivo*. The growing leaves of *Nicotiana tabacum.* L. of the same ages were selected. The tobacco mosaic virus (concentration of 6 × 10^−3^ mg/mL) was dipped and inoculated on the whole leaves. The leaves were then washed with water and dried. The compound solution was smeared on the left side and the solvent was kept on the right side for control. The local lesion numbers were then recorded 3–4 days after inoculation [30]. For each compound, three repetitions were conducted to ensure the reliability of the results, which were measured according to the following formula:

$$\text{Inhibition rate }(\%)=\frac{\text{av local}\;\text{lesion numbers of control }(\text{not treated}\;\text{with compound})-\text{av local lesion numbers}\;\text{smeared with drugs}}{\text{av local lesion numbers without drugs}}$$

## 4. Conclusions

In summary, an efficient and simple method for the synthesis of quinazolin-4-ylamino)methylphosphonate derivatives was developed through the reaction of *N*′-(substituted-2-cyanophenyl)-*N*,*N*-dimethylformamidine derivatives with dialkyl amino (phenyl)methylphosphonate under microwave irradiation. This novel method has several advantages over the other methods, such as high yield, fewer reaction steps, faster reaction rate, and easy work up without producing any significant by-product. By contrast, the classical method (Scheme 1) of quinazoline derivatives bearing α-aminophosphonate moiety formation involves long reaction times (6 h). All compounds, 3a to 3×, were fully characterized using the spectroscopic methods. Preliminary bioassays indicated that some of these compounds are also associated with good inhibitory activities against TMV at 500 mg/L. Further studies are currently underway to establish a definite structure activity relationship.

## Supporting Information



## Figures and Tables

**Scheme I:**
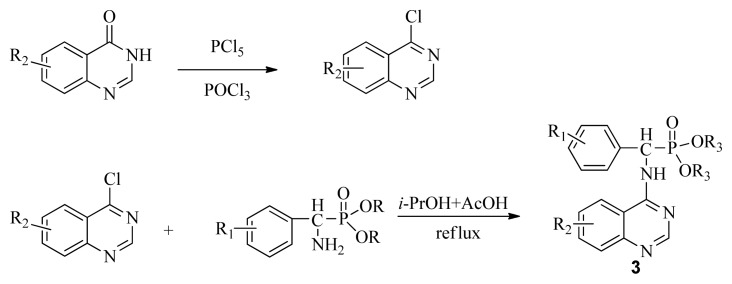
The traditional method of producing the title compound **3**.

**Scheme II:**
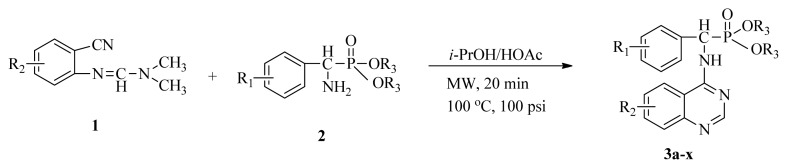
Synthesis of the title compounds **3a** to **3x**.

**Scheme III:**
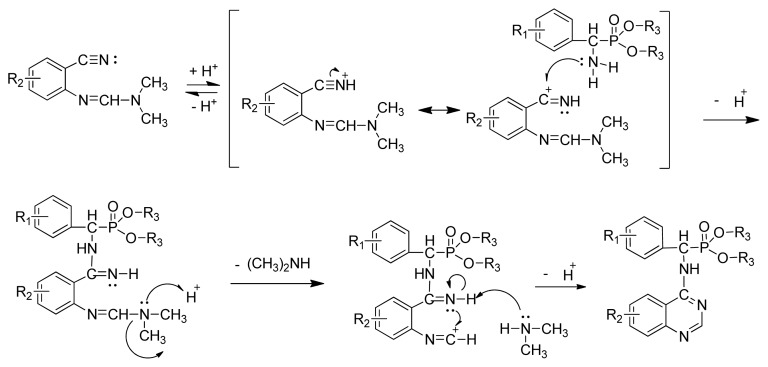
Possible mechanism for the formation of the title compounds.

**Table 1:** Synthesis of **3a** under different reaction conditions.

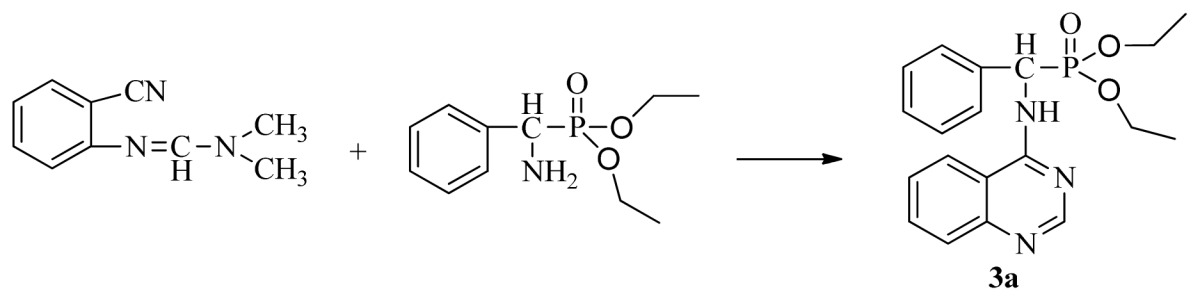						

Entry	Solvent	Time (min)	Reaction Temperature (°C)	Power(W)	Pressure (psi)	Yield (%)
1	isopropanol/acetic acid (v/v,4:1)	20	100	100	100	59.0
2	acetonitrile/acetic acid (v/v, 4:1)	20	100	100	100	40.1
3	chloroform/acetic acid (v/v, 4:1)	20	100	100	100	43.9
4	isopropanol/acetic acid (v/v, 4:2)	20	100	100	100	51.3
5	isopropanol/acetic acid (v/v, 4:1)	10	100	100	100	45.1
6	isopropanol/acetic acid (v/v, 4:1)	30	100	100	100	62.5
7	isopropanol/acetic acid (v/v, 4:1)	20	130	100	130	69.6
8	isopropanol/acetic acid (v/v, 4:1)	20	150	100	150	79.0
9	isopropanol/acetic acid (v/v, 4:1)	20	170	100	170	77.0
10	isopropanol/acetic acid (v/v, 4:1)	20	150	60	100	43.0
11	isopropanol/acetic acid (v/v, 4:1)	20	150	80	100	70.0
12	isopropanol/acetic acid (v/v, 4:1)	360	reflux	0	0	33.5

**Table 2:** Synthesis of (quinazolin-4-ylamino)methylphosphonate derivatives via microwave irradiation.

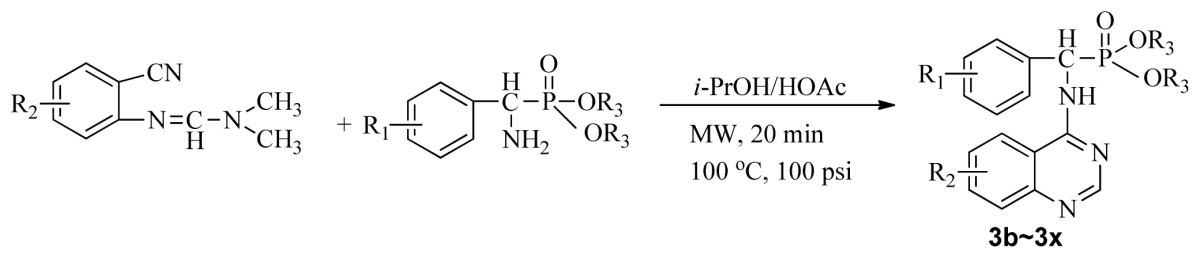	

Entry	Product
1	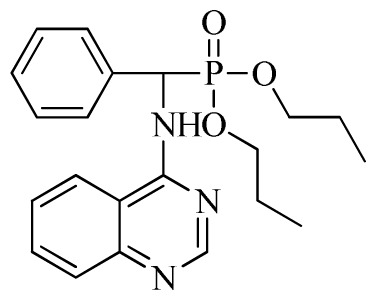 **3b**(78.0%)
2	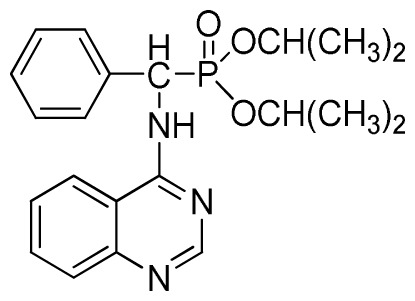 **3c** (78.1%)
3	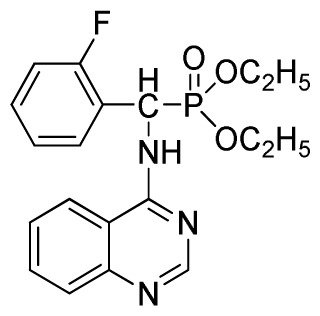 **3d** (77.8%)
4	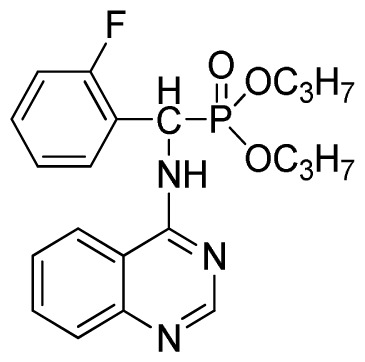 **3e** (77.5%)
5	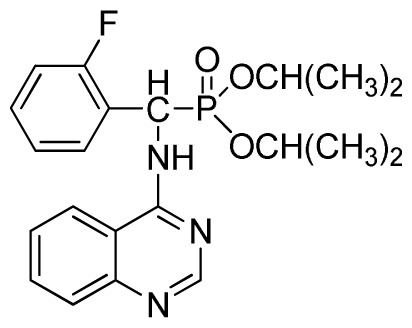 **3f** (77.5%)
6	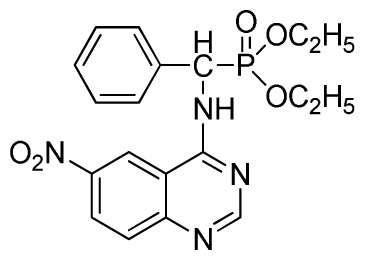 **3g** (85.9%)
7	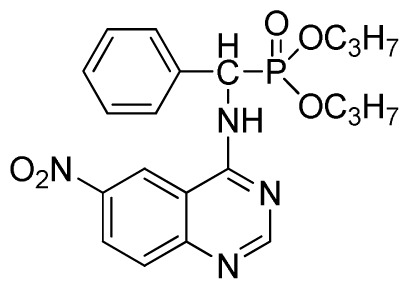 **3h** (85.7%)
8	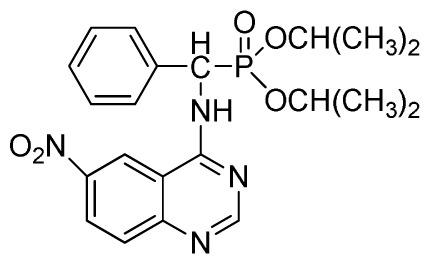 **3i** (85.6%)
9	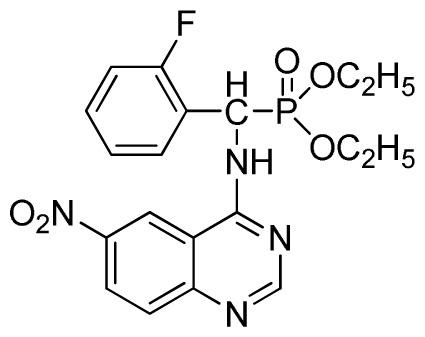 **3j** (85.2%)
10	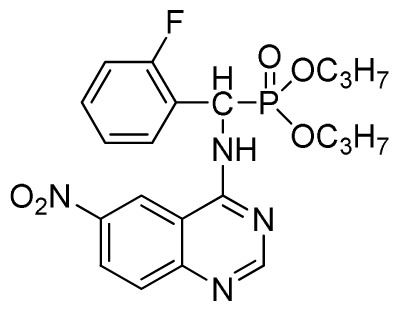 **3k** (85.0%)
11	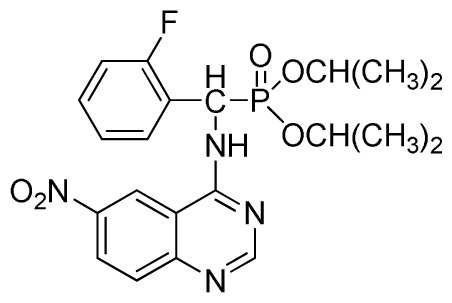 **3l** (83.9%)
12	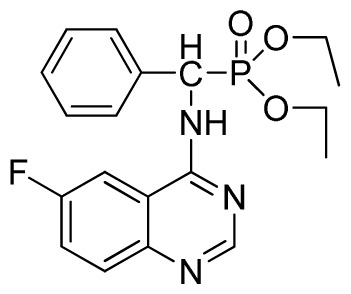 **3m** (86.1%)
13	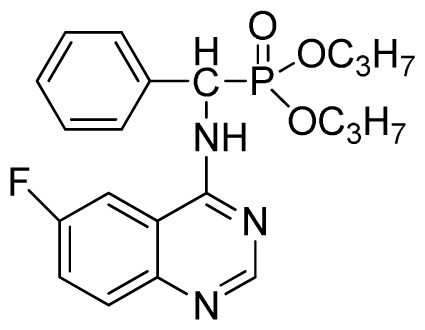 **3n** (86.1%)
14	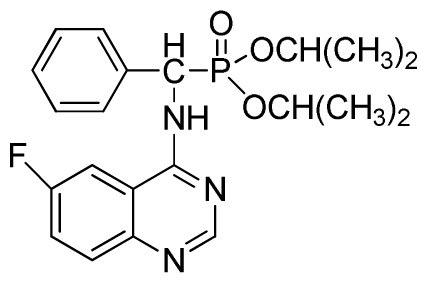 **3o** (85.3%)
15	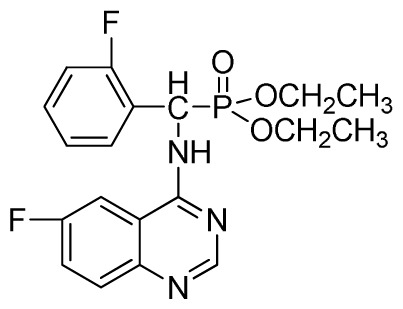 **3p** (85.3%)
16	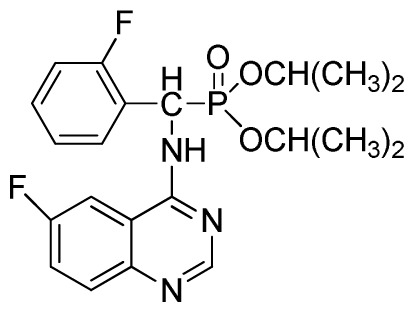 **3q** (84.8%)
17	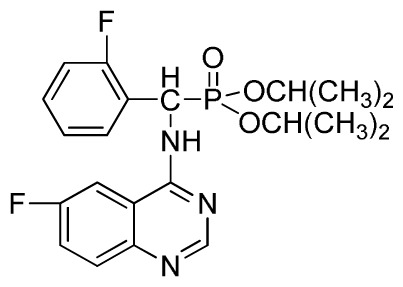 **3r** (83.7%)
18	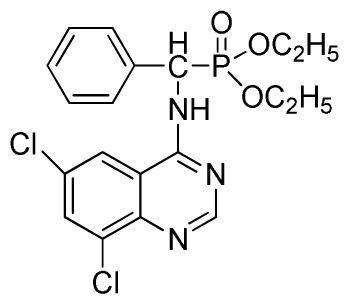 **3s** (86.0%)
19	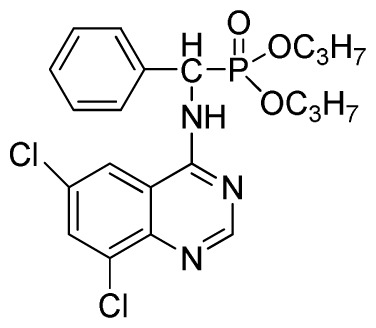 **3t** (85.7%)
20	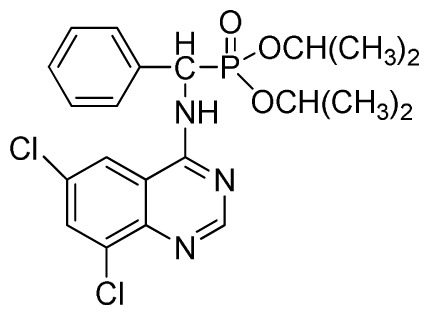 **3u** (85.2%)
21	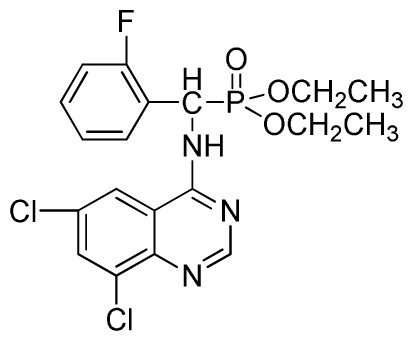 **3v** (84.5%)
22	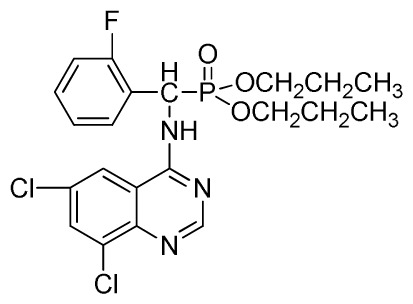 **3w** (83.4%)
23	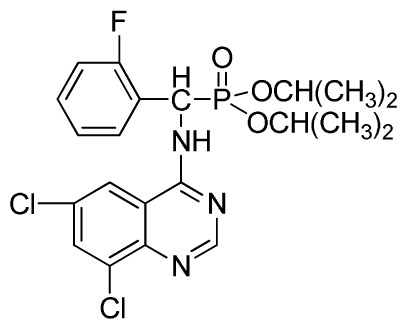 **3x** (82.6%)

**Table 3:** The curative effect of the compounds **3a**–**x** against TMV *in vivo*.

Agent	Concentration (μg/mL)	Curative Effect (%)
**3a**	500	43.2
**3b**	500	38.4
**3c**	500	32.0
**3d**	500	37.8
**3e**	500	31.9
**3f**	500	30.1
**3g**	500	35.9
**3h**	500	33.0
**3i**	500	33.9
**3j**	500	36.0
**3k**	500	31.9
**3l**	500	38.0
**3m**	500	44.0
**3n**	500	45.6
**3o**	500	41.2
**3p**	500	52.0
**3q**	500	49.1
**3r**	500	40.1
**3v**	500	51.9
**3w**	500	44.8
**3x**	500	47.9
Ningnanmycin	500	55.9
