# Supplementary material for: Rapid Synthesis and Antiviral Activity of (Quinazolin-4-Ylamino)Methyl-Phosphonates Through Microwave Irradiation
Source: Int J Mol Sci. 2012 Jun 1;13(6):6730–46. doi: 10.3390/ijms13066730 (PMC3397492; doi:10.3390/ijms13066730)
Supplement: Supplementary file 1 [file ijms-13-06730-s001.pdf]

Rapid Synthesis and Antiviral Activity of (Quinazolin-4-Ylamino)Methyl-Phosphonates Through Microwave Irradiation

Supporting Information

1. Instruments

The melting points of the products were determined on a XT-4 binocular microscope (Beijing Tech Instrument Co., China) and are uncorrected. IR spectra (KBr disks) were recorded on a Bruker Vector 22 spectrometer. ^{1}H, ^{13}C, and ^{31}P NMR spectra were recorded at room temperature on a JEOL-ECX 500 NMR spectrometer operating at 500 and 125 MHz, respectively, using TMS as an internal standard. Microwave irradiations were carried out in a Discovery^{TM} LabMate instrument. Elemental analysis was performed on an Elementar Vario-III CHN analyzer. Analytical TLC was performed on silica gel GF254. Column chromatographic purification was carried out using silica gel. All reagents were of analytical reagent grade or chemically pure. All solvents were dried, deoxygenated, and redistilled before use.

2. Synthesis of Intermediate 2a-f

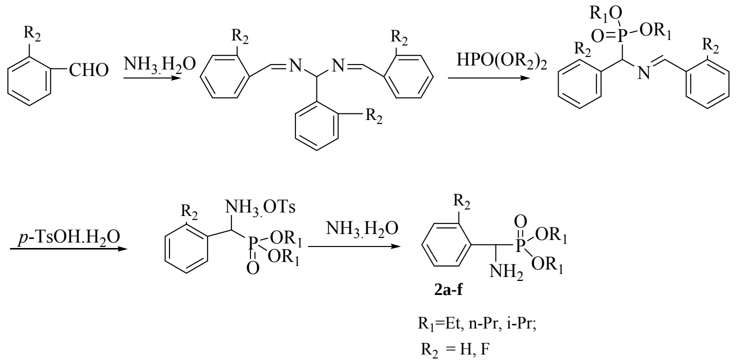

Diethyl amino(phenyl)methylphosphonate (2a)

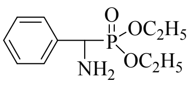

The benzaldehyde (21.05 g, 0.2 mol) was slowly added to ammonium hydroxide (30%, 15 mL) and the mixture was stirred for 2 h under reflux. Then, a white precipitate was formed which was separated by filtration and dried. Intermediate 4 (6.02 g, 0.02 mol) was added to diethyl phosphonate (0.03 mol) and the mixture was stirred for 8 h at 75–85 °C. Chromatography on silica gel with EtOAc/Petroleum ether (1:1) gave the pure intermediate 5 as oils. *p*-Toluenesulfonic acid (3.46 g, 0.02 mol) in 60 mL THF was added to the intermediate 5 (6.65 g, 0.02 mol) and the reaction mixture was stirred for 2 h at 0 °C. The precipitate formed was removed by filtration and washed by THF (30 mL) several times to obtain white solid intermediate 6（5.24 g，0.013 mol. Ammonium hydroxide (30%, 80 mL) was slowly added to intermediate 6 and the mixture was stirred for 2 h at room temperature; the mixture was extracted with ethyl ether (2 × 20 mL), followed by evaporation to obtain crude products (2.02 g).

During this time, this products was chrom-atographed over a silica gel column using a solvent system (EtOAc/Petroleum ether = 1:10 to 1:0) to give the pure target intermediate 2a (1.79 g, 58.2%) as Colorless viscous liquid; nD^{20} = 1.4950; ^{1}H NMR ($CDCl_3$, 500 MHz) δ: 7.32–7.45 (m, 5H, Ar-2,3,4,5,6-H), 4.23 (d, *J* = 10.0 Hz, 1H, CH), 4.02~4.09 (m, 4H, $2CH_2$), 2.01 (s, 2H, NH_2), 1.16–1.25 (m, 6H, $2CH_3$), ^{13}C NMR ($CDCl_3$, 125MHz): *δ* 137.60, 128.35, 127.76, 127.73, 127.68, 77.40, 62.79, 62.67, 16.39, 16.35, ^{31}P NMR ($CDCl_3$, 500 MHz): δ 25.3; IR (KBr): ν 3379.08, 2981.34, 1238. 56, 1026.39; *Anal.* Calcd for $C_{11}H_{18}NO_3P$: C 54.32, H 7.46, N 5.76; Found. C 54.56, H 7.25, N 5.84.

Di-*n*-propyl amino(phenyl)methylphosphonate (2b)

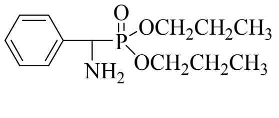

Intermediate **2b** was prepared by reacting intermediate **4** (6.02 g, 0.02 mol) with dipropyl phosphonate (0.03 mol) using a procedure similar to the preparation of **2a** to obtain colorless liquid of **2b** (1.83 g, 56.1%); nD^{20} = 1.4865; ^{1}H NMR: ($CDCl_3$, 500 MHz) δ: 7.35–7.46 (m, 5H, Ar-2,3,4,5,6-H), 4.24 (d, *J* = 10.0 Hz, 1H, CH), 3.93–4.12 (m, 4H, $2OCH_2$), 2.05 (s, 2H, NH_2), 0.94–1.26 (m , 4H, $2CH_2$), 0.87–0.93 (m, 6H, $2CH_3$), ^{13}C NMR ($CDCl_3$, 125 MHz): δ 137.86, 128.32, 77.72, 77.18, 68.01, 60.29, 54. 61, 53.43, 23.86, 20.94, 14.14, 9.97, 9.90, ^{31}P NMR ($CDCl_3$, 500 MHz): δ 24.7; IR (KBr): ν 3377.02, 2981.56, 1232.20, 1026.06); *Anal.* Calcd for $C_{13}H_{22}NO_3P$: C 57.55, H 8.17, N 5.16; Found. C 57.26, H 8.23, N 10.95.

Diispropyl amino(phenyl)methylphosphonate (2c)

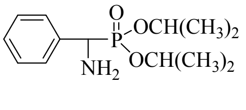

Intermediate **2c** was prepared by contacting intermediate **4** (6.02 g, 0.02 mol) with disopropyl phosphonate (0.03 mol) using a procedure similar to the preparation of **2a** to obtain colorless liquid of **2c** (1.87 g, 56.7%); nD^{20} = 1.4867. ^{1}H NMR: ($CDCl_3$, 500 MHz) δ: 7.33–7.45 (m, 5H, Ar-2,3,4,5,6-H), 4.76 (d, *J* = 8.5Hz, 1H, CH), 4.59–4.66 (m, 1H, OCH), 4.48–4.54 (m, 1H, OCH), 2.04 (s , 2H, NH_2), 1.24–1.30 (m, 12H, $4CH_3$), ^{13}C NMR ($CDCl_3$,125MHz): δ 128.33, 127.94, 127.90, 127.76, 127.75, 77.30, 77.06, 76.80, 71.46, 55.05, 24.20, 24.19, 23.90, ^{31}P NMR ($CDCl_3$, 500 MHz): δ 23.9; IR (KBr, cm^{-1}): ν 3379.02, 2981.56, 1233.21, 1025.86; *Anal.* Calcd for $C_{13}H_{22}NO_3P$: C 57.55, H 8.17, N 5.16; Found. C 57.41, H 8.16, N 10.87.

Diethyl amino(2-fluorophenyl)methylphosphonate (2d)

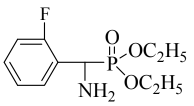

The 2-fluorobenzaldehyde (24.62 g, 0.2 mol) was slowly added to ammonium hydroxide (30%, 15 mL) and the mixture was stirred for 2 h under reflux.Then, a white precipitate was formed which

was separated by filtration and dried. Intermediate 7 (7.06 g, 0.02 mol) was added to diethyl phosphonate (0.03 mol) and the mixture was stirred for 8 h at 75–85 °C. Chromatography on silica gel with EtOAc/Petroleum ether (1:1) gave the pure intermediate **8** as oils. 4-Methylbenzenesulfonic acid(3.46 g, 0.02 mol) in 60 mL THF was added to the intermediate **8** (6.65 g, 0.02 mol) and the reaction mixture was stirred for 2 h at 0 °C. The precipitate formed was removed by filtration and washed by THF (30 mL) several times to obtain white solid intermediate of **9** **(**4.35 g, 0.01 mol). Ammonium hydroxide (30%, 80 mL) was slowly added to intermediate **9** and the Mixture was stirred for 2h at room temperature; the mixture was extracted with ethyl ether (2 × 20 mL), followed by evaporation to obtain crude products (1.68 g). During this time, this products was chromatographed over a silica gel column using a solvent system (EtOAc/Petroleum ether = 1:10 to 1:0) to give Colorless viscous liquid of the pure target intermediate **2d** (1.40 g, 53.2%) as Colorless viscous liquid; n^{D20} = 1.4766; ^{1}H NMR: ($CDCl_3$, 500 MHz): δ 7.05–7.57 (m, 4H, Ar-3,4,5,6-H), 4.59 (d, *J* = 15.0 Hz, 1H, CH), 4.12–4.15 (m, 4H, $2CH_2$), 2.36 (s, 2H, NH_2), 1.15–1.32 (m, 6H, $2CH_3$), ^{13}C NMR ($CDCl_3$, 125 MHz) δ: 137.72, 128.18, 127.64, 127.60, 77.59, 62.61, 62.56, 16.28, 16.23, 16.15, ^{31}P NMR ($CDCl_3$, 500 MHz): δ 24.7; IR (KBr): ν 3377.36, 2981.95, 1234.44, 1028.06; *Anal.* Calcd for $C_{11}H_{17}FNO_3P$: C 50.58, H 6.56, N 5.36; Found. C 50.41, H 6.27, N 5.74

Di-*n*-propyl amino(2-fluorophenyl)methylphosphonate (2e)

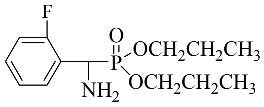

Intermediate **2e** was prepared by reacting intermediate **7** (7.06 g, 0.02 mol) with dipropyl phosphonate (5.10 g, 0.03 mol) using a procedure similar to the preparation of **2d** to obatin colorless liquid of **2e** (1.49 g, 52.7%); nD^{20} = 1.4826; ^{1}H NMR: ($CDCl_3$, 500 MHz): δ 7.04–7.57 (m, 4H, Ar-3,4,5,6-H), 4.62 (d, 1H, *J* = 15Hz, CH), 4.03–4.13 (m, 4H, $2OCH_2$), 2.05 (s, 2H, NH_2), 0.96–1.26 (m , 4H, $2CH_2$), 0.82–0.94 (m, 6H, $2CH_3$), ^{13}C NMR ($CDCl_3$, 125MHz): δ 129.42, 124.45, 77.38, 77.12, 68.44, 60.50, 47.05, 45.86, 22.98, 22.89, 21.15, 14.29, 9.94, ^{31}P NMR ($CDCl_3$, 500 MHz) δ: 24.7; IR (KBr, cm^{-1}): ν 3296.35, 2931.80, 1235.56, 1053.13; *Anal.* Calcd for $C_{13}H_{21}FNO_3P$: C 53.97, H 7.32, N 4.84; Found. C 54.21, H 7.16, N 4.87.

Diispropyl amino(2-fluorophenyl)methylphosphonate(2f)

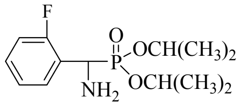

Intermediate **2f** was prepared by reacting intermediate **7** (7.06 g, 0.02 mol) with disopropyl phosphonate (5.10 g, 0.03 mol) using a procedure similar to the preparation of **2d** to obtain colorless liquid of **2f** (1.70 g, 52.1%); nD^{20} = 1.4826; ^{1}H NMR: ($CDCl_3$, 500 MHz): *δ* 7.05~7.59 (m, 4H, Ar-3,4,5,6-H), 4.72–4.74 (m, 2H,2OCH), 4.52 (d, *J* = 15Hz, 1H, CH), 2.17 (s, 2H, NH_2), 1.24–1.34 (m, 12H, $4CH_3$); ^{13}C NMR ($CDCl_3$,125MHz) δ: 128.97, 125.68, 125.57, 123.99, 115.01, 114.70, 77.50, 71.23, 45.87, 45.77, 23.70, 23.83, 23.57; ^{31}P NMR: ($CDCl_3$, 500 MHz) δ: 23.0; IR (KBr, cm^{-1}): ν 3346.16, 2951.73, 1223.90, 1015.46; *Anal.* Calcd for $C_{13}H_{21}FNO_3P$: C 53.97, H 7.32, N 4.84; Found.

C 54.16, H 7.53, N 4.99.

3. NMR Spectra (^{1}H, ^{13}C and ^{31}P) for 1a-d, 2a-f, 3a-3x

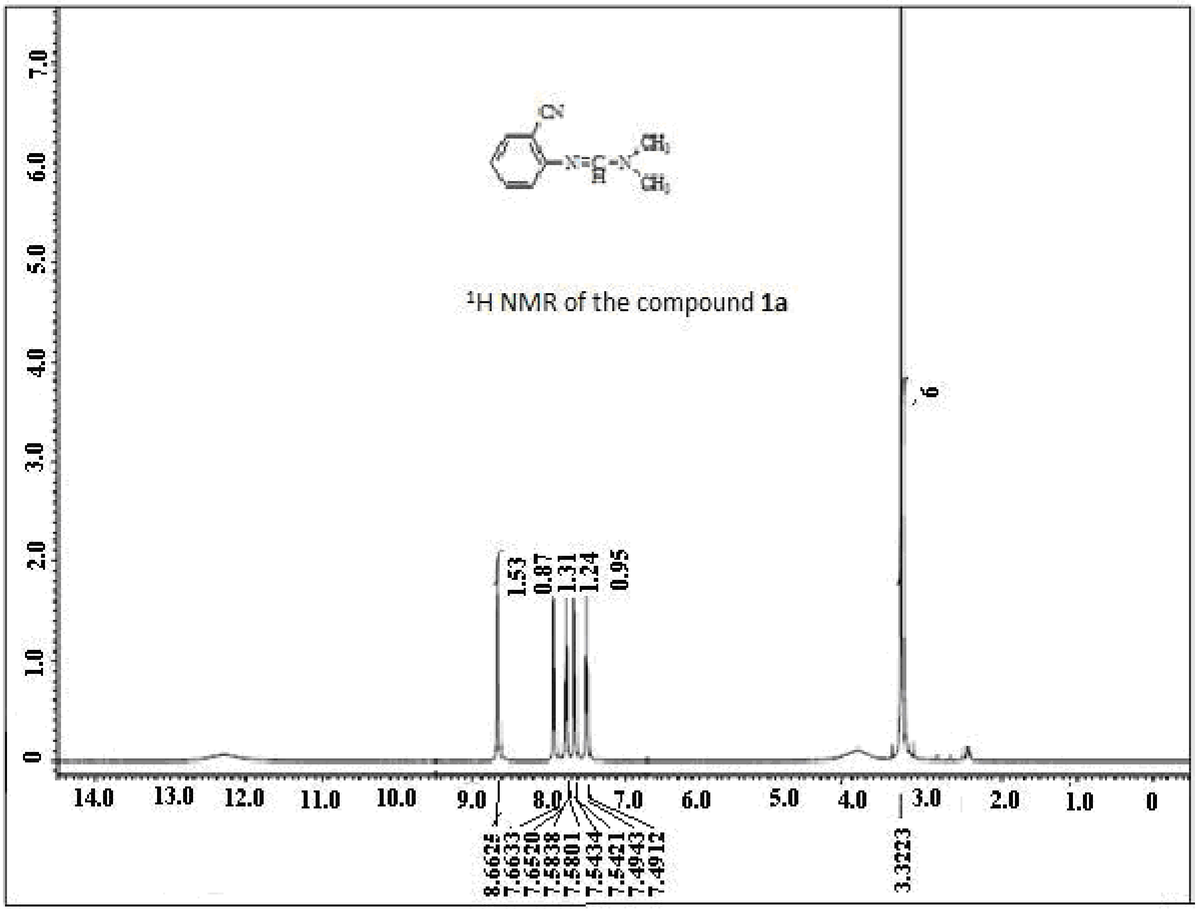

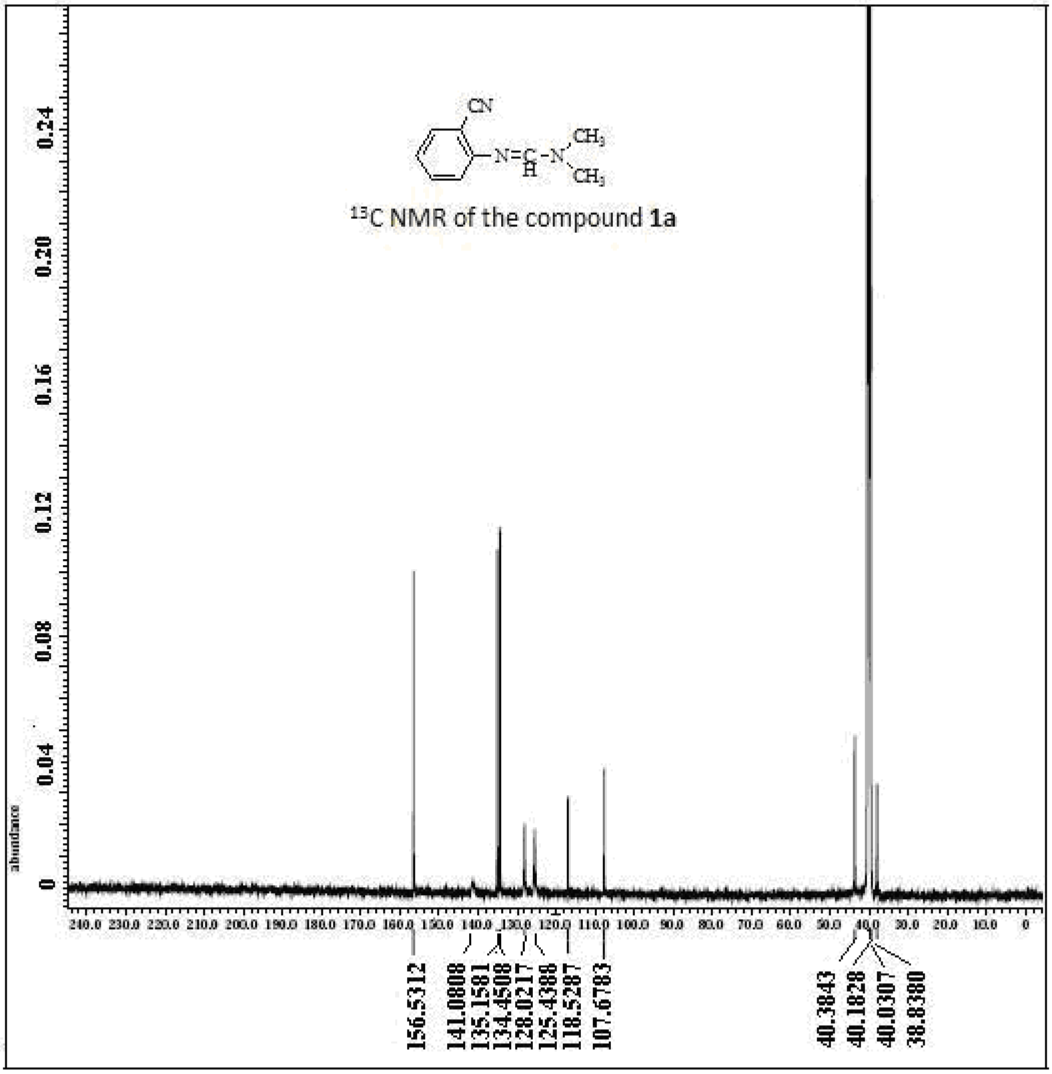

CN
N=C-N
H
CH3
CH3
13C NMR of the compound 1a
abundance
0
0.04
0.08
0.12
0.16
0.20
0.24
240.0 230.0 220.0 210.0 200.0 190.0 180.0 170.0 160.0 150.0 140.0 130.0 120.0 110.0 100.0 90.0 80.0 70.0 60.0 50.0 40.0 30.0 20.0 10.0 0
156.5312
141.0808
135.1581
134.4508
128.0217
125.4388
118.5287
107.6783
40.3843
40.1828
40.0307
38.8380

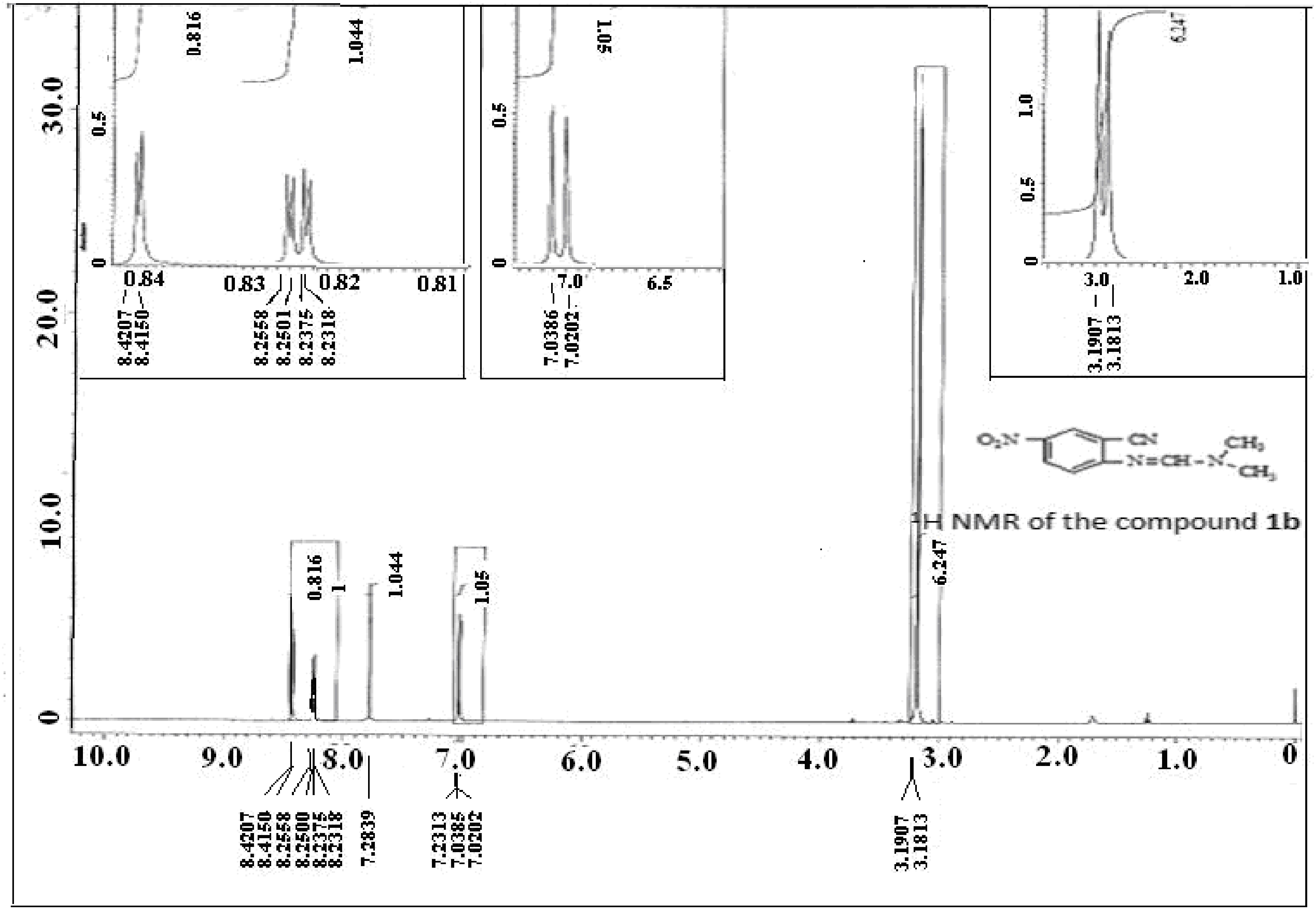

1H NMR of the compound 1b
10.0
9.0
8.0
7.0
6.0
5.0
4.0
3.0
2.0
1.0
0
0.816
1
1.044
1.05
6.247
8.4207
8.4150
8.2558
8.2500
8.2375
8.2318
7.2839
7.2313
7.0385
7.0202
3.1907
3.1813

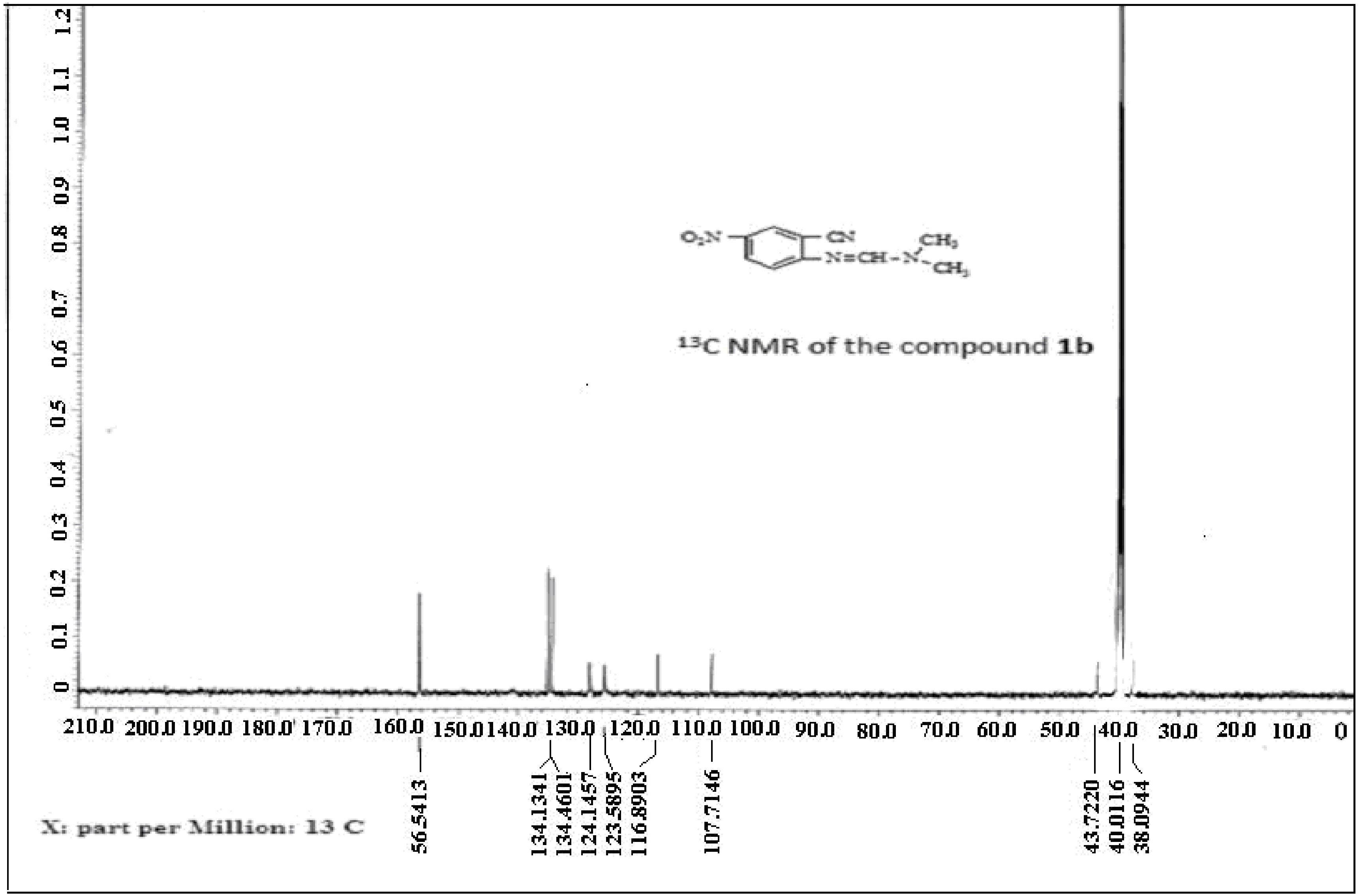

O_2N
CN
N=CH-N
CH_3
CH_3
^{13}C NMR of the compound **1b**
X: part per Million: 13 C
210.0 200.0 190.0 180.0 170.0 160.0 150.0 140.0 130.0 120.0 110.0 100.0 90.0 80.0 70.0 60.0 50.0 40.0 30.0 20.0 10.0 0
0 0.1 0.2 0.3 0.4 0.5 0.6 0.7 0.8 0.9 1.0 1.1 1.2
56.5413
134.1341
134.4601
124.1457
123.5895
116.8903
107.7146
43.7220
40.0116
38.0944

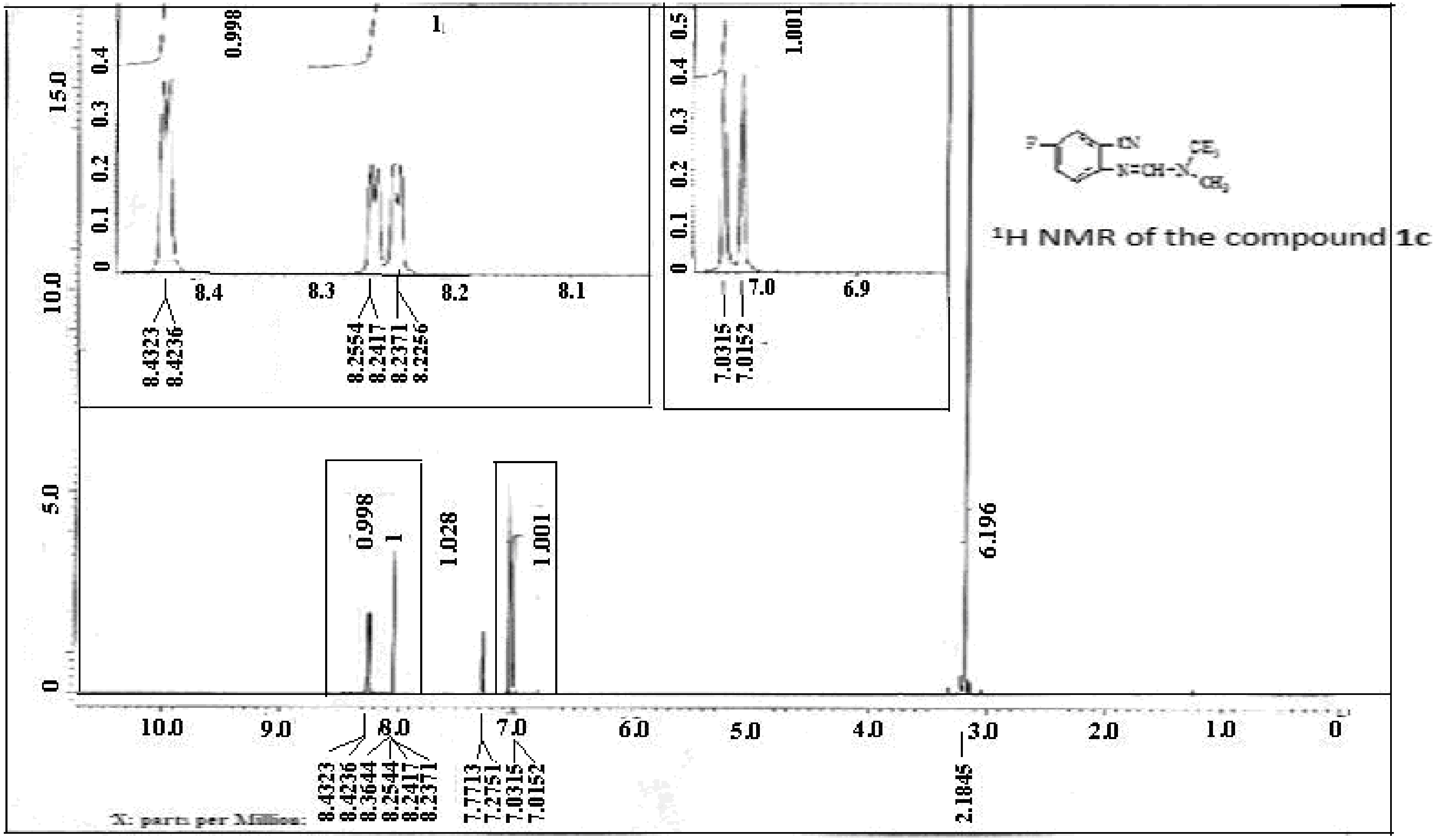
1H NMR of the compound 1c
X: parts per Million
0.998
1
1.028
1.001
6.196
8.4323
8.4236
8.3644
8.2544
8.2417
8.2371
7.7713
7.2751
7.0315
7.0152
2.1845
8.2554
8.2256

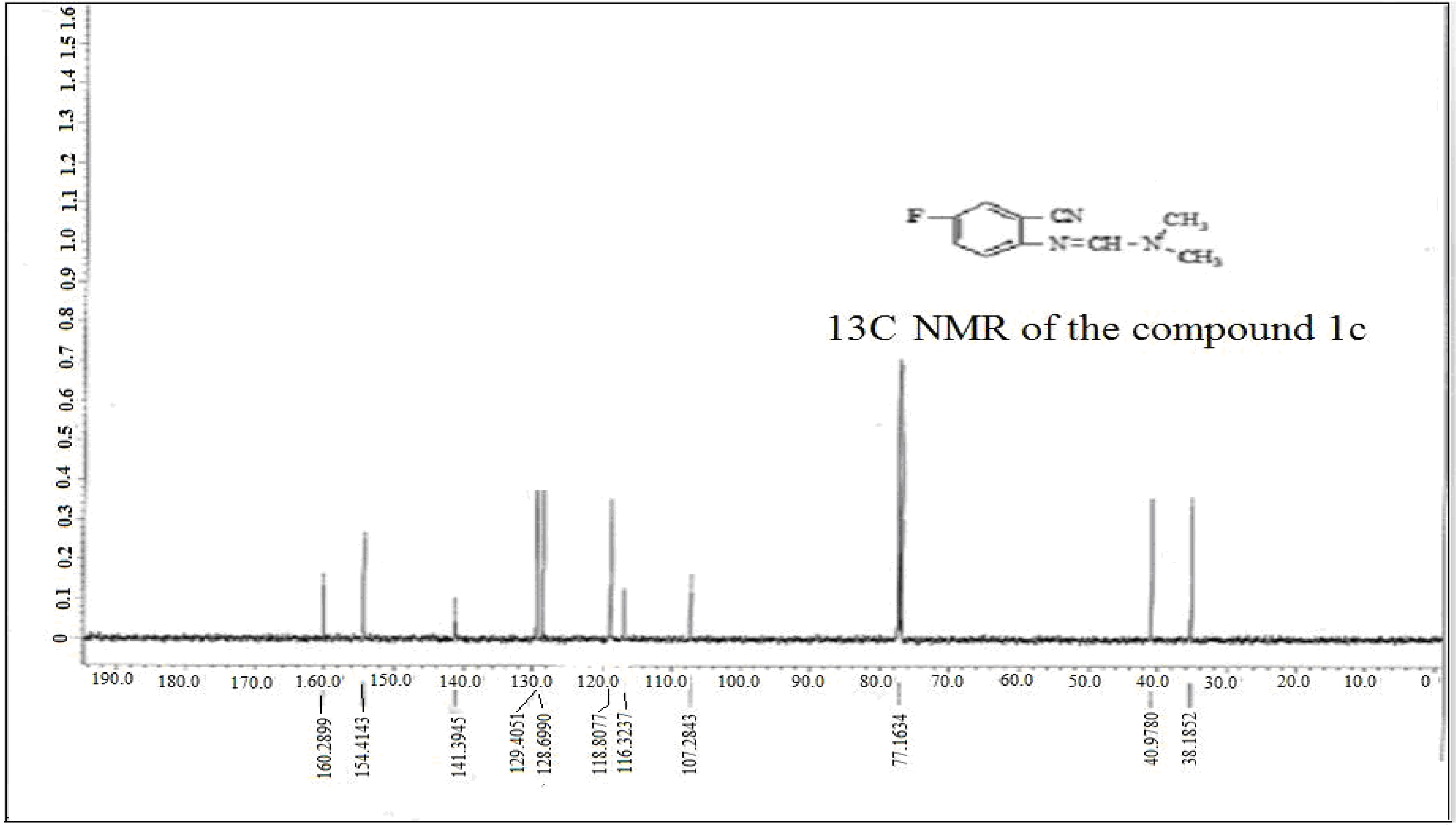
F
CN
N=CH-N
CH3
CH3
13C NMR of the compound 1c
160.2899
154.4143
141.3945
129.4051
128.6990
118.8077
116.3237
107.2843
77.1634
40.9780
38.1852

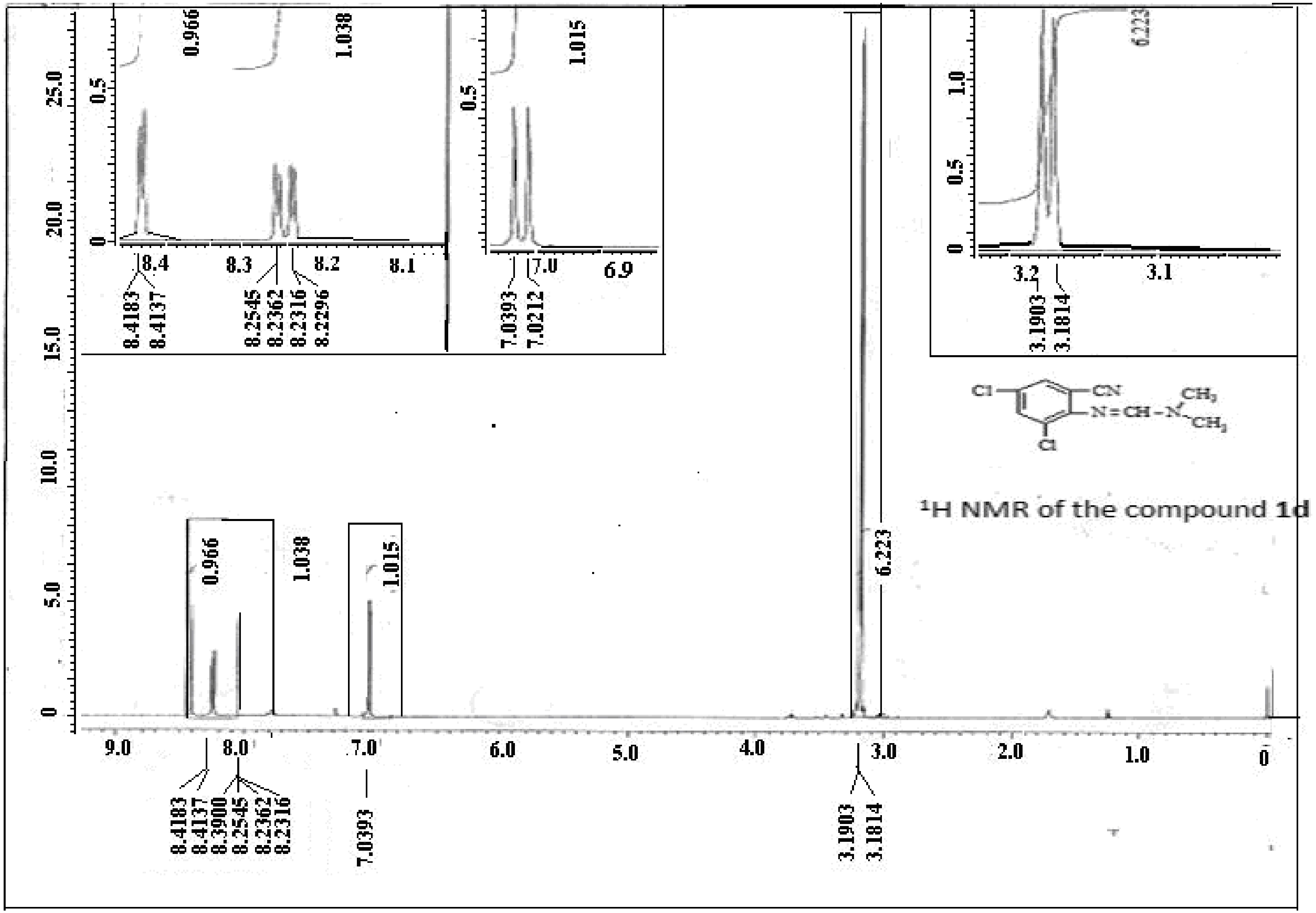

¹H NMR of the compound 1d
Cl
CN
N=CH–N
CH3
CH3
Cl
0.966
1.038
1.015
6.223
8.4183
8.4137
8.3900
8.2545
8.2362
8.2316
8.2296
7.0393
7.0212
3.1903
3.1814
9.0
8.0
7.0
6.0
5.0
4.0
3.0
2.0
1.0
0

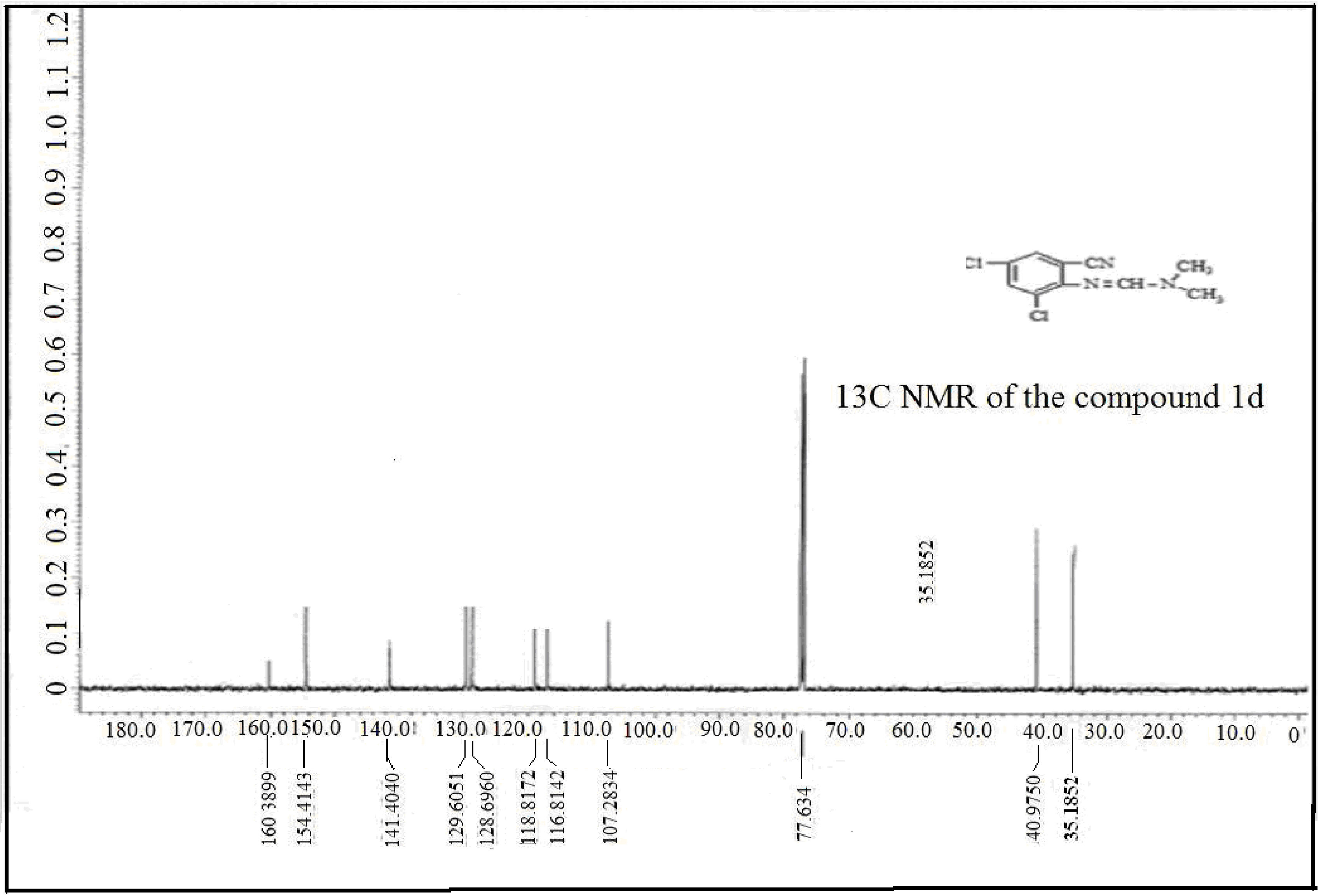
13C NMR of the compound 1d
Cl
CN
N=CH-N
CH3
CH3
Cl
160.3899
154.4143
141.4040
129.6051
128.6960
118.8172
116.8142
107.2834
77.634
35.1852
40.9750
35.1852
180.0
170.0
160.0
150.0
140.0
130.0
120.0
110.0
100.0
90.0
80.0
70.0
60.0
50.0
40.0
30.0
20.0
10.0
0
1.2
1.1
1.0
0.9
0.8
0.7
0.6
0.5
0.4
0.3
0.2
0.1
0

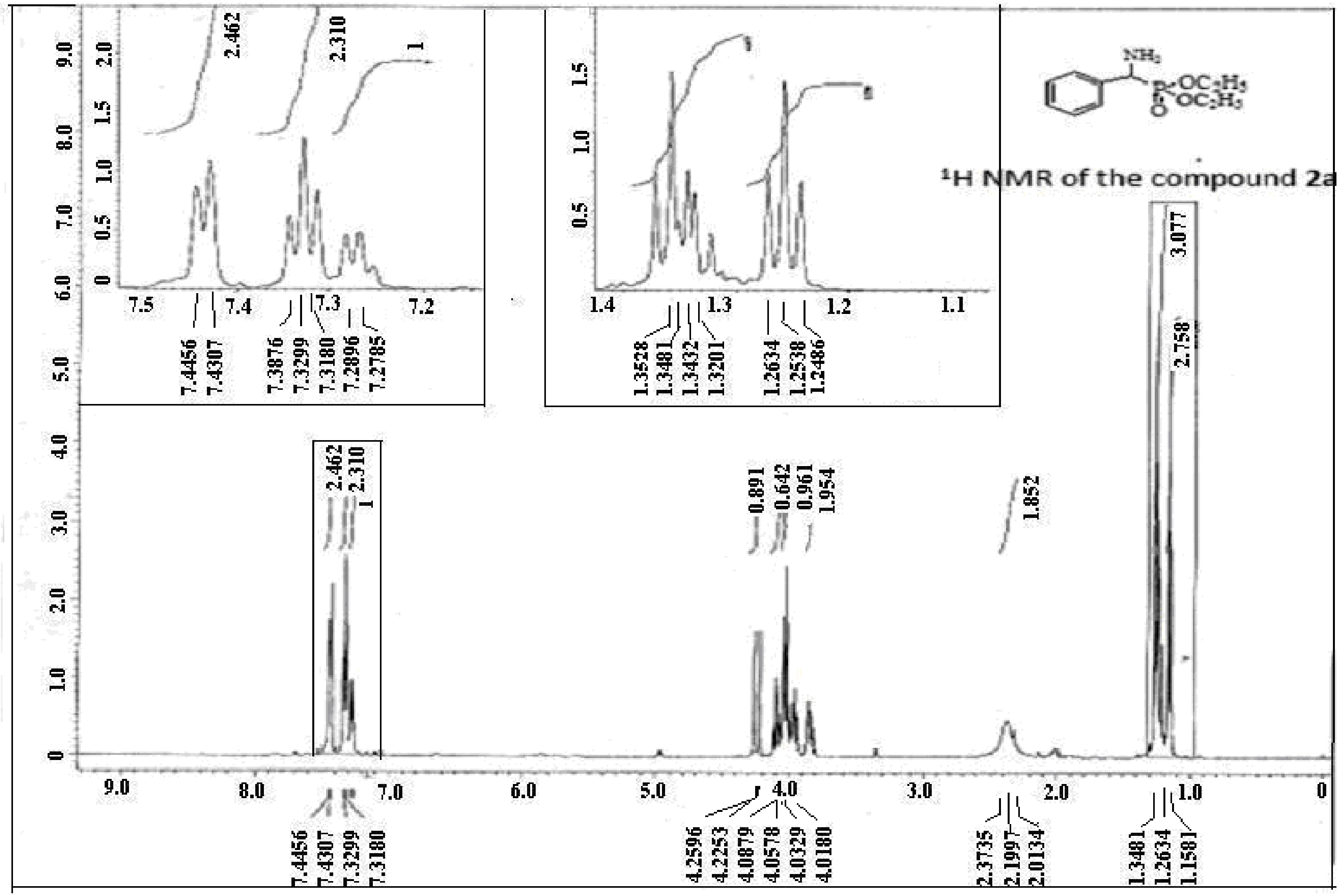
¹H NMR of the compound 2a
NH₂
P-OC₂H₅
OC₂H₅
O

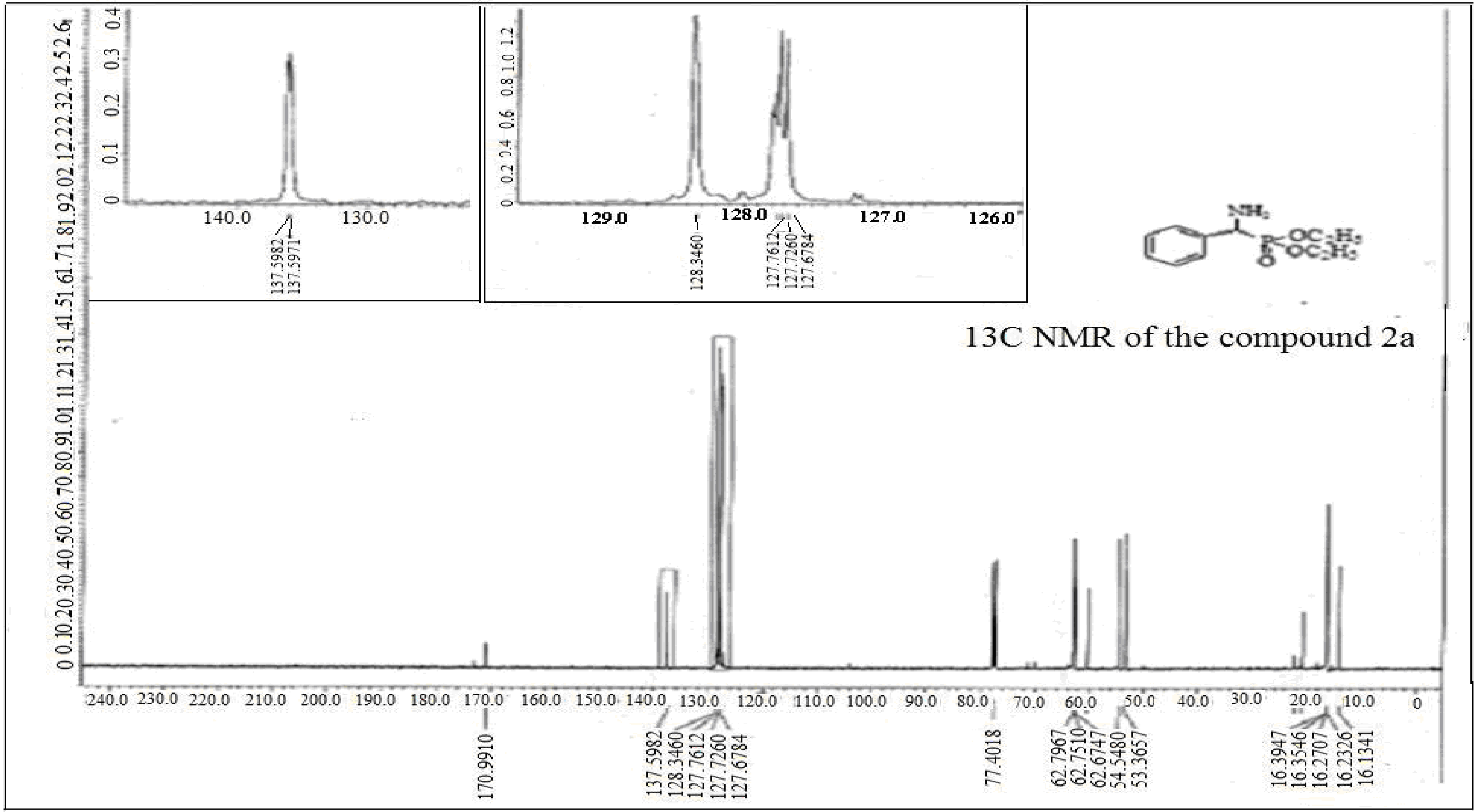
13C NMR of the compound 2a
137.5982
137.5971
128.3460
127.7612
127.7260
127.6784
170.9910
77.4018
62.7967
62.7510
62.6747
54.5480
53.3657
16.3947
16.3546
16.2707
16.2326
16.1341

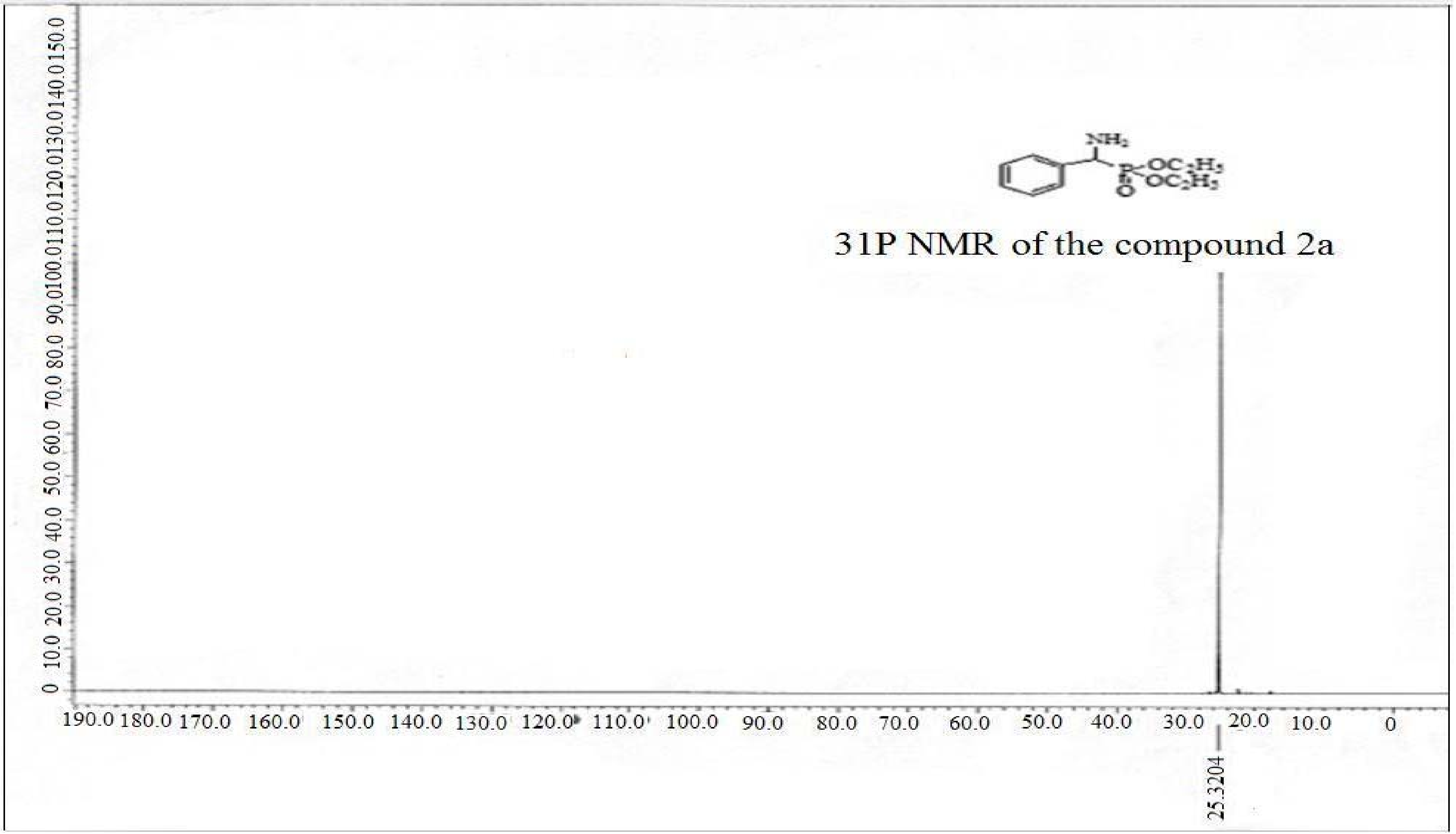
NH2
OC2H5
OC2H5
31P NMR of the compound 2a
190.0 180.0 170.0 160.0 150.0 140.0 130.0 120.0 110.0 100.0 90.0 80.0 70.0 60.0 50.0 40.0 30.0 20.0 10.0 0
25.3204
0 10.0 20.0 30.0 40.0 50.0 60.0 70.0 80.0 90.0 100.0 110.0 120.0 130.0 140.0 150.0

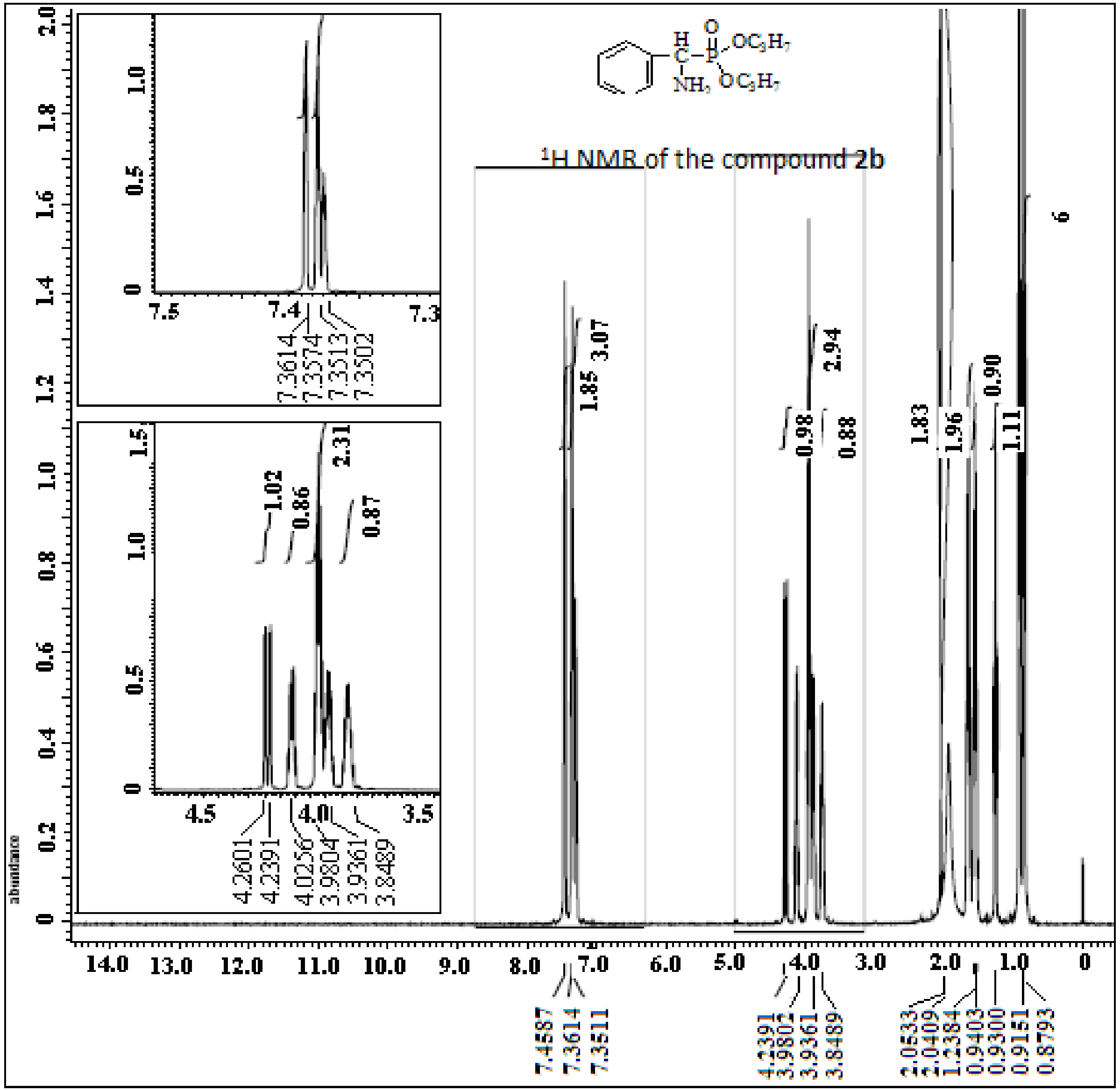

¹H NMR of the compound 2b
OC3H7
NH2
abundance
7.3614
7.3574
7.3513
7.3502
1.02
0.86
2.31
0.87
4.2601
4.2391
4.0256
3.9804
3.9361
3.8489
1.85
3.07
0.98
2.94
0.88
1.83
1.96
0.90
1.11
6
7.4587
7.3614
7.3511
4.2391
3.9802
3.9361
3.8489
2.0533
2.0409
1.2384
0.9403
0.9300
0.9151
0.8793

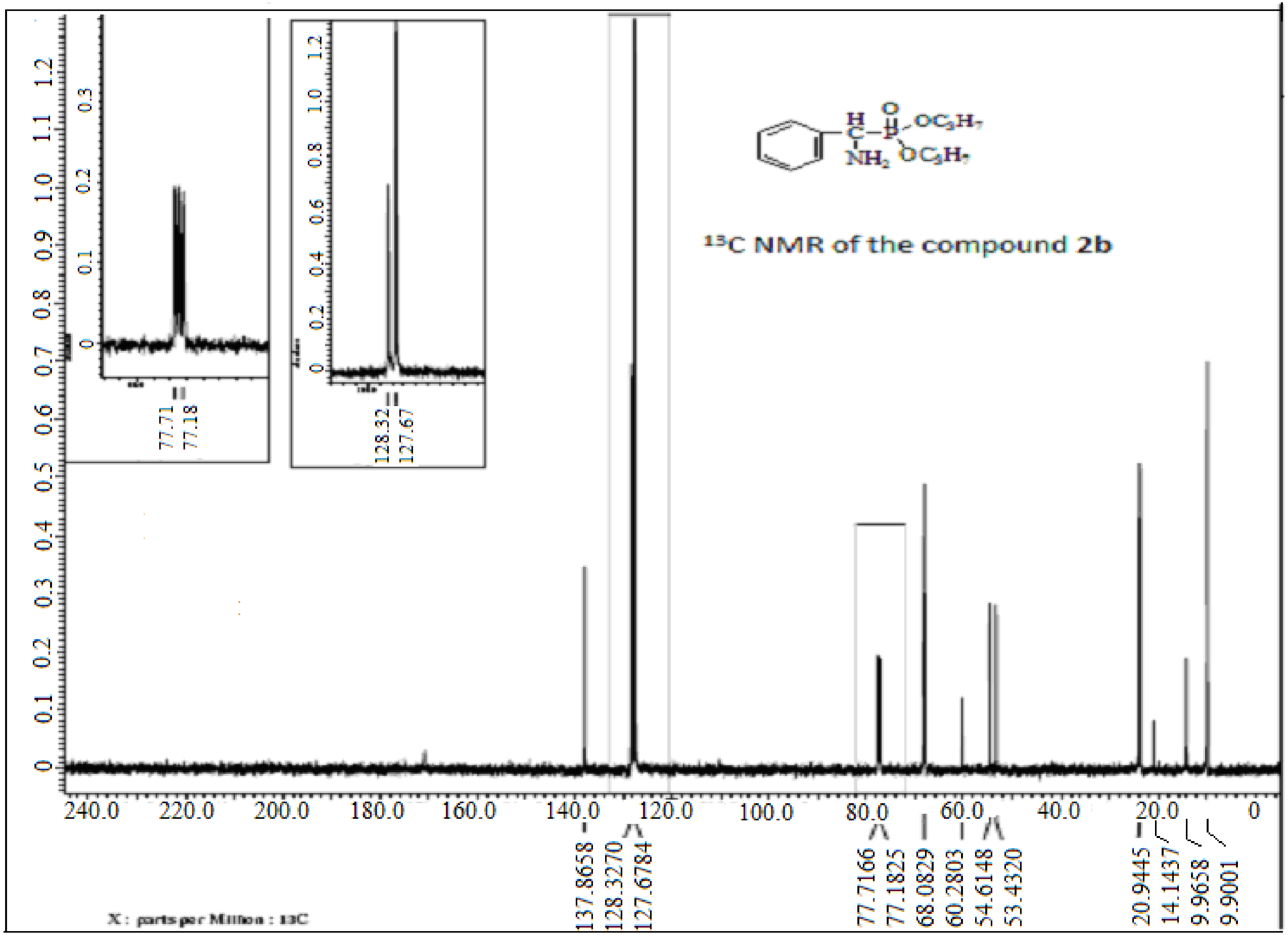
13C NMR of the compound 2b
X : parts per Million : 13C
137.8658
128.3270
127.6784
77.7166
77.1825
68.0829
60.2803
54.6148
53.4320
20.9445
14.1437
9.9658
9.9001
77.71
77.18
128.32
127.67

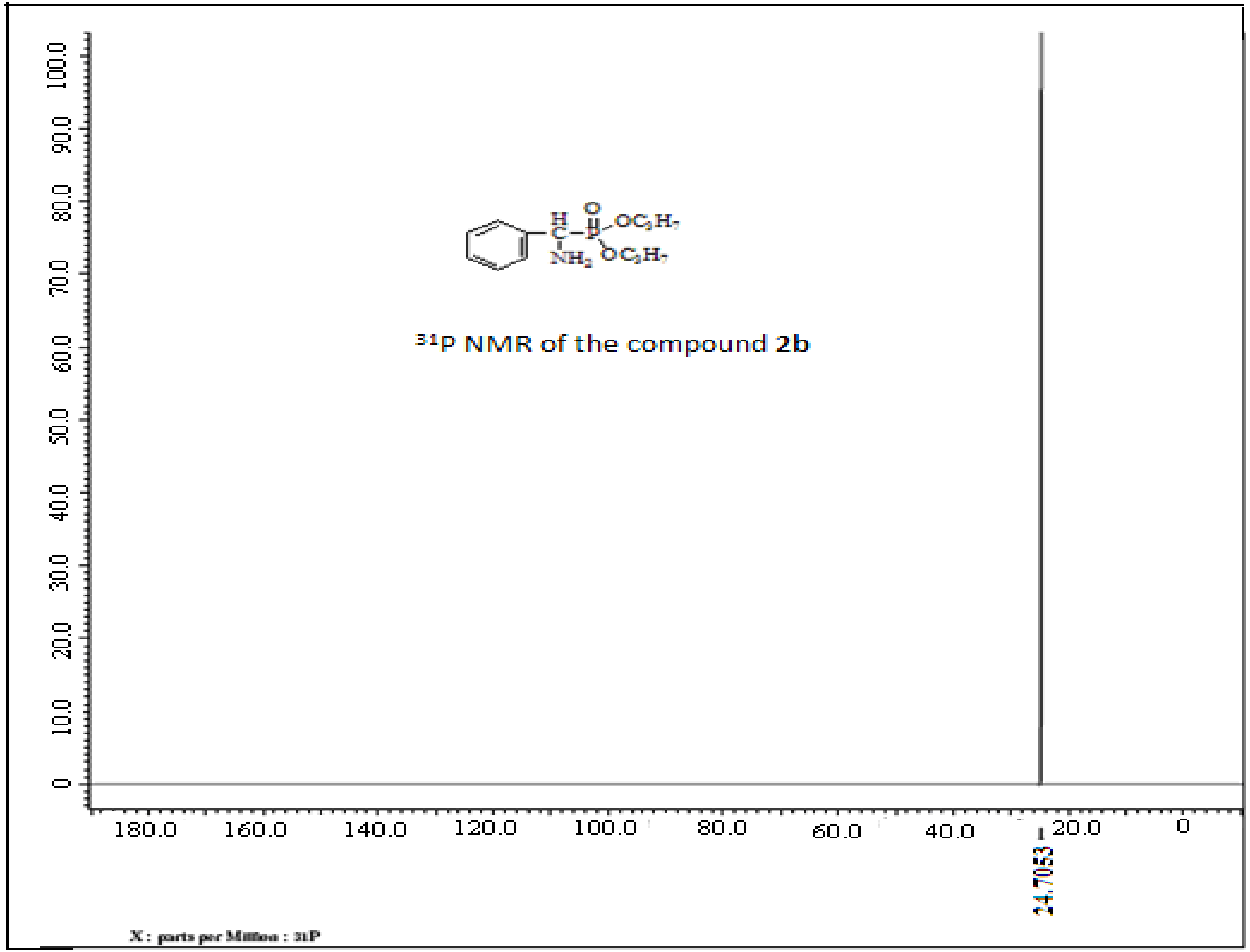

OC3H7
OC3H7
NH2
31P NMR of the compound 2b
100.0
90.0
80.0
70.0
60.0
50.0
40.0
30.0
20.0
10.0
0
180.0
160.0
140.0
120.0
100.0
80.0
60.0
40.0
20.0
0
24.7053
X : parts per Million : 31P

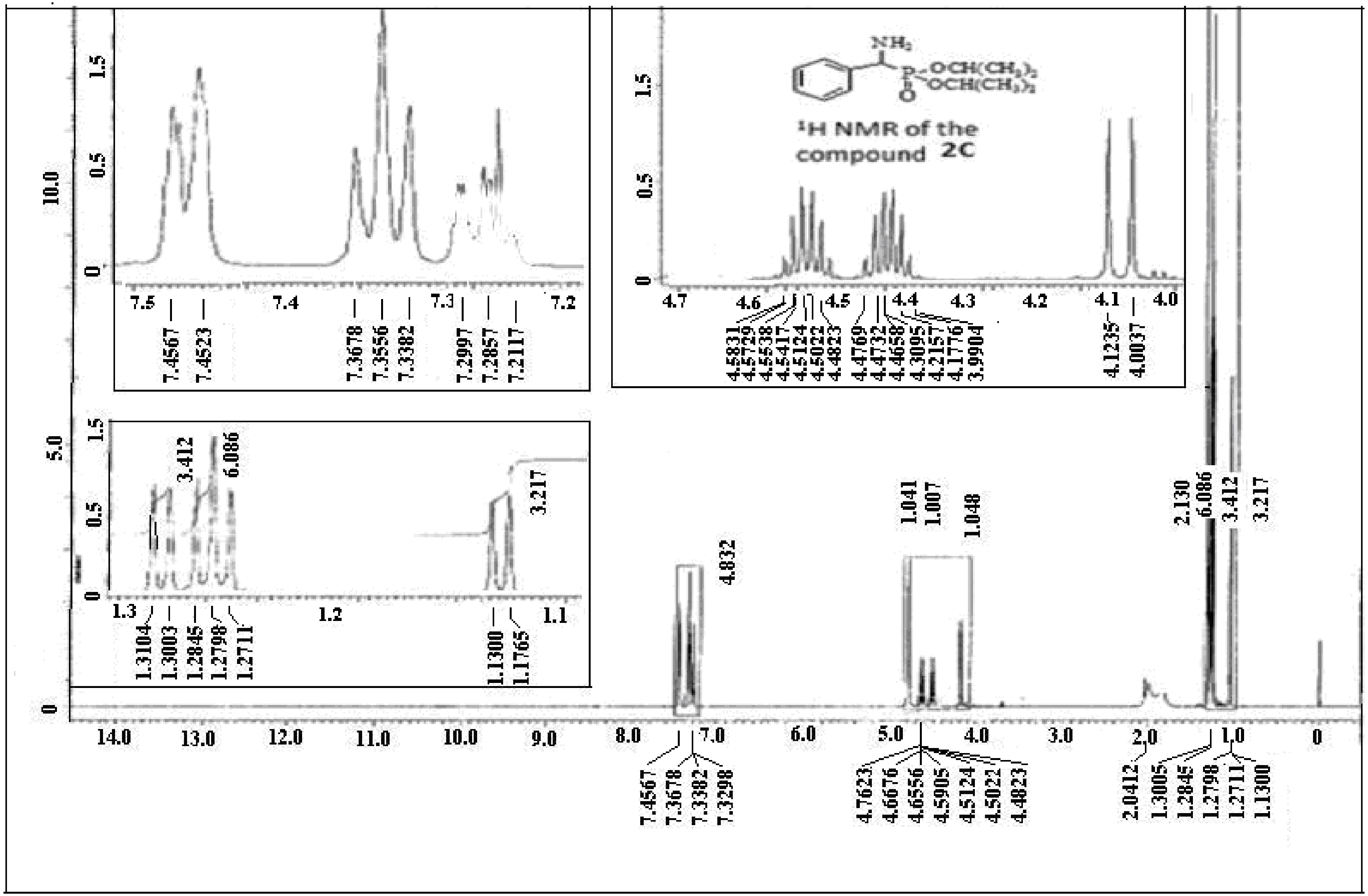
^{1}H NMR of the compound 2C

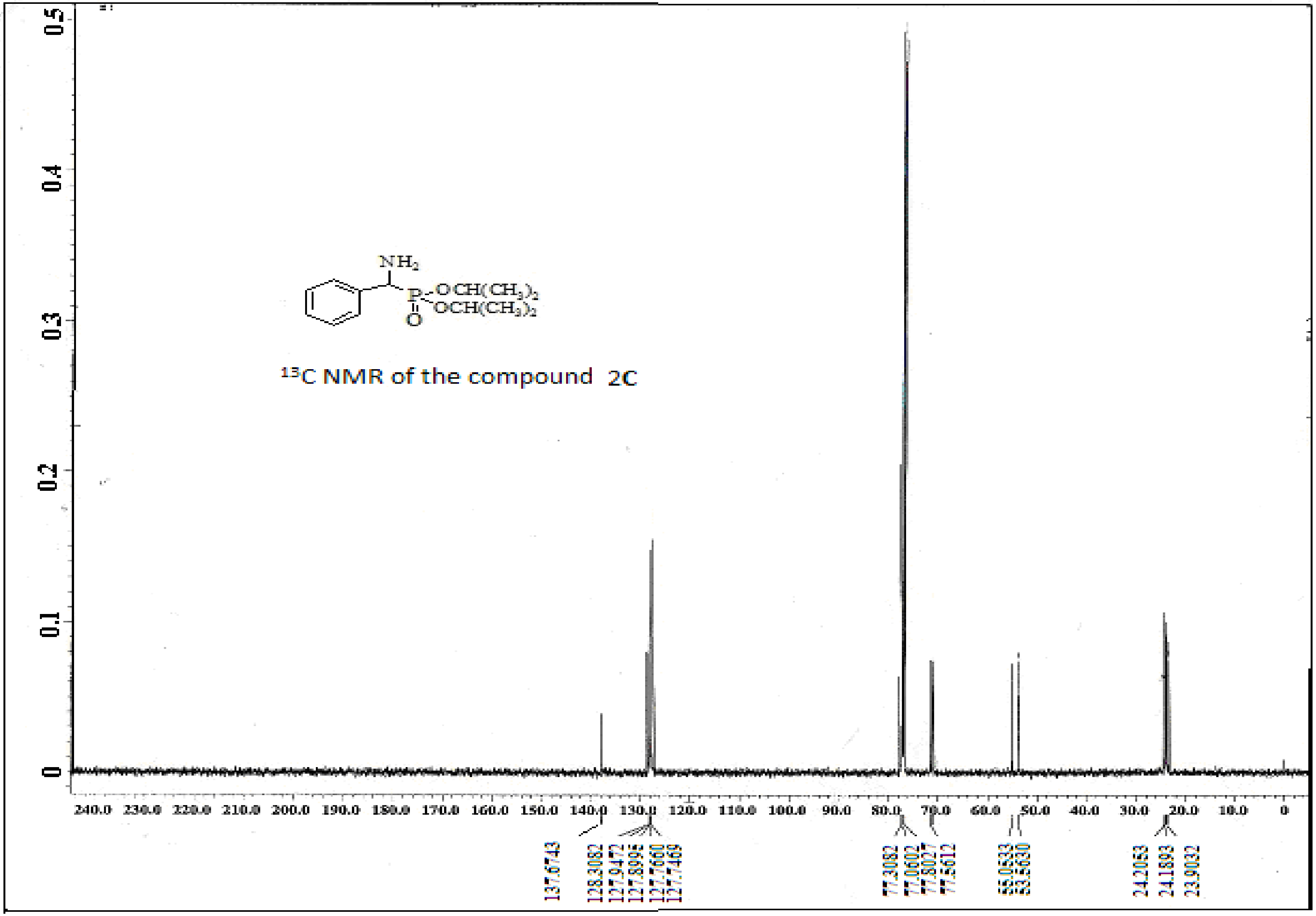
NH$_2$
P
O
OCH(CH$_3$)$_2$
OCH(CH$_3$)$_2$
^{13}C NMR of the compound 2C
0.5
0.4
0.3
0.2
0.1
0
240.0 230.0 220.0 210.0 200.0 190.0 180.0 170.0 160.0 150.0 140.0 130.0 120.0 110.0 100.0 90.0 80.0 70.0 60.0 50.0 40.0 30.0 20.0 10.0 0
137.6743
128.3082
127.9472
127.8995
127.7660
127.7469
77.3082
77.0602
77.8027
77.5612
55.0533
53.5630
24.2053
24.1893
23.9032

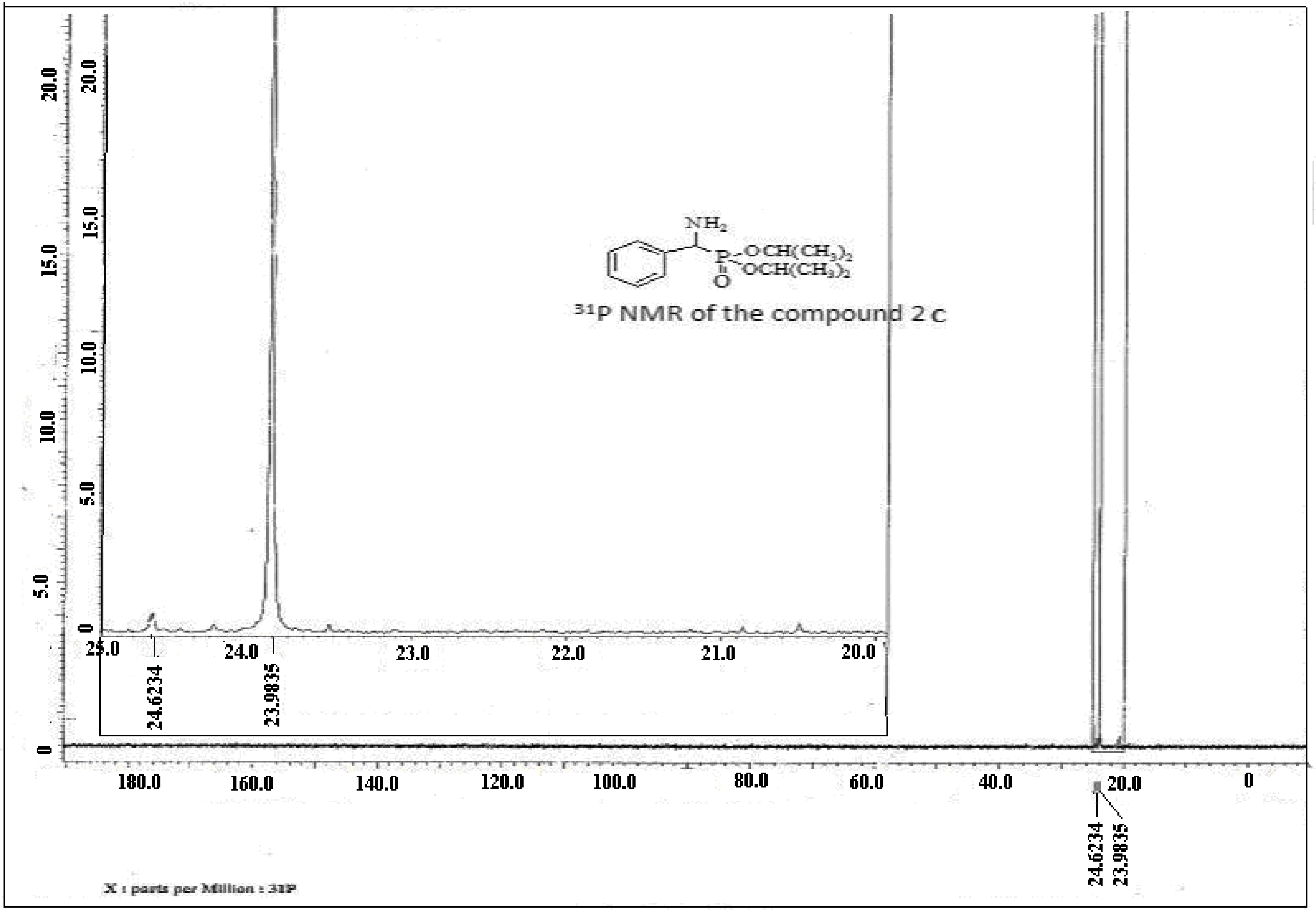
NH2
OCH(CH3)2
OCH(CH3)2
O
P
31P NMR of the compound 2 c
24.6234
23.9835
X : parts per Million : 31P

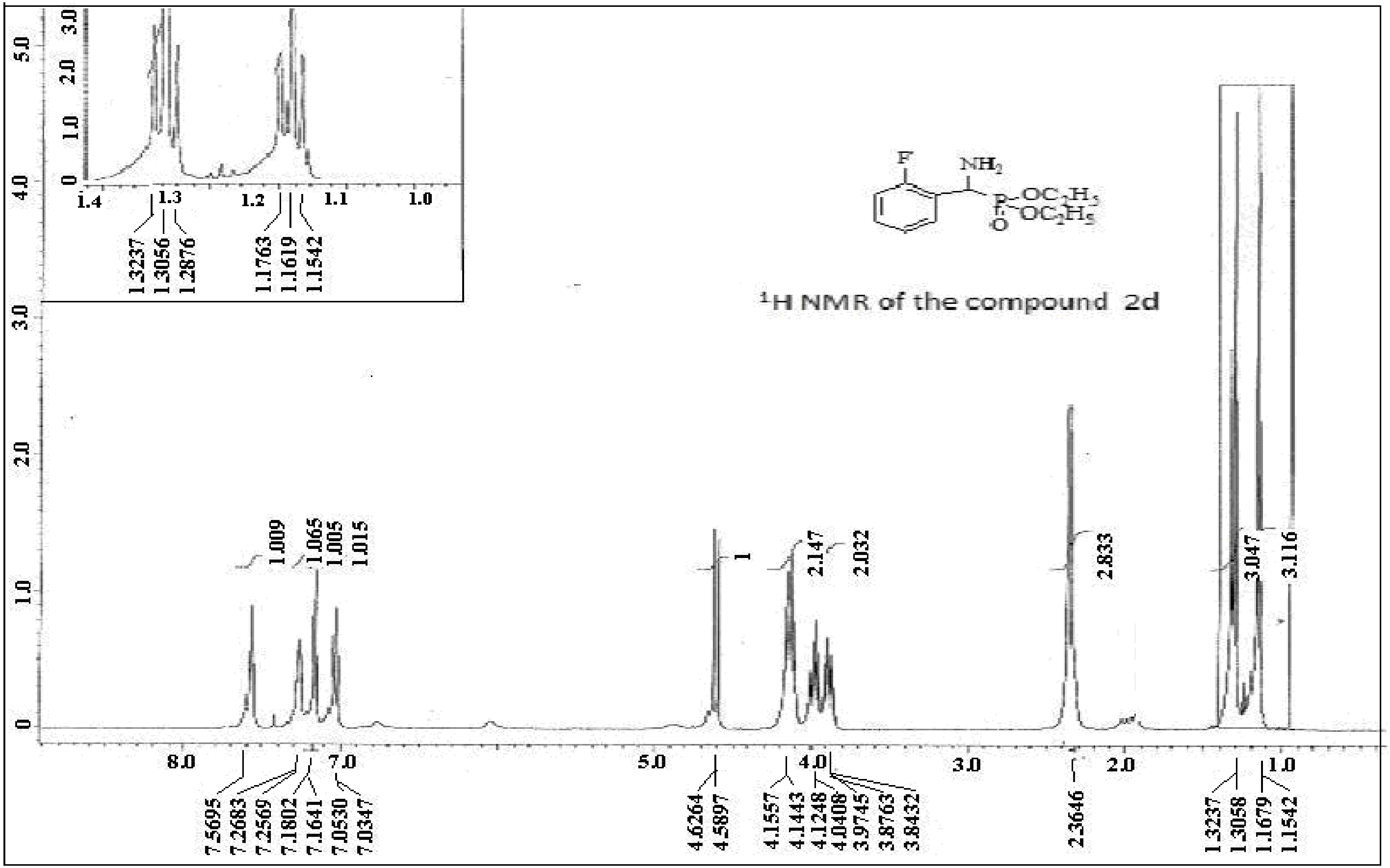
NH2
F
P-OC2H5
O OC2H5
1H NMR of the compound 2d
1.009
1.065
1.005
1.015
1
2.147
2.032
2.833
3.047
3.116
8.0
7.0
5.0
4.0
3.0
2.0
1.0
7.5695
7.2683
7.2569
7.1802
7.1641
7.0530
7.0347
4.6264
4.5897
4.1557
4.1443
4.1248
4.0408
3.9745
3.8763
3.8432
2.3646
1.3237
1.3058
1.1679
1.1542
1.4
1.3
1.2
1.1
1.0
1.3237
1.3056
1.2876
1.1763
1.1619
1.1542

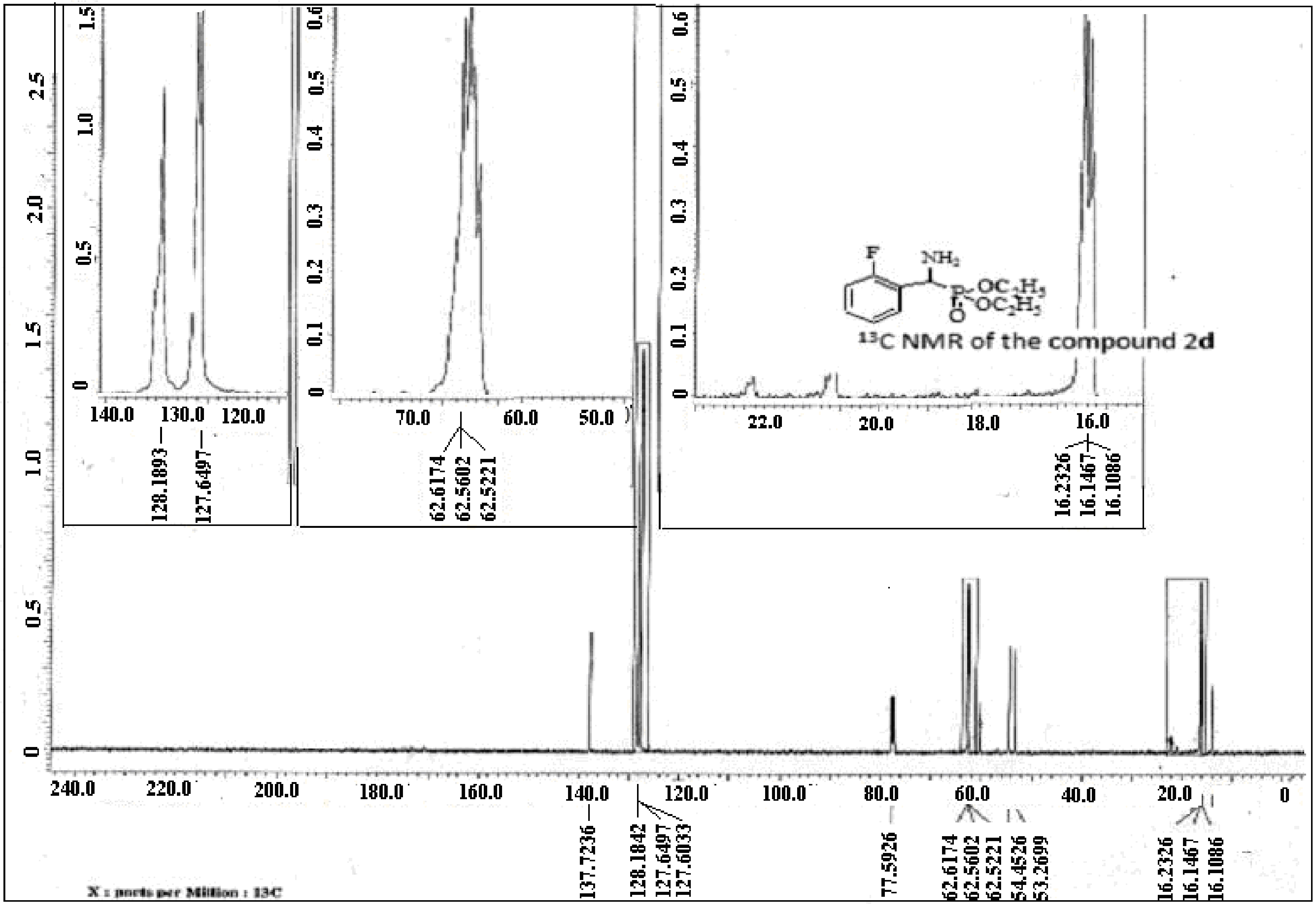

13C NMR of the compound 2d
X : parts per Million : 13C
137.7236
128.1842
127.6497
127.6033
77.5926
62.6174
62.5602
62.5221
54.4526
53.2699
16.2326
16.1467
16.1086
128.1893

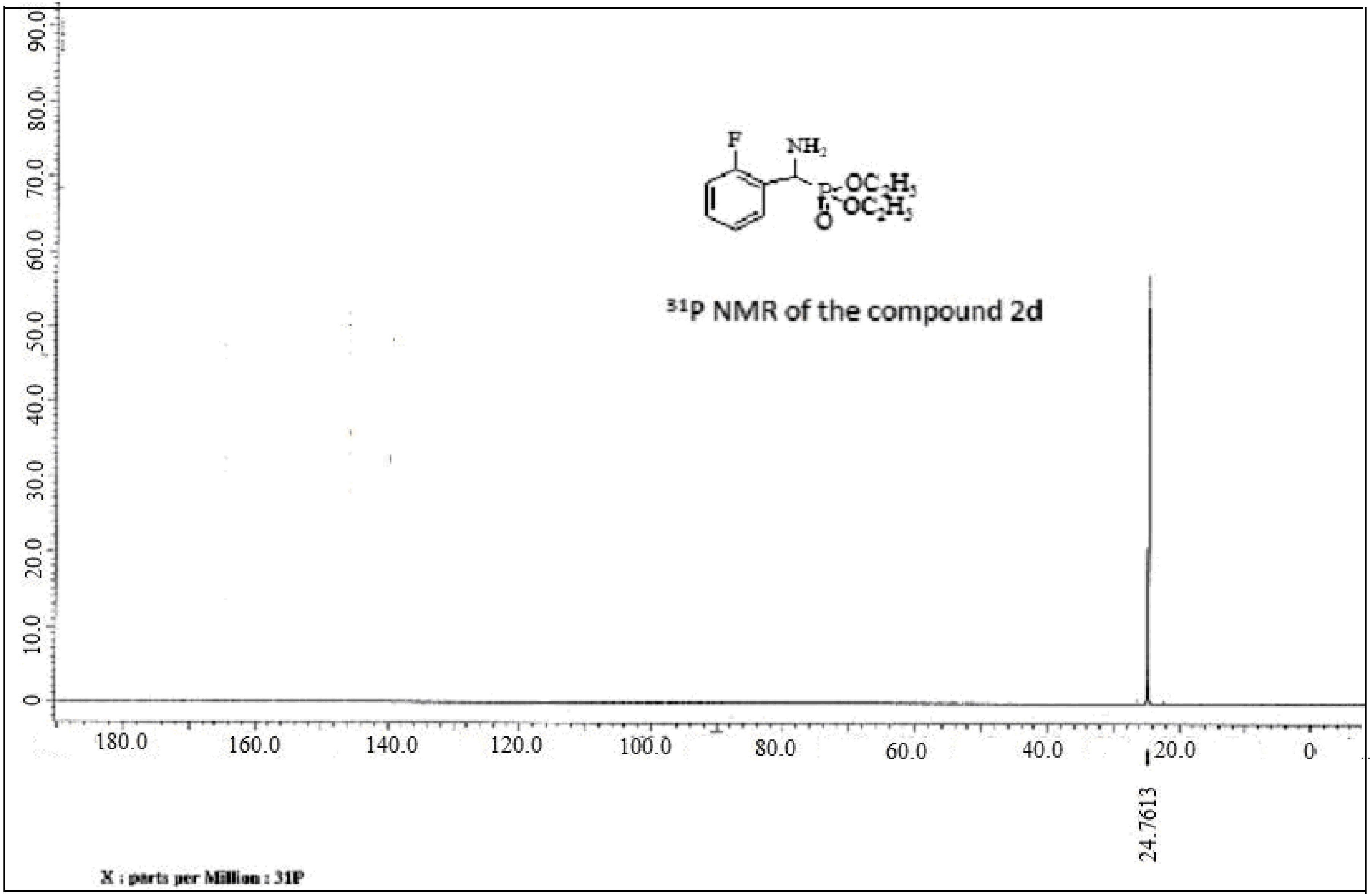
F
NH_2
OC_2H_5
OC_2H_5
P
O
^{31}P NMR of the compound 2d
90.0
80.0
70.0
60.0
50.0
40.0
30.0
20.0
10.0
0
180.0
160.0
140.0
120.0
100.0
80.0
60.0
40.0
20.0
0
24.7613
X : parts per Million : 31P

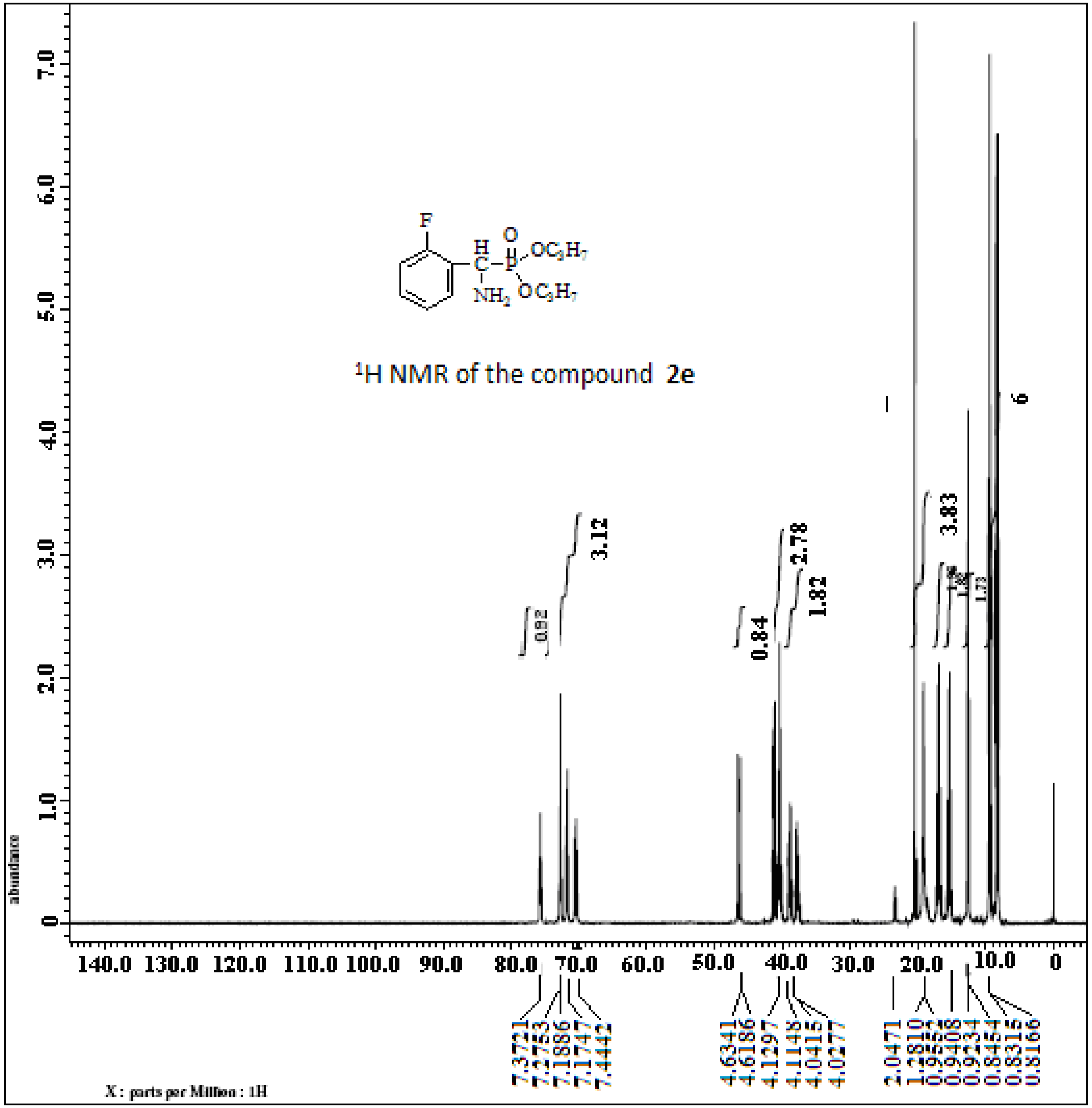

F
H
O
OC_3H_7
NH_2
OC_3H_7
^{1}H NMR of the compound 2e
abundance
X : parts per Million : 1H

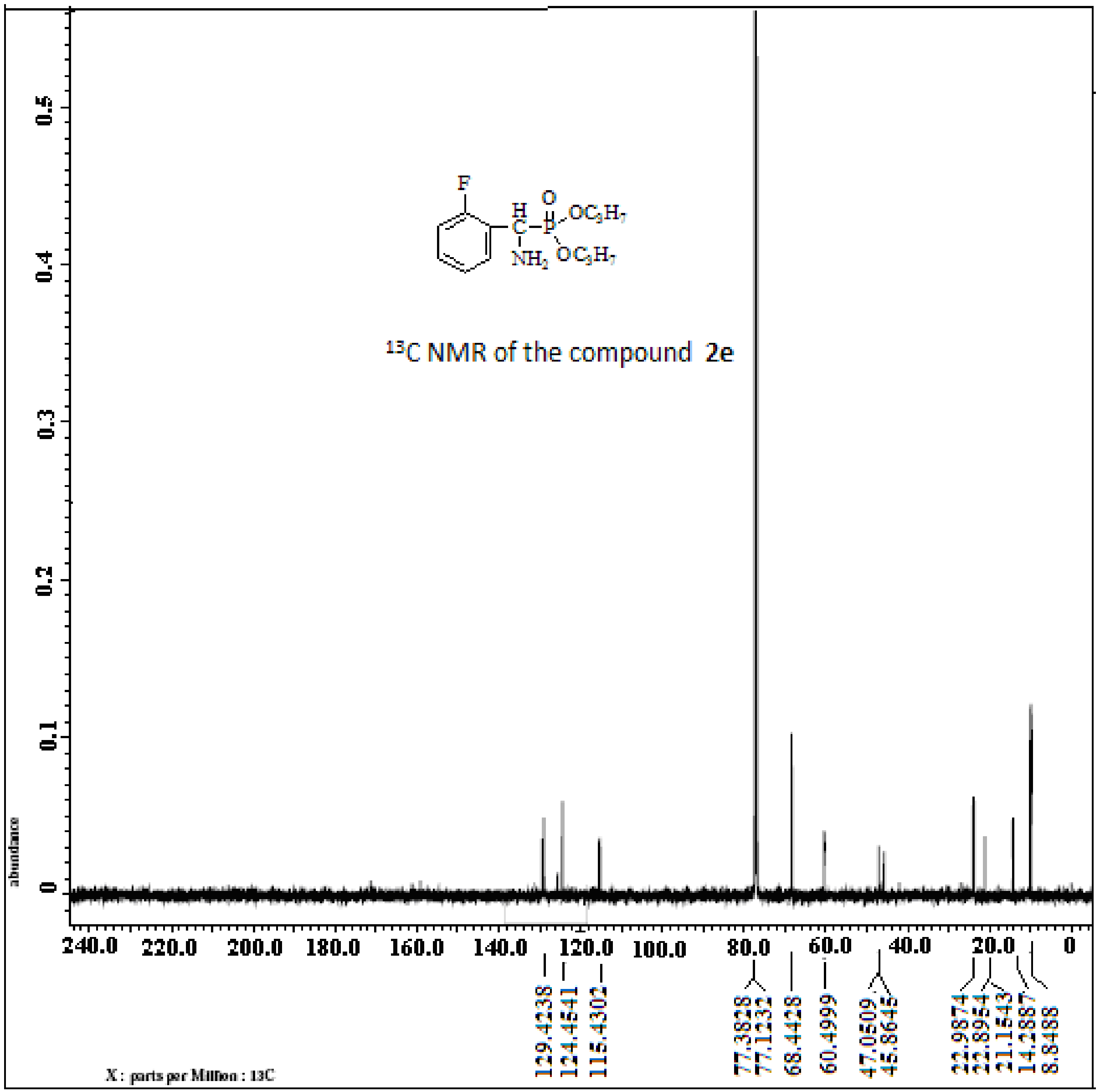
F
H
O
C
P
OC_3H_7
NH_2
OC_3H_7
^{13}C NMR of the compound 2e
abundance
0.5
0.4
0.3
0.2
0.1
0
240.0 220.0 200.0 180.0 160.0 140.0 120.0 100.0 80.0 60.0 40.0 20.0 0
129.4238
124.4541
115.4302
77.3828
77.1232
68.4428
60.4999
47.0509
45.8645
22.9874
22.8954
21.1543
14.2887
8.8488
X : parts per Million : 13C

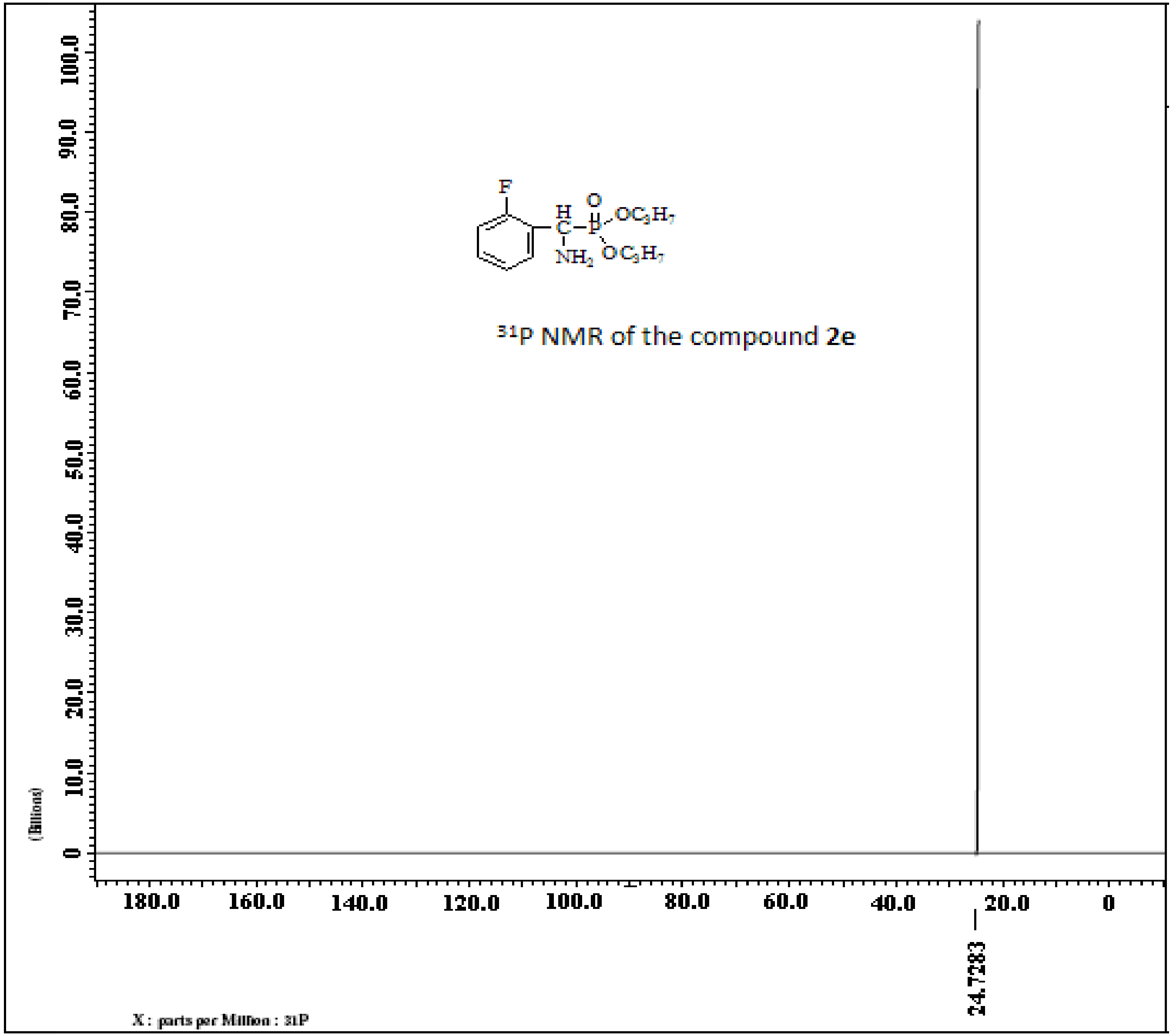
F
H
O
C
P
OC3H7
NH2
OC3H7
31P NMR of the compound 2e
100.0
90.0
80.0
70.0
60.0
50.0
40.0
30.0
20.0
10.0
0
(Billions)
180.0
160.0
140.0
120.0
100.0
80.0
60.0
40.0
20.0
0
24.7283
X : parts per Million : 31P

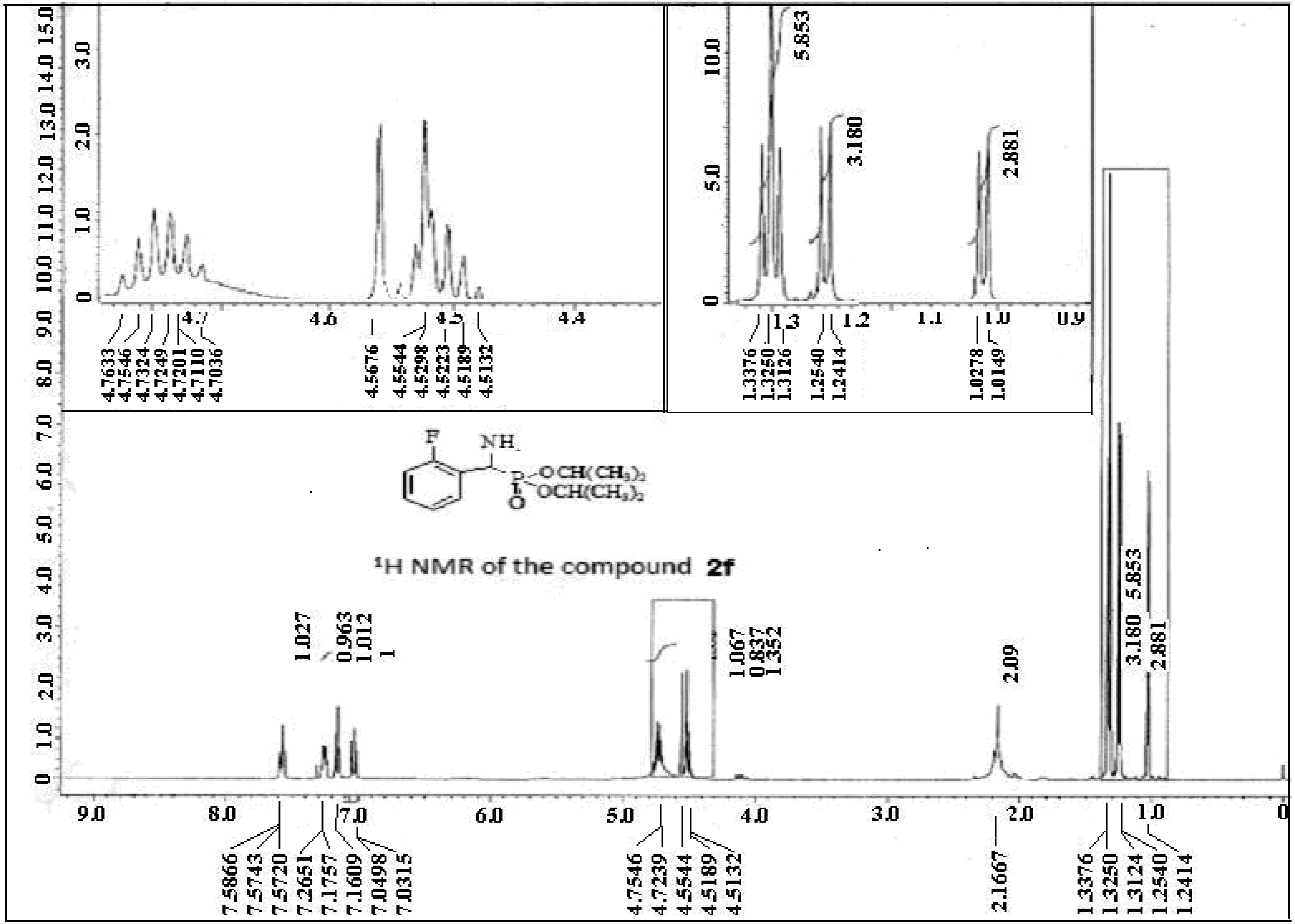

¹H NMR of the compound 2f
NH
OCH(CH3)2
F
O

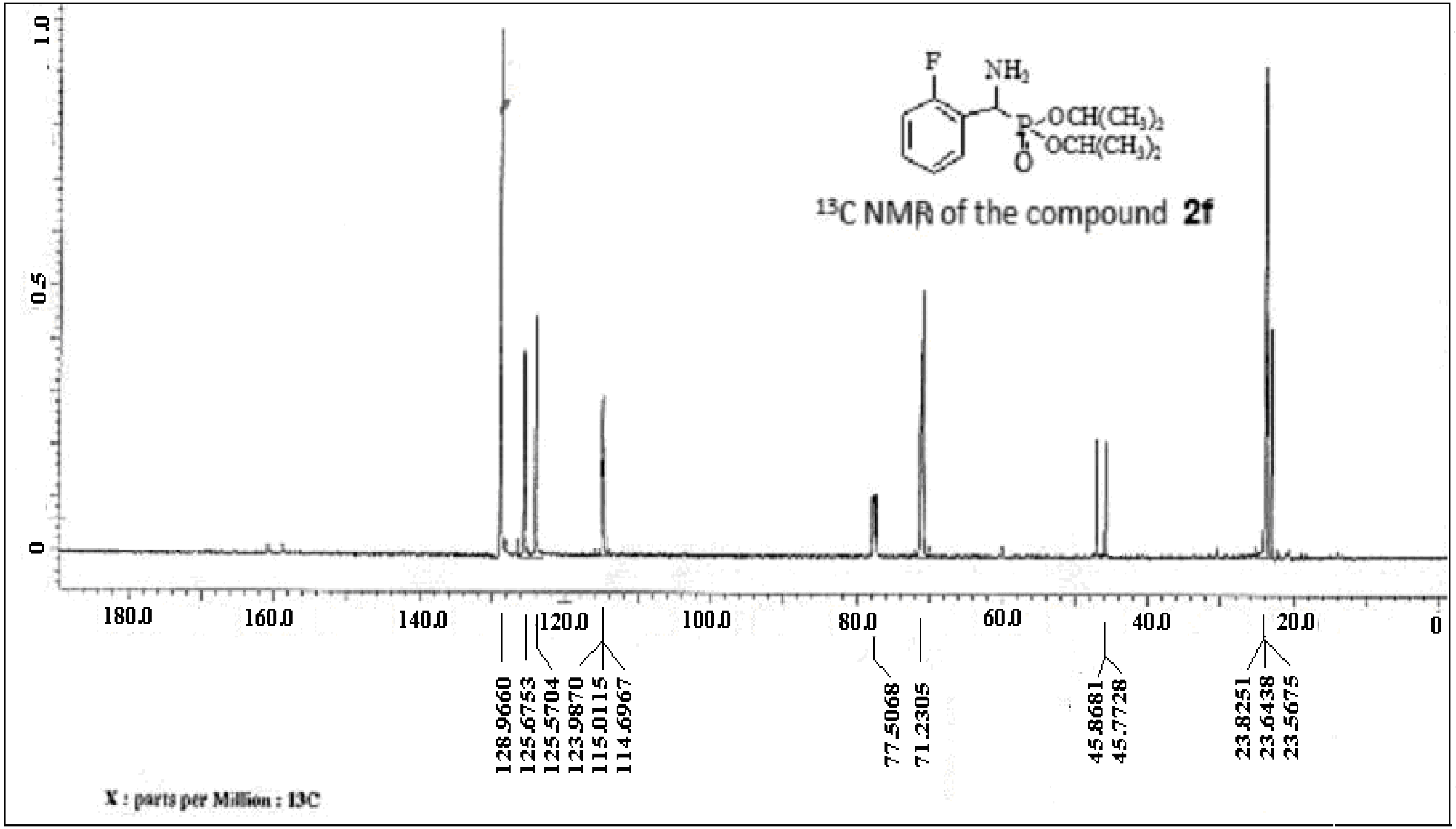
F
NH2
OCH(CH3)2
OCH(CH3)2
O
13C NMR of the compound 2f
1.0
0.5
0
180.0
160.0
140.0
120.0
100.0
80.0
60.0
40.0
20.0
0
128.9660
125.6753
125.5704
123.9870
115.0115
114.6967
77.5068
71.2305
45.8681
45.7728
23.8251
23.6438
23.5675
X : parts per Million : 13C

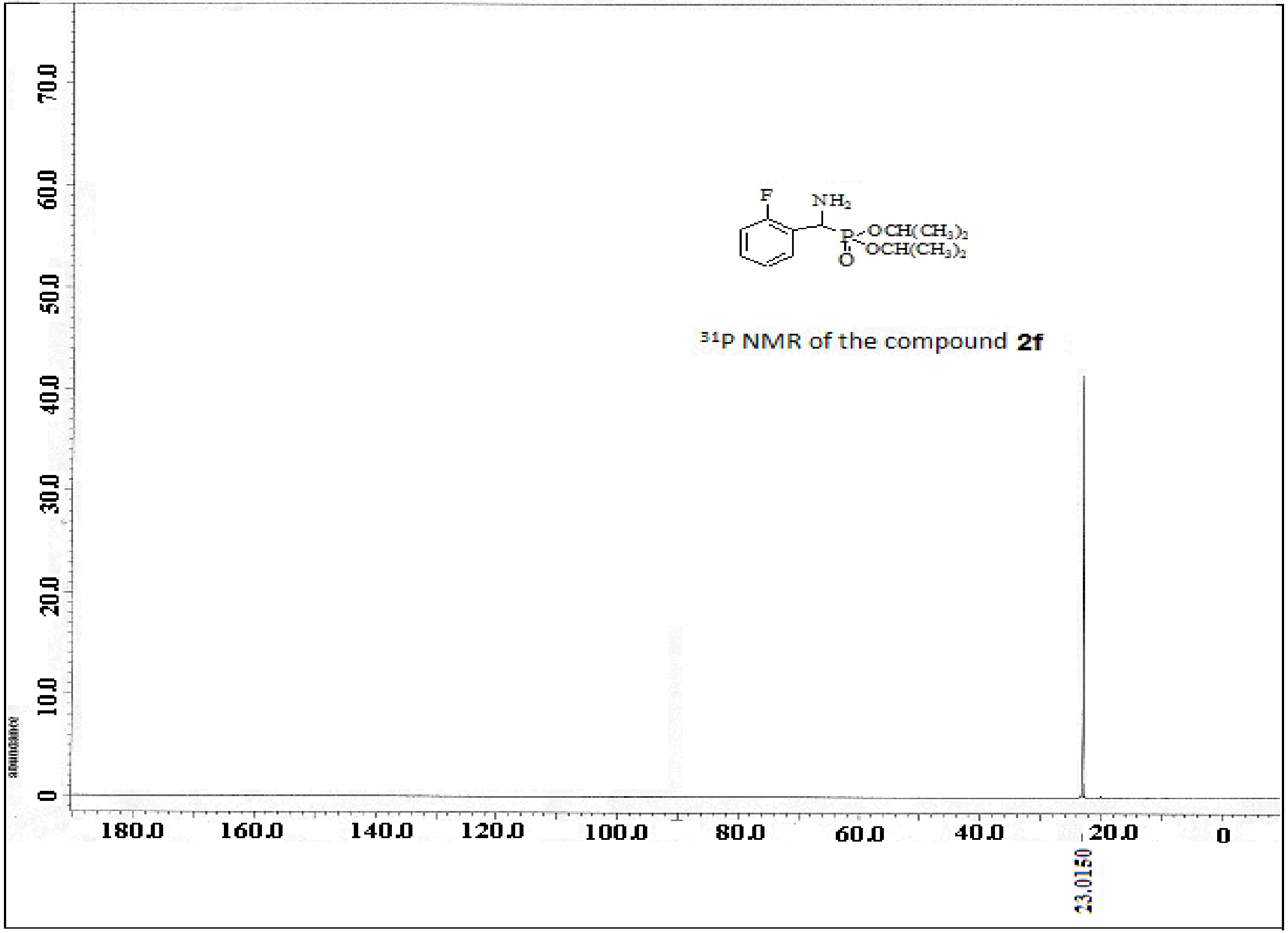

F
NH2
OCH(CH3)2
OCH(CH3)2
O
31P NMR of the compound 2f
abundance
180.0
160.0
140.0
120.0
100.0
80.0
60.0
40.0
20.0
0
23.0150

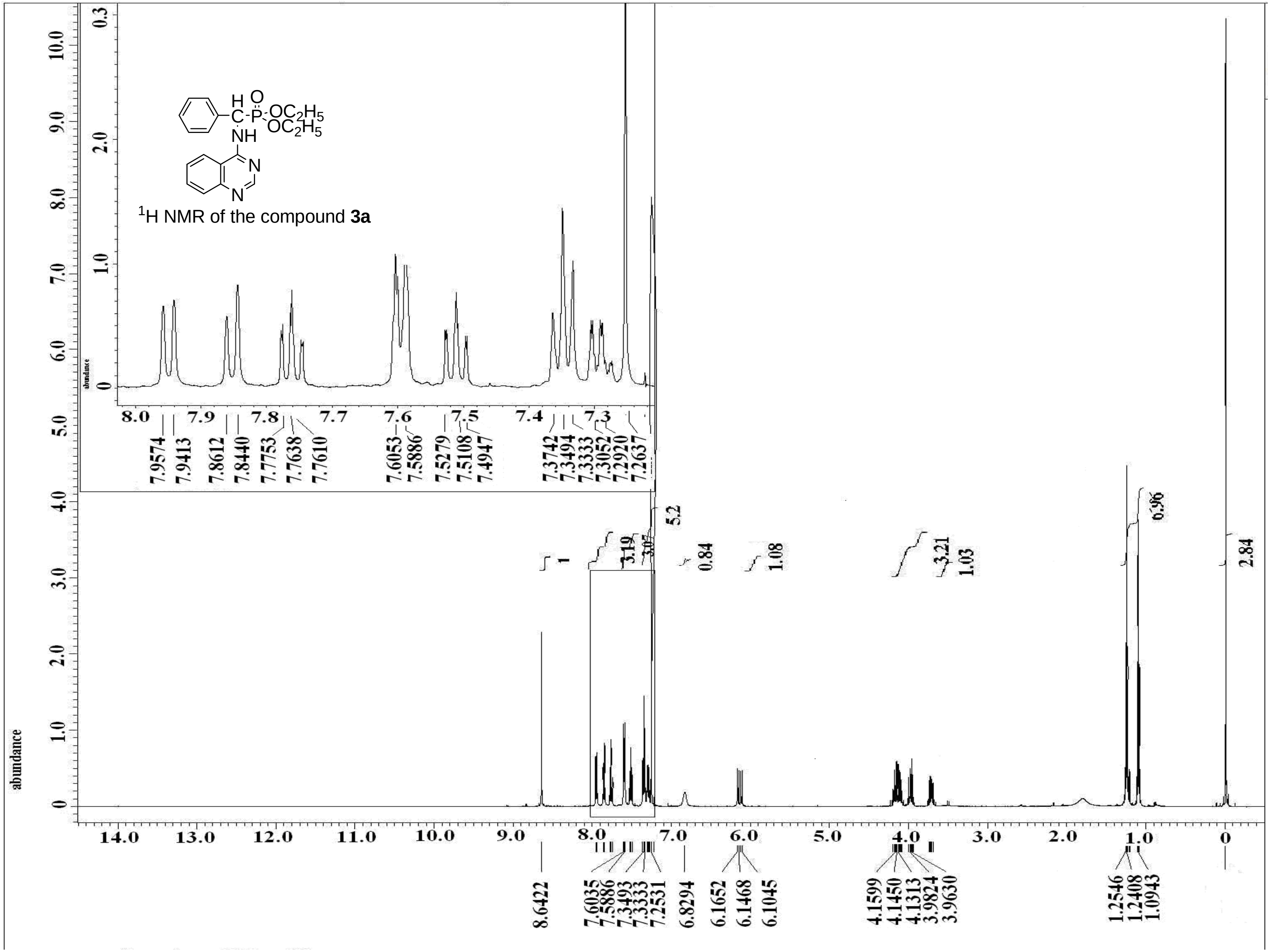

^{1}H NMR of the compound **3a**

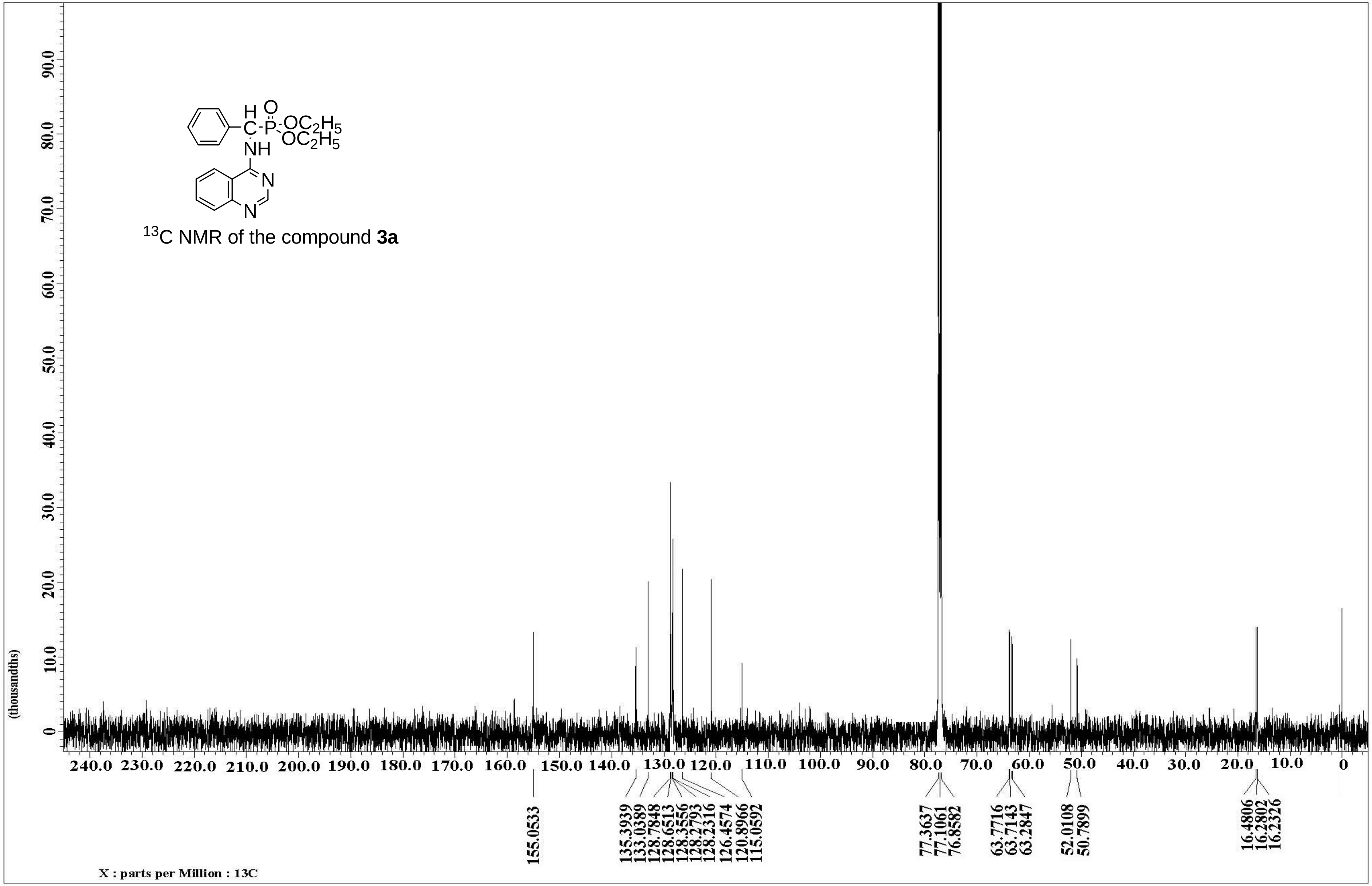

^{13}C NMR of the compound **3a**
(thousandths)
X : parts per Million : 13C
155.0533
135.3939
133.0389
128.7848
128.6513
128.3556
128.2793
128.2316
126.4574
120.8966
115.0592
77.3637
77.1061
76.8582
63.7716
63.7143
63.2847
52.0108
50.7899
16.4806
16.2802
16.2326

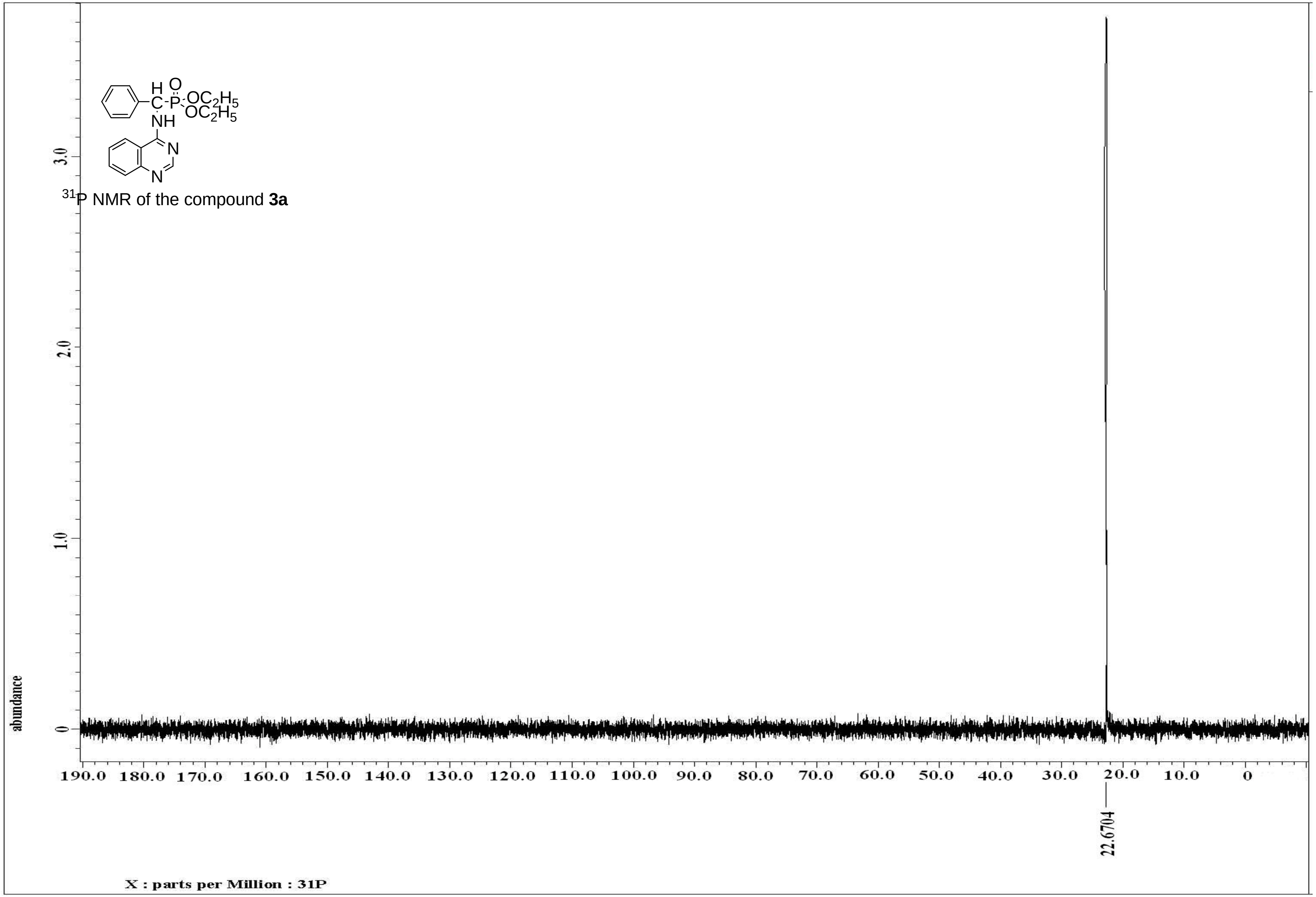

H O
C P OC2H5
OC2H5
NH
N
N
^{31}P NMR of the compound **3a**
abundance
3.0
2.0
1.0
0
190.0 180.0 170.0 160.0 150.0 140.0 130.0 120.0 110.0 100.0 90.0 80.0 70.0 60.0 50.0 40.0 30.0 20.0 10.0 0
22.6704
X : parts per Million : 31P

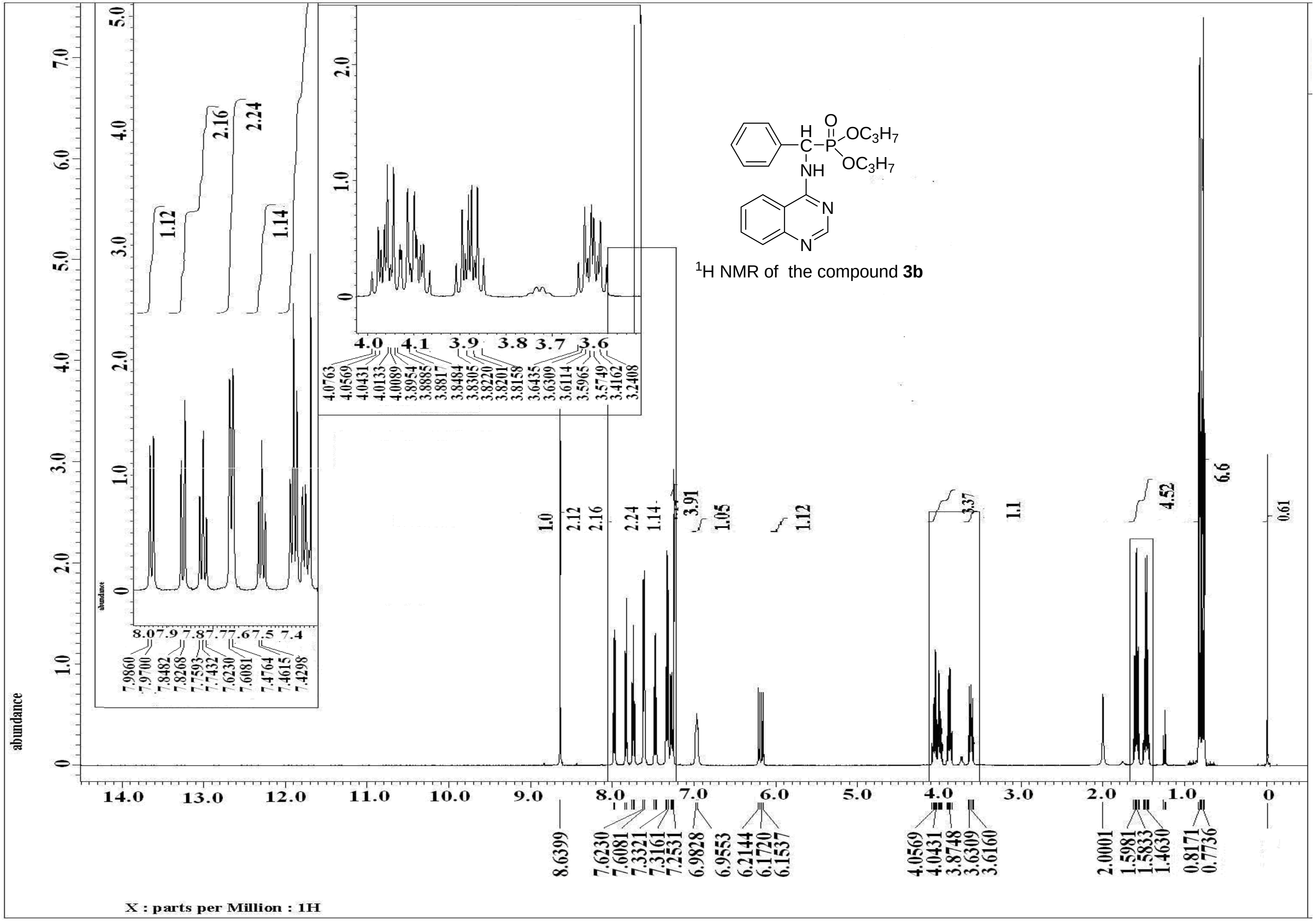

^{1}H NMR of the compound **3b**

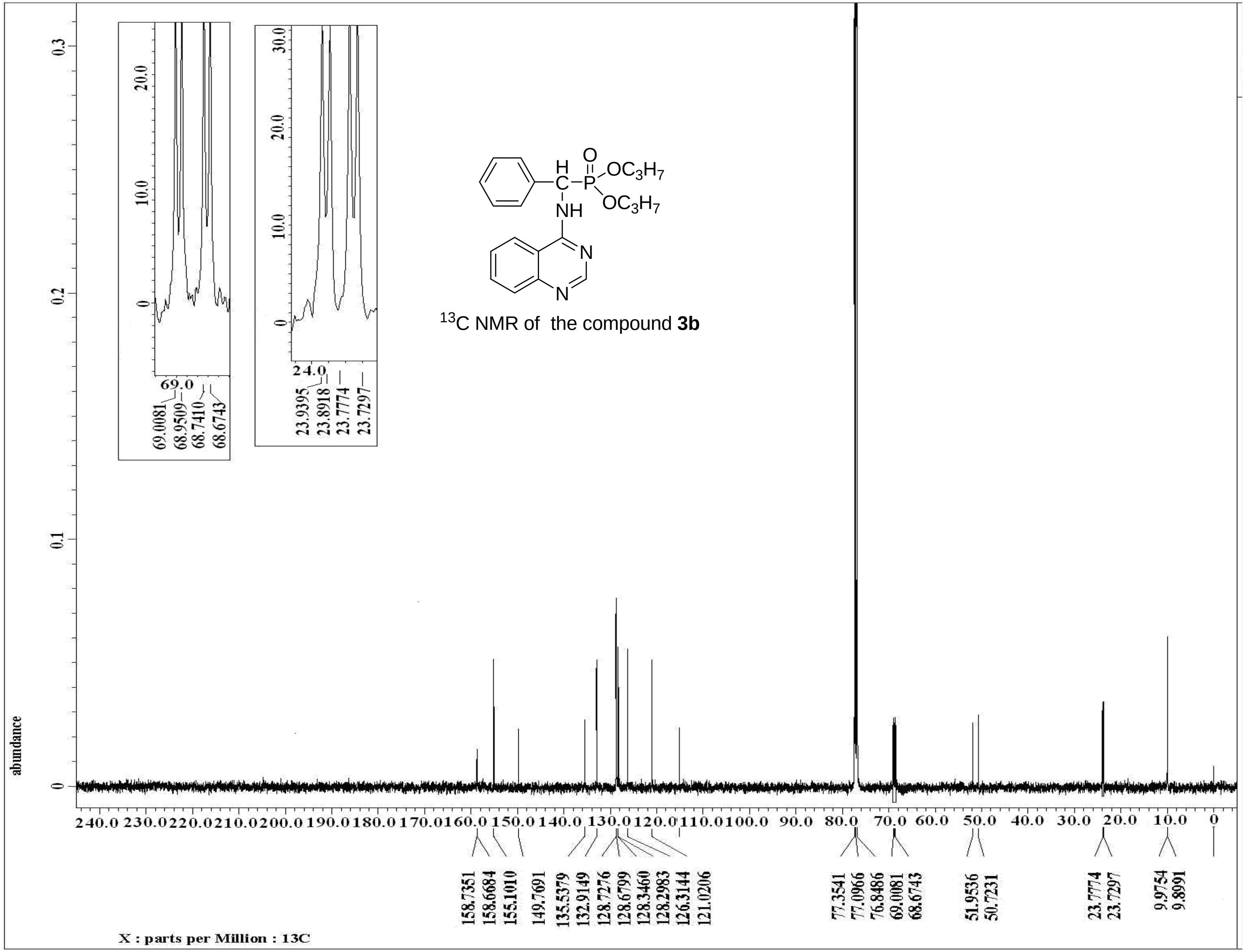

^{13}C NMR of the compound **3b**
abundance
X : parts per Million : 13C
158.7351
158.6684
155.1010
149.7691
135.5379
132.9149
128.7276
128.6799
128.3460
128.2983
126.3144
121.0206
77.3541
77.0966
76.8486
69.0081
68.6743
51.9536
50.7231
23.7774
23.7297
9.9754
9.8991
69.0081
68.9509
68.7410
68.6743
23.9395
23.8918
23.7774
23.7297

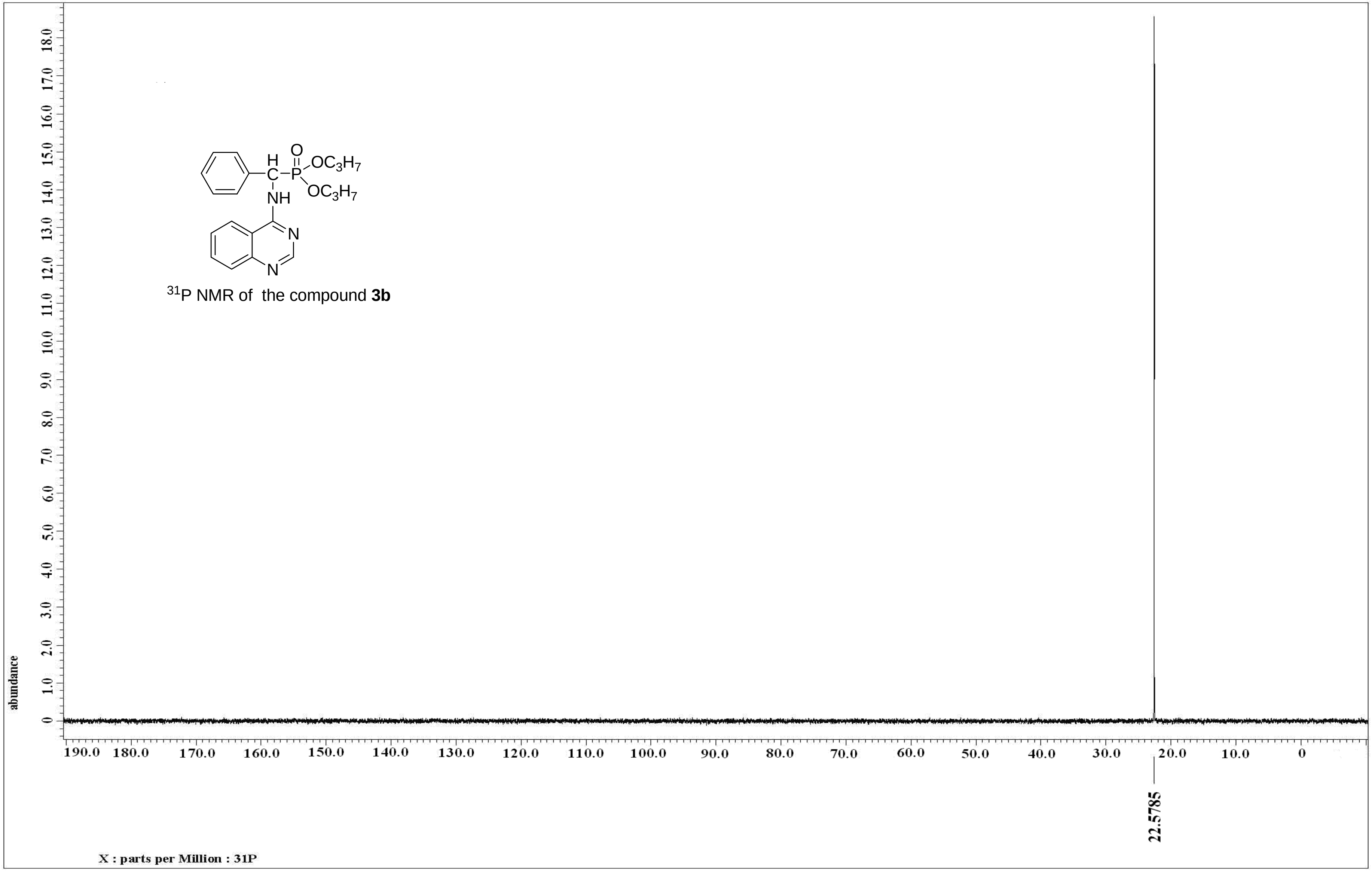
^{31}P NMR of the compound **3b**
abundance
X : parts per Million : 31P
22.5785

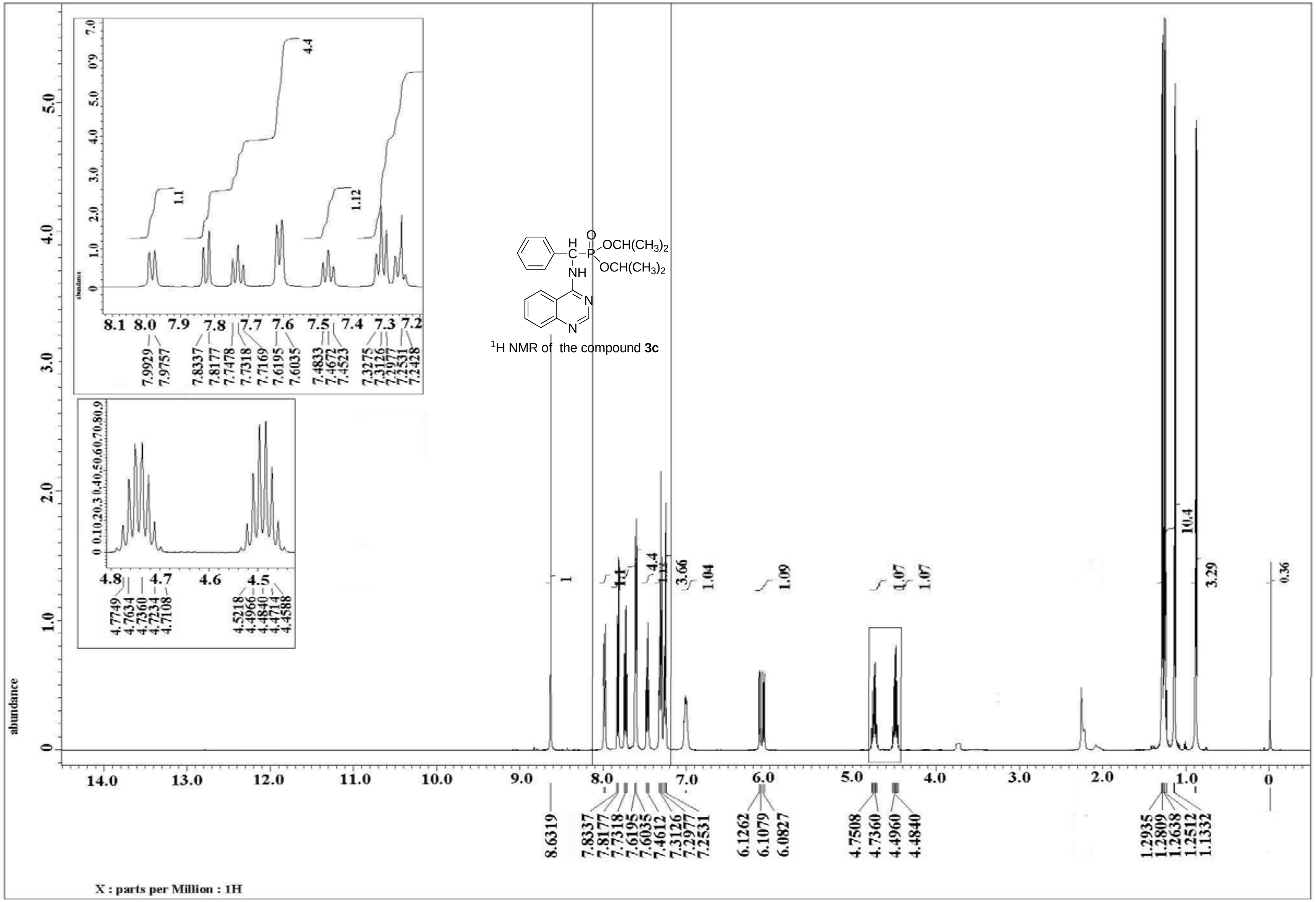

¹H NMR of the compound 3c
OCH(CH3)2
NH
X : parts per Million : 1H
abundance

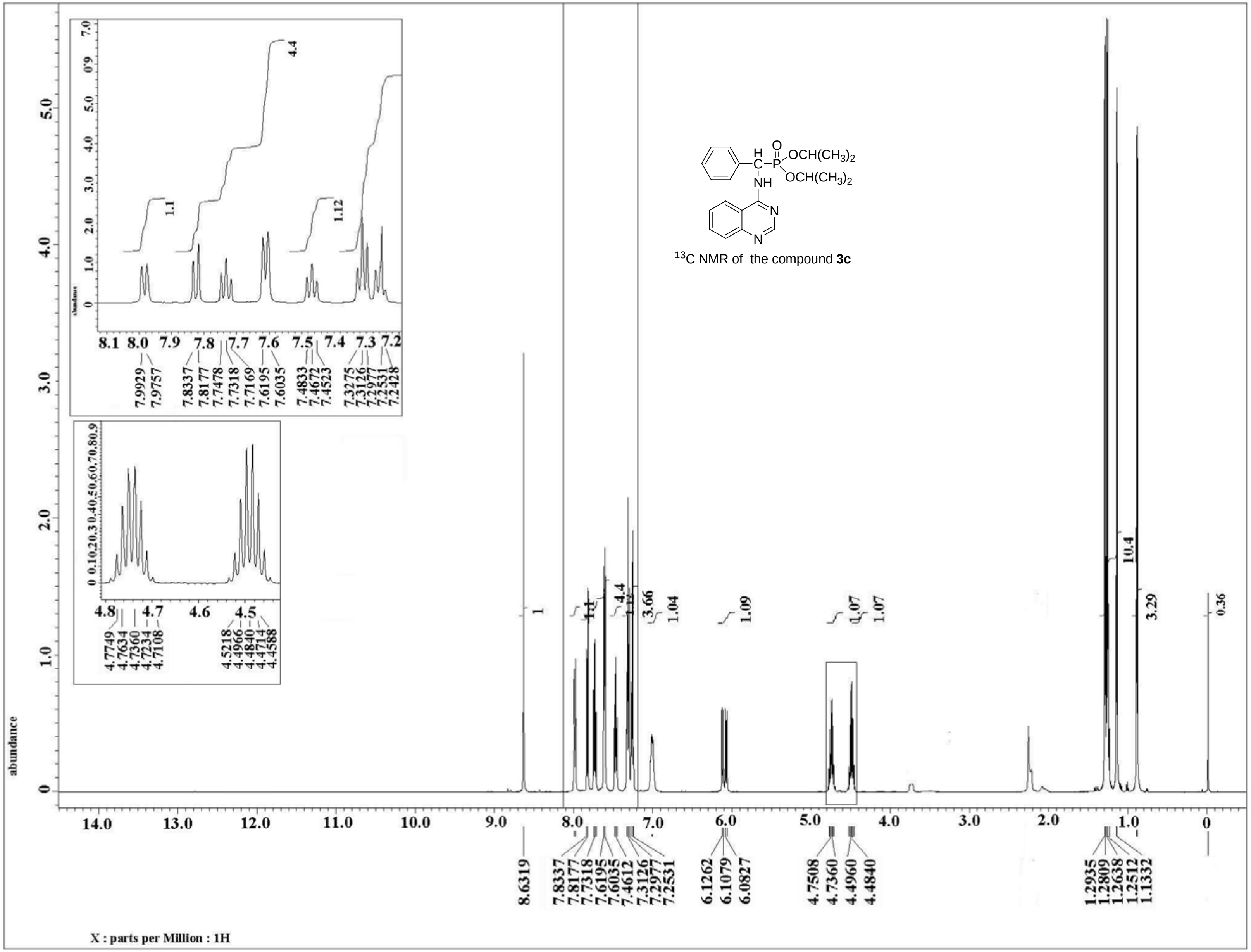

OCH(CH3)2
OCH(CH3)2
NH
13C NMR of the compound 3c
abundance
X : parts per Million : 1H

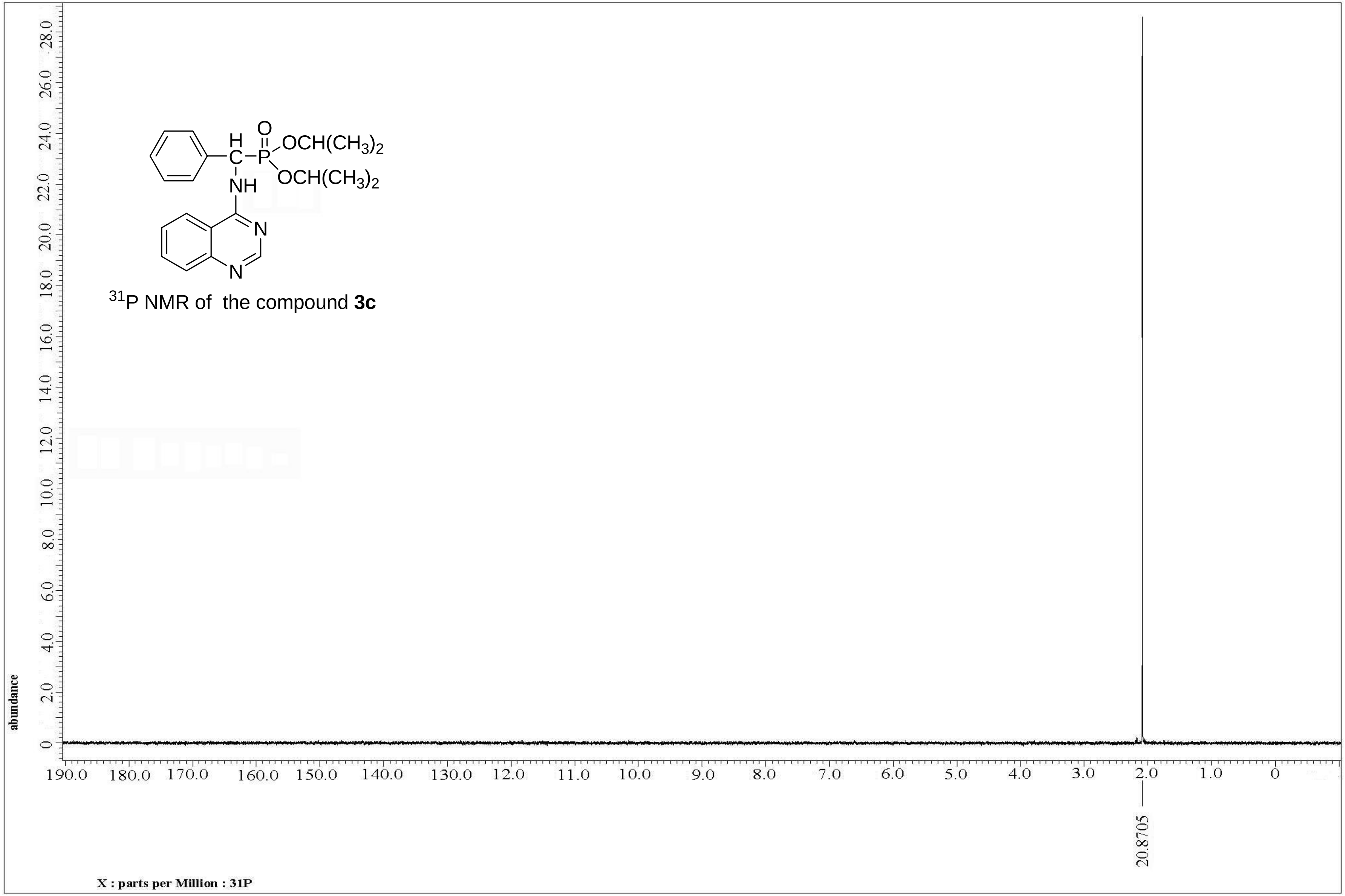

31P NMR of the compound 3c
abundance
X : parts per Million : 31P
20.8705

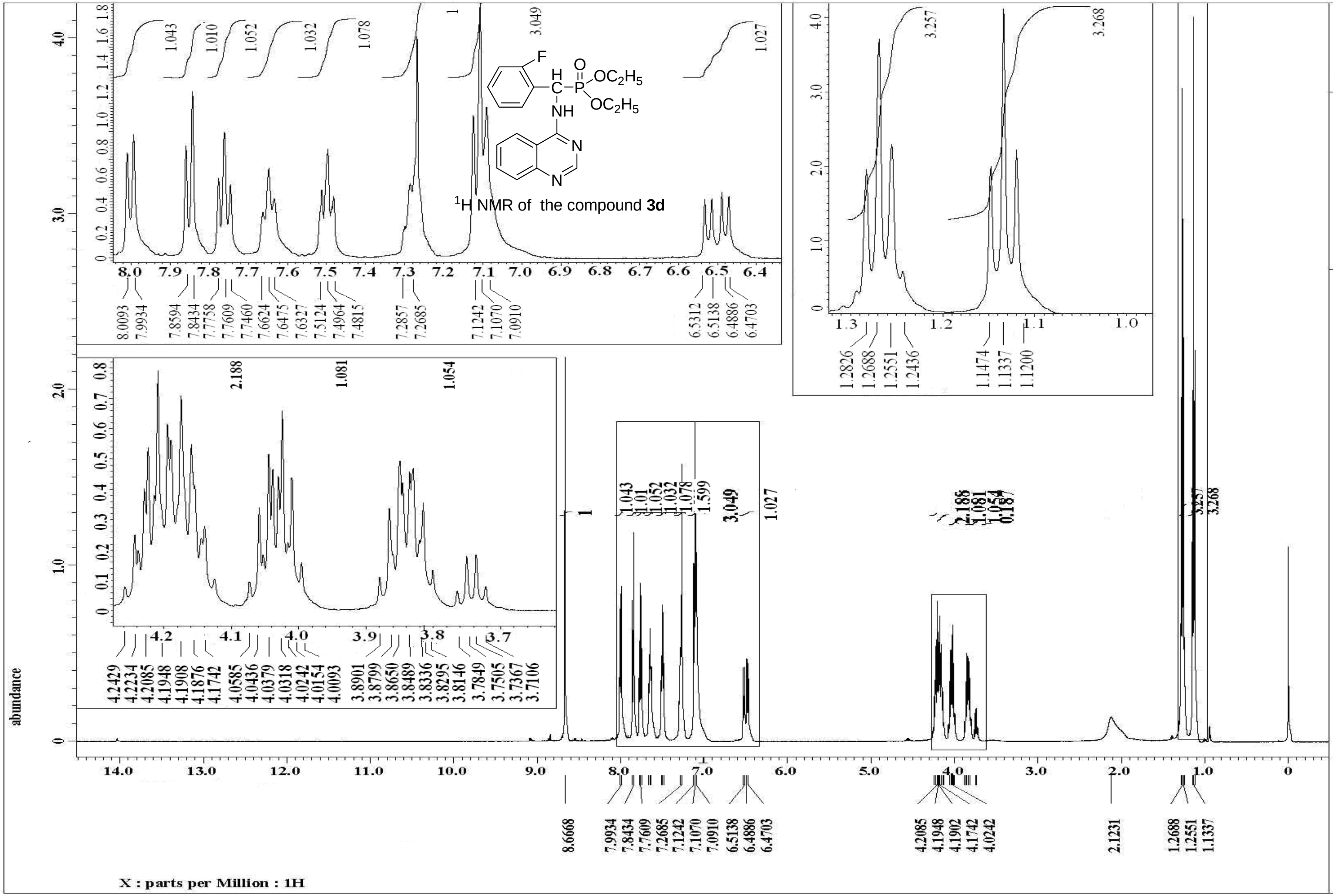
^{1}H NMR of the compound **3d**
X : parts per Million : 1H
abundance

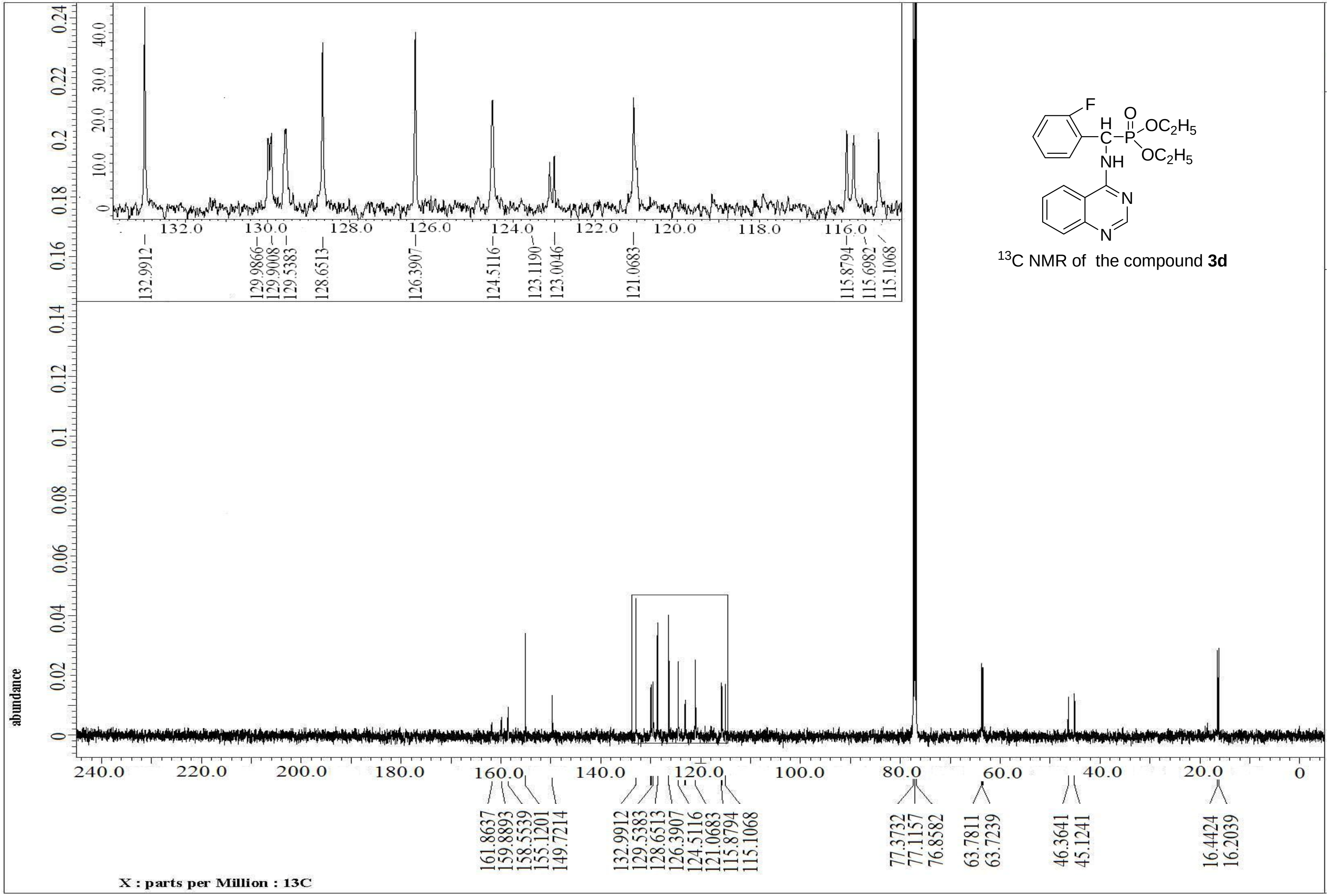

13C NMR of the compound 3d
abundance
X : parts per Million : 13C
161.8637
159.8893
158.5539
155.1201
149.7214
132.9912
129.5383
128.6513
126.3907
124.5116
121.0683
115.8794
115.1068
77.3732
77.1157
76.8582
63.7811
63.7239
46.3641
45.1241
16.4424
16.2039
129.9866
129.9008
123.1190
123.0046
115.6982

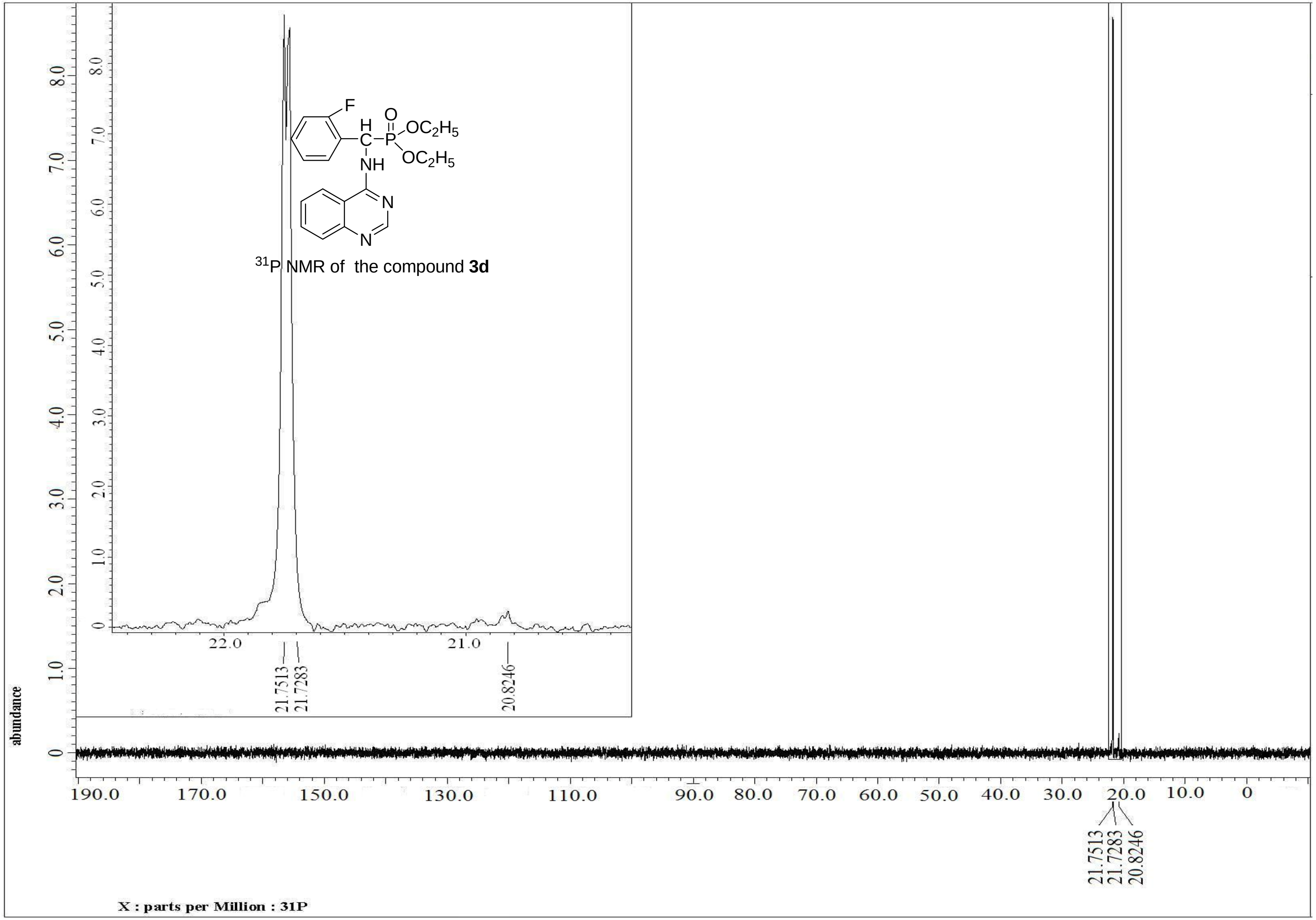
F
H
O
C
P
OC2H5
OC2H5
NH
N
N
31P NMR of the compound 3d
22.0
21.0
21.7513
21.7283
20.8246
abundance
190.0
170.0
150.0
130.0
110.0
90.0
80.0
70.0
60.0
50.0
40.0
30.0
20.0
10.0
0
X : parts per Million : 31P

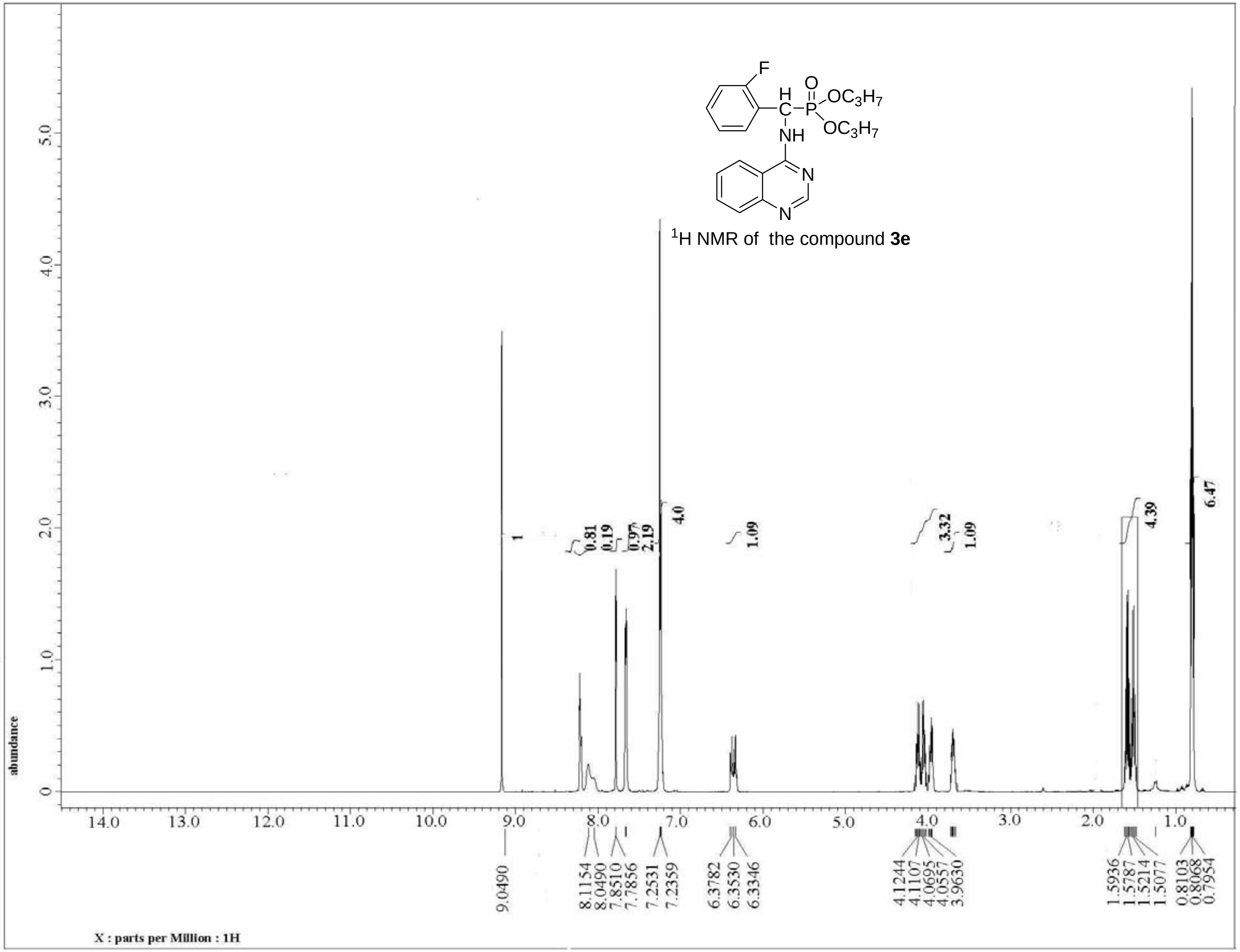

F
O
H
C
P
OC3H7
OC3H7
NH
N
N
1H NMR of the compound 3e
abundance
X : parts per Million : 1H
9.0490
8.1154
8.0490
7.8510
7.7856
7.2531
7.2359
6.3782
6.3530
6.3346
4.1244
4.1107
4.0695
4.0557
3.9630
1.5936
1.5787
1.5214
1.5077
0.8103
0.8068
0.7954
1
0.81
0.19
0.97
2.19
4.0
1.09
3.32
1.09
4.39
6.47

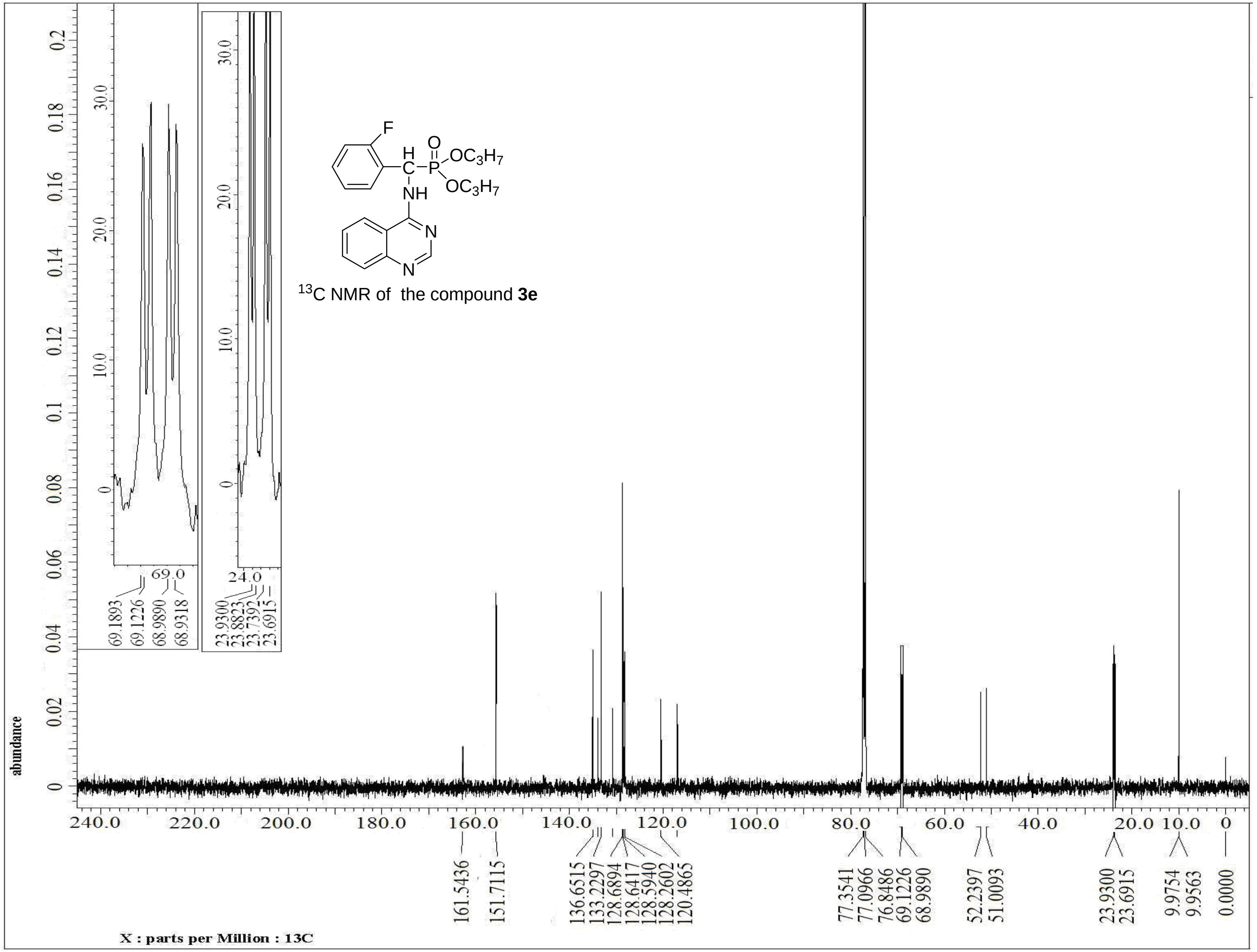

^{13}C NMR of the compound **3e**
abundance
X : parts per Million : 13C
161.5436
151.7115
136.6515
133.2297
128.6894
128.6417
128.5940
128.2602
120.4865
77.3541
77.0966
76.8486
69.1226
68.9890
52.2397
51.0093
23.9300
23.6915
9.9754
9.9563
0.0000
69.1893
69.1226
68.9890
68.9318
23.9300
23.8823
23.7392
23.6915

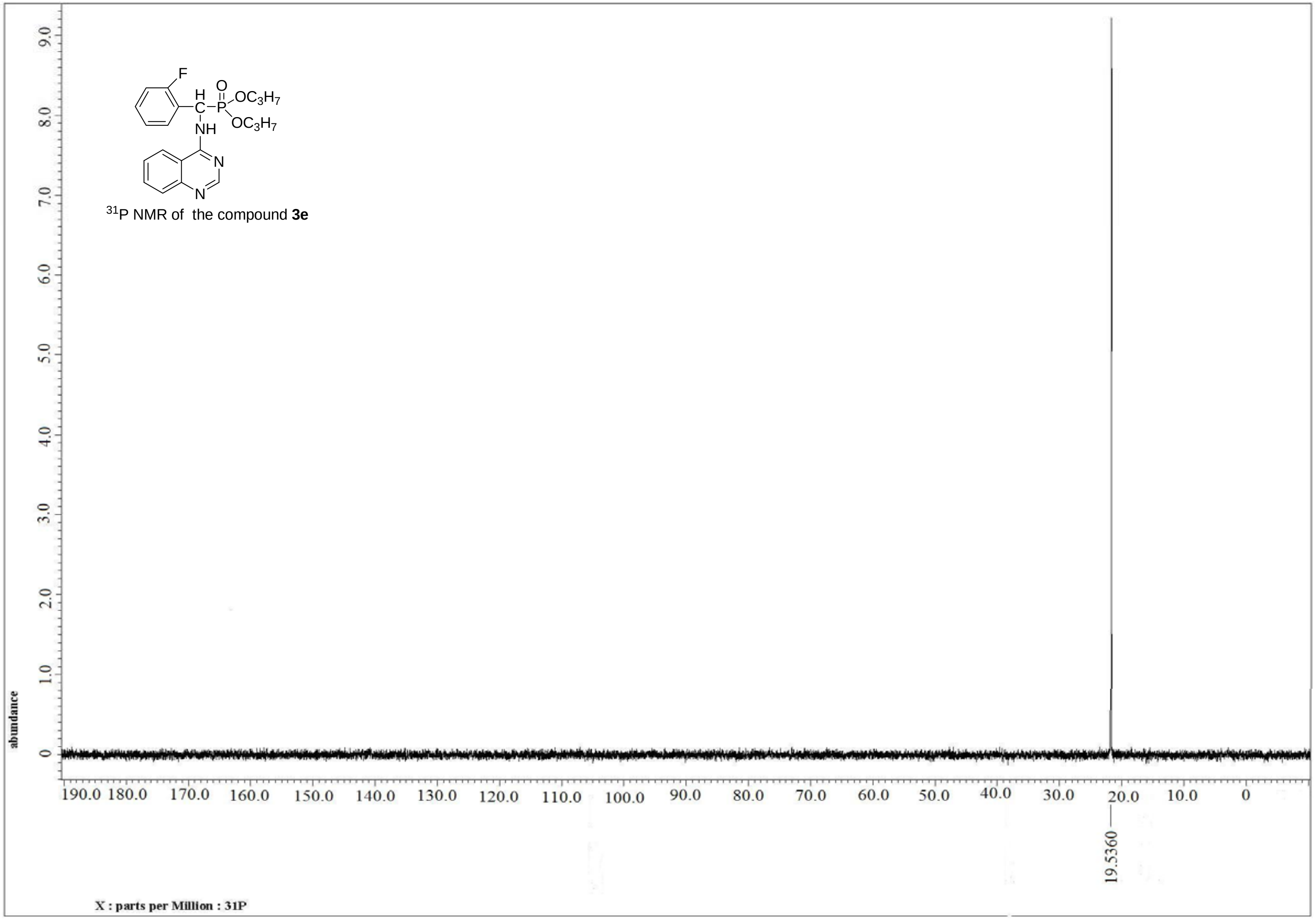

^{31}P NMR of the compound **3e**

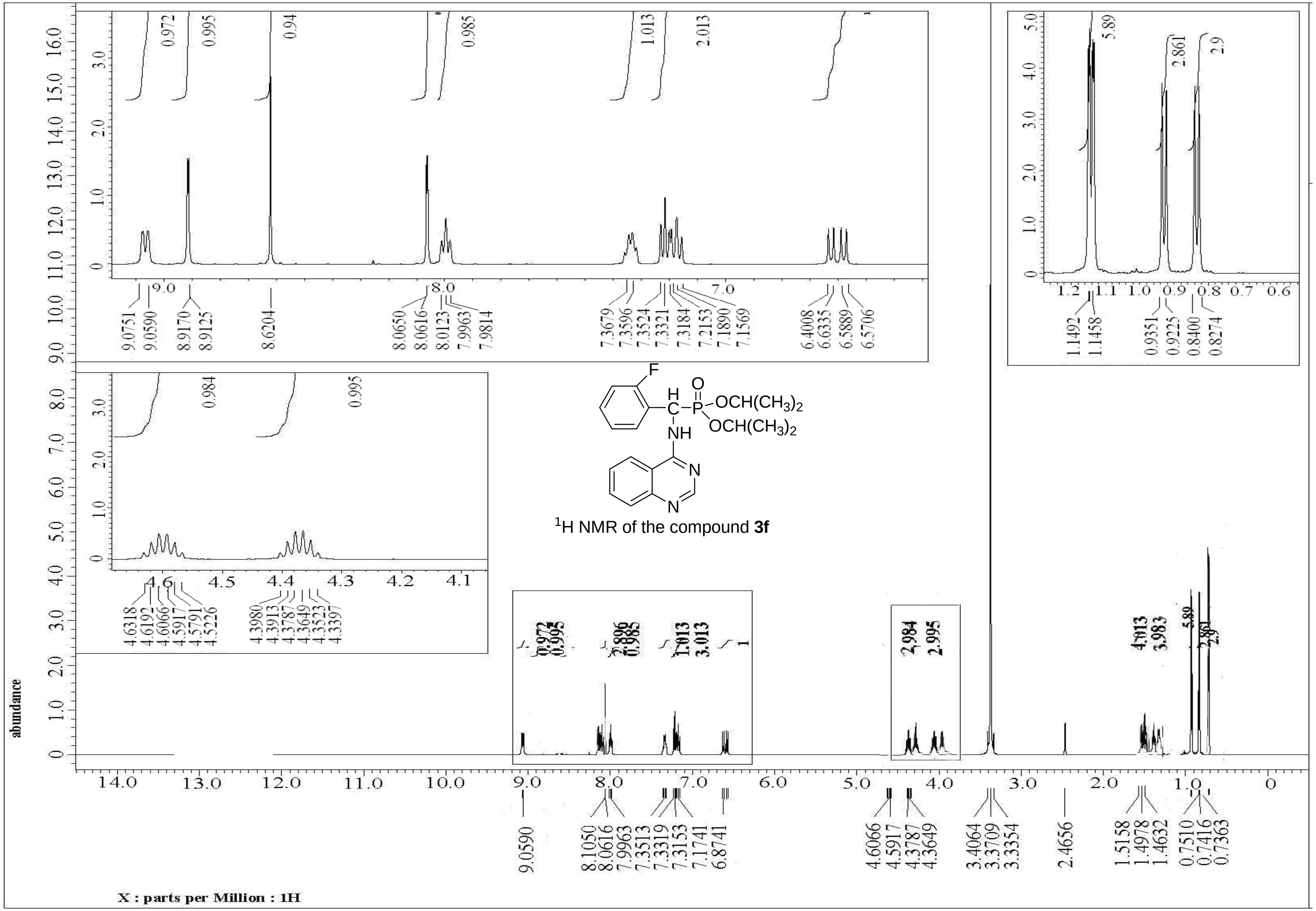

^{1}H NMR of the compound **3f**

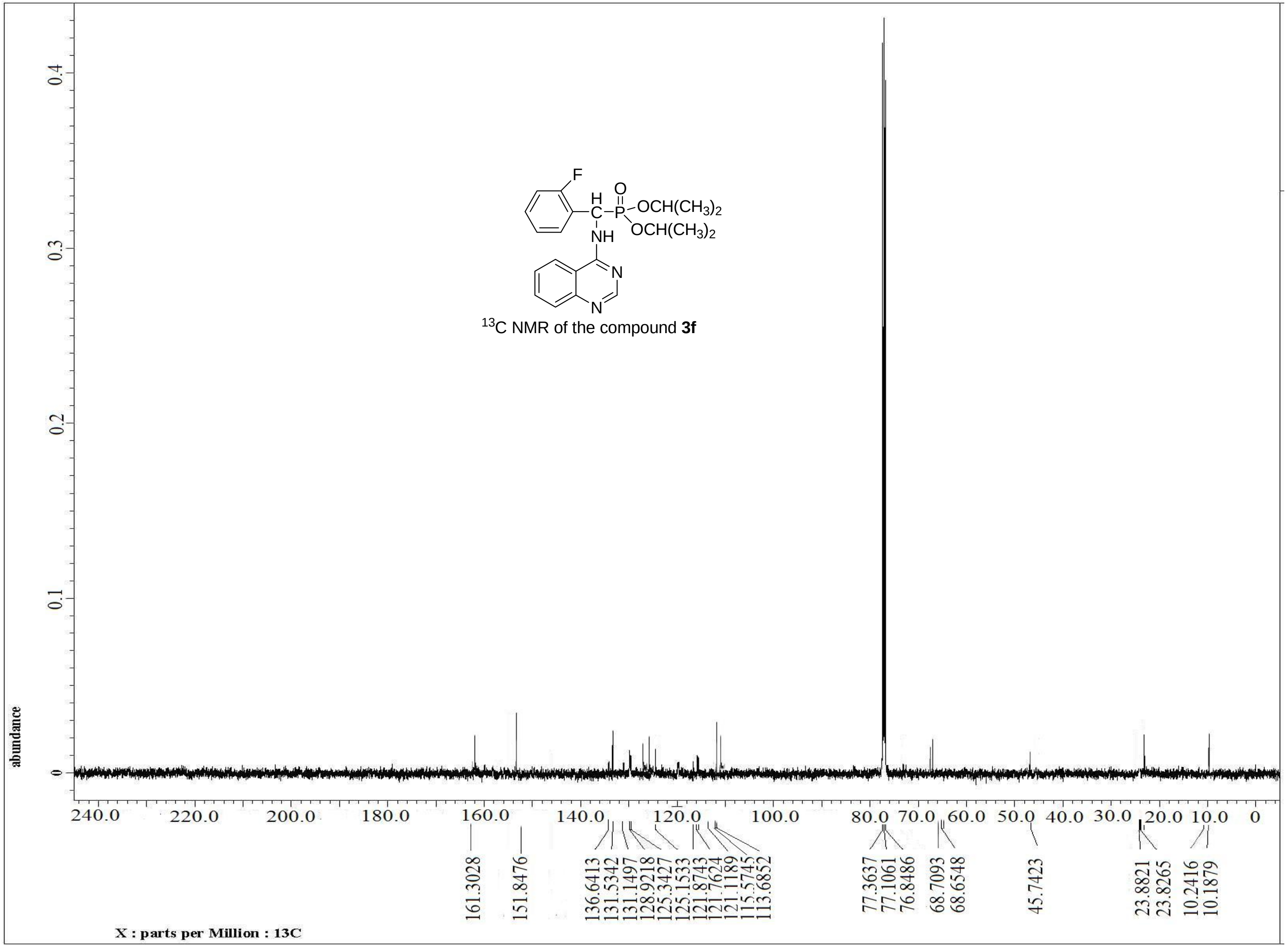
F
H
C
P
O
OCH(CH3)2
OCH(CH3)2
NH
N
N
13C NMR of the compound 3f
abundance
0.4
0.3
0.2
0.1
0
240.0
220.0
200.0
180.0
160.0
140.0
120.0
100.0
80.0
70.0
60.0
50.0
40.0
30.0
20.0
10.0
0
161.3028
151.8476
136.6413
131.5342
131.1497
128.9218
125.3427
125.1533
121.8743
121.7624
121.1189
115.5745
113.6852
77.3637
77.1061
76.8486
68.7093
68.6548
45.7423
23.8821
23.8265
10.2416
10.1879
X : parts per Million : 13C

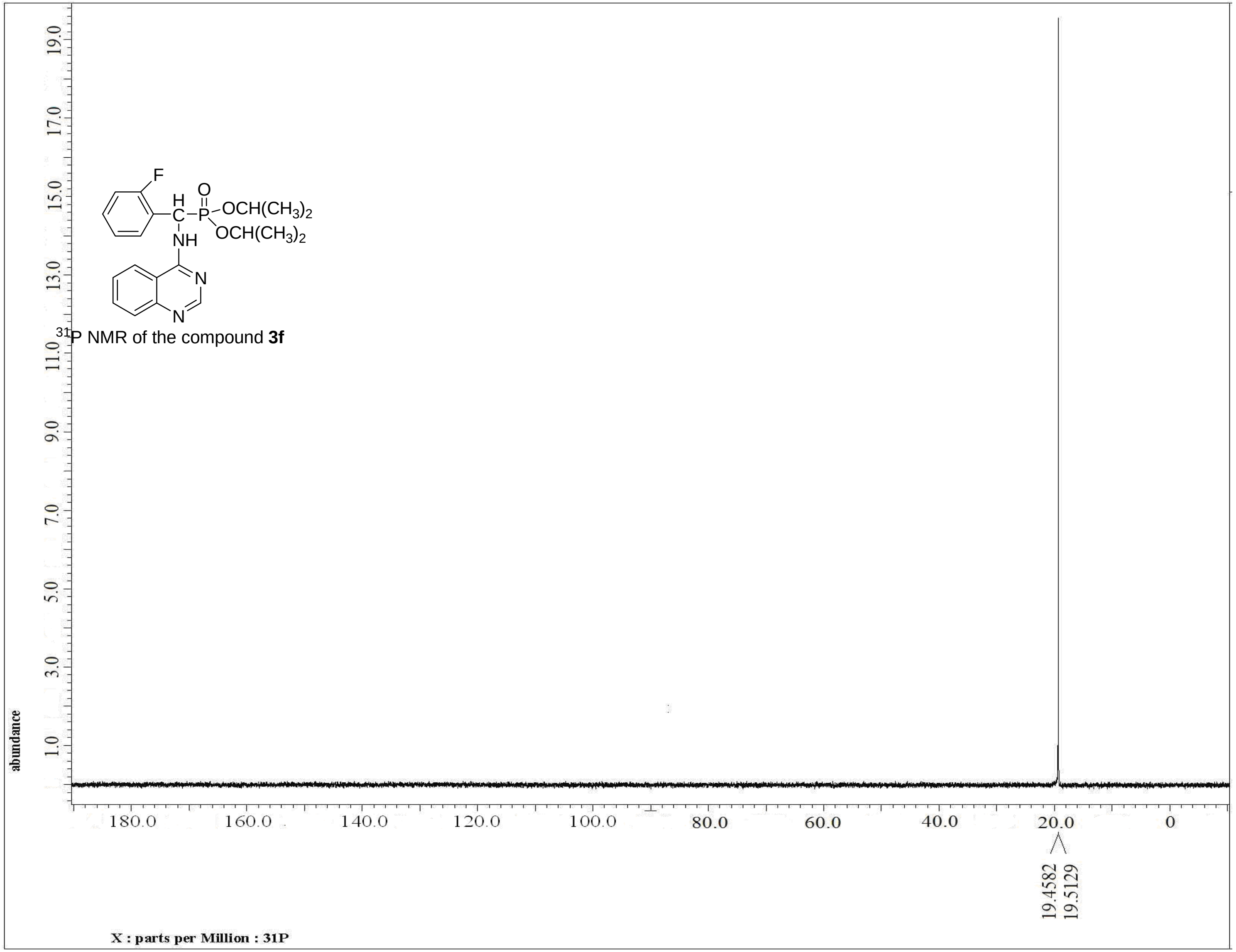
F
O
H
C
P
OCH(CH3)2
OCH(CH3)2
NH
N
N
31P NMR of the compound 3f
abundance
19.0
17.0
15.0
13.0
11.0
9.0
7.0
5.0
3.0
1.0
180.0
160.0
140.0
120.0
100.0
80.0
60.0
40.0
20.0
0
19.4582
19.5129
X : parts per Million : 31P

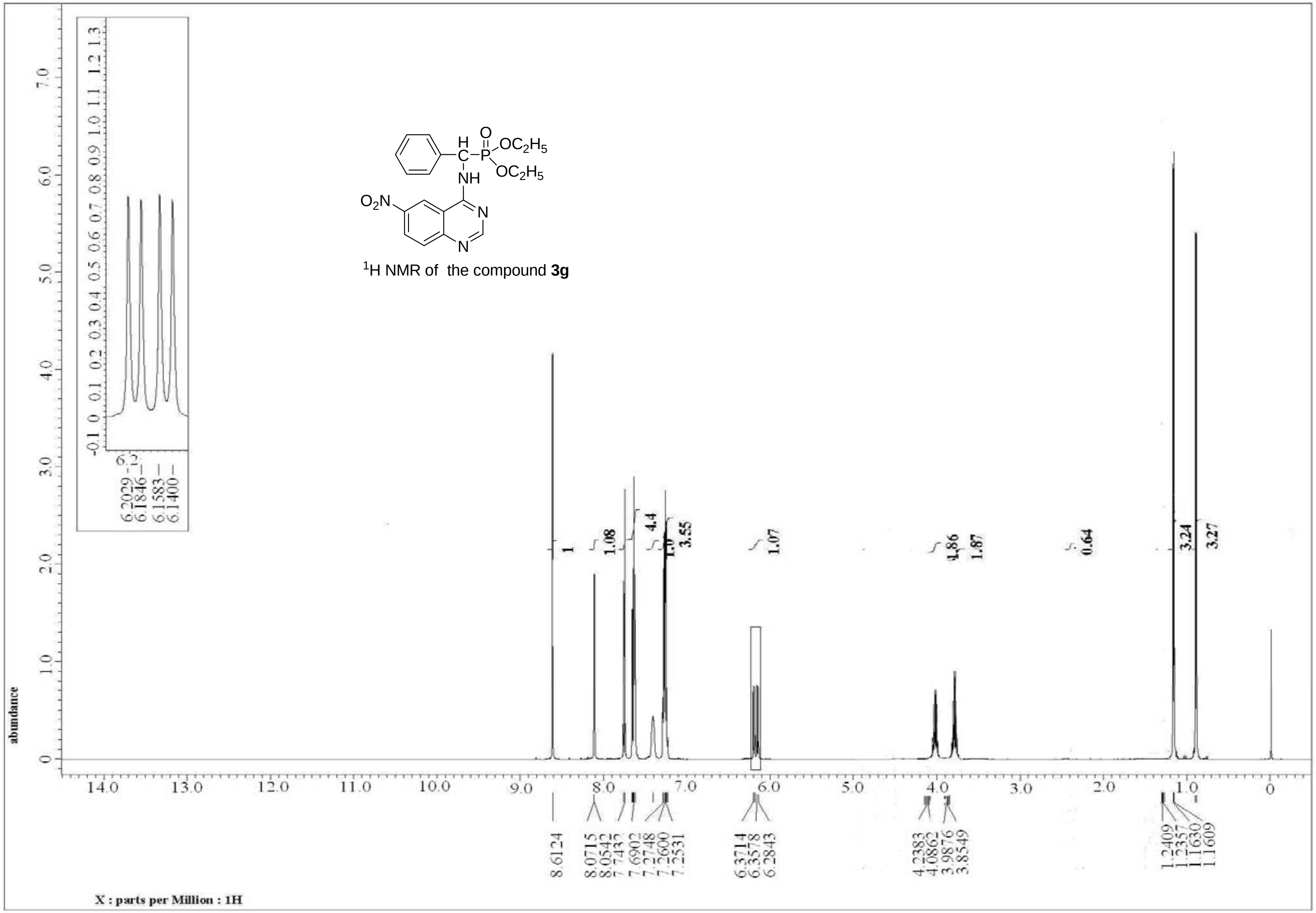

^{1}H NMR of the compound **3g**
abundance
X : parts per Million : 1H

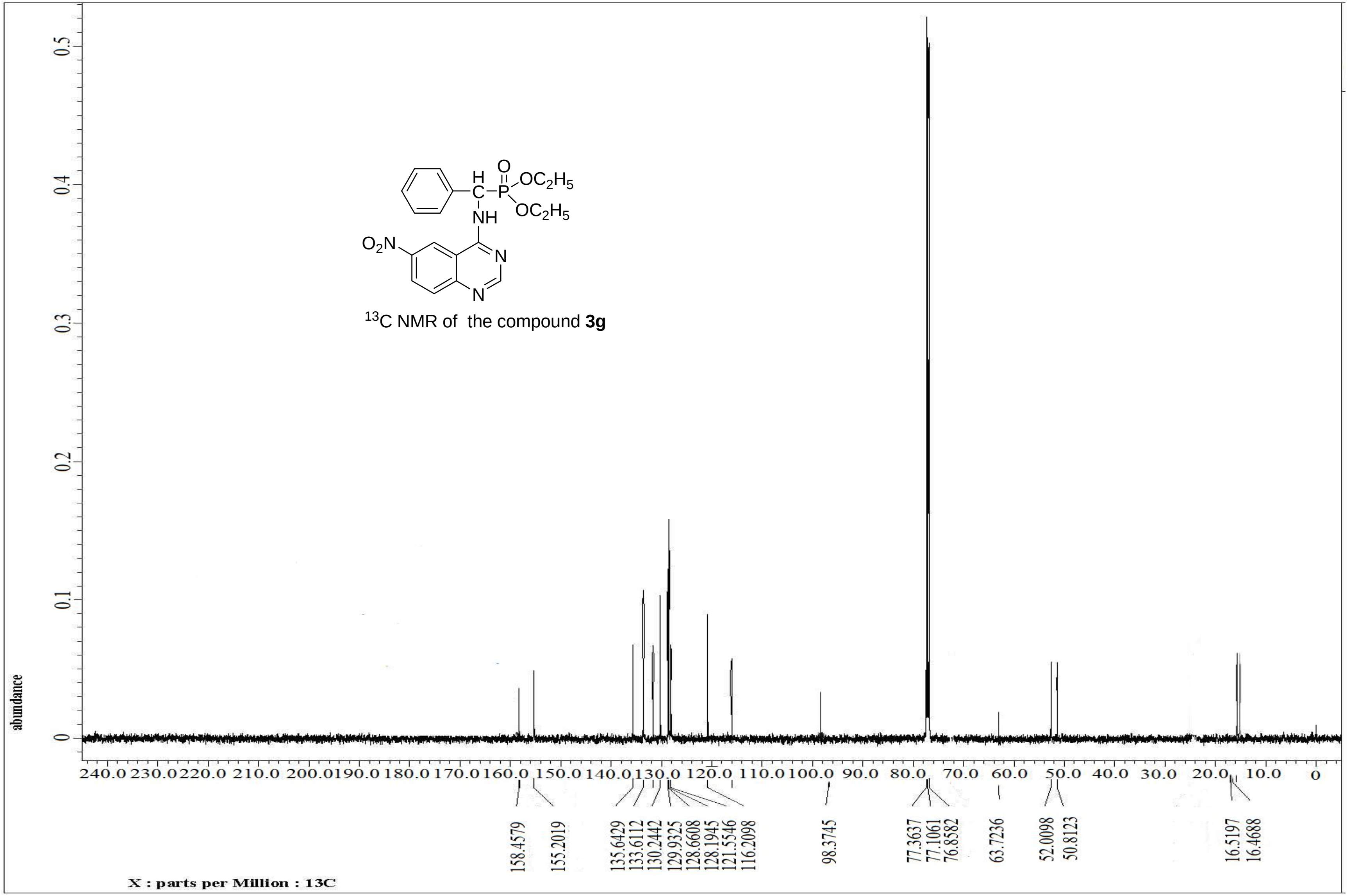

O
H
C
P
OC2H5
OC2H5
NH
O2N
N
N
13C NMR of the compound 3g
abundance
0.5
0.4
0.3
0.2
0.1
0
240.0 230.0 220.0 210.0 200.0 190.0 180.0 170.0 160.0 150.0 140.0 130.0 120.0 110.0 100.0 90.0 80.0 70.0 60.0 50.0 40.0 30.0 20.0 10.0 0
158.4579
155.2019
135.6429
133.6112
130.2442
129.9325
128.6608
128.1945
121.5546
116.2098
98.3745
77.3637
77.1061
76.8582
63.7236
52.0098
50.8123
16.5197
16.4688
X : parts per Million : 13C

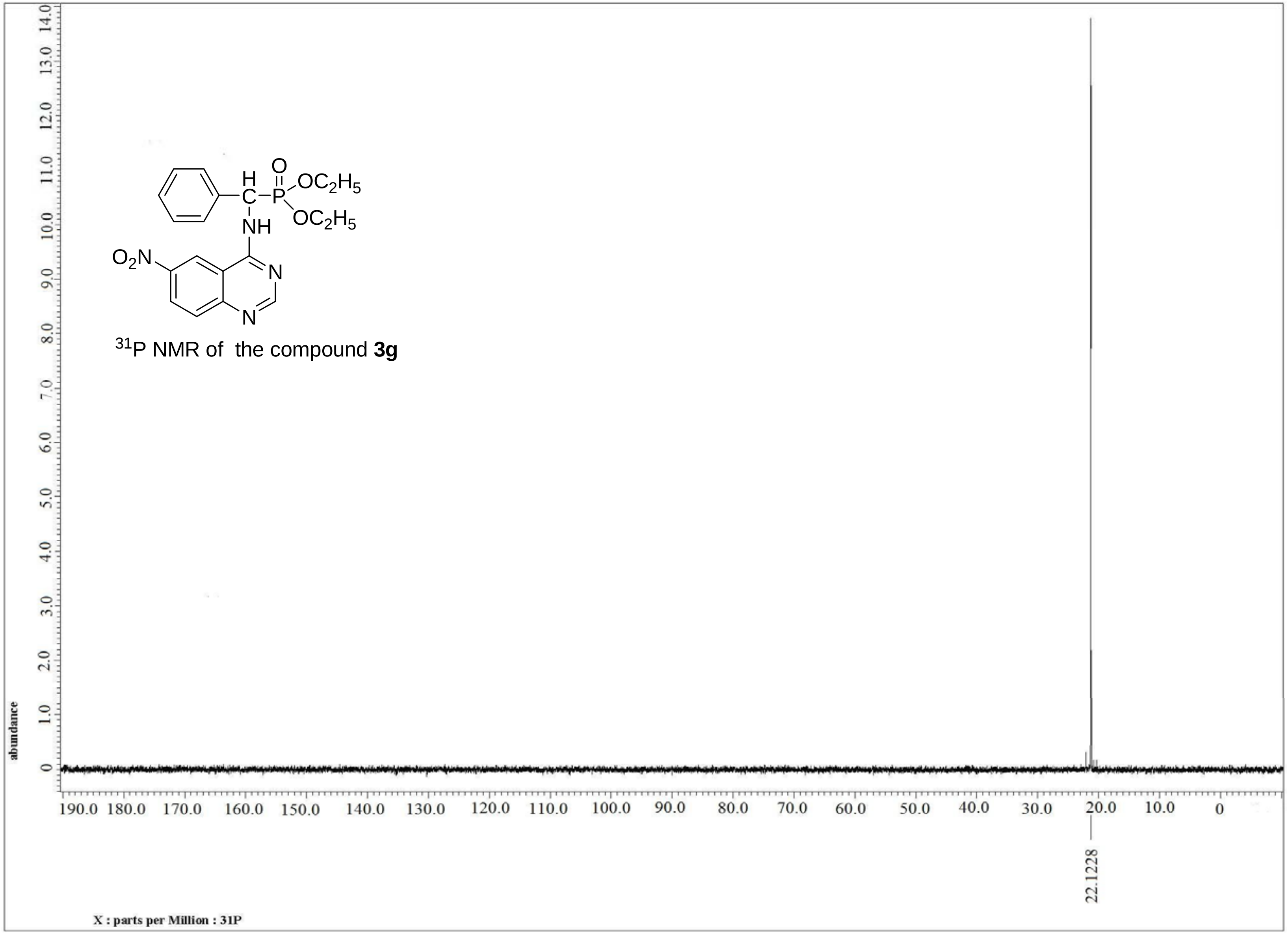

O
H
OC2H5
C
P
NH
OC2H5
O2N
N
N
31P NMR of the compound 3g
abundance
14.0
13.0
12.0
11.0
10.0
9.0
8.0
7.0
6.0
5.0
4.0
3.0
2.0
1.0
0
190.0 180.0 170.0 160.0 150.0 140.0 130.0 120.0 110.0 100.0 90.0 80.0 70.0 60.0 50.0 40.0 30.0 20.0 10.0 0
22.1228
X : parts per Million : 31P

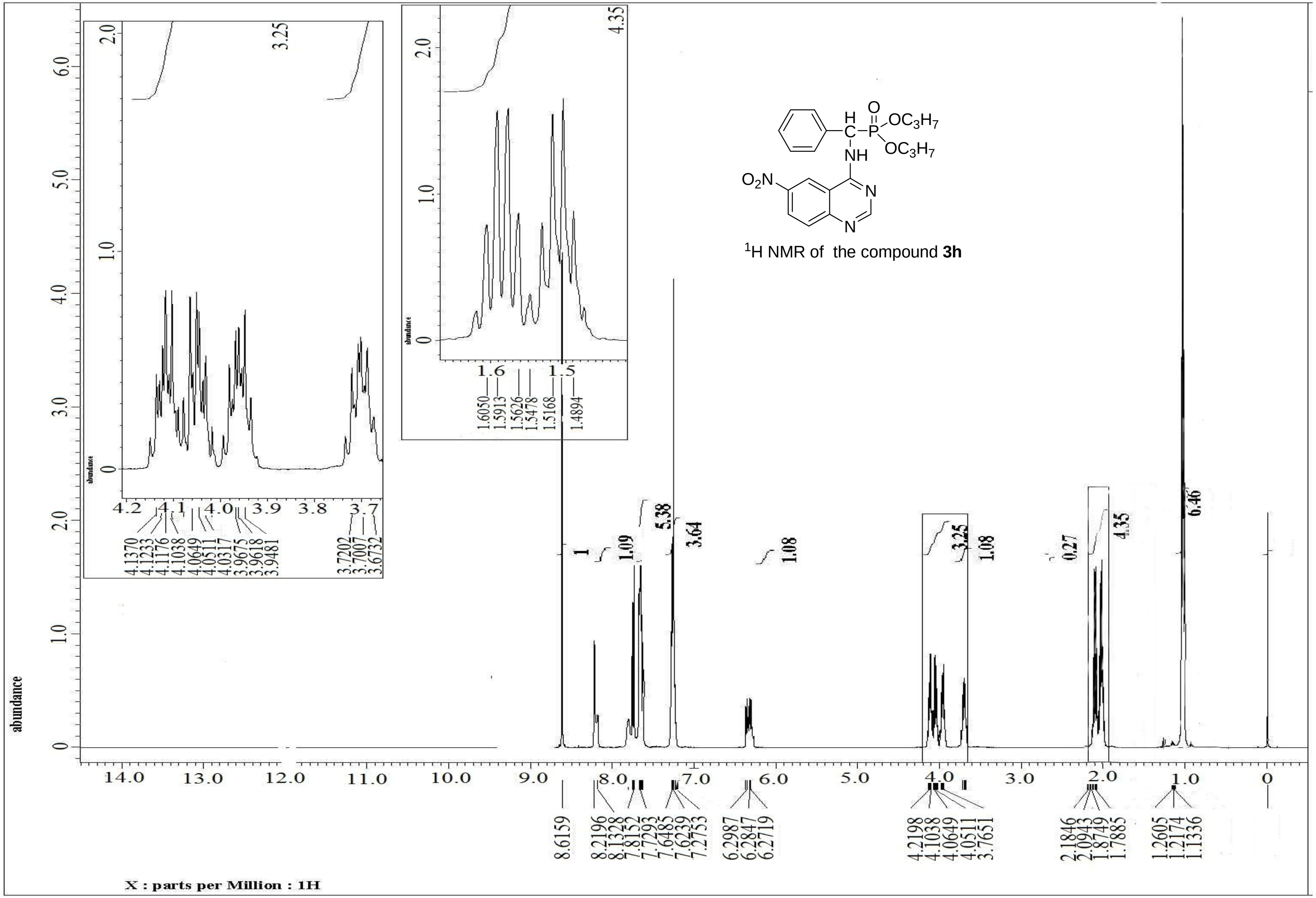

^{1}H NMR of the compound **3h**
X : parts per Million : 1H
abundance

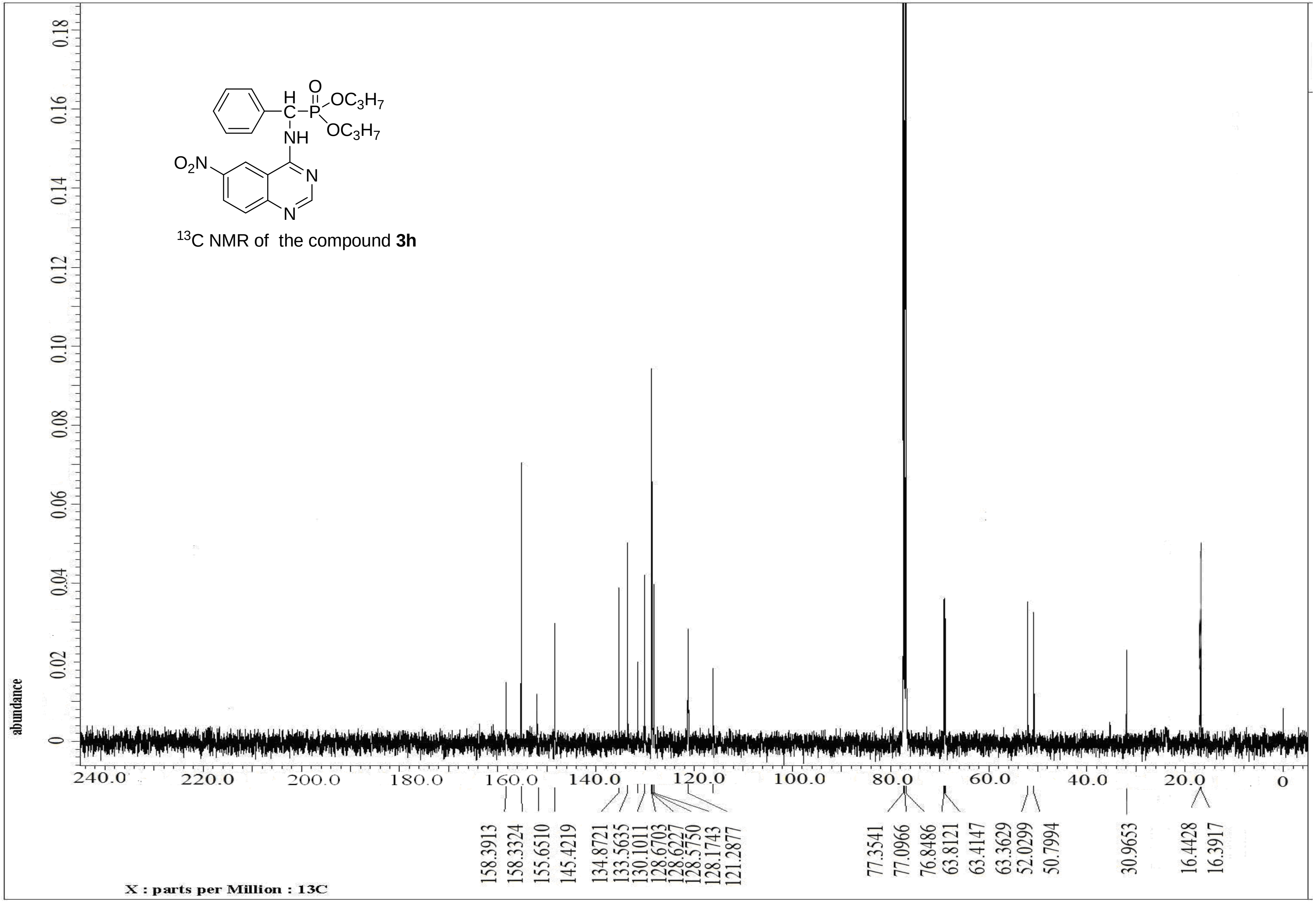

O
H
C
P
OC3H7
OC3H7
NH
O2N
N
N
13C NMR of the compound 3h
abundance
0.18
0.16
0.14
0.12
0.10
0.08
0.06
0.04
0.02
0
240.0
220.0
200.0
180.0
160.0
140.0
120.0
100.0
80.0
60.0
40.0
20.0
0
158.3913
158.3324
155.6510
145.4219
134.8721
133.5635
130.1011
128.6703
128.6227
128.5750
128.1743
121.2877
77.3541
77.0966
76.8486
63.8121
63.4147
63.3629
52.0299
50.7994
30.9653
16.4428
16.3917
X : parts per Million : 13C

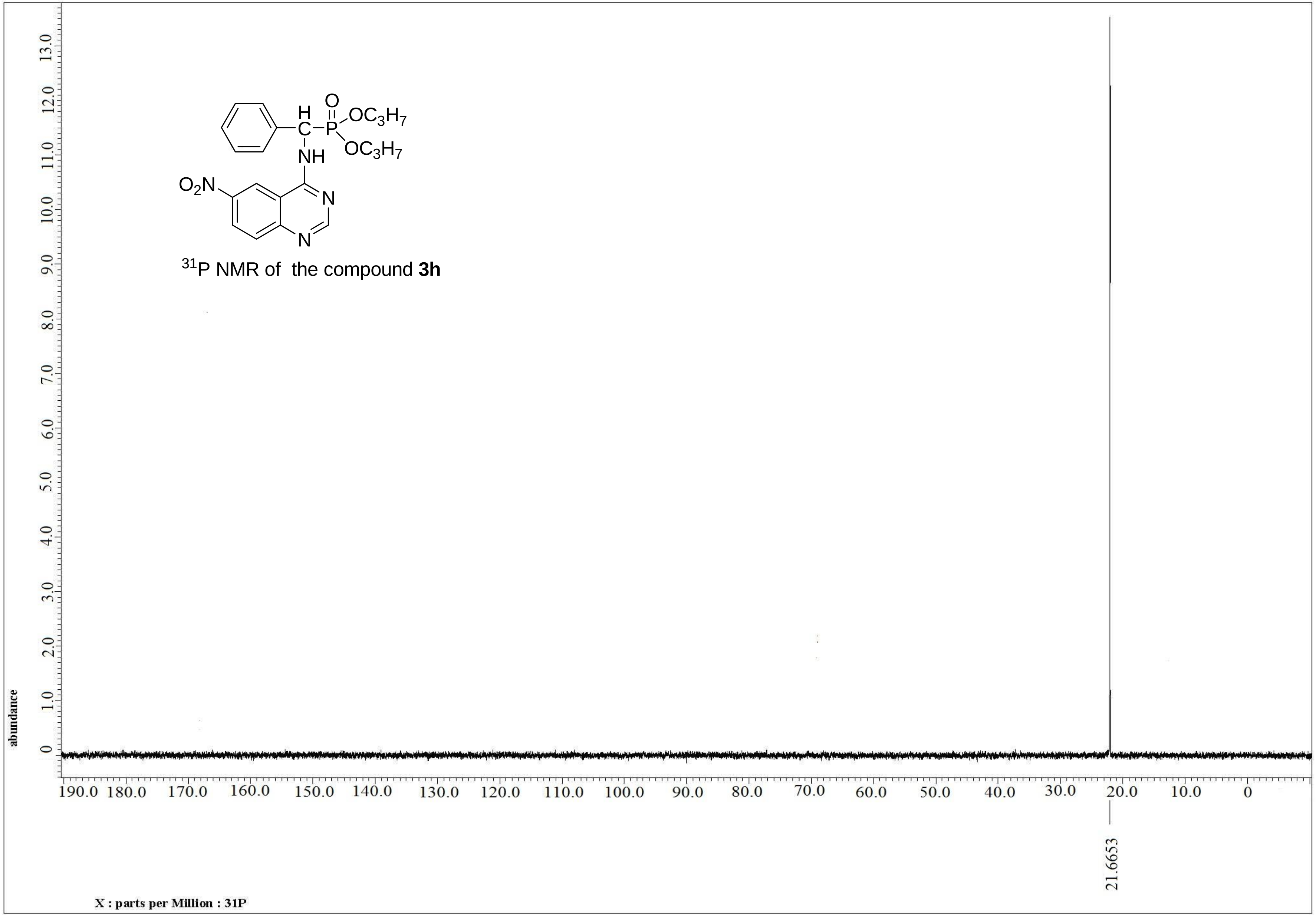

^{31}P NMR of the compound **3h**

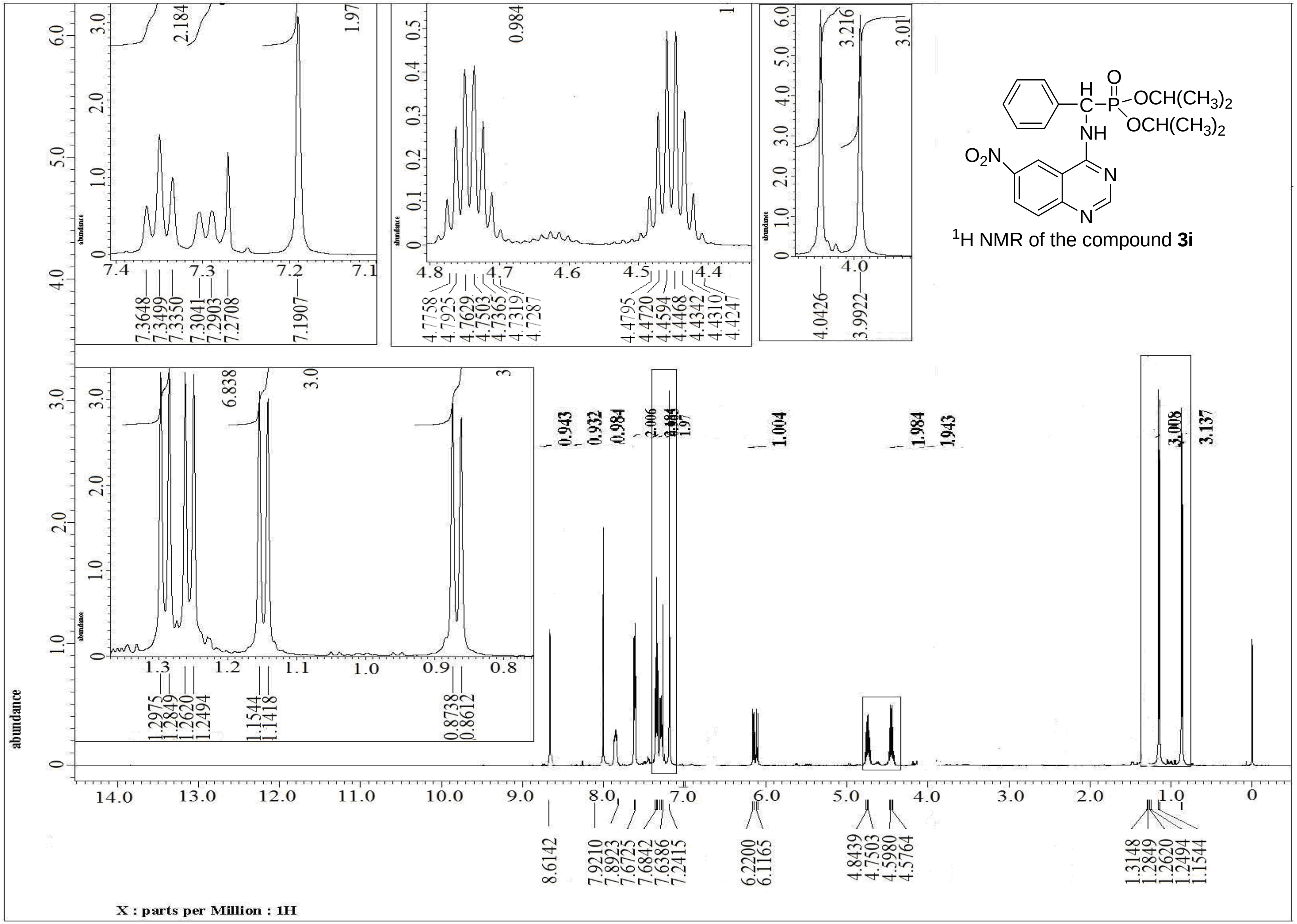

^{1}H NMR of the compound **3i**

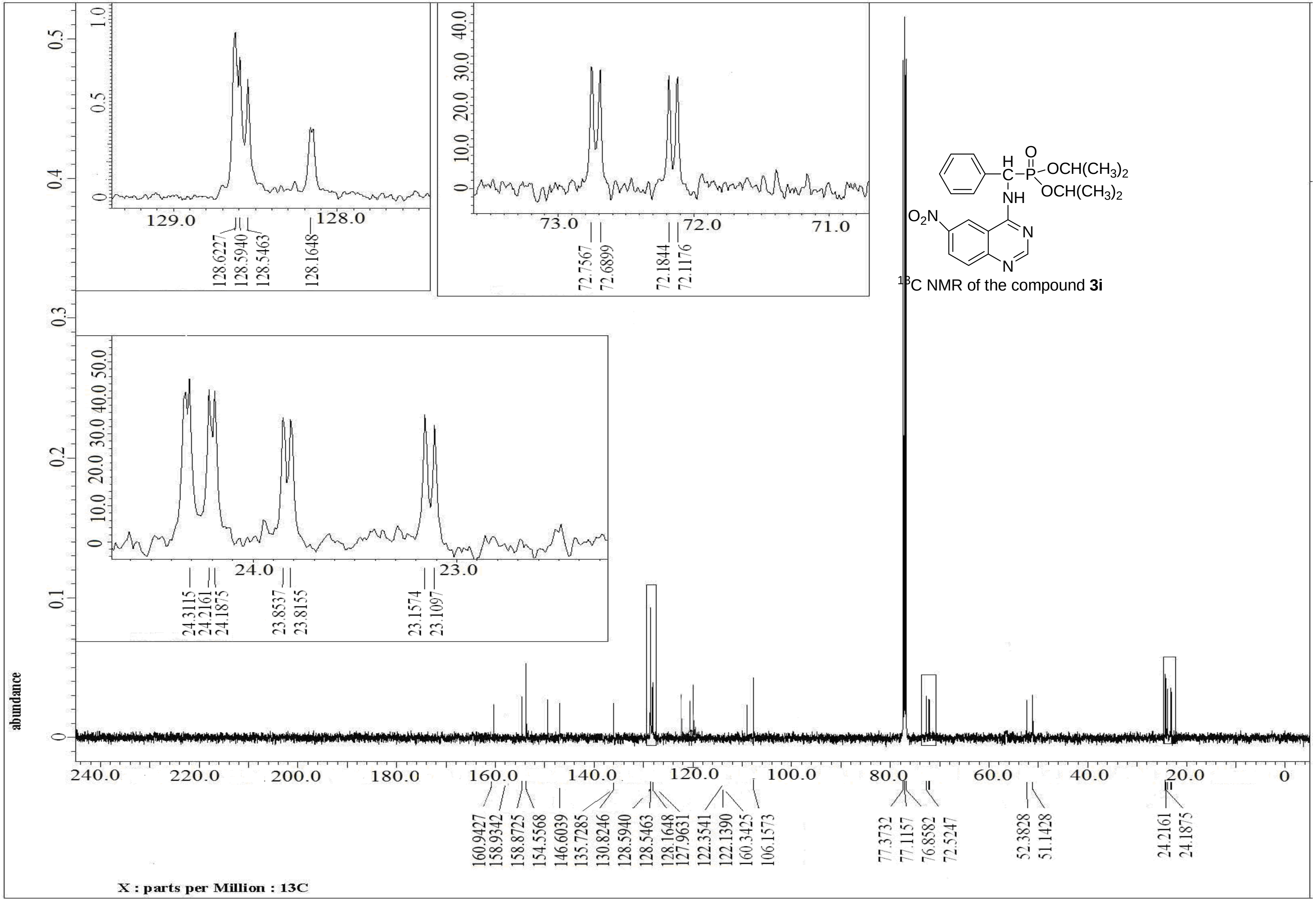

^{13}C NMR of the compound **3i**

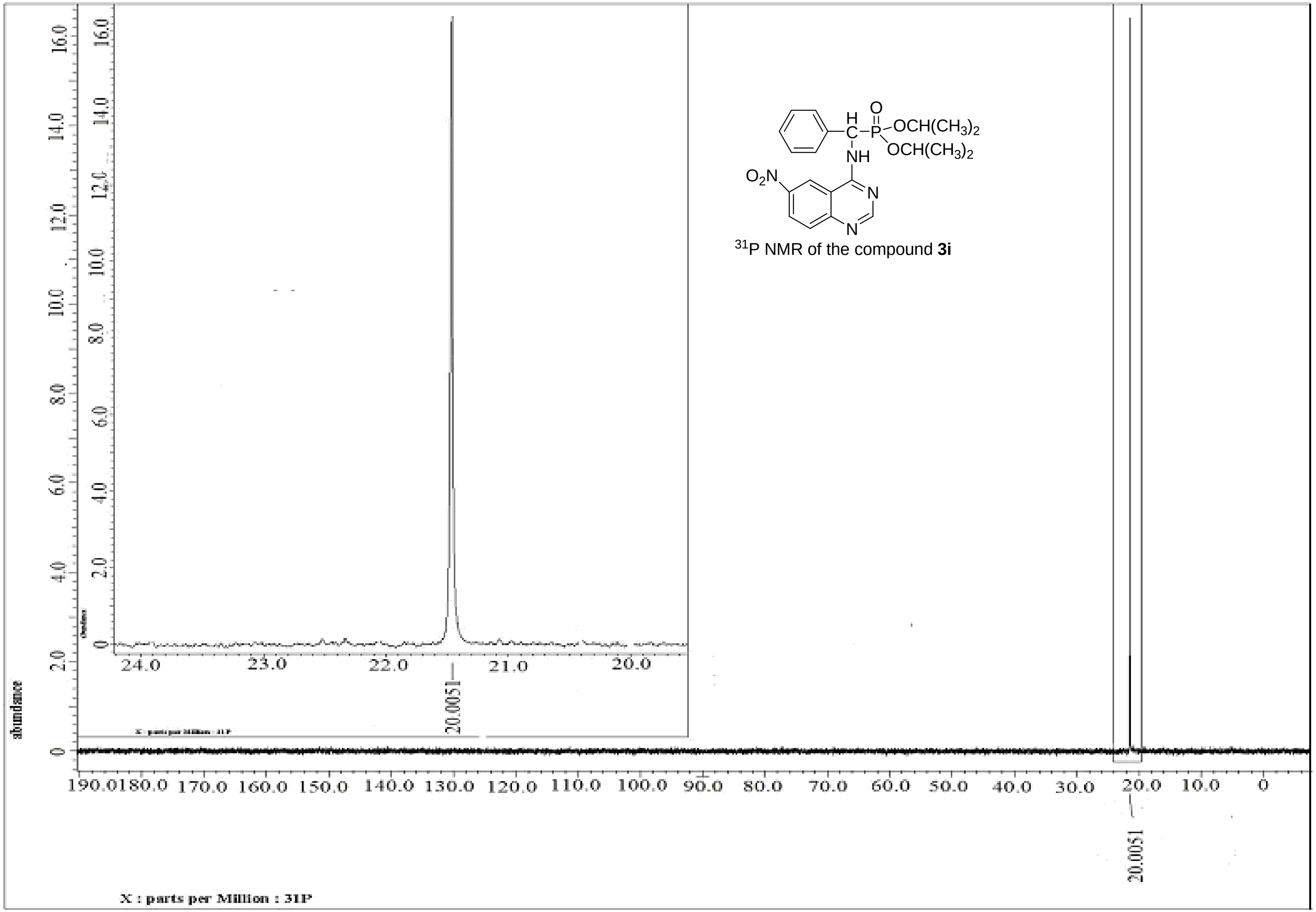

O
H
C
P
OCH(CH3)2
OCH(CH3)2
NH
O2N
N
N
31P NMR of the compound 3i
abundance
X : parts per Million : 31P
20.0051
190.0
180.0
170.0
160.0
150.0
140.0
130.0
120.0
110.0
100.0
90.0
80.0
70.0
60.0
50.0
40.0
30.0
20.0
10.0
0
24.0
23.0
22.0
21.0
20.0
2.0
4.0
6.0
8.0
10.0
12.0
14.0
16.0

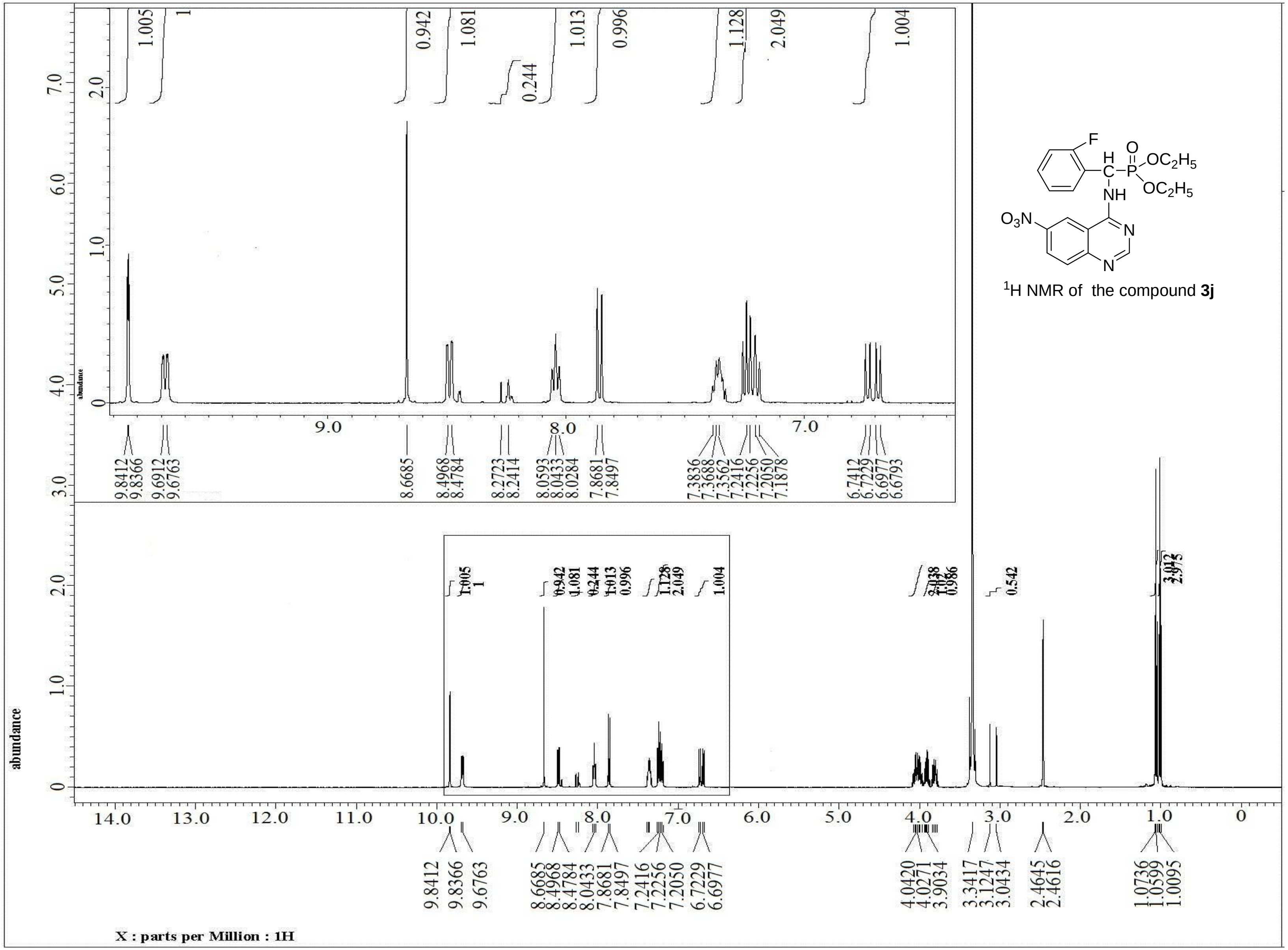

^{1}H NMR of the compound **3j**
X : parts per Million : 1H
abundance

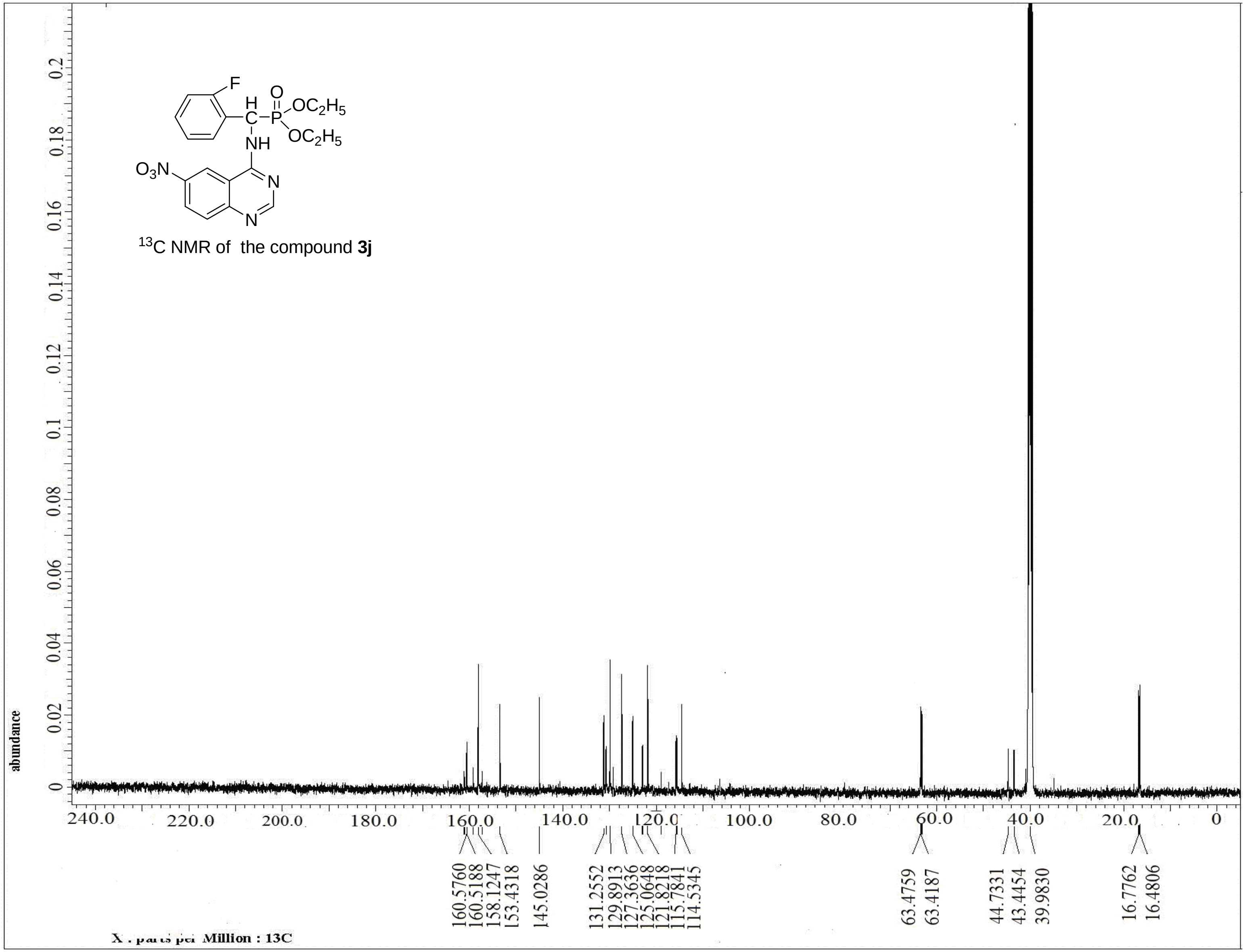

F
H
O
C
P
OC_2H_5
OC_2H_5
NH
O_3N
N
N
^{13}C NMR of the compound **3j**
abundance
0.2
0.18
0.16
0.14
0.12
0.1
0.08
0.06
0.04
0.02
0
240.0
220.0
200.0
180.0
160.0
140.0
120.0
100.0
80.0
60.0
40.0
20.0
0
160.5760
160.5188
158.1247
153.4318
145.0286
131.2552
129.8913
127.3636
125.0648
121.8218
115.7841
114.5345
63.4759
63.4187
44.7331
43.4454
39.9830
16.7762
16.4806
X : parts per Million : 13C

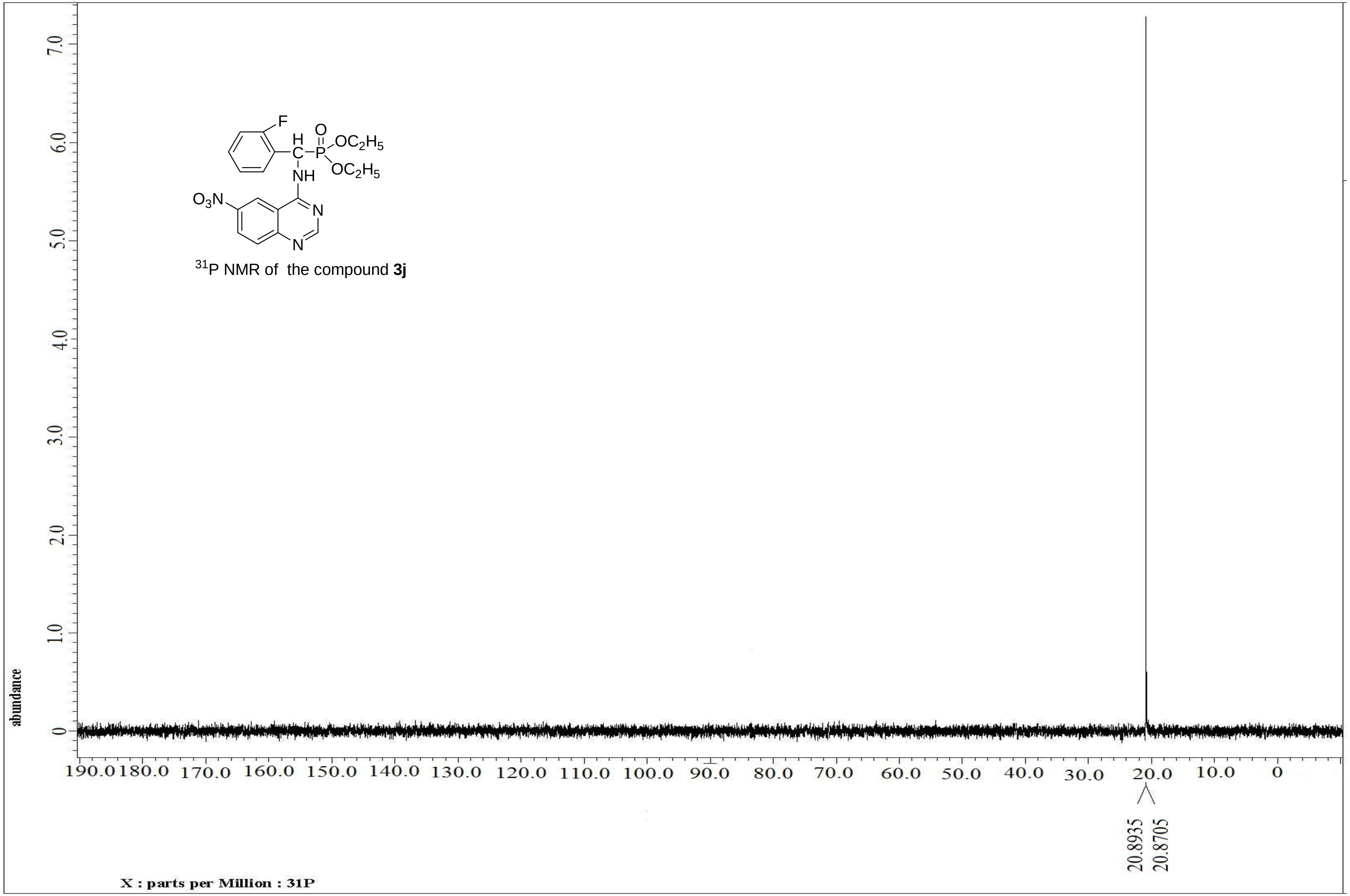

^{31}P NMR of the compound **3j**

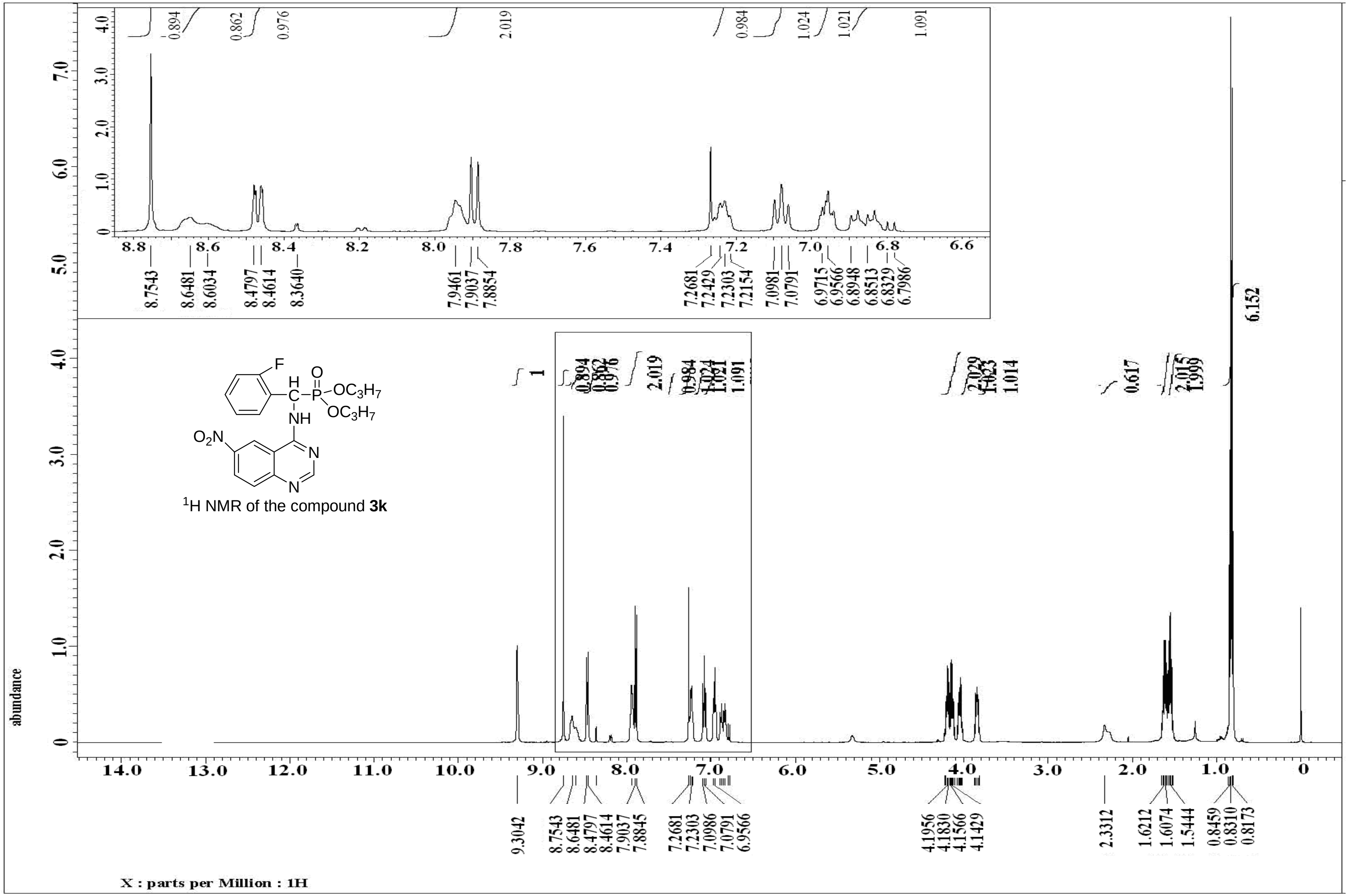

^{1}H NMR of the compound **3k**

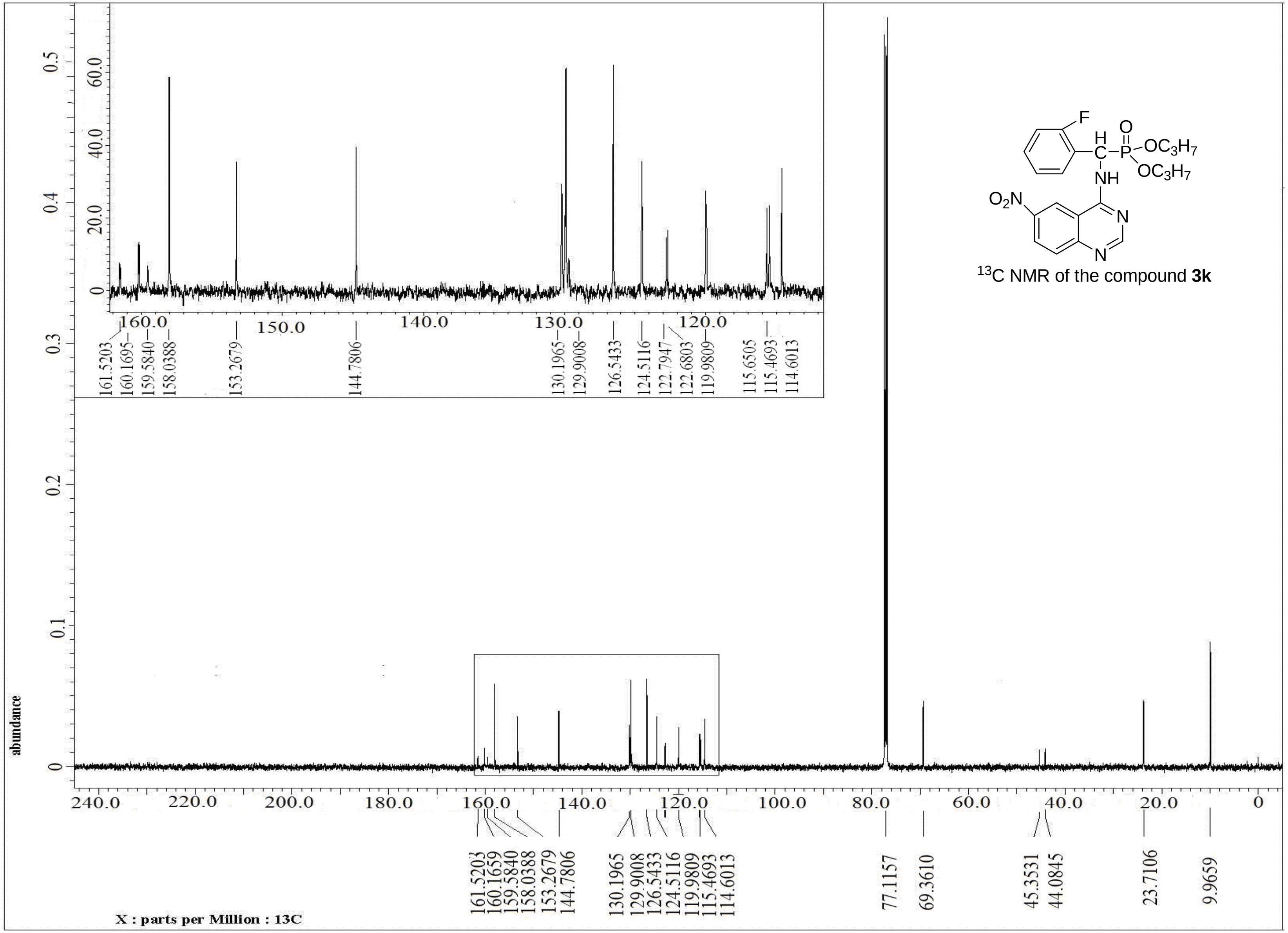
^{13}C NMR of the compound **3k**
F
O
H
C
P
OC_3H_7
OC_3H_7
NH
O_2N
N
N
abundance
X : parts per Million : 13C
161.5203
160.1659
159.5840
158.0388
153.2679
144.7806
130.1965
129.9008
126.5433
124.5116
119.9809
115.4693
114.6013
77.1157
69.3610
45.3531
44.0845
23.7106
9.9659
161.5203
160.1695
159.5840
158.0388
153.2679
144.7806
130.1965
129.9008
126.5433
124.5116
122.7947
122.6803
119.9809
115.6505
115.4693
114.6013

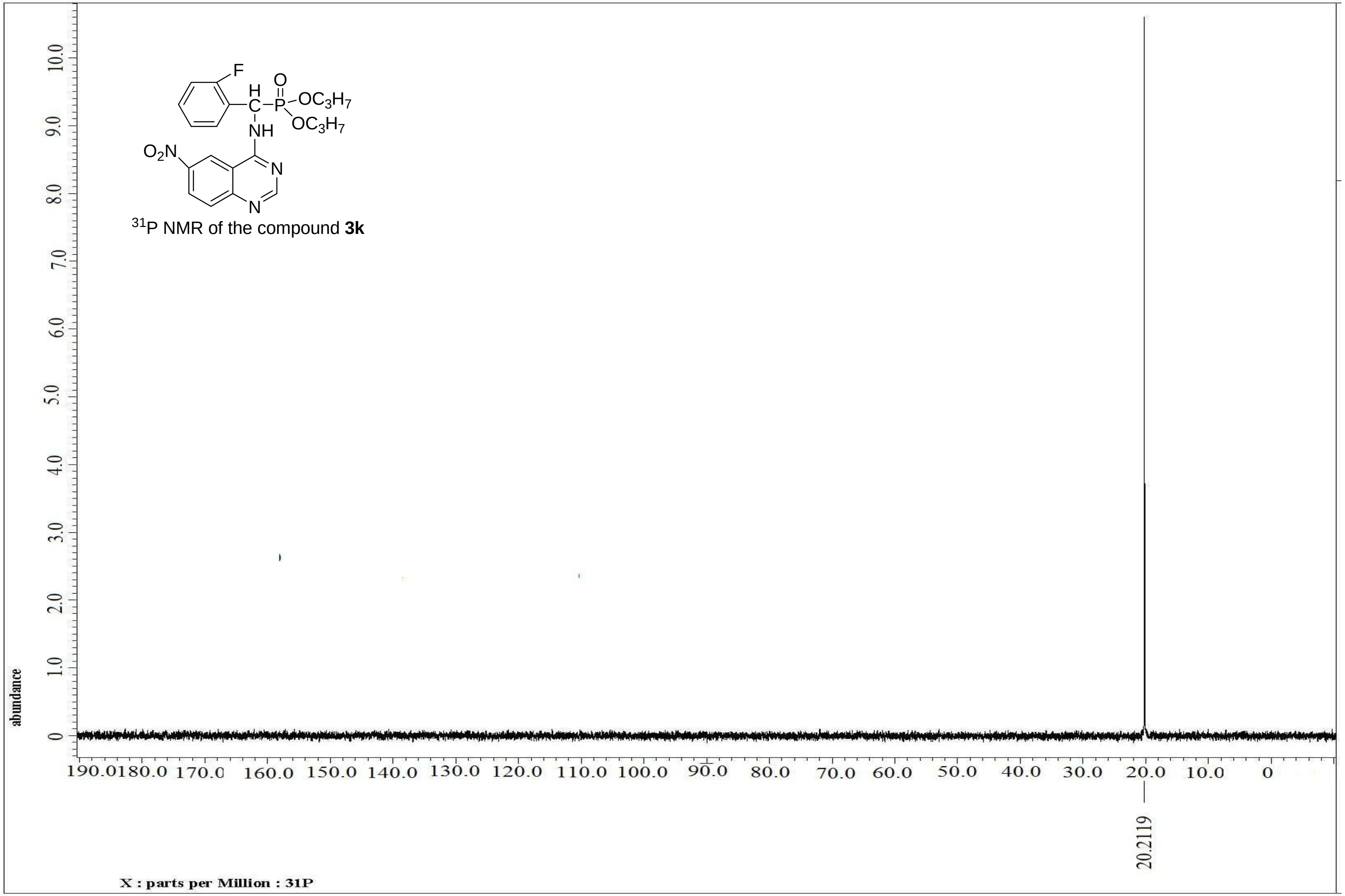

F
H
O
C
P
OC_3H_7
OC_3H_7
NH
O_2N
N
N
^{31}P NMR of the compound **3k**
abundance
0
1.0
2.0
3.0
4.0
5.0
6.0
7.0
8.0
9.0
10.0
190.0
180.0
170.0
160.0
150.0
140.0
130.0
120.0
110.0
100.0
90.0
80.0
70.0
60.0
50.0
40.0
30.0
20.0
10.0
0
20.2119
X : parts per Million : 31P

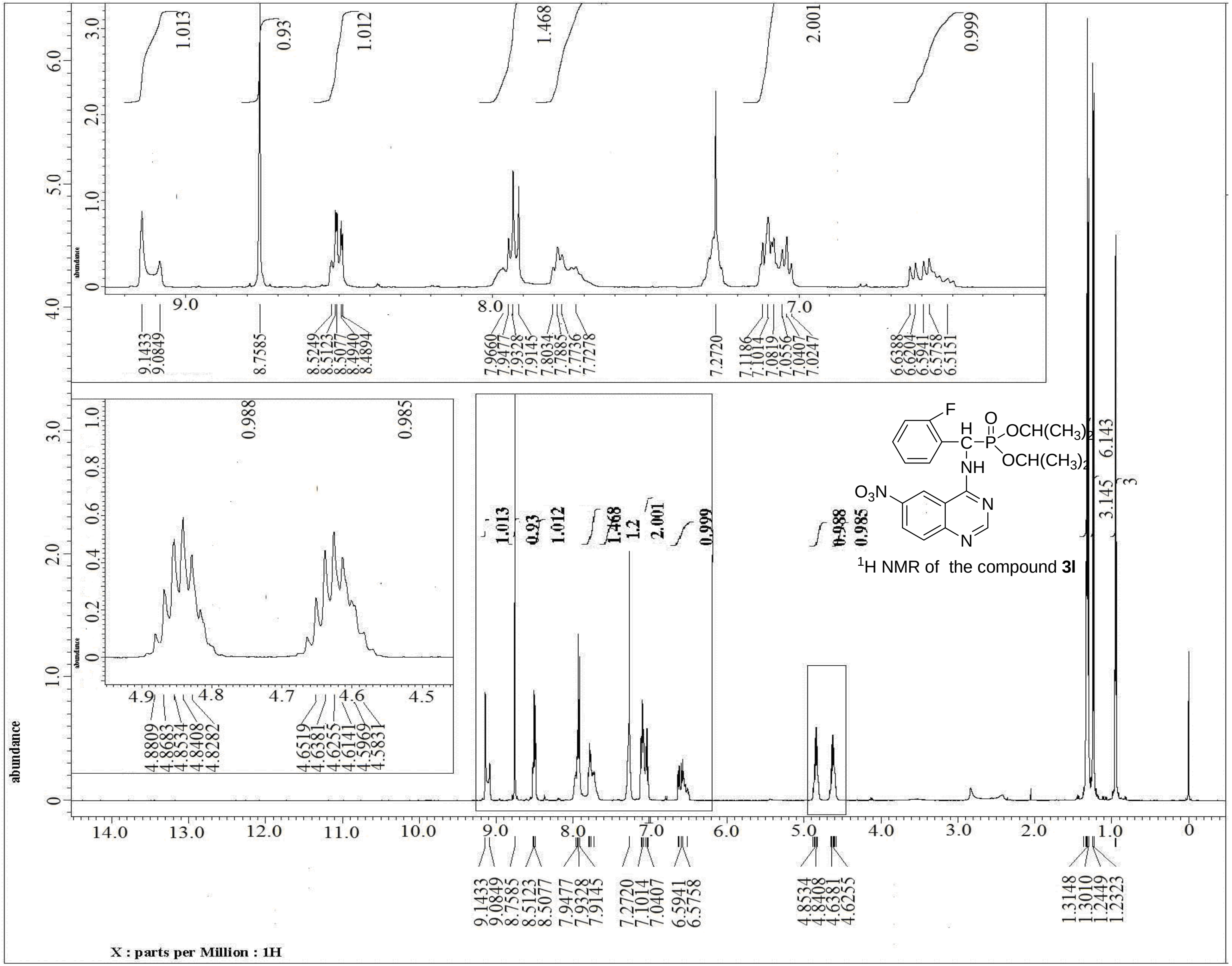

^{1}H NMR of the compound **3l**

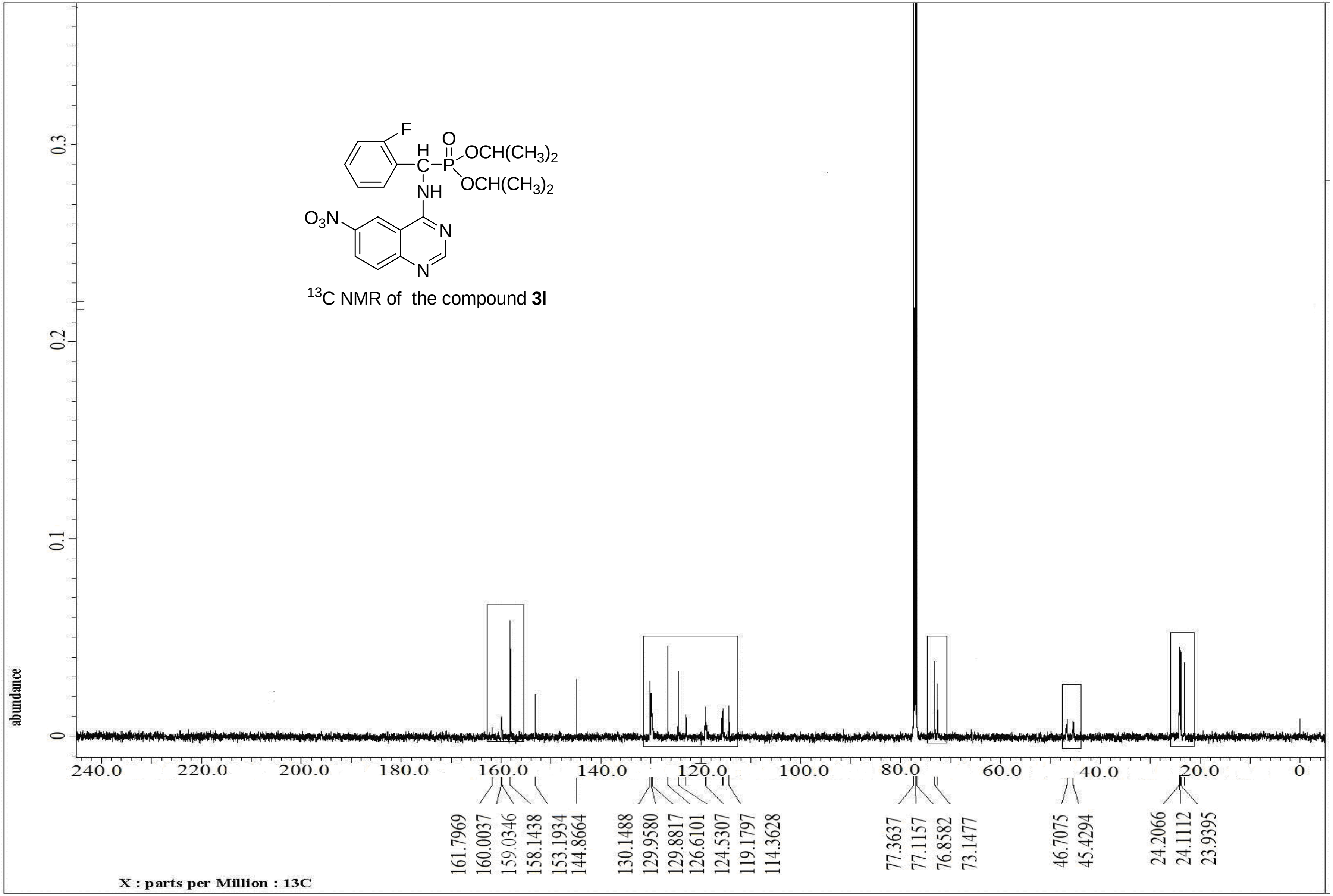
F
O
H
OCH(CH3)2
C
P
OCH(CH3)2
NH
O3N
N
N
13C NMR of the compound 3l
abundance
0.3
0.2
0.1
0
240.0
220.0
200.0
180.0
160.0
140.0
120.0
100.0
80.0
60.0
40.0
20.0
0
161.7969
160.0037
159.0346
158.1438
153.1934
144.8664
130.1488
129.9580
129.8817
126.6101
124.5307
119.1797
114.3628
77.3637
77.1157
76.8582
73.1477
46.7075
45.4294
24.2066
24.1112
23.9395
X : parts per Million : 13C

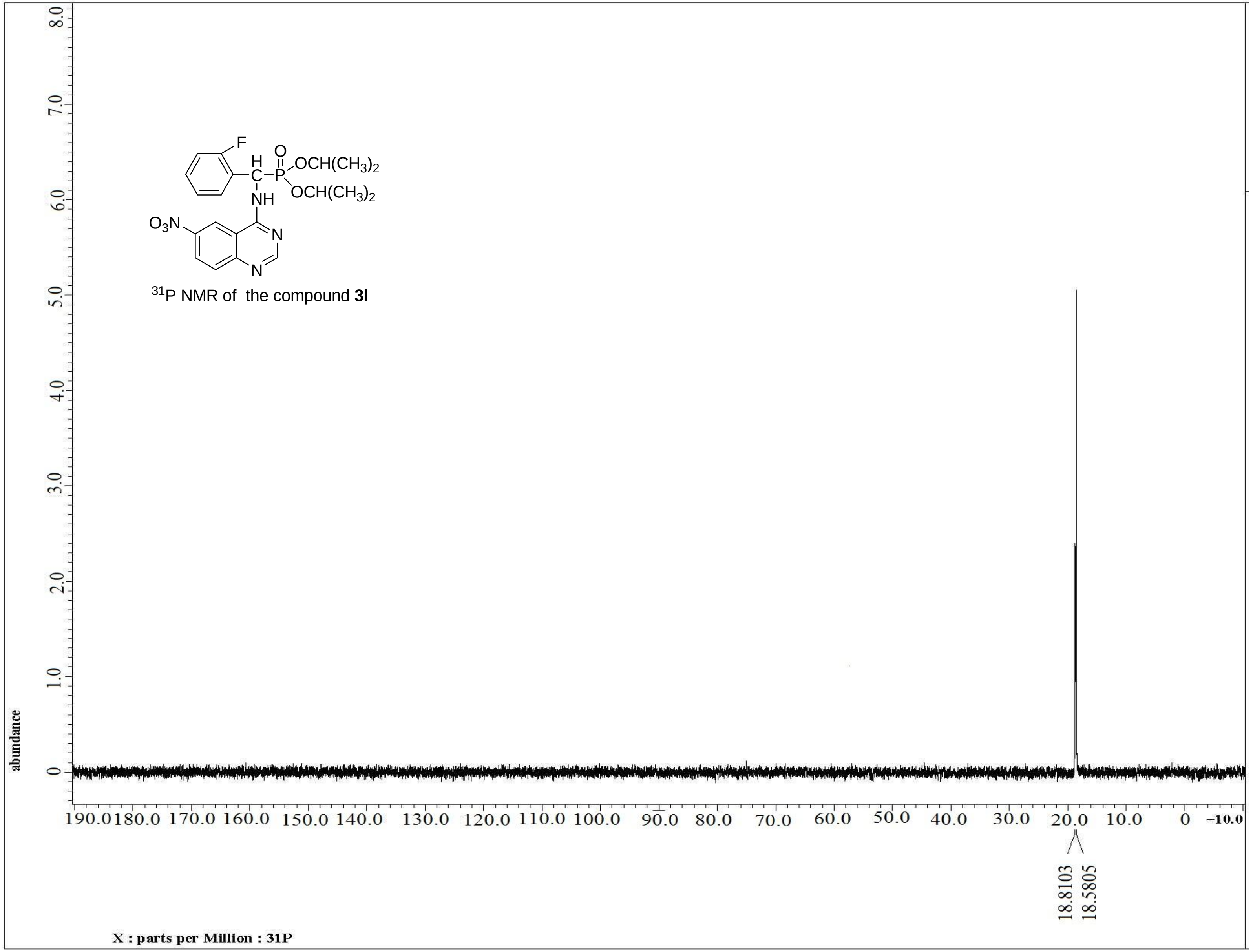

^{31}P NMR of the compound **3l**
abundance
X : parts per Million : 31P
18.8103
18.5805

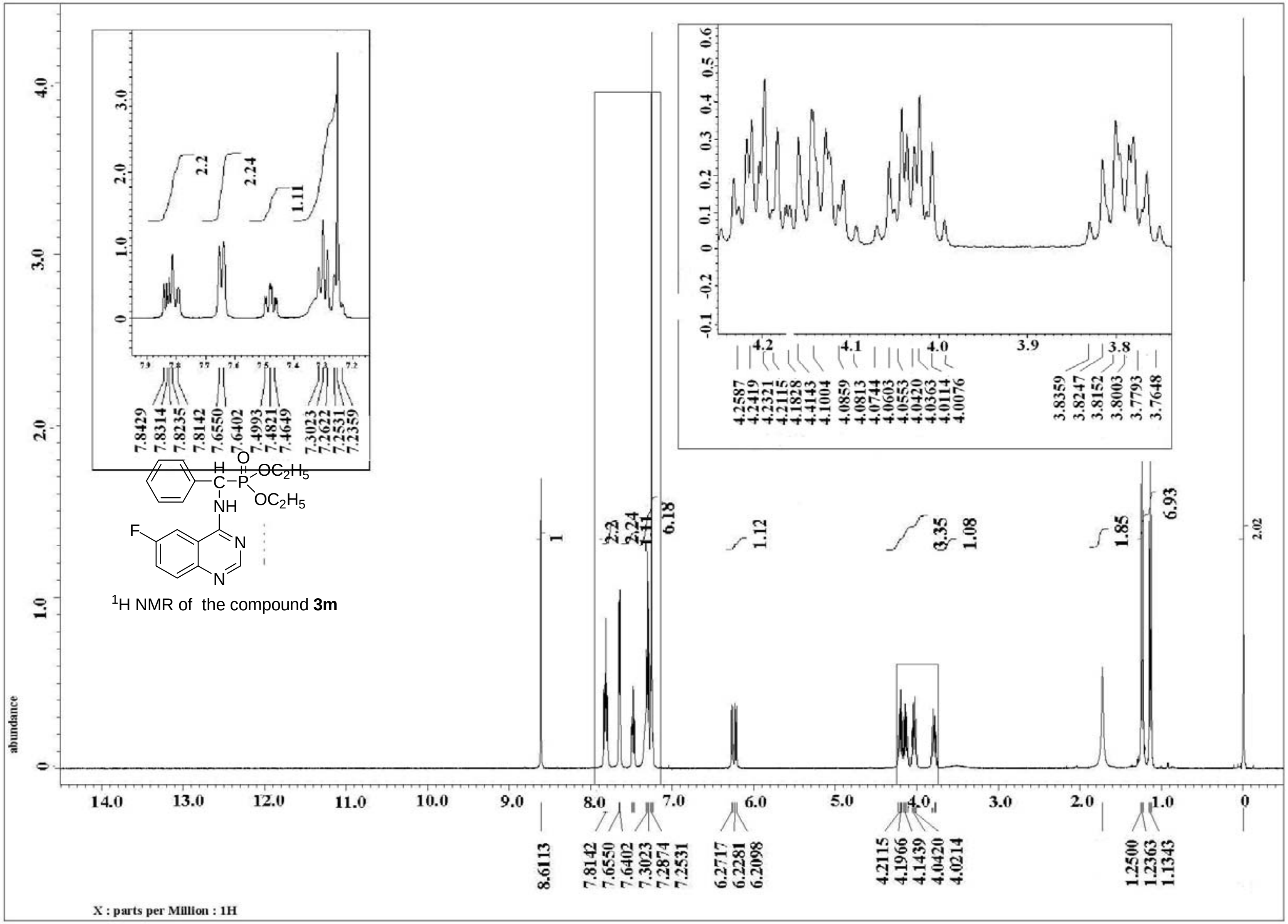

^{1}H NMR of the compound **3m**

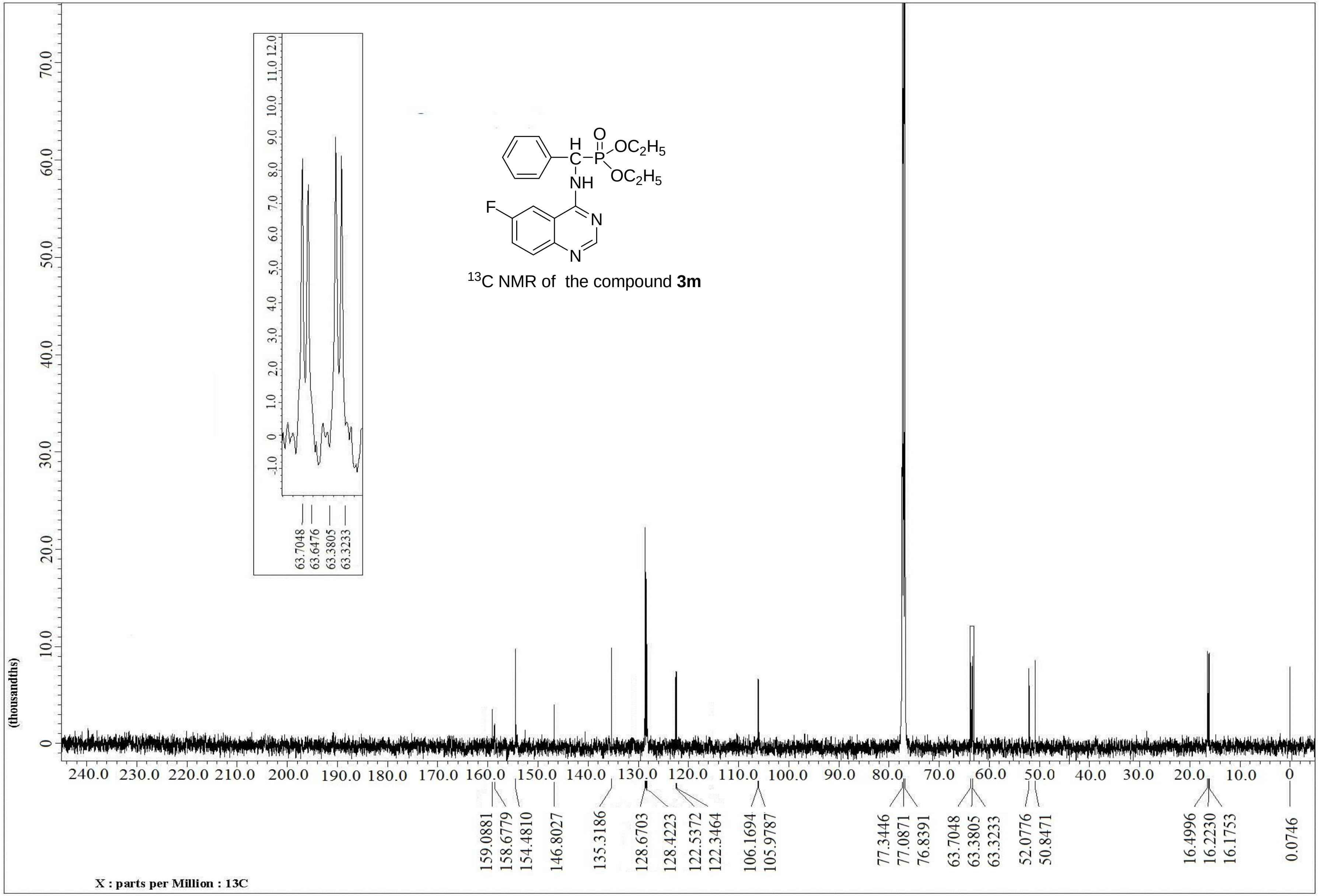

^{13}C NMR of the compound **3m**
X : parts per Million : 13C
(thousandths)
159.0881
158.6779
154.4810
146.8027
135.3186
128.6703
128.4223
122.5372
122.3464
106.1694
105.9787
77.3446
77.0871
76.8391
63.7048
63.3805
63.3233
52.0776
50.8471
16.4996
16.2230
16.1753
0.0746
63.7048
63.6476
63.3805
63.3233

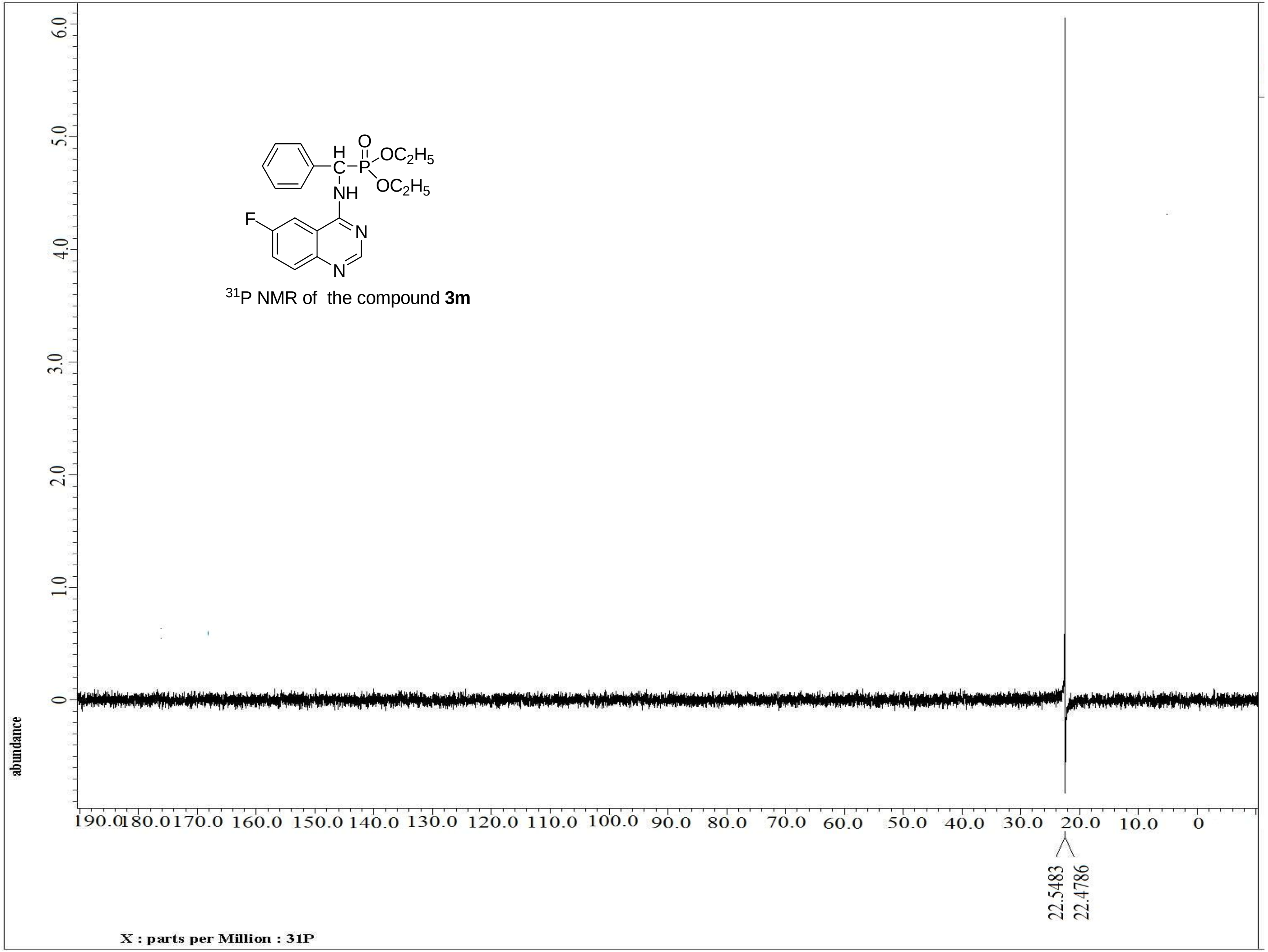

31P NMR of the compound **3m**
OC2H5
OC2H5
NH
F
abundance
X : parts per Million : 31P
22.5483
22.4786

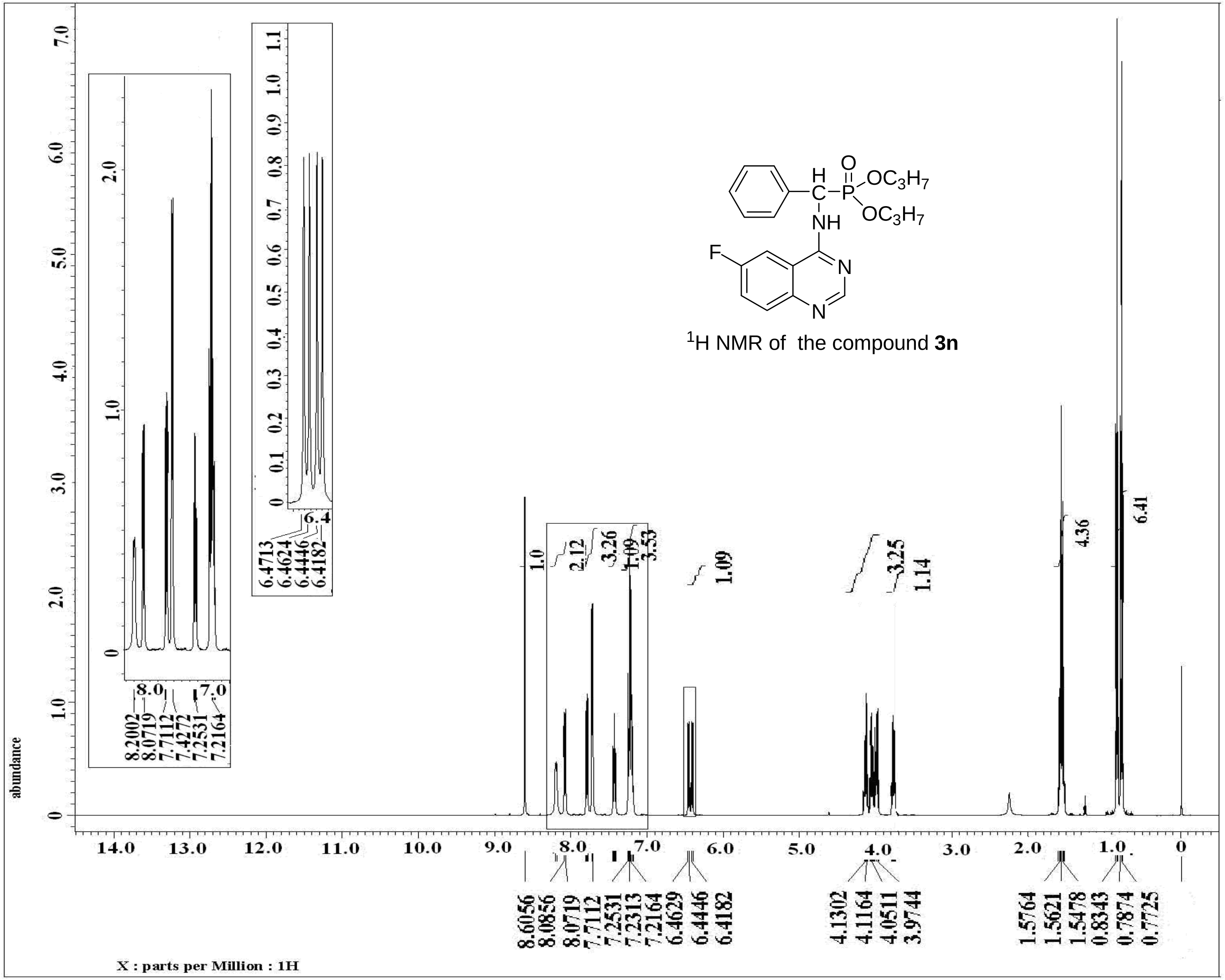

^{1}H NMR of the compound **3n**

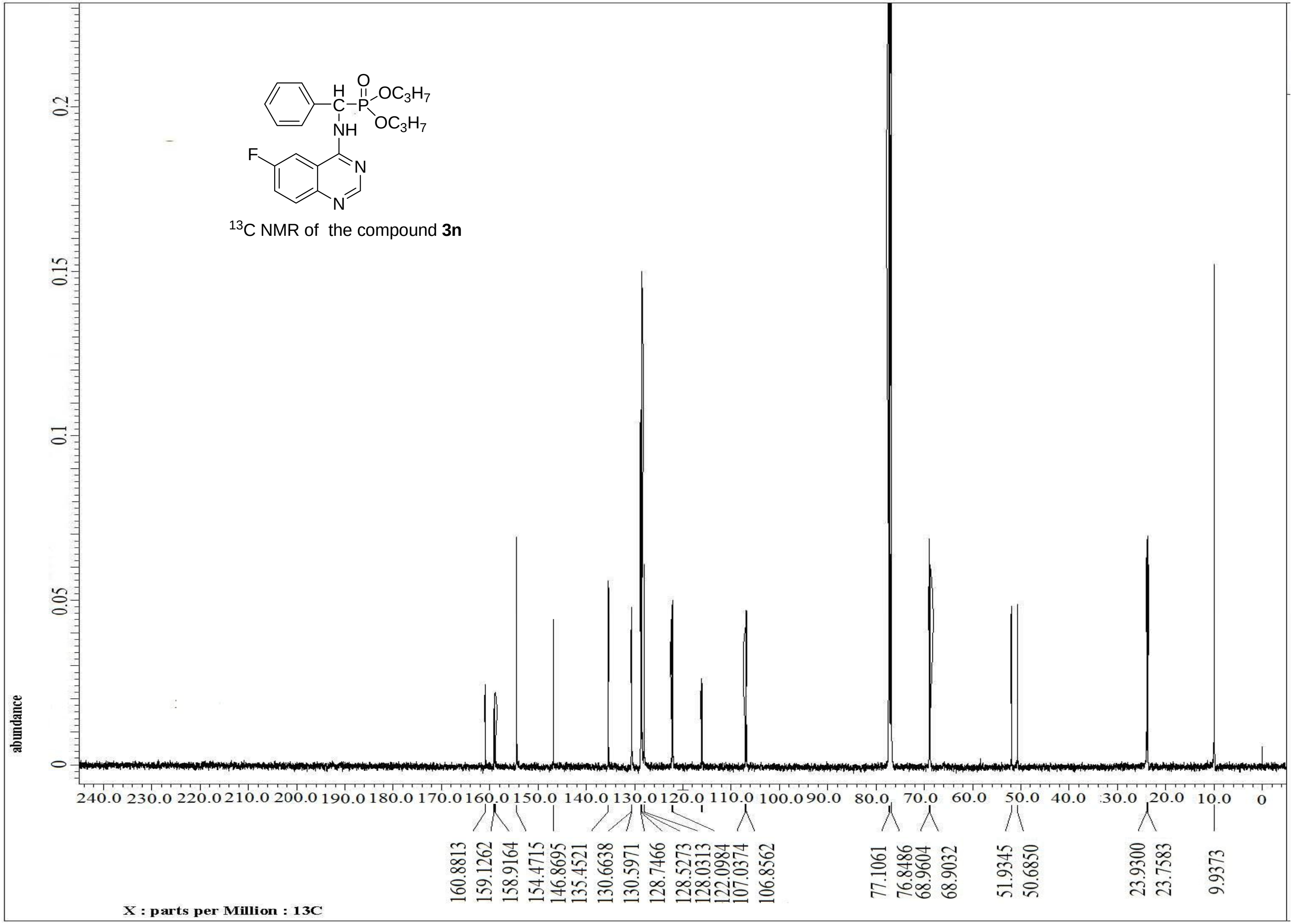

^{13}C NMR of the compound **3n**
abundance
X : parts per Million : 13C
160.8813
159.1262
158.9164
154.4715
146.8695
135.4521
130.6638
130.5971
128.7466
128.5273
128.0313
122.0984
107.0374
106.8562
77.1061
76.8486
68.9604
68.9032
51.9345
50.6850
23.9300
23.7583
9.9373

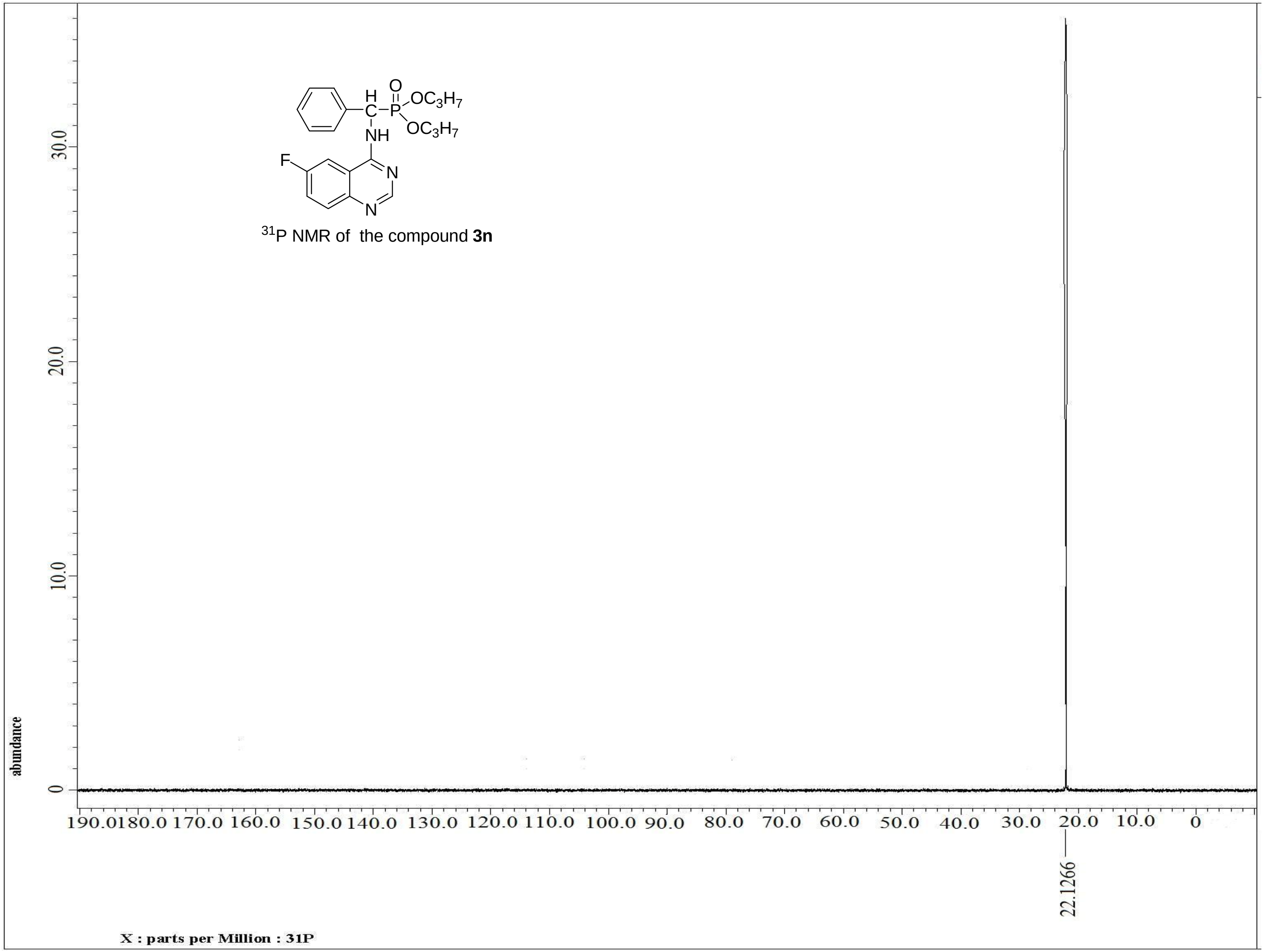

OC3H7
OC3H7
NH
F
N
N
31P NMR of the compound 3n
abundance
30.0
20.0
10.0
0
190.0180.0 170.0 160.0 150.0 140.0 130.0 120.0 110.0 100.0 90.0 80.0 70.0 60.0 50.0 40.0 30.0 20.0 10.0 0
22.1266
X : parts per Million : 31P

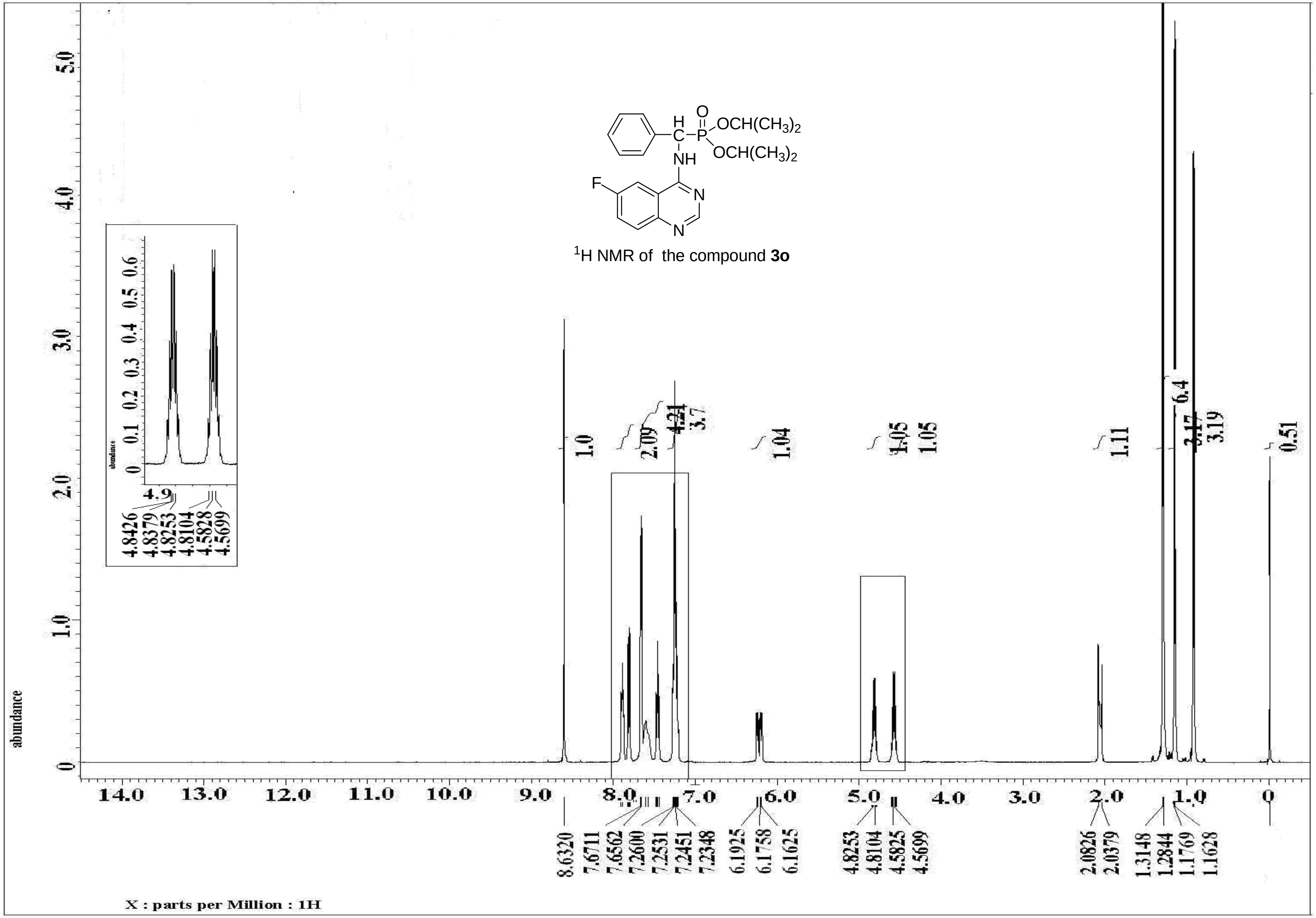

1H NMR of the compound 3o
OCH(CH3)2
OCH(CH3)2
NH
F
N
N
abundance
X : parts per Million : 1H
8.6320
7.6711
7.6562
7.2600
7.2531
7.2451
7.2348
6.1925
6.1758
6.1625
4.8253
4.8104
4.5825
4.5699
2.0826
2.0379
1.3148
1.2844
1.1769
1.1628
4.8426
4.8379
4.8253
4.8104
4.5828
4.5699

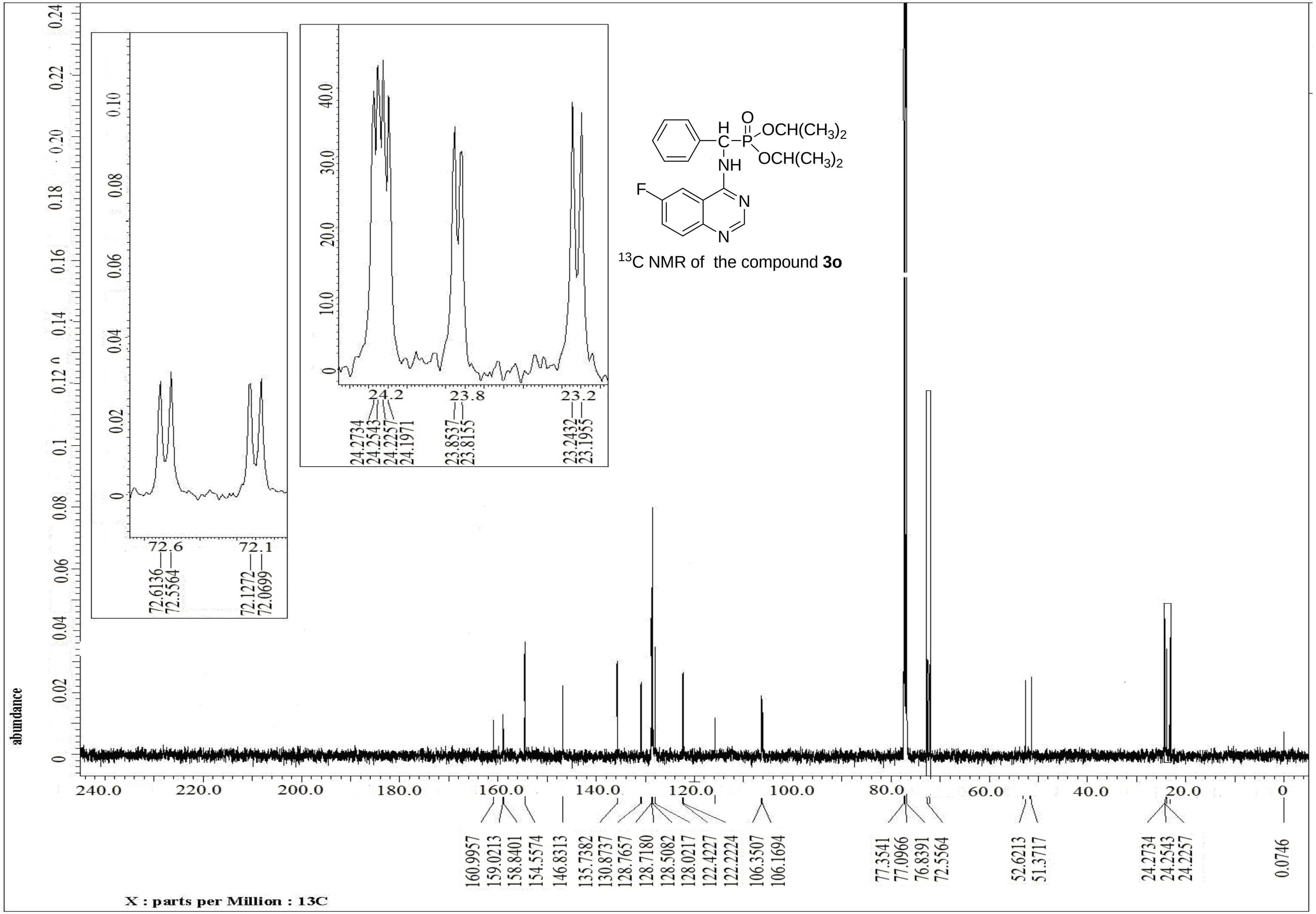
OCH(CH3)2
OCH(CH3)2
NH
F
N
N
13C NMR of the compound 3o
abundance
X : parts per Million : 13C
160.9957
159.0213
158.8401
154.5574
146.8313
135.7382
130.8737
128.7657
128.7180
128.5082
128.0217
122.4227
122.2224
106.3507
106.1694
77.3541
77.0966
76.8391
72.5564
52.6213
51.3717
24.2734
24.2543
24.2257
0.0746
72.6136
72.5564
72.1272
72.0699
24.2734
24.2543
24.2257
24.1971
23.8537
23.8155
23.2432
23.1955

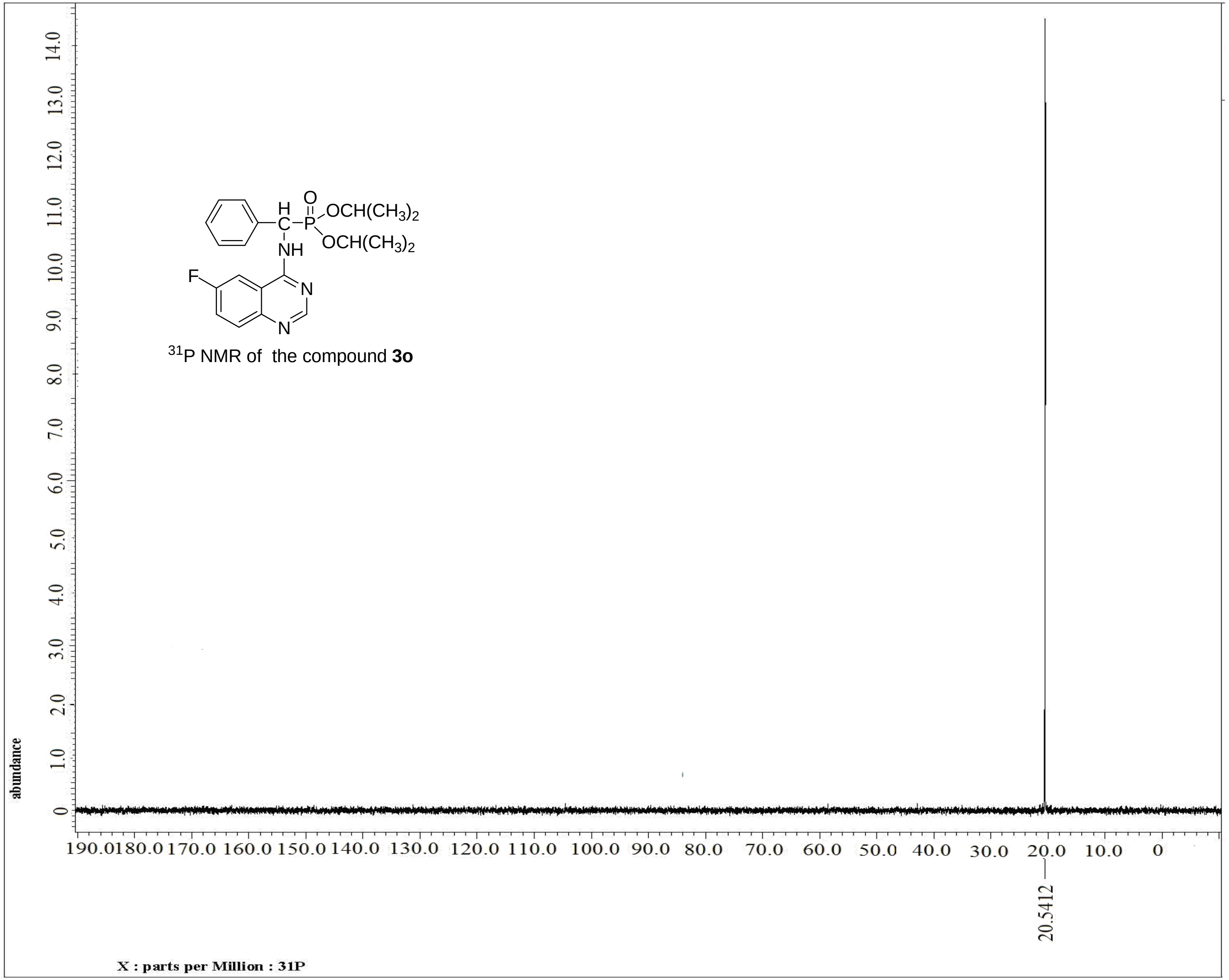

OCH(CH3)2
OCH(CH3)2
NH
F
N
N
31P NMR of the compound 3o
abundance
20.5412
X : parts per Million : 31P

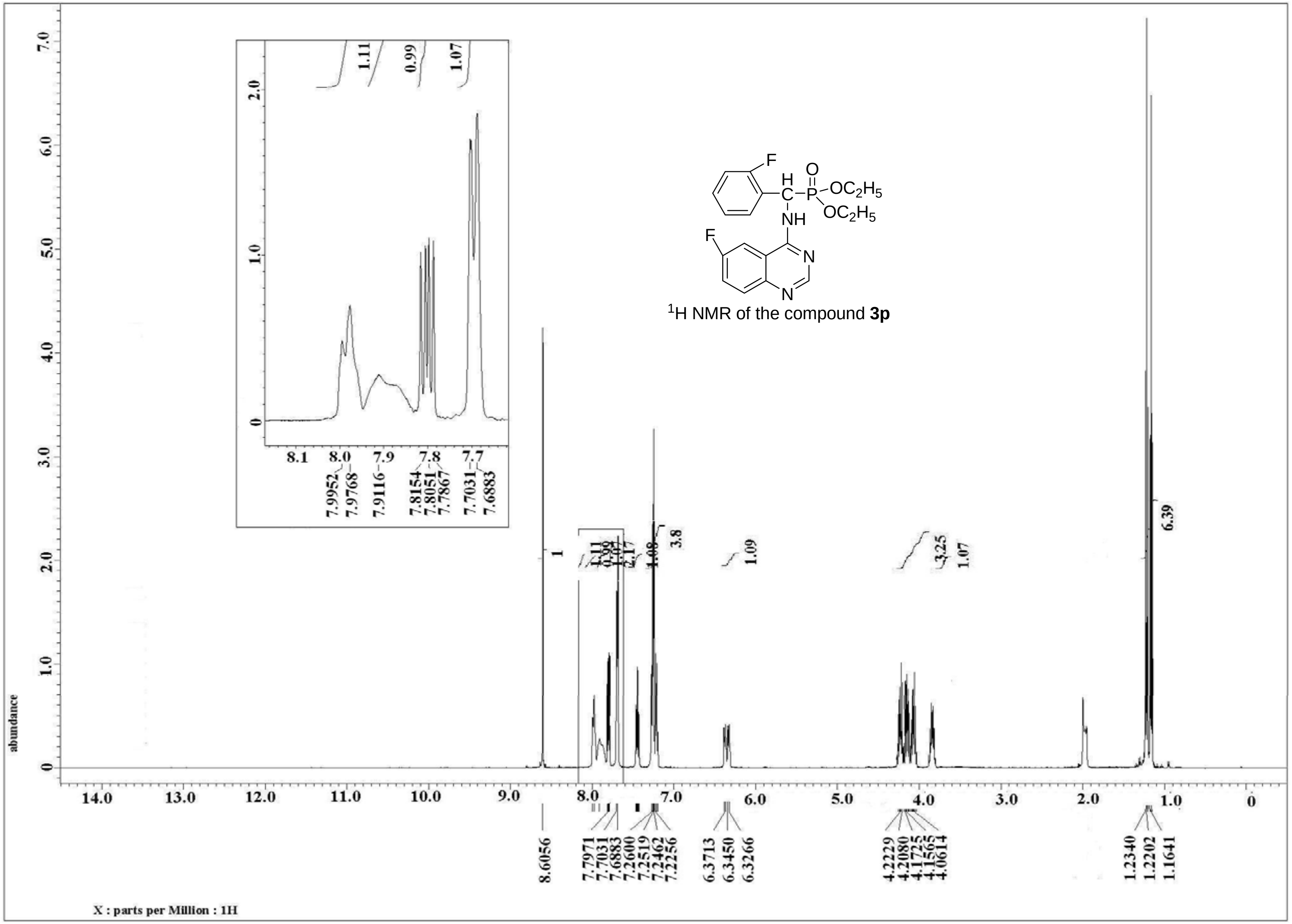

^{1}H NMR of the compound **3p**

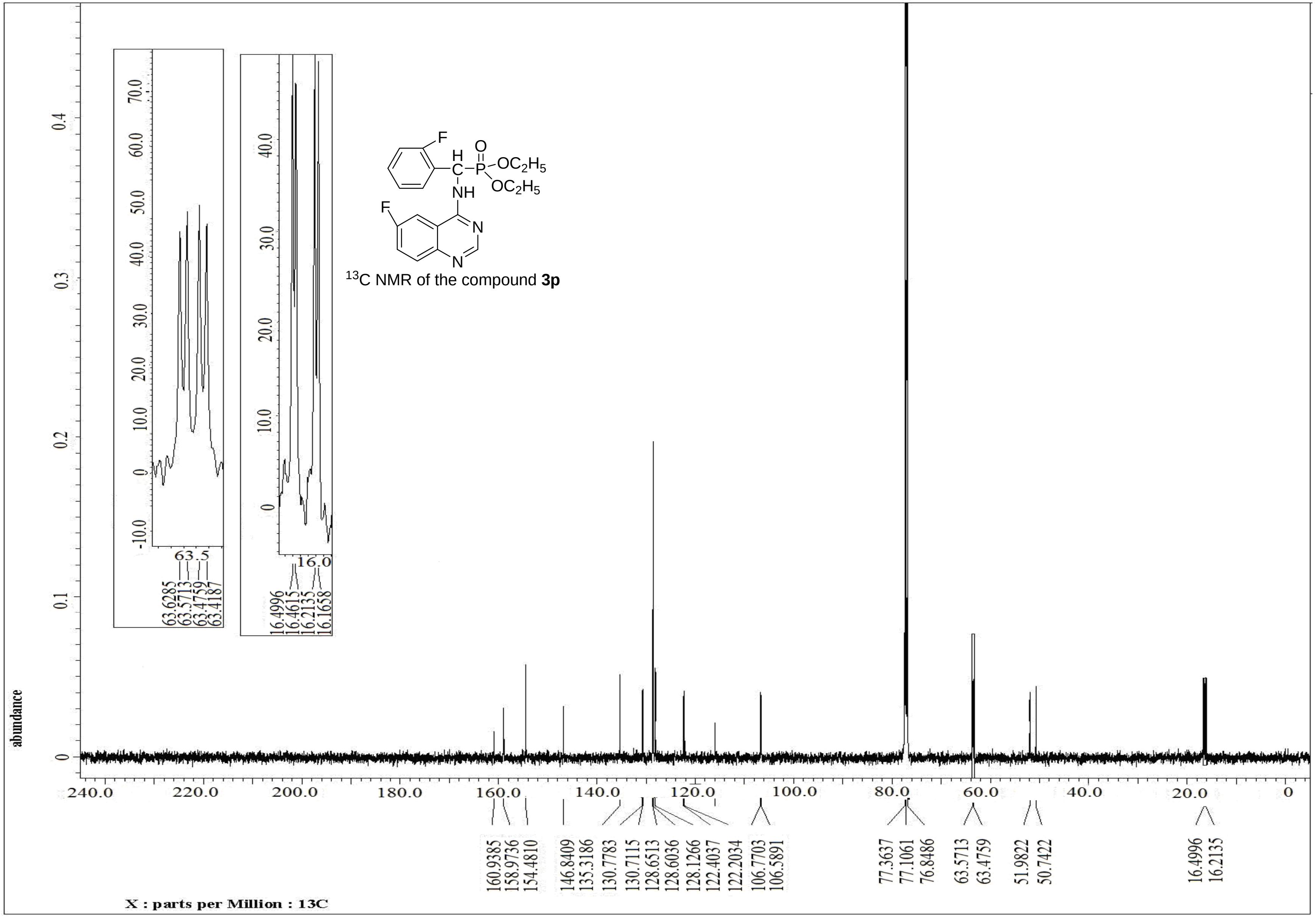

^{13}C NMR of the compound **3p**
abundance
X : parts per Million : 13C
160.9385
158.9736
154.4810
146.8409
135.3186
130.7783
130.7115
128.6513
128.6036
128.1266
122.4037
122.2034
106.7703
106.5891
77.3637
77.1061
76.8486
63.5713
63.4759
51.9822
50.7422
16.4996
16.2135
63.6285
63.5713
63.4759
63.4187
16.4996
16.4615
16.2135
16.1658

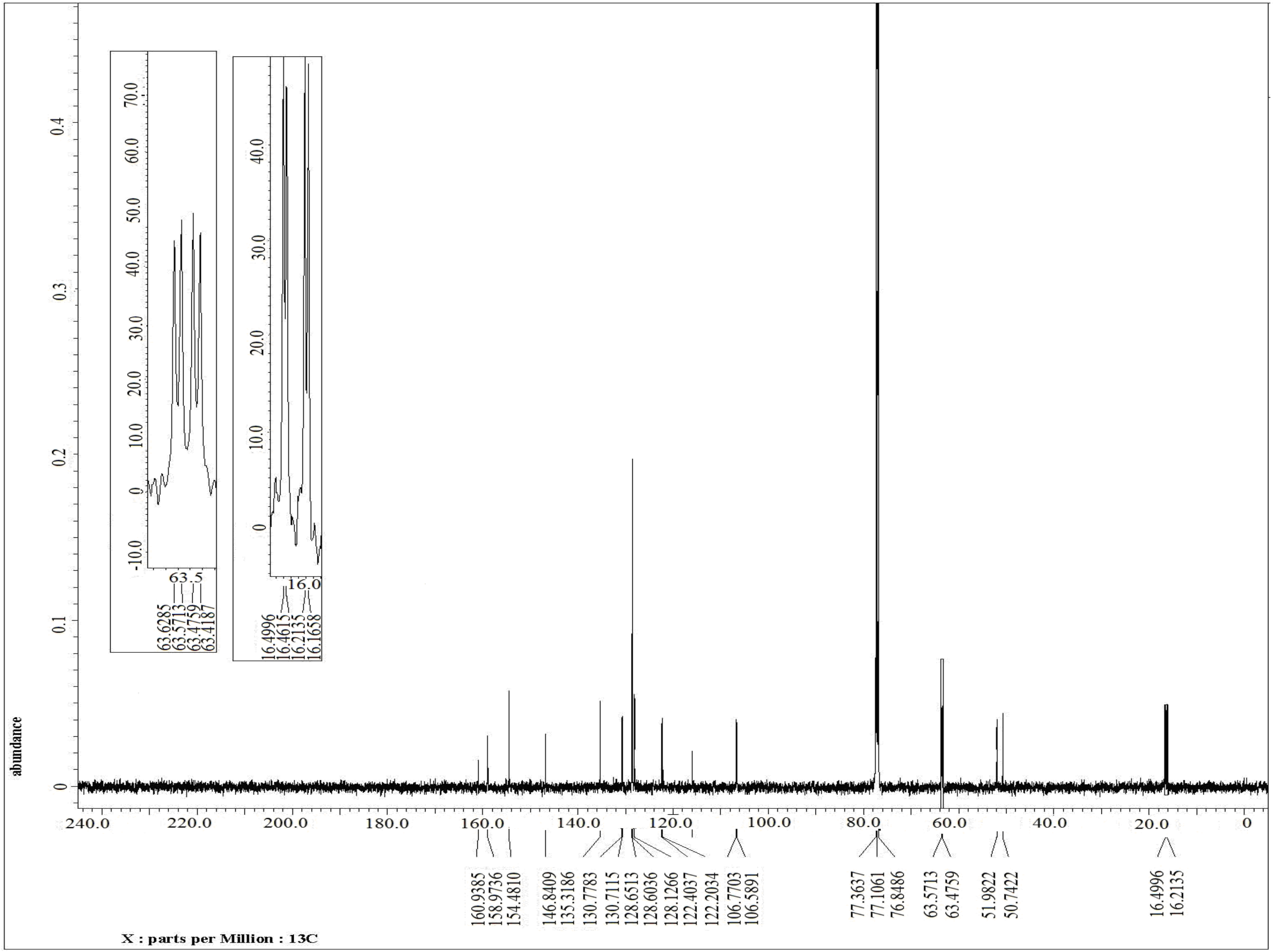
abundance
X : parts per Million : 13C
160.9385
158.9736
154.4810
146.8409
135.3186
130.7783
130.7115
128.6513
128.6036
128.1266
122.4037
122.2034
106.7703
106.5891
77.3637
77.1061
76.8486
63.5713
63.4759
51.9822
50.7422
16.4996
16.2135
63.6285
63.5713
63.4759
63.4187
16.4996
16.4615
16.2135
16.1658

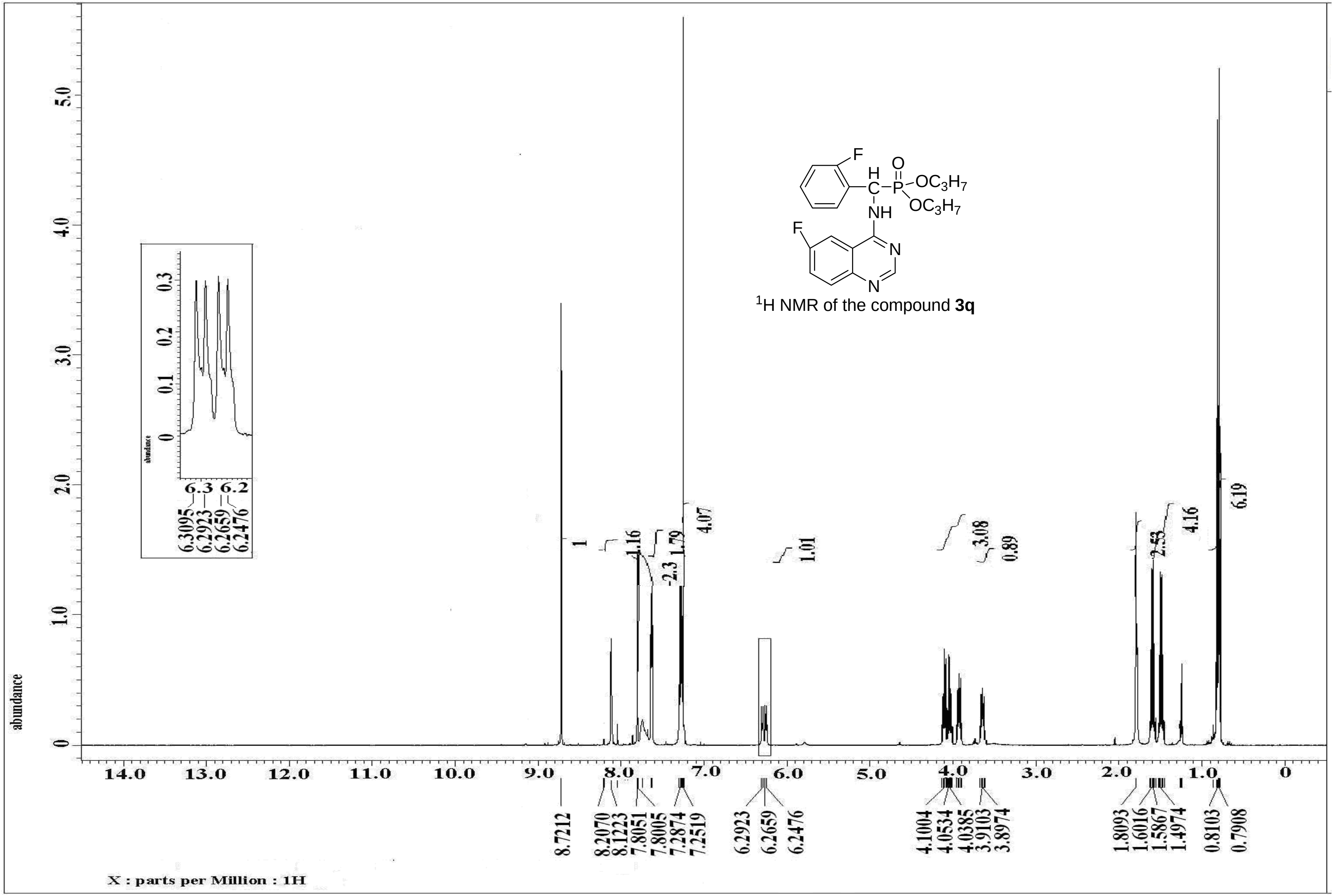

F
H
O
C–P–OC3H7
OC3H7
NH
F
N
N
1H NMR of the compound 3q
abundance
X : parts per Million : 1H

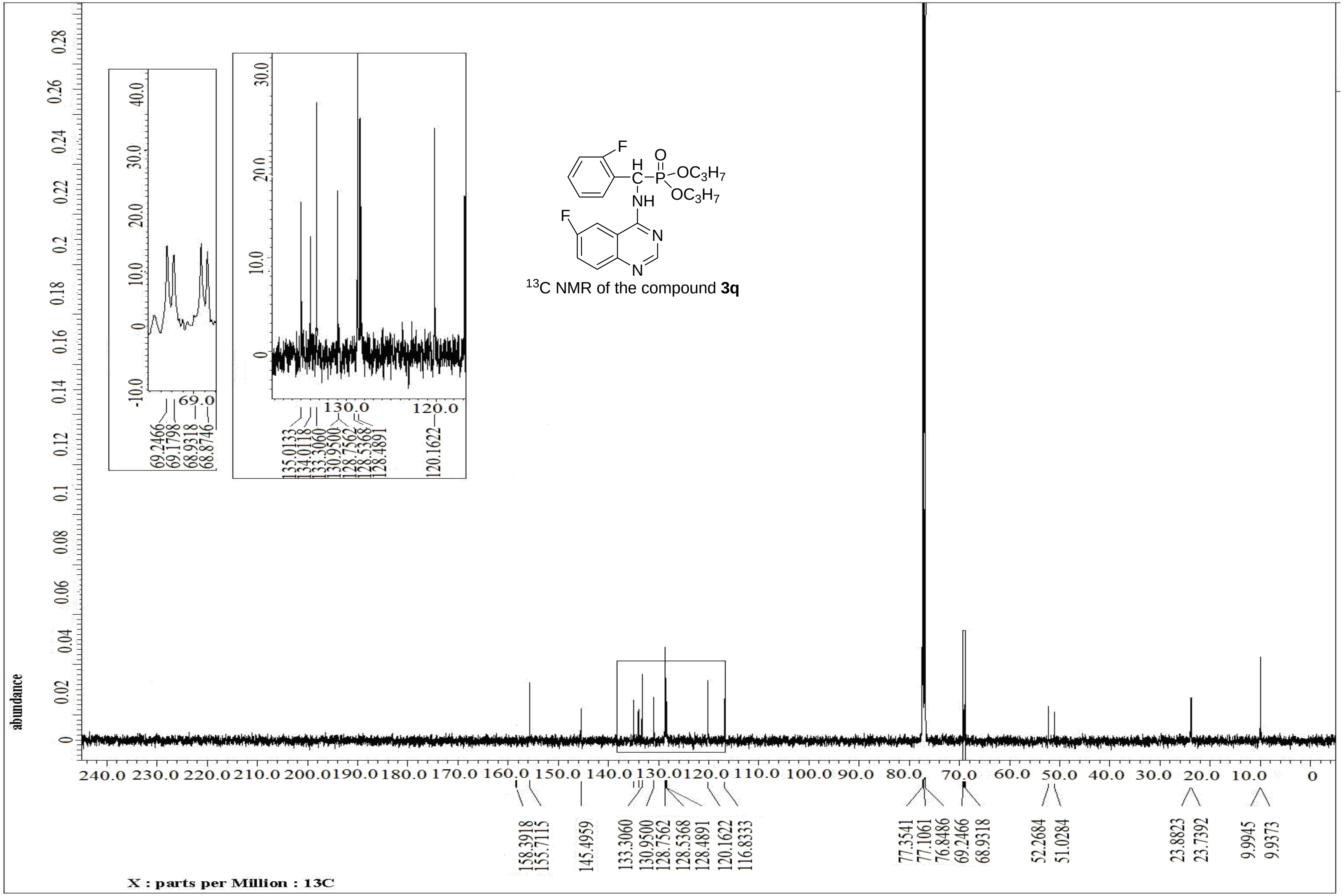

^{13}C NMR of the compound **3q**
abundance
X : parts per Million : 13C
158.3918
155.7115
145.4959
133.3060
130.9500
128.7562
128.5368
128.4891
120.1622
116.8333
77.3541
77.1061
76.8486
69.2466
68.9318
52.2684
51.0284
23.8823
23.7392
9.9945
9.9373
69.2466
69.1798
68.9318
68.8746
135.0133
134.0118
133.3060
130.9500
128.7562
128.5368
128.4891
120.1622

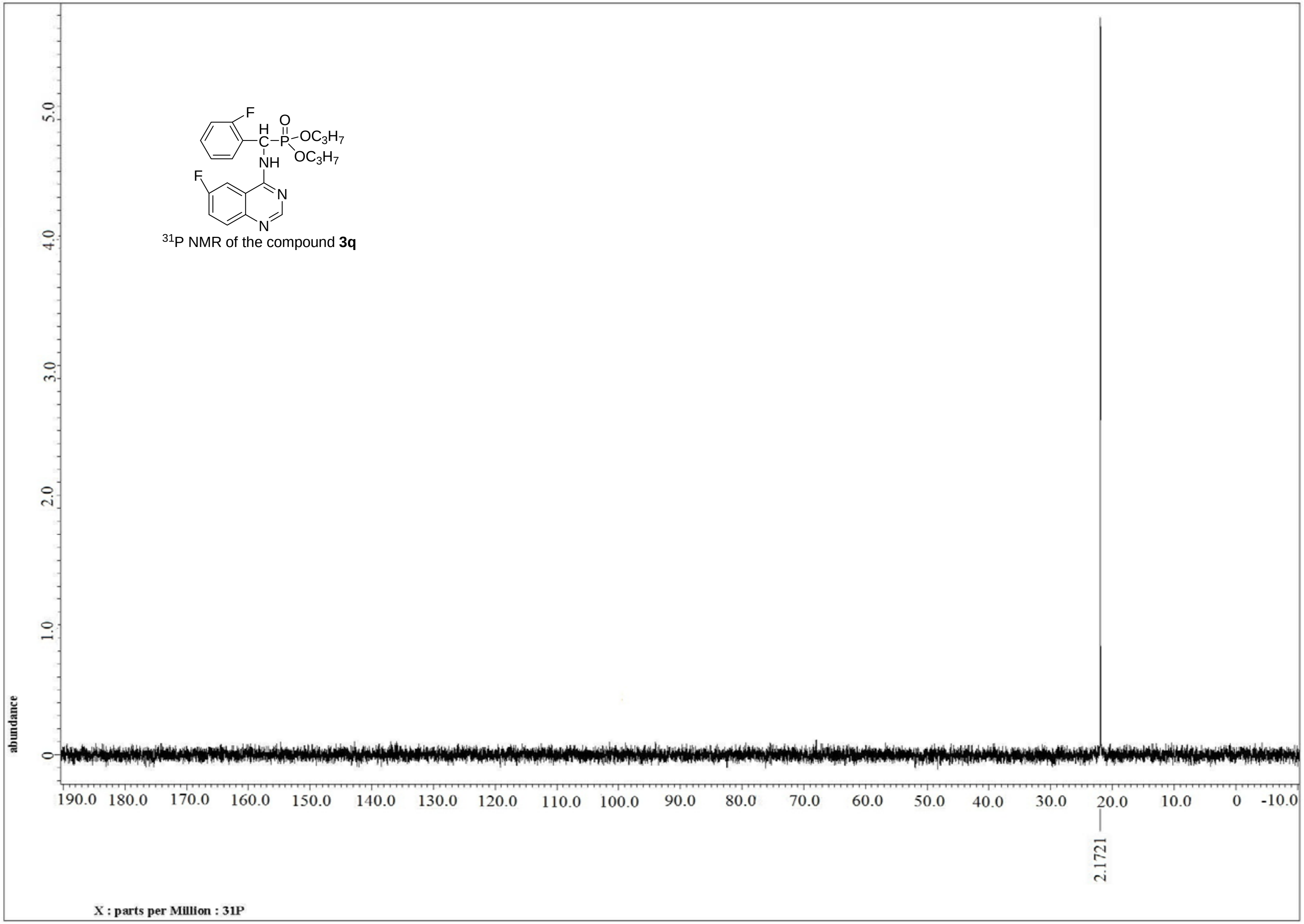

F
H
O
C
P
OC3H7
OC3H7
NH
F
N
N
31P NMR of the compound 3q
abundance
0
1.0
2.0
3.0
4.0
5.0
190.0 180.0 170.0 160.0 150.0 140.0 130.0 120.0 110.0 100.0 90.0 80.0 70.0 60.0 50.0 40.0 30.0 20.0 10.0 0 -10.0
2.1721
X : parts per Million : 31P

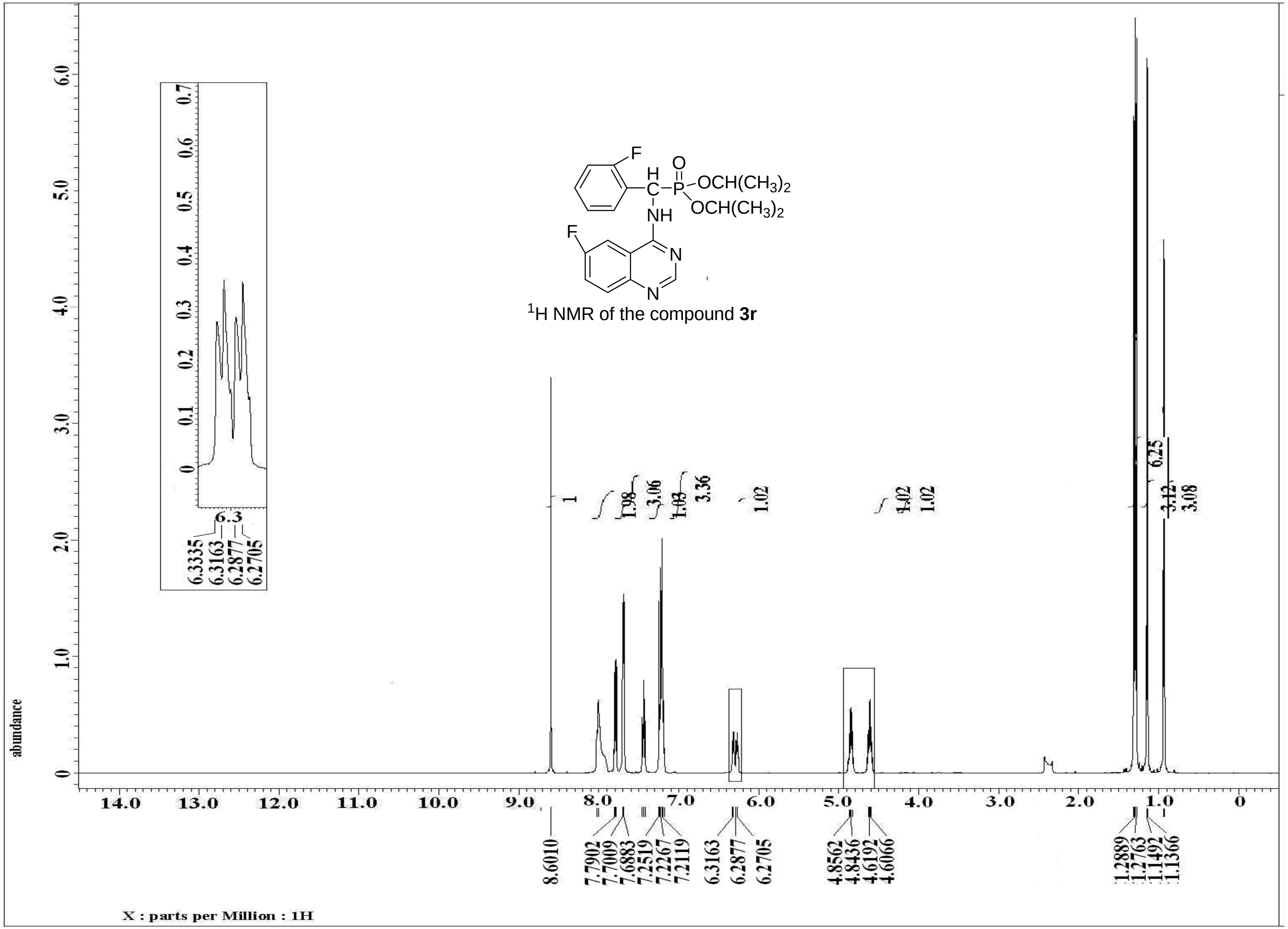

^{1}H NMR of the compound **3r**

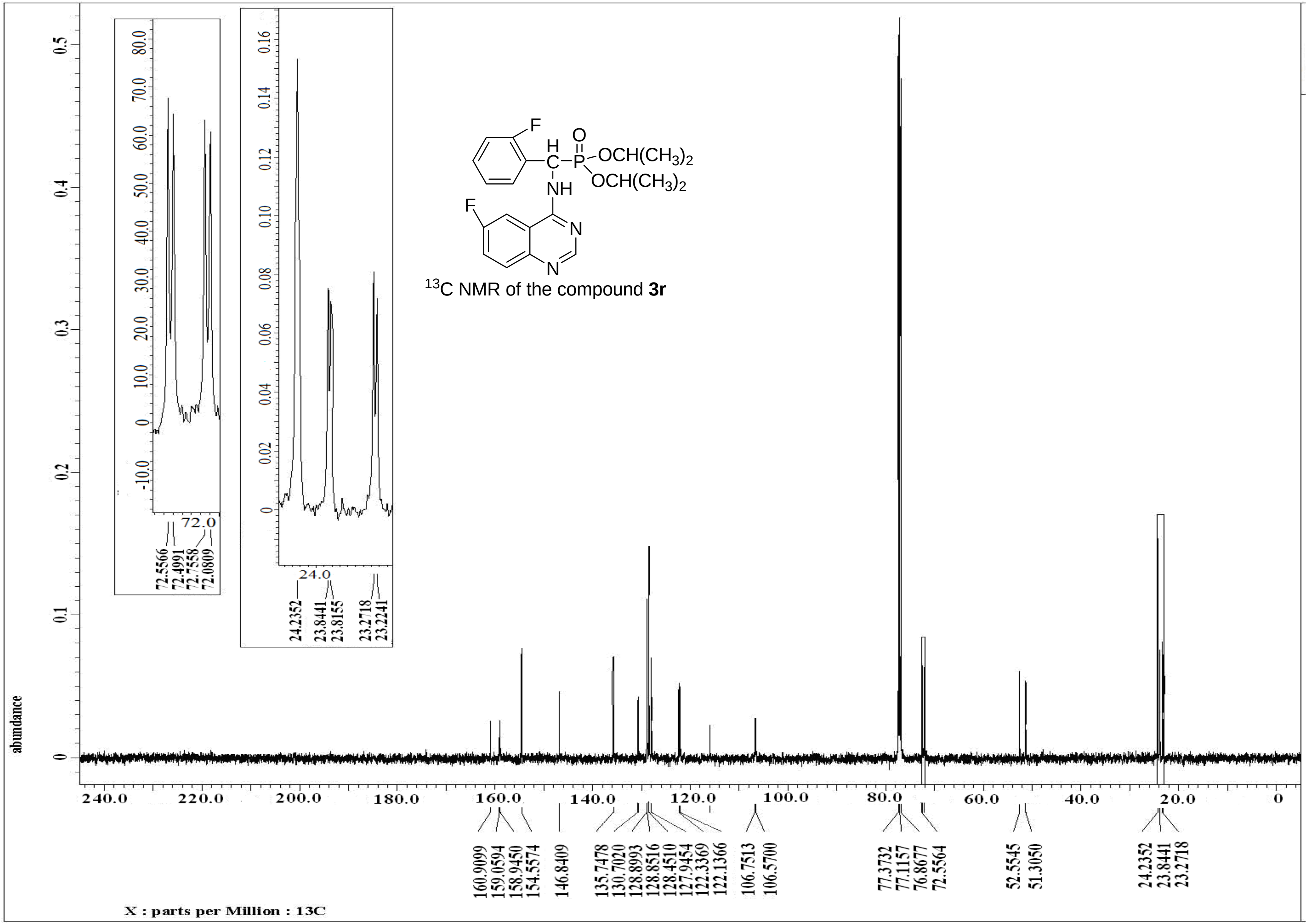

^{13}C NMR of the compound **3r**
abundance
X : parts per Million : 13C
160.9099
159.0594
158.9450
154.5574
146.8409
135.7478
130.7020
128.8993
128.8516
128.4510
127.9454
122.3369
122.1366
106.7513
106.5700
77.3732
77.1157
76.8677
72.5564
52.5545
51.3050
24.2352
23.8441
23.2718
72.5566
72.4991
72.7558
72.0809
24.2352
23.8441
23.8155
23.2718
23.2241

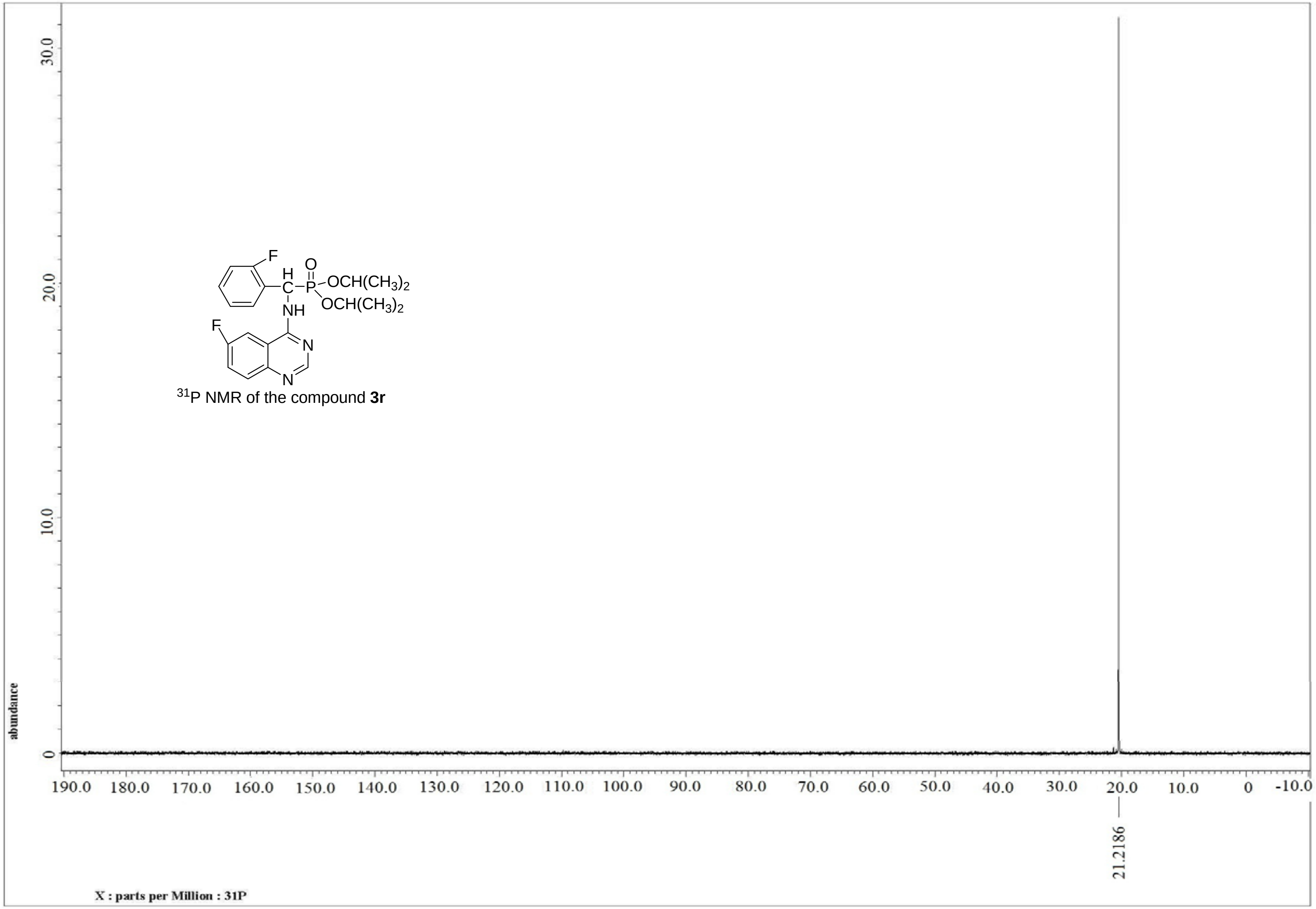
F
H
O
C
P
OCH(CH3)2
OCH(CH3)2
NH
F
N
N
31P NMR of the compound 3r
30.0
20.0
10.0
0
abundance
190.0 180.0 170.0 160.0 150.0 140.0 130.0 120.0 110.0 100.0 90.0 80.0 70.0 60.0 50.0 40.0 30.0 20.0 10.0 0 -10.0
21.2186
X : parts per Million : 31P

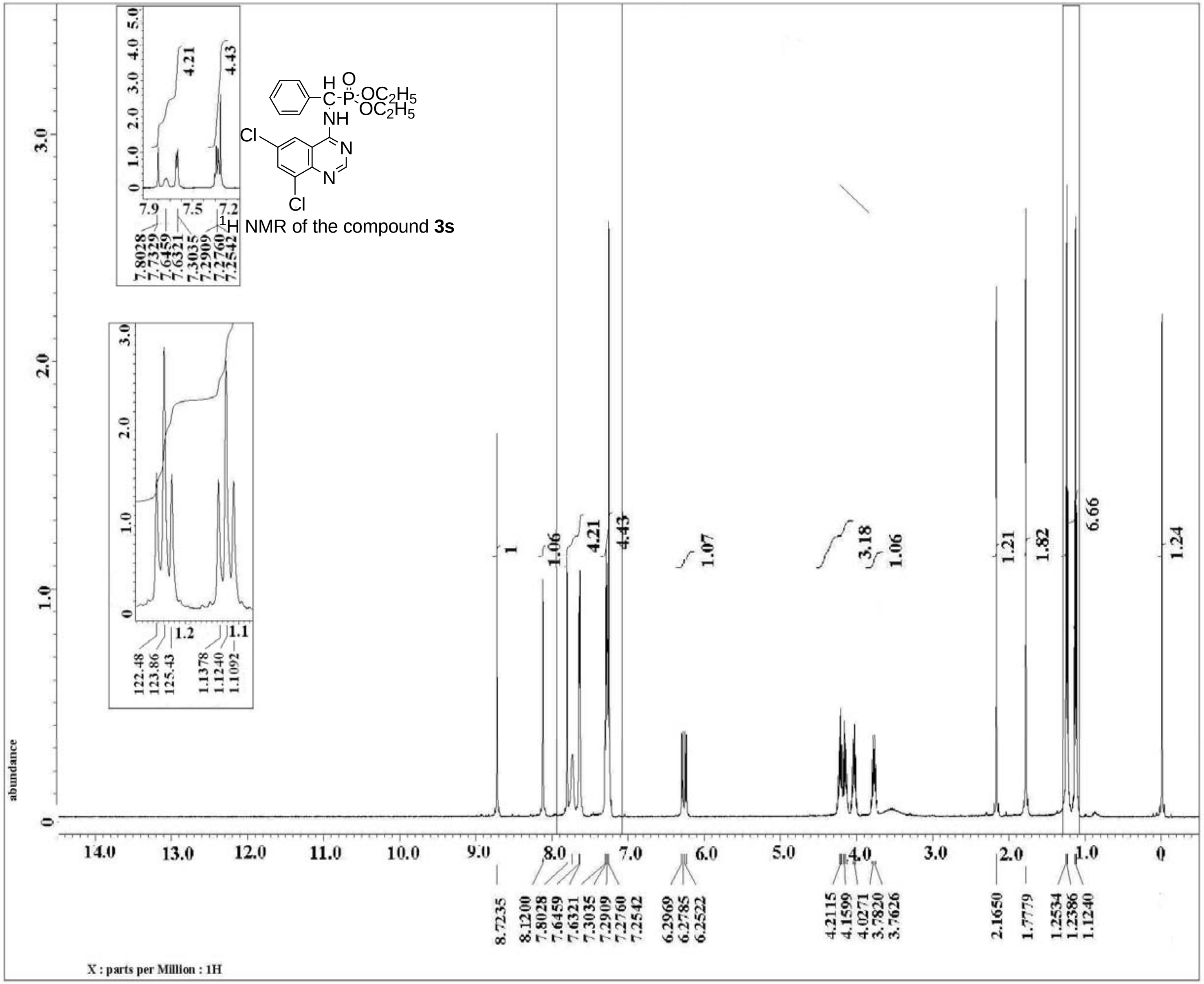
^{1}H NMR of the compound **3s**
abundance
X : parts per Million : 1H
8.7235
8.1200
7.8028
7.6459
7.6321
7.3035
7.2909
7.2760
7.2542
6.2969
6.2785
6.2522
4.2115
4.1599
4.0271
3.7820
3.7626
2.1650
1.7779
1.2534
1.2386
1.1240
1.1378
1.1092

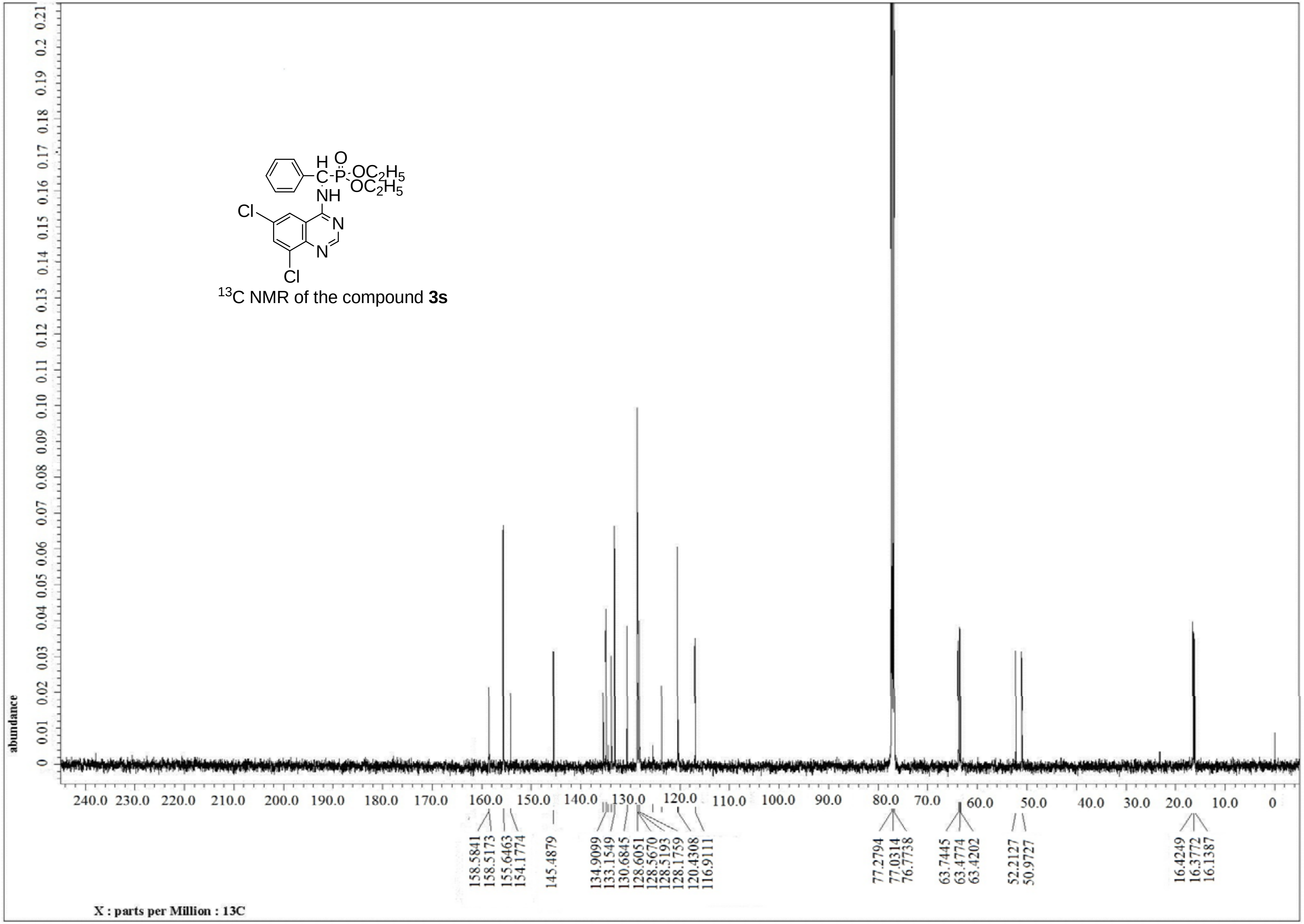

^{13}C NMR of the compound **3s**
abundance
X : parts per Million : 13C
158.5841
158.5173
155.6463
154.1774
145.4879
134.9099
133.1549
130.6845
128.6051
128.5670
128.5193
128.1759
120.4308
116.9111
77.2794
77.0314
76.7738
63.7445
63.4774
63.4202
52.2127
50.9727
16.4249
16.3772
16.1387

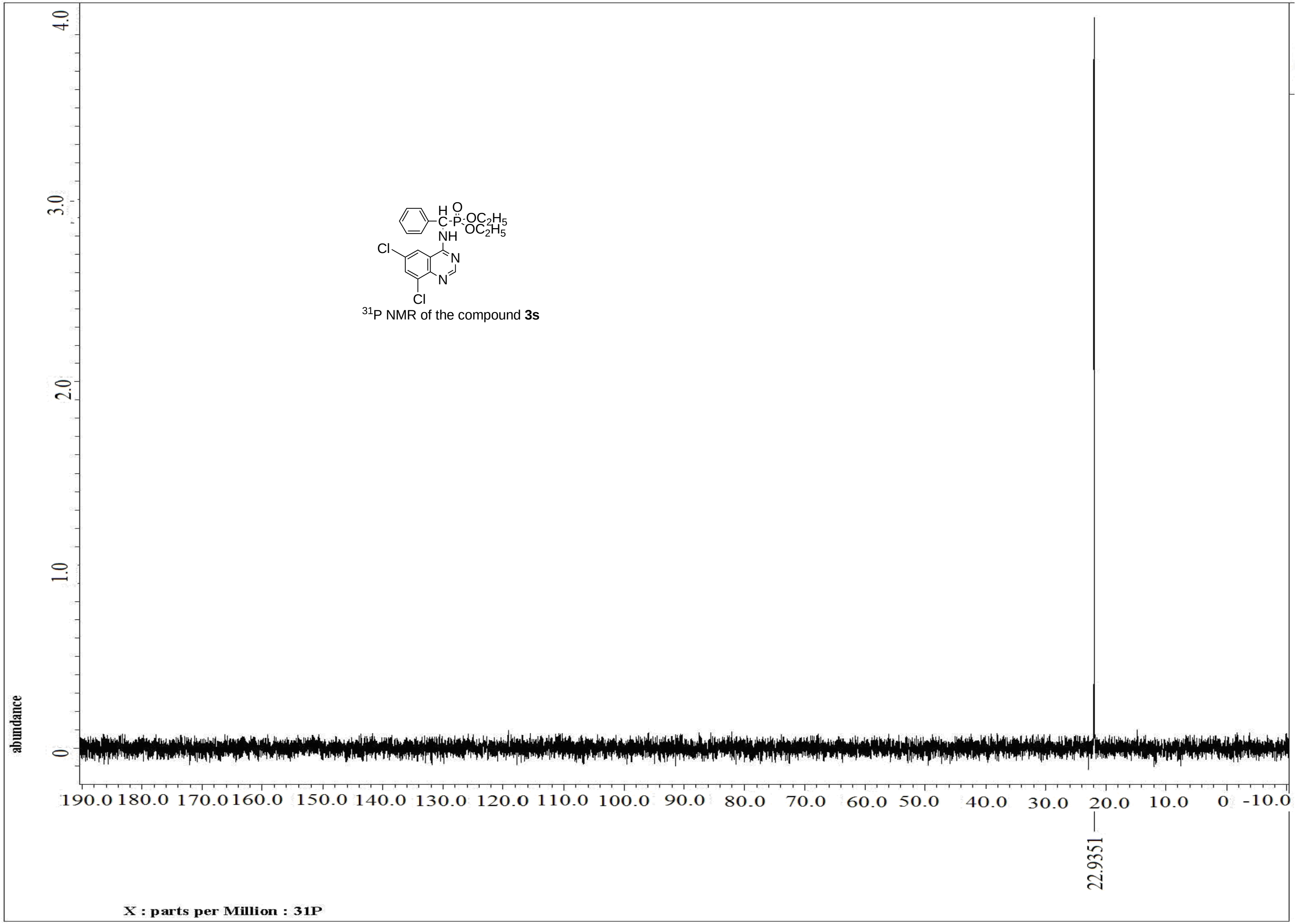

^{31}P NMR of the compound **3s**
abundance
0
1.0
2.0
3.0
4.0
190.0 180.0 170.0 160.0 150.0 140.0 130.0 120.0 110.0 100.0 90.0 80.0 70.0 60.0 50.0 40.0 30.0 20.0 10.0 0 -10.0
22.9351
X : parts per Million : 31P

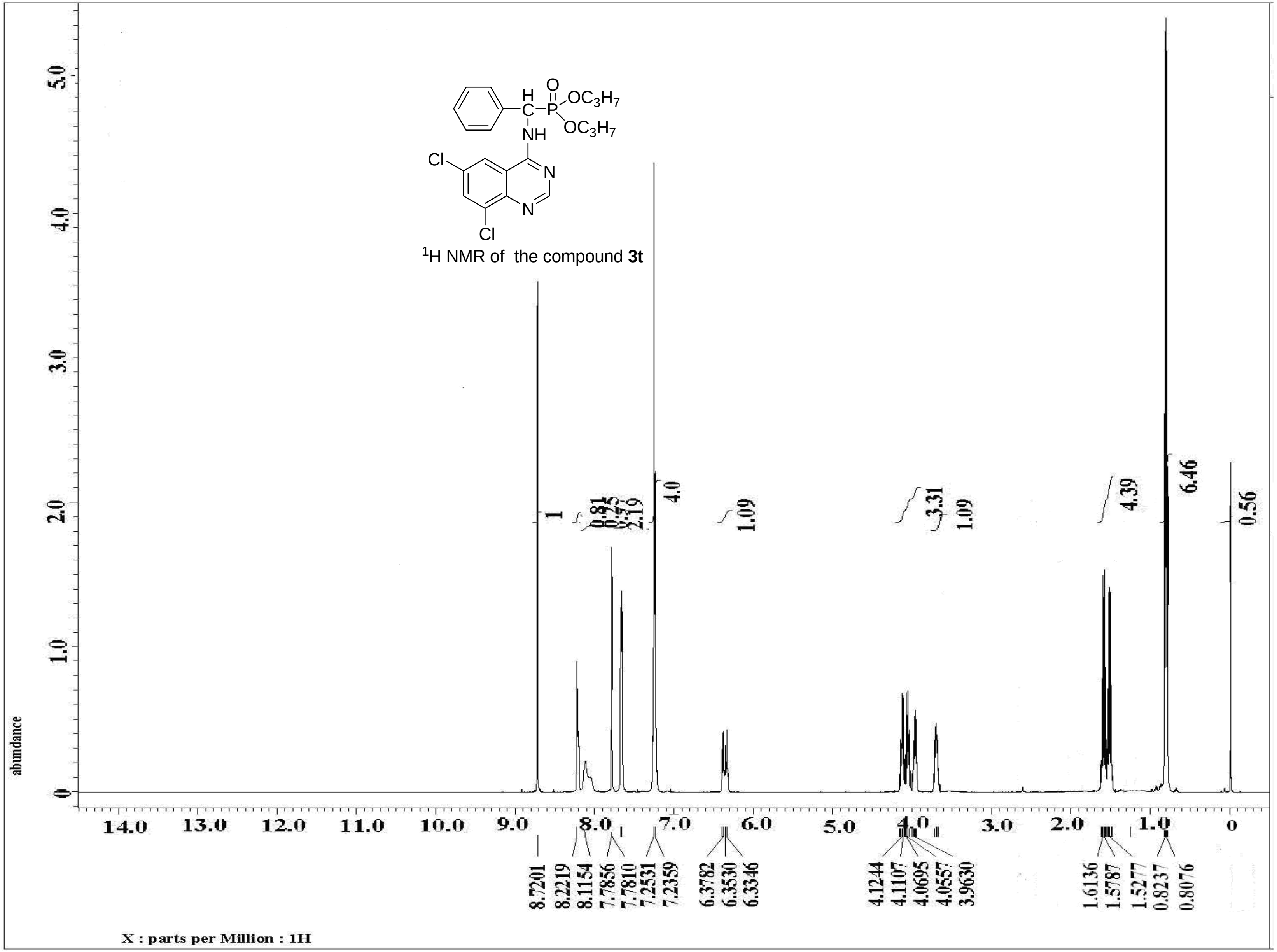

OC3H7
OC3H7
NH
Cl
N
N
Cl
1H NMR of the compound 3t
abundance
X : parts per Million : 1H

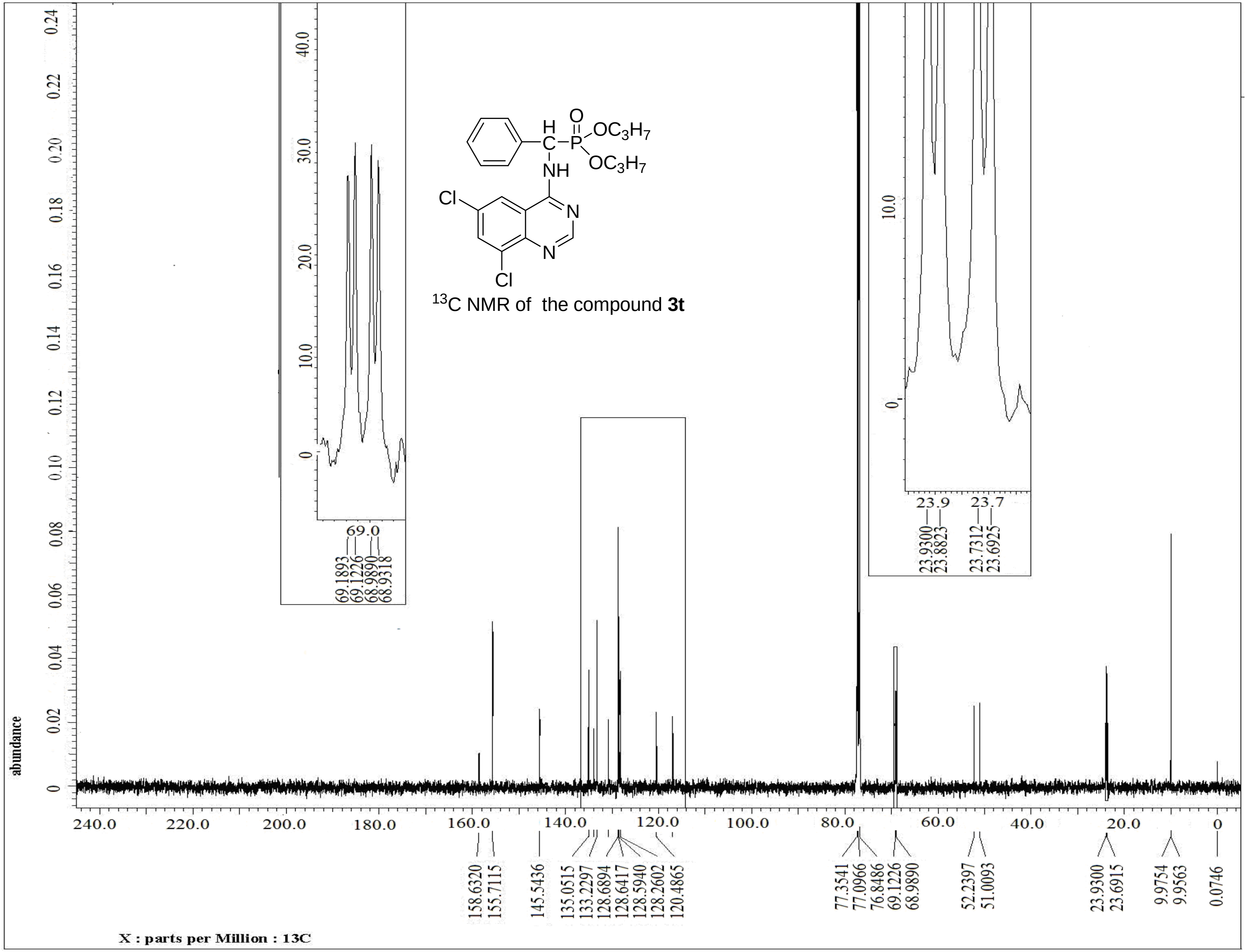

13C NMR of the compound **3t**
abundance
X : parts per Million : 13C
158.6320
155.7115
145.5436
135.0515
133.2297
128.6894
128.6417
128.5940
128.2602
120.4865
77.3541
77.0966
76.8486
69.1226
68.9890
52.2397
51.0093
23.9300
23.6915
9.9754
9.9563
0.0746
69.1893
69.1226
68.9890
68.9318
23.9300
23.8823
23.7312
23.6925

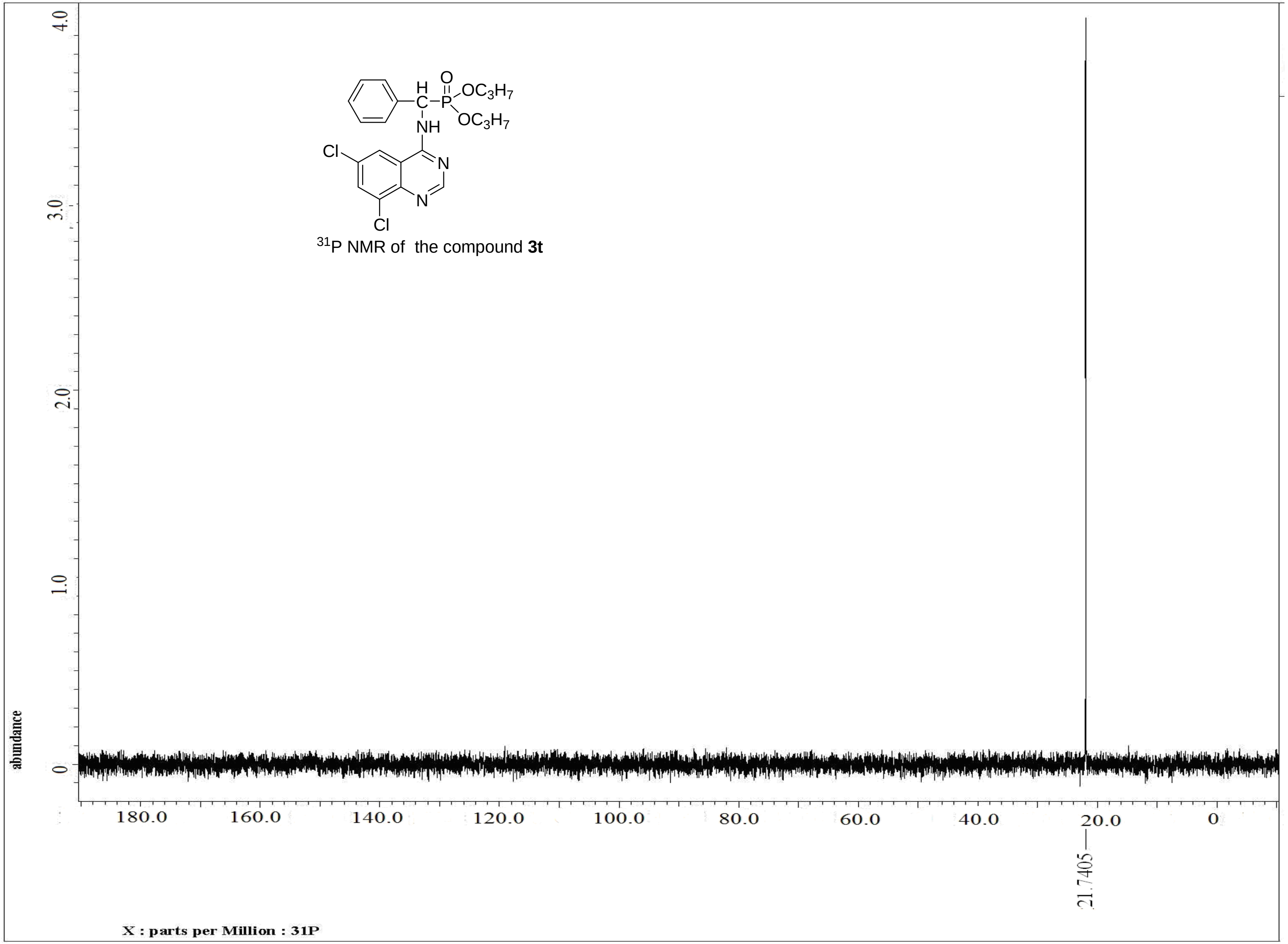

31P NMR of the compound 3t
abundance
4.0
3.0
2.0
1.0
0
180.0
160.0
140.0
120.0
100.0
80.0
60.0
40.0
20.0
0
21.7405
X : parts per Million : 31P

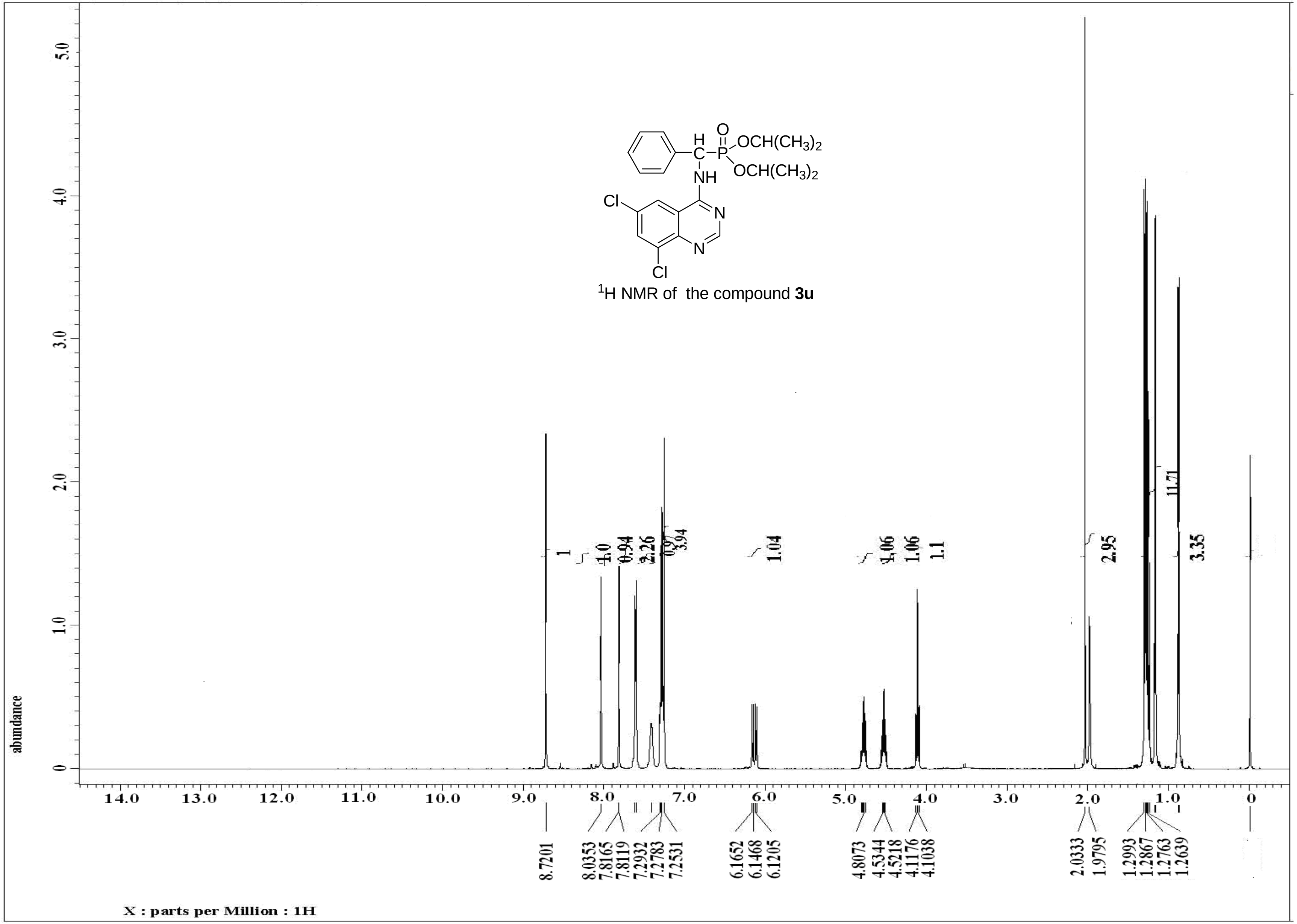

OCH(CH3)2
OCH(CH3)2
NH
Cl
N
N
Cl
1H NMR of the compound 3u
abundance
X : parts per Million : 1H
8.7201
8.0353
7.8165
7.8119
7.2932
7.2783
7.2531
6.1652
6.1468
6.1205
4.8073
4.5344
4.5218
4.1176
4.1038
2.0333
1.9795
1.2993
1.2867
1.2763
1.2639

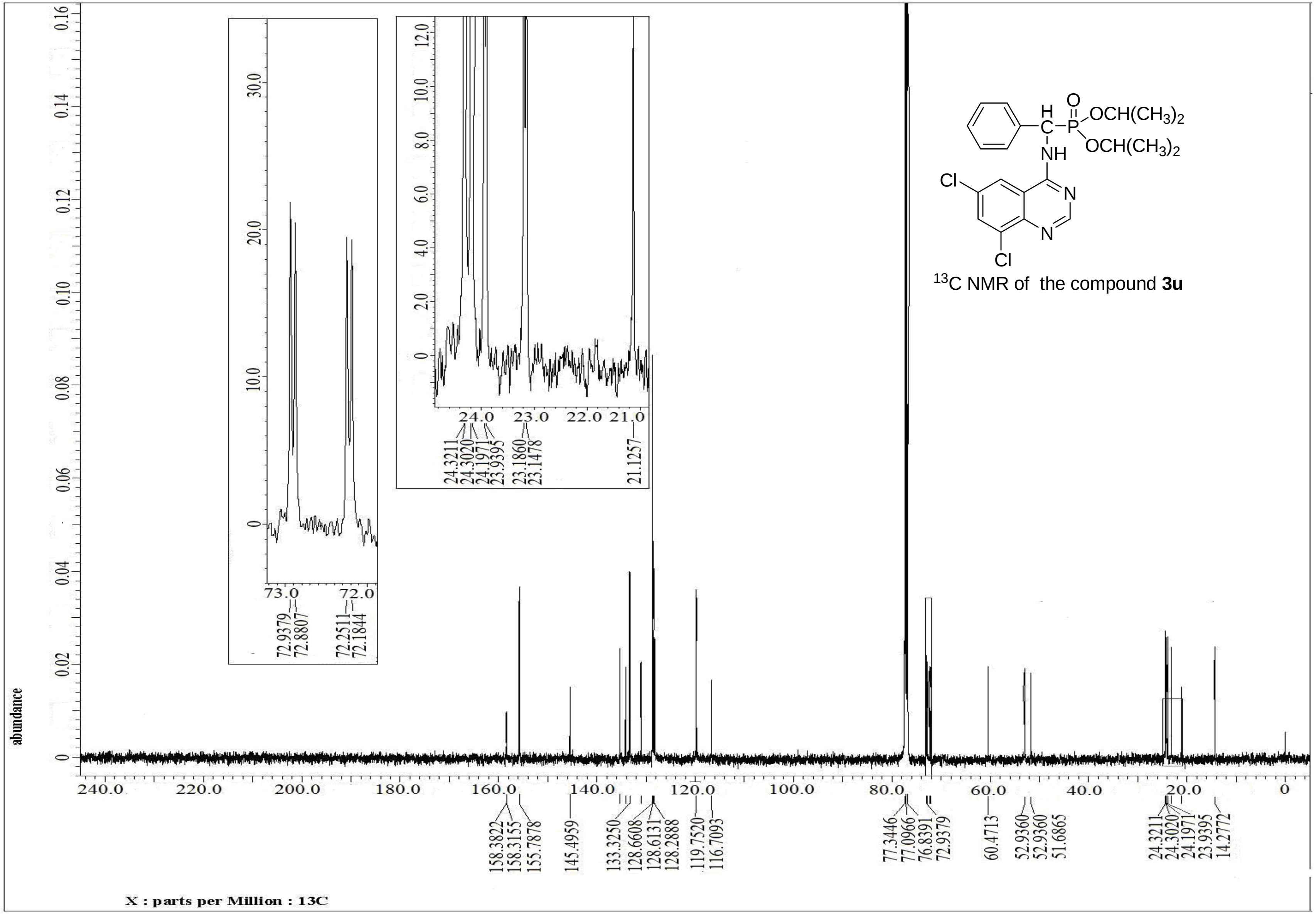

OCH(CH3)2
OCH(CH3)2
NH
Cl
N
N
Cl
13C NMR of the compound 3u
abundance
X : parts per Million : 13C
158.3822
158.3155
155.7878
145.4959
133.3250
128.6608
128.6131
128.2888
119.7520
116.7093
77.3446
77.0966
76.8391
72.9379
60.4713
52.9360
52.9360
51.6865
24.3211
24.3020
24.1971
23.9395
14.2772
72.9379
72.8807
72.2511
72.1844
23.1860
23.1478
21.1257

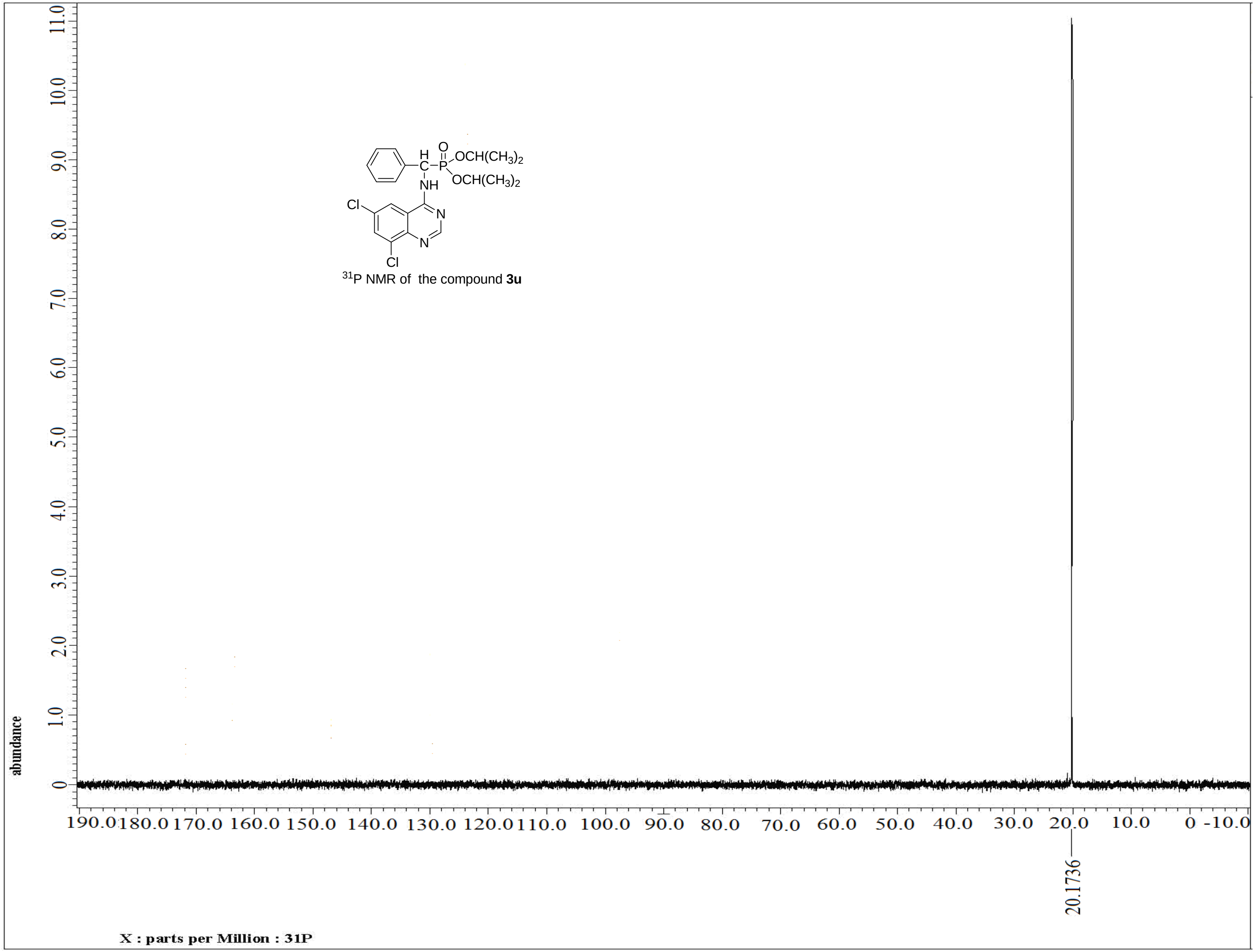
OCH(CH3)2
OCH(CH3)2
NH
Cl
N
N
Cl
31P NMR of the compound 3u
abundance
20.1736
X : parts per Million : 31P

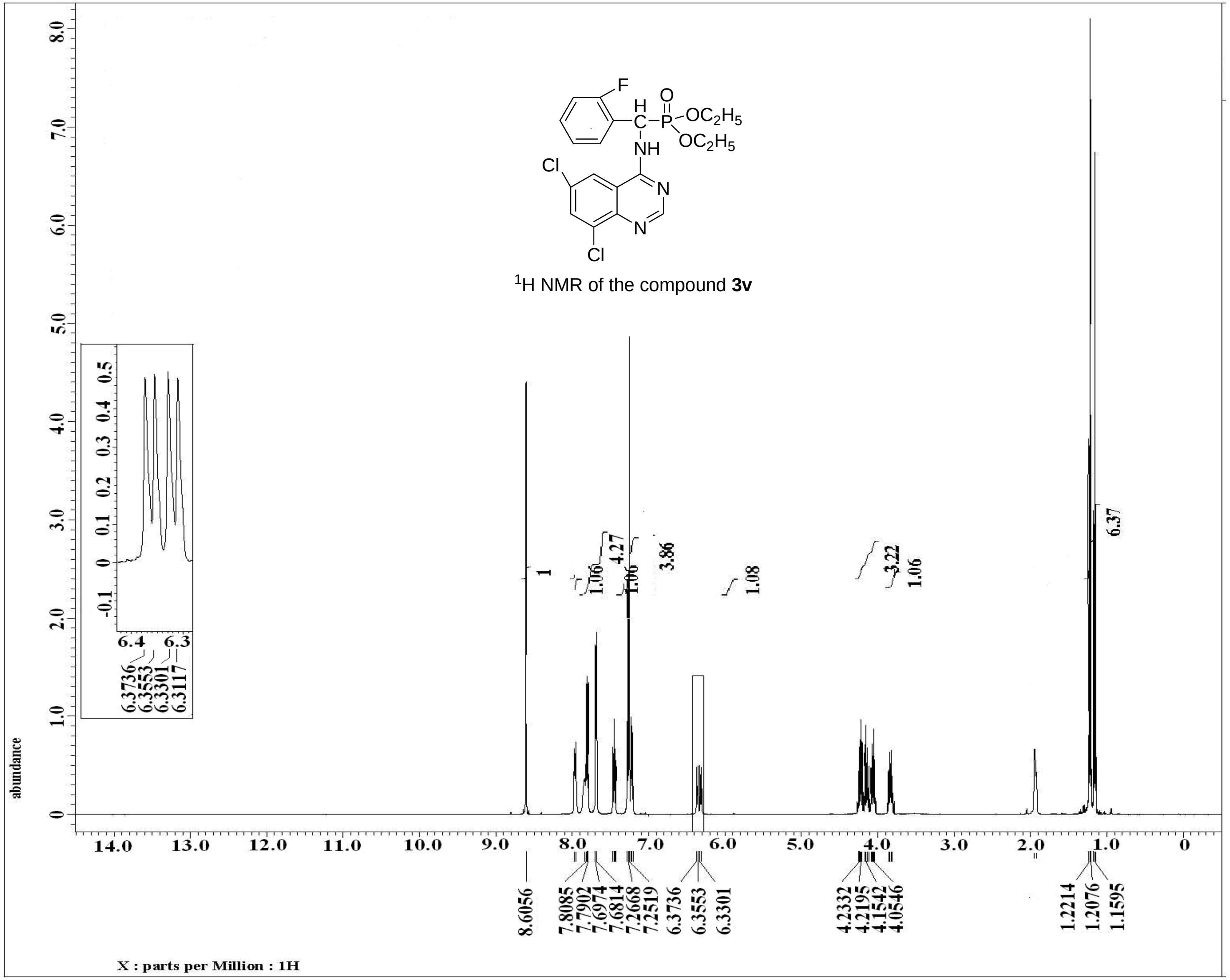

^{1}H NMR of the compound **3v**

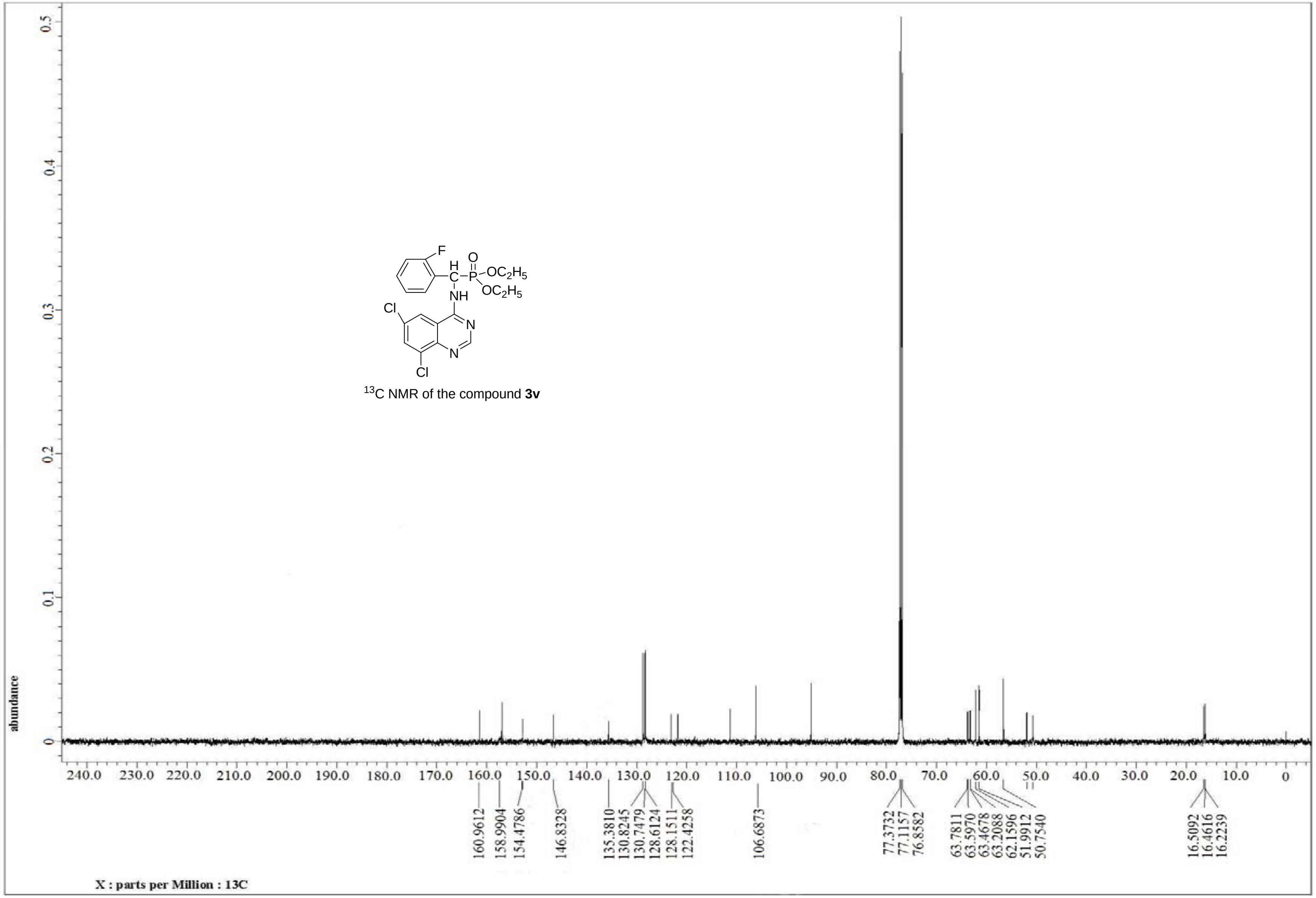

^{13}C NMR of the compound **3v**

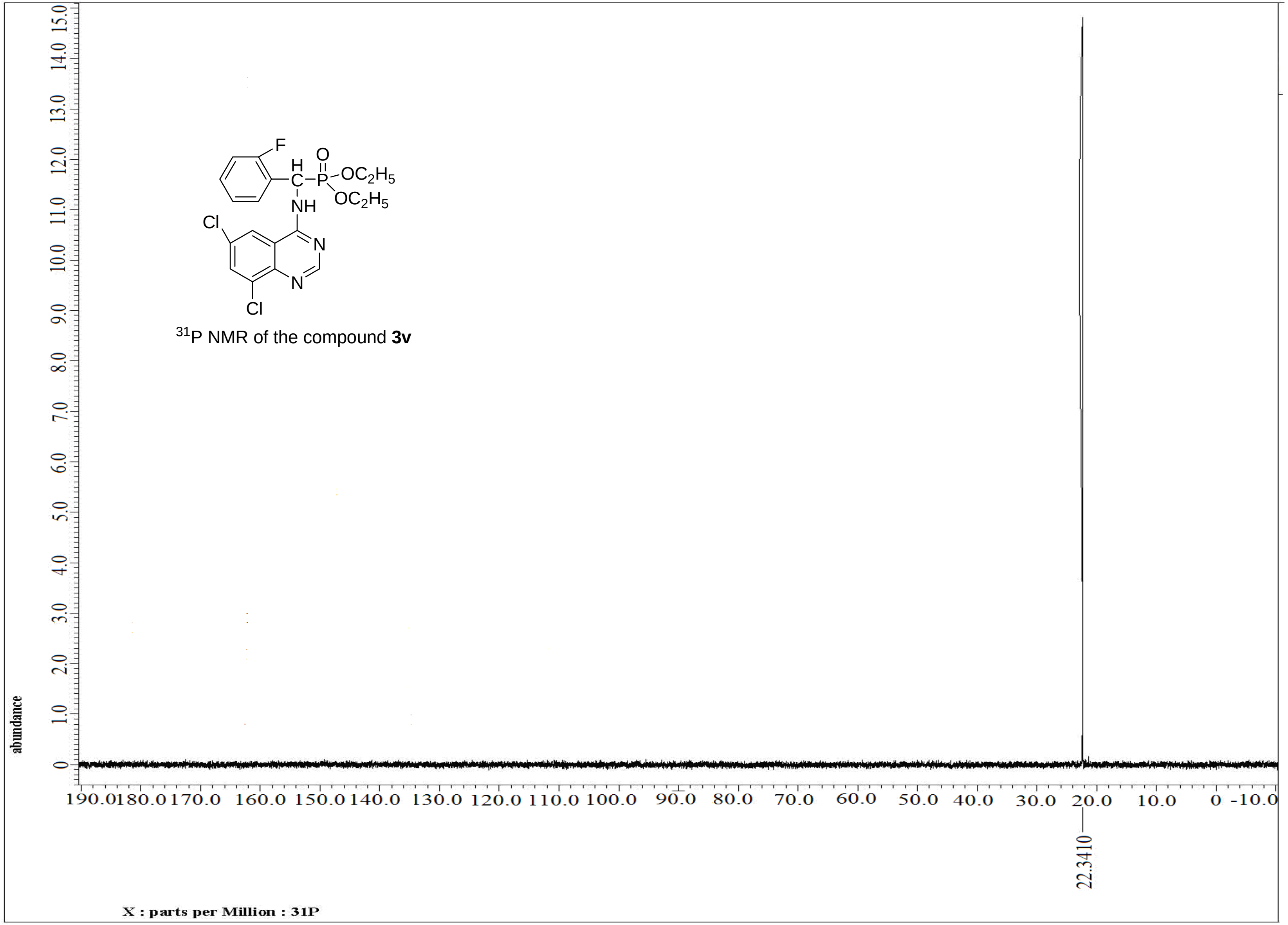

F
H
O
C
P
OC2H5
OC2H5
NH
Cl
N
N
Cl
^{31}P NMR of the compound **3v**
abundance
0
1.0
2.0
3.0
4.0
5.0
6.0
7.0
8.0
9.0
10.0
11.0
12.0
13.0
14.0
15.0
190.0180.0170.0 160.0 150.0140.0 130.0 120.0 110.0 100.0 90.0 80.0 70.0 60.0 50.0 40.0 30.0 20.0 10.0 0 -10.0
22.3410
X : parts per Million : 31P

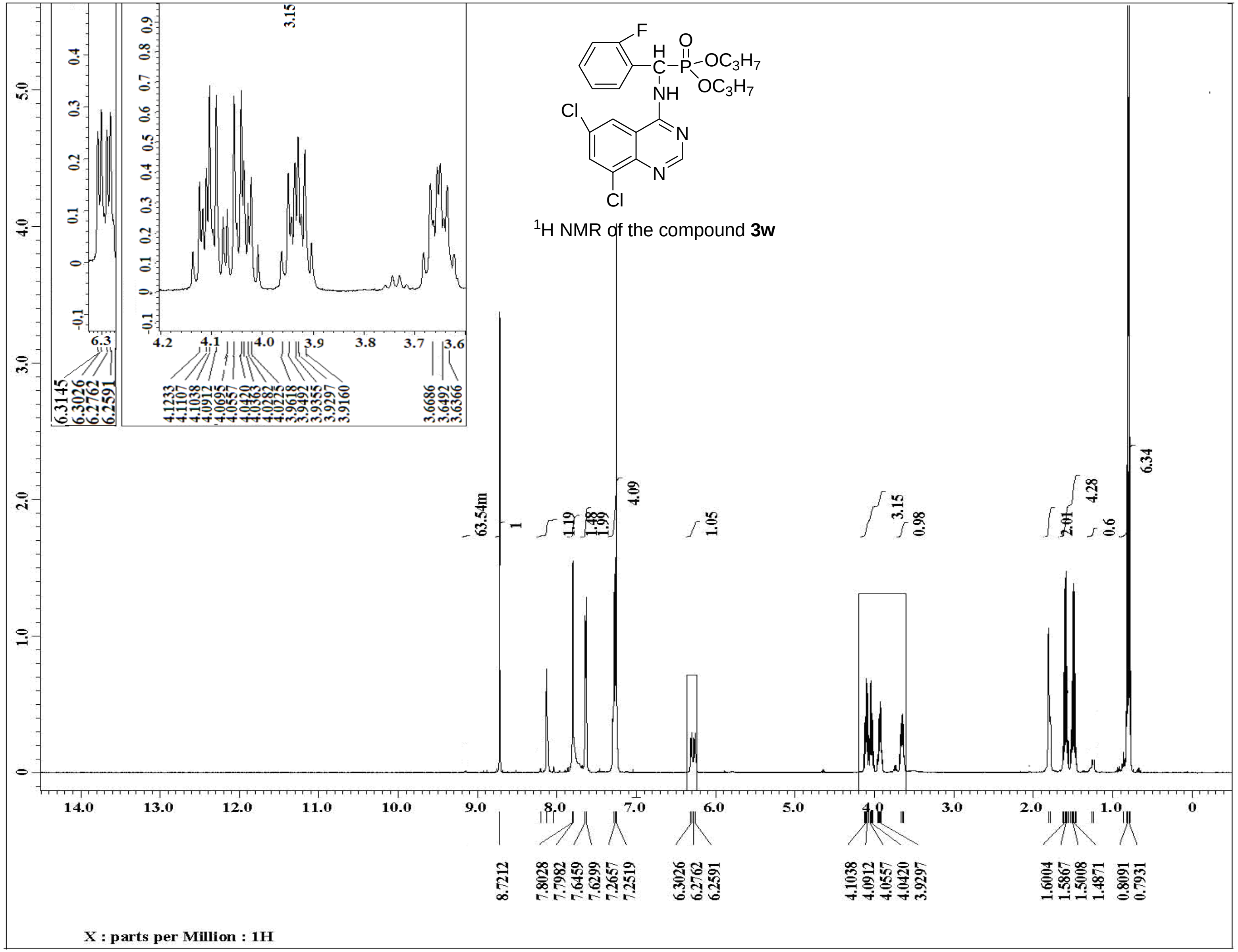

F
H
O
C
P
OC3H7
OC3H7
NH
Cl
N
N
Cl
1H NMR of the compound 3w
X : parts per Million : 1H

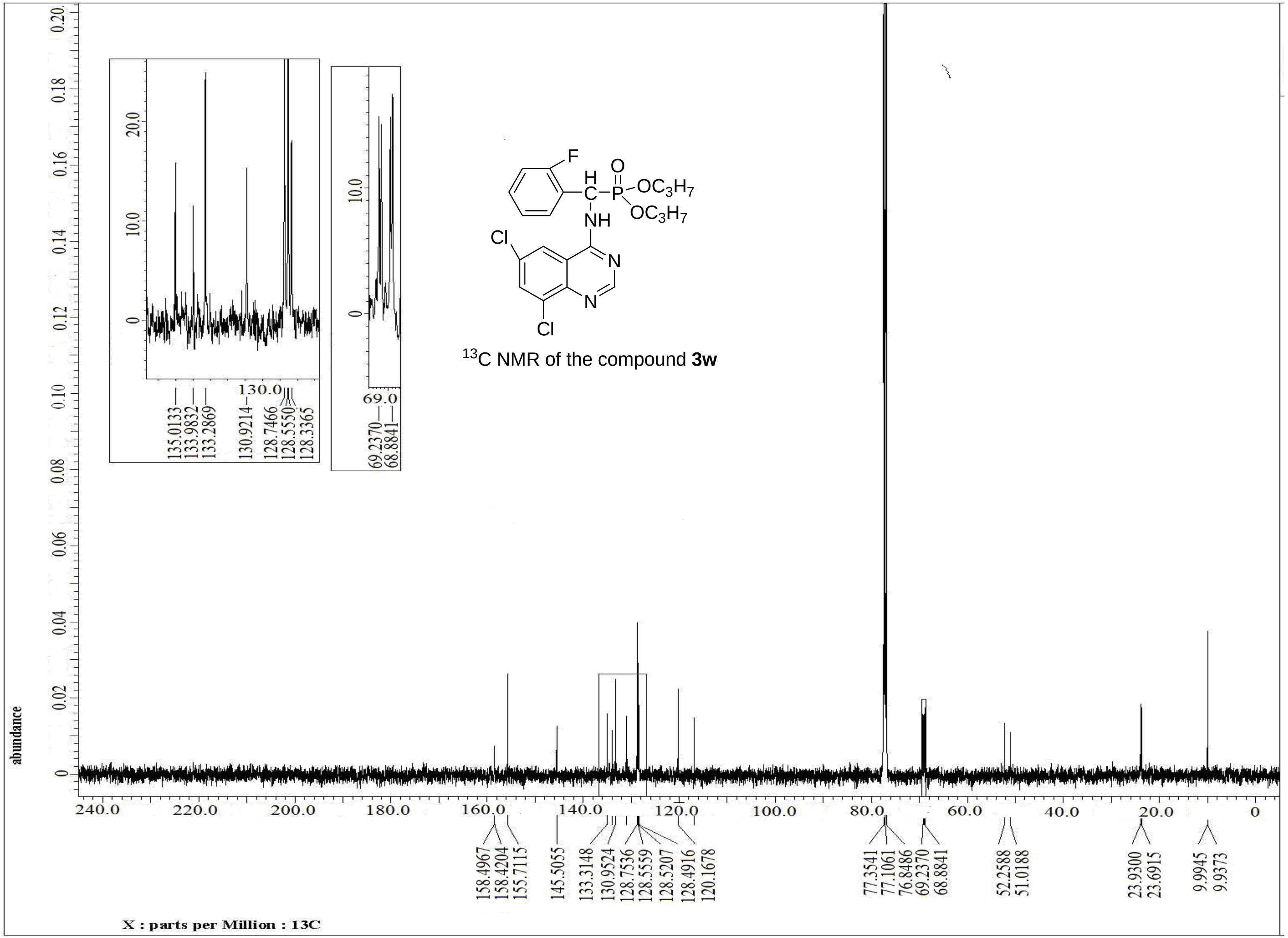

^{13}C NMR of the compound **3w**
abundance
X : parts per Million : 13C
158.4967
158.4204
155.7115
145.5055
133.3148
130.9524
128.7536
128.5559
128.5207
128.4916
120.1678
77.3541
77.1061
76.8486
69.2370
68.8841
52.2588
51.0188
23.9300
23.6915
9.9945
9.9373
135.0133
133.9832
133.2869
130.9214
128.7466
128.5550
128.3365
69.2370
68.8841

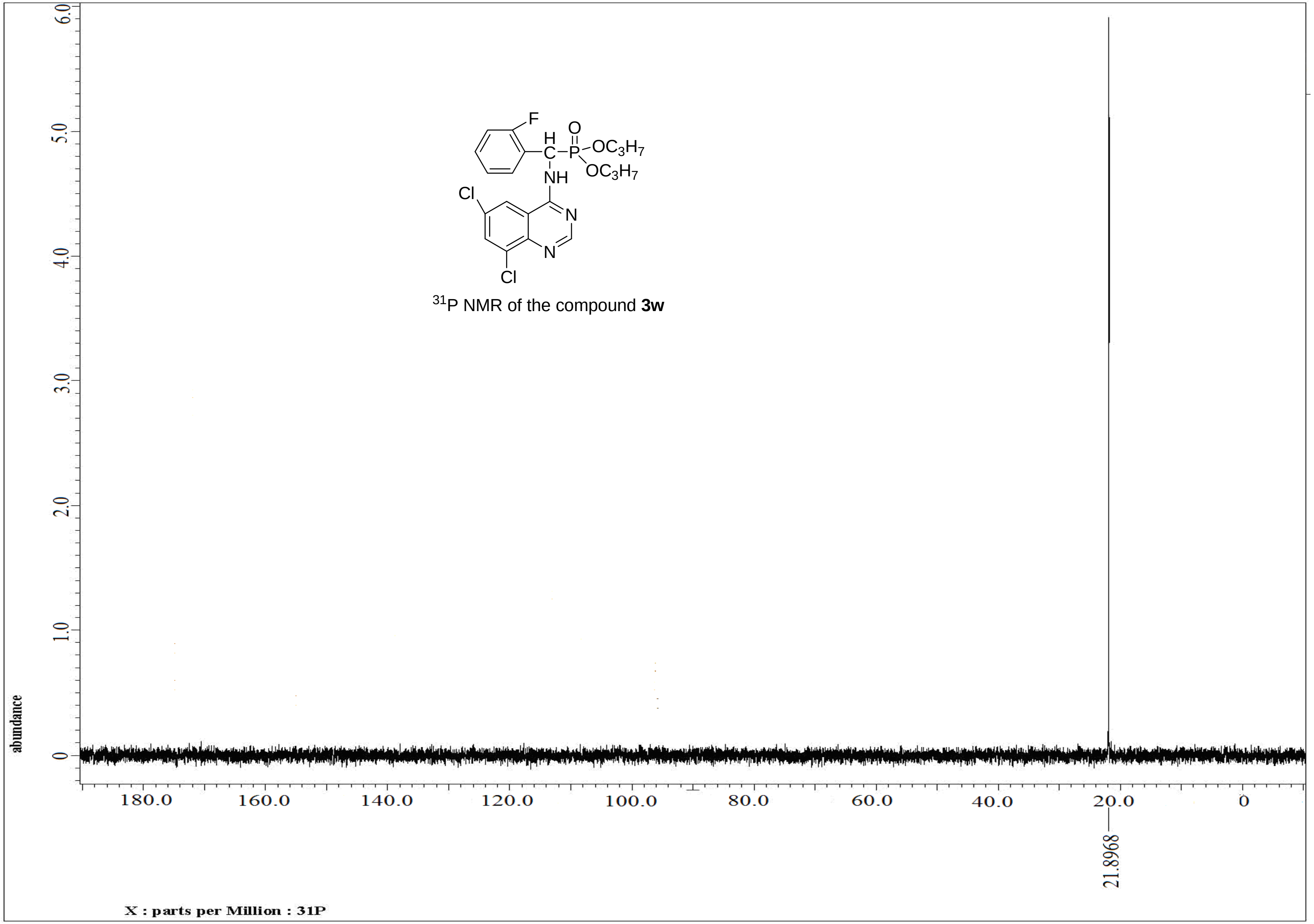

^{31}P NMR of the compound **3w**

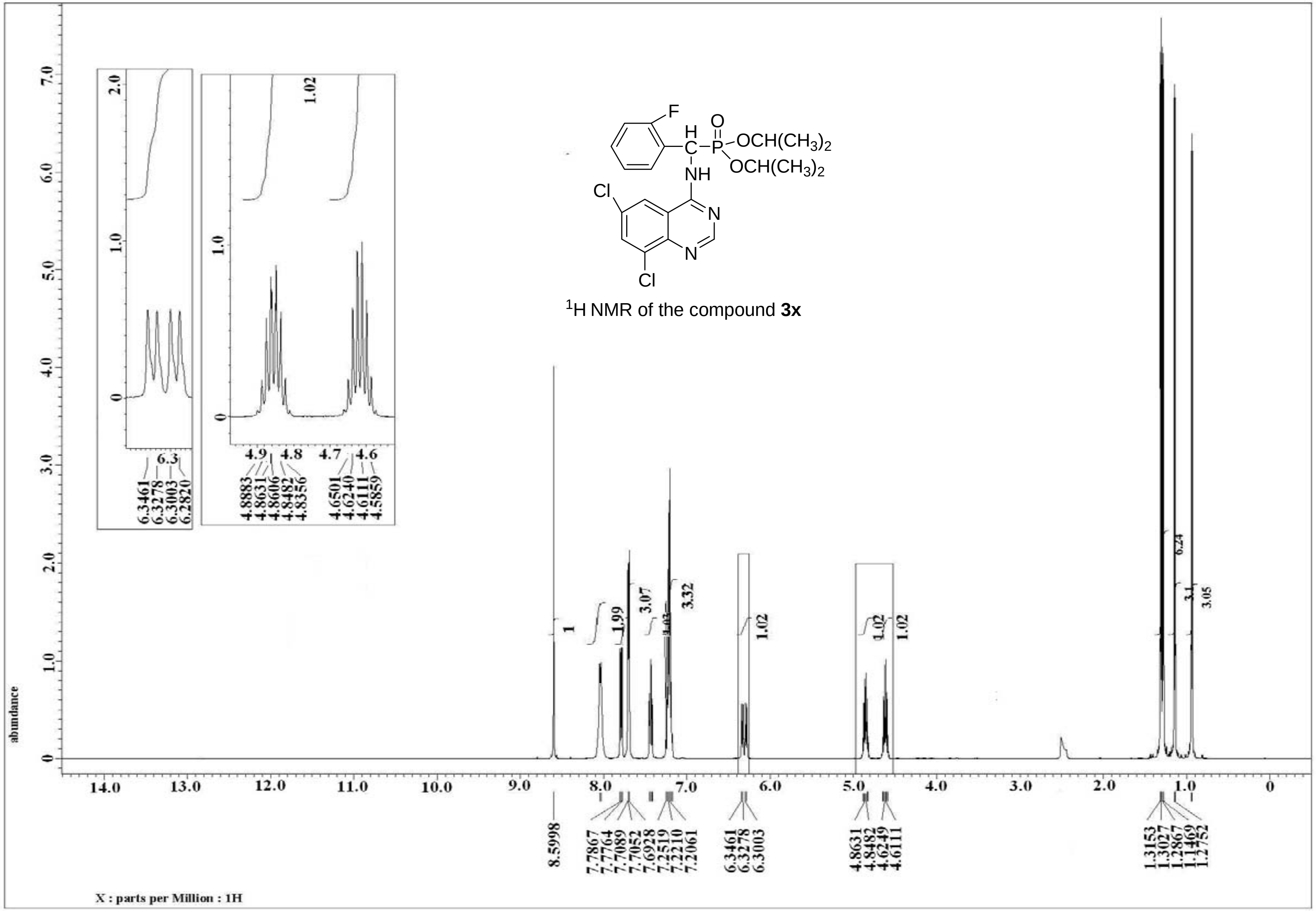

F
H
O
C
P
OCH(CH3)2
OCH(CH3)2
NH
Cl
N
N
Cl
1H NMR of the compound 3x
abundance
X : parts per Million : 1H

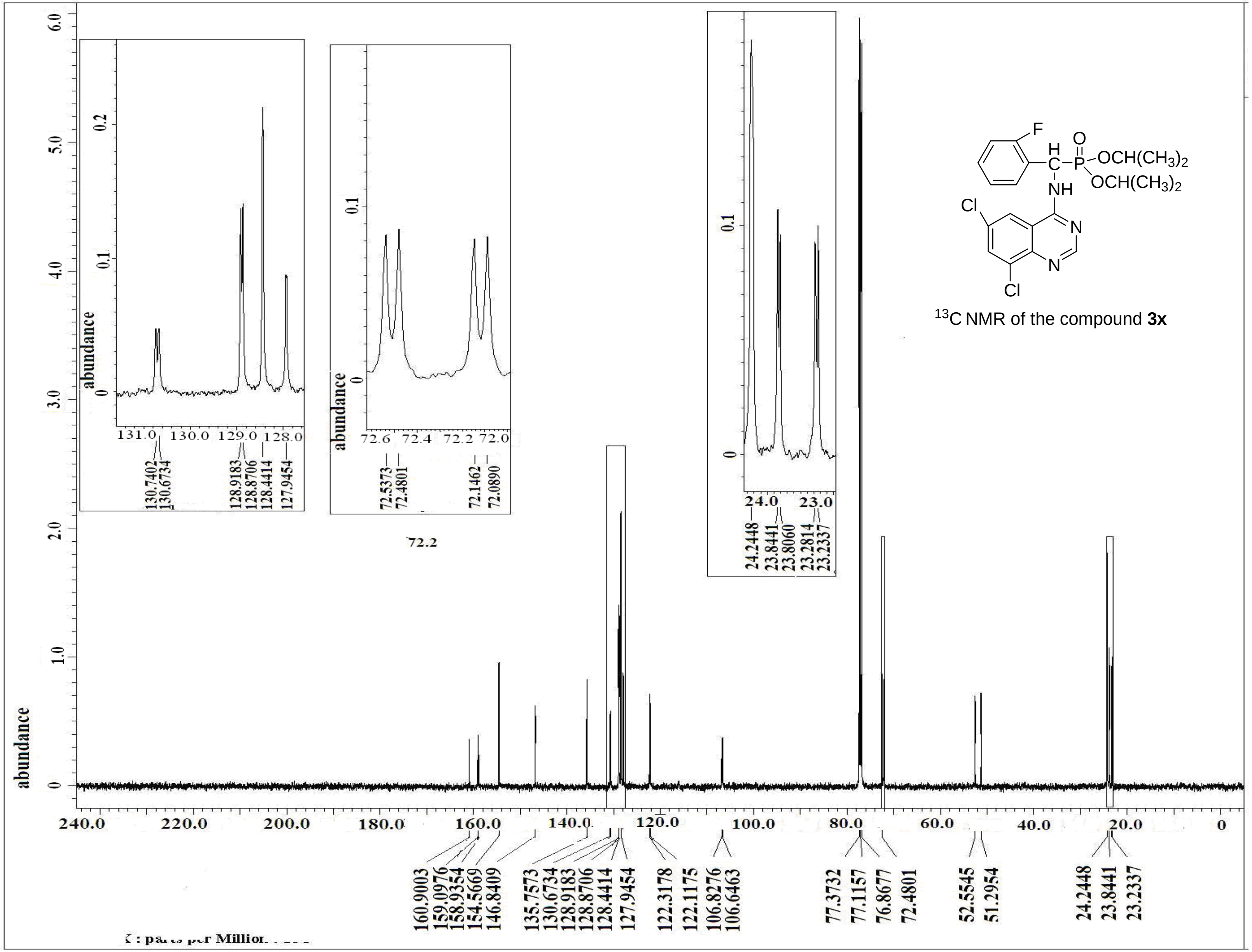

^{13}C NMR of the compound **3x**

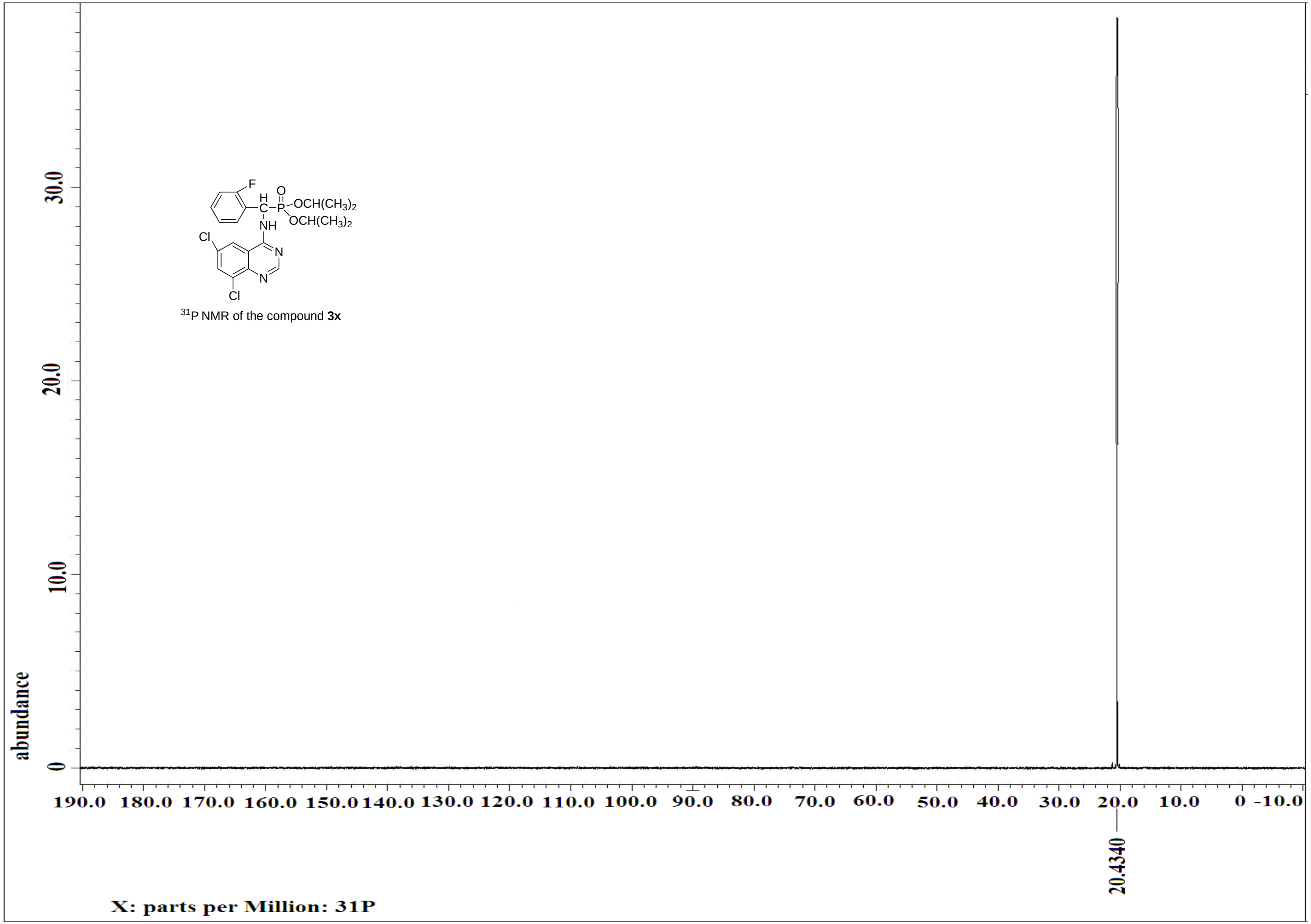
F
H
O
C
P
OCH(CH3)2
OCH(CH3)2
NH
Cl
N
N
Cl
31P NMR of the compound 3x
abundance
30.0
20.0
10.0
0
190.0 180.0 170.0 160.0 150.0 140.0 130.0 120.0 110.0 100.0 90.0 80.0 70.0 60.0 50.0 40.0 30.0 20.0 10.0 0 -10.0
20.4340
X: parts per Million: 31P
